# Elevated ozone activates maternal immunity to drive offspring IgG-mediated anaphylaxis through IgG glycosylation alterations

**DOI:** 10.1038/s41467-026-77756-x

**Published:** 2026-09-23

**Authors:** Xiaoyun Wu, Zhihua Gong, Yuchai Tian, Xiaomin Liang, Mudan Wu, Huizhen Zhu, Jiyue Zhang, Yangcheng Hu, Bin Li, Pannan Li, Xiaotong Ji, Huifeng Yue, Nan Sang

**Affiliations:** 1 Shanxi Key Laboratory of Coal-based Emerging Pollutant Identification and Risk Control, Research Center of Environment and Health, College of Environment and Resource, Shanxi University, Taiyuan, Shanxi PR China; 2 Shanxi Bethune Hospital, Shanxi Academy of Medical Sciences, Third Hospital of Shanxi Medical University, Tongji Shanxi Hospital, Taiyuan, Shanxi PR China; 3 Xiaodian District Maternal and Child Health Care Hospital, Taiyuan, Shanxi PR China; 4 Department of Environmental Health, School of Public Health, Center for Ecological Public Health Security of Yellow River Basin, Shanxi Key Laboratory of Environmental Health Impairment and Prevention, MOE Key Laboratory of Coal Environmental Pathogenicity and Prevention, Shanxi Medical University, Taiyuan, Shanxi PR China

**Keywords:** Environmental sciences, Risk factors, Antibodies, Allergy, Interleukins

## Abstract

Global allergic disease burden is rising, driven by environmental factors like ozone (O_3_)-induced oxidative stress. Epidemiological studies have linked prenatal oxidative stress to offspring allergy susceptibility, but causality remains unproven. Here, our retrospective analysis including 5,299 pregnancies postulates that mid-to-late gestational O_3_ exposure triggers maternal immune activation (MIA), increasing neutrophil/monocyte abnormality risks. Using an animal model of prenatal O_3_ exposure-induced MIA, we demonstrate that offspring of MIA dams—especially males—exhibit heightened allergy susceptibility. Mechanistically, MIA disrupts maternal-fetal IgG transfer and neonatal glycosylation, particularly *Mgat3*-mediated N-acetylglucosamine (GlcNAc) modifications. This perturbation preferentially activates juvenile male IL-17/TNF signaling. In adulthood, downstream transcription factors c-Jun/c-Fos activate *Mgat4a* to sustain GlcNAc glycosylation. This glycosylation of IgG is associated with enhanced IgG-FcγRIII affinity and more severe inflammatory responses during anaphylaxis. Our study bridges environmental epidemiology, immunology, and glycobiology to suggest how prenatal oxidative stress reprograms offspring immunity, offering insights into developmental origins of allergic diseases.

## Abstract (web summary)

Environmental pollution may have effects upon unborn children. Here the authors look at human retrospective data and use mouse models to show that ozone exposure during pregnancy can promote allergy susceptibility in adult offspring and this may involve changes in IgG glycosylation and increased inflammation.

## Introduction

The global prevalence of allergic diseases has escalated to alarming levels, with 30–40% of the world’s population currently affected, as reported by the World Allergy Organization^1^. Emerging evidence implicates dysregulated immune responses as a fundamental driver of allergic conditions^2,3^. The Developmental Origins of Health and Disease (DOHaD) hypothesis posits that adverse prenatal and early-life exposures exert enduring effects on immune system programming, i.e., awakening the memory of prior encounters incorrectly in response to subsequent challenges^4^. Notably, maternal immune activation (MIA) has been identified as a significant risk factor for immune homeostasis disruption in offspring^5,6^. Recent epidemiological and experimental studies further highlight oxidative stress as a key mediator in this process^7–9^. Ozone (O_3_), a potent source of oxidative stress, recent data indicate that 66.2% of the global population experiences short-term O_3_ exposure exceeding safety thresholds (>30 days per year), while 94.2% is subjected to long-term exposure^10^. But as a critical environmental risk factor triggering endogenous oxidative stress, the global health threats posed by current O_3_ pollution are likely underestimated in terms of immunological impact^10^. Partially, that is, whether O_3_ exposure can induce MIA and further lead to enhanced offspring allergic susceptibility remain fragmented.

During human gestation, the maternal-fetal dyad functions as an integrated immune unit, wherein maternal immunity transmitted to the fetus modulates the developmental programming of the newborn immune system^11^. At birth, the neonatal adaptive immune system is not yet fully mature; thus, antibodies in the offspring are primarily maternally derived immunoglobulin G (IgG), which crosses the placental barrier into fetal circulation during mid-to-late gestation in humans, while in mice, this transfer occurs via the yolk sac-placenta barrier^12,13^. Postnatally, infants continue to receive maternal IgG through breast milk^14^. In mice, this lactogenic IgG transfer, mediated by the neonatal Fc receptor (FcRn) in the intestine, continues until approximately postnatal day 16 (PND16), whereas in humans, it persists throughout breastfeeding, providing sustained mucosal protection^15,16^. As a pivotal component of neonatal immunity, maternally derived IgG engages innate immune cells to instruct functional responses, such as enhanced phagocytic capacity and broader immunomodulation^17,18^. Crucially, the immunological functions of IgG are dictated by its structural dynamics, which are finely regulated by glycosylation modifications. Glycosyltransferases modulate the biantennary core glycan of IgG by incorporating terminal residues (galactose, sialic acid) and structural modifications (bisecting N-acetylglucosamine (GlcNAc), core fucose), each conferring distinct functional properties. These glycosylation patterns critically influence IgG’s interactions with complement molecules and Fc receptors, positioning it as a critical regulator in balancing pro-inflammatory and anti-inflammatory immune responses^19^. Notably, chronic inflammatory conditions are characterized by dysregulated IgG glycosylation^20^. Clinical studies indicate that reduced IgG galactosylation contributes to chronic thromboembolic pulmonary hypertension (CTEPH) by promoting FcγR-dependent innate immune activation and pro-inflammatory signaling^21^. In patients with COVID-19, IgG lacking core fucosylation exhibits increased Fc gamma receptor (FcγR) IIIa affinity, thereby exacerbating antibody-dependent cytotoxicity and amplifying inflammation severity^22^. Therefore, the selective glycosylation of IgG may act as an initial molecular switch influencing different immune response outcomes in the juvenile offspring, significantly shaping the function of their immune system development and functional programming of their immune system. However, existing studies have predominantly focused on correlating IgG glycosylation patterns between MIA and offspring disease phenotypes^23,24^, whereas the precise mechanistic role and status of key glycosylation on IgG during postnatal development remains elusive.

The immune landscape in early life plays a pivotal role in shaping the developmental trajectory of allergic diseases throughout adulthood and across the lifespan^12^. Classic allergic responses are predominantly mediated by allergen-specific IgE binding to Fc epsilon receptor (FcεR) I receptors on mast cells, initiating histamine release^25^. However, Galli’s research revealed IgG-FcγR-granulocyte-platelet activation as an equally critical allergic pathway^26^. In IgG-mediated anaphylaxis, allergen-specific IgG binds to allergen to form immune complexes (IgG-ICs) that engage high-affinity FcγRs on myeloid cells, such as granulocytes and macrophages, triggering downstream inflammatory cascades^27^. Experimental evidence underscores the lung—characterized by its dense vascular network is the primary candidates for IgG-induced anaphylaxis represented by hypothermia and respiratory distress^28^. Moreover, immune activation has been closely linked to IgG-driven pulmonary allergic responses^29^. The regulatory role of glycosylated IgG in allergic diseases remains unexplored and requires further evidence^30^.

In this work, the correlation between hematological abnormalities in a retrospective mother-infant cohort and the daily O_3_ level is analyzed. In mouse models, gestational MIA is induced via O_3_ exposure at environmentally relevant concentrations from embryonic day 9.5 (E9.5) onward, allowing for an in-depth examination of how neonatal IgG glycosylation is rearranged and its subsequent impact on inflammatory pathways. Utilizing established allergic challenge models, long-term immune consequences in offspring are systematically evaluated, with a specific focus on elucidating the mechanistic role of glycosylated IgG in predisposing MIA progeny to anaphylaxis. These findings provide further insights into the immunological imprinting effects of oxidative stress-induced MIA.

## Results

### Prenatal O_3_ exposure induces MIA in human cohorts and mouse models

This study enrolled 5299 pregnant women (basic characteristics shown in Supplementary Table 1 and Supplementary Fig. 1). After adjusting for potential confounders, O_3_ exposure during pregnancy demonstrated a greater risk association with abnormal hematological inflammatory markers compared to other air pollutants (particulate matter (PM)_2.5_, PM_10_, sulfur dioxide (SO_2_), and nitrogen dioxide (NO_2_)) (Supplementary Tables 2–5). Window-specific analyses revealed significant associations between O_3_ exposure and abnormal risk during mid-to-late gestation, with the strongest effects observed in the third trimester. Each 10 μg/m^3^ increase in O_3_ concentration during mid-late gestation was associated with 2.0% (95% confidence interval (CI): 1.001–1.039) and 2.1% (95% CI: 1.000–1.041) elevated risks for neutrophil and monocyte abnormalities, respectively. In contrast, the same increment in O_3_ concentration during the first trimester was associated with odds ratios of 0.984 (95% CI: 0.972–0.996) for neutrophil and 0.982 (95% CI: 0.969–0.995) for monocyte abnormalities. Notably, the third trimester exposure specifically demonstrated a 3.0% increased risk of fibrinogen (FIB) abnormalities (95% CI: 1.010–1.050) (Table 1). As a supplementary finding, O_3_ exposure in the third trimester was also found to be significantly associated with an increased risk of hemoglobin (HGB) abnormality (OR = 1.030, 95% CI: 1.012–1.048 per 10 μg/m^3^ increment), with 4510 pregnant women exhibiting normal HGB levels (124.57 ± 8.88 g/L) and 789 exhibiting abnormal HGB levels (102.22 ± 7.21 g/L) (Table 1). Crucially, to verify the robustness of these primary findings, sensitivity analyses including multi-pollutant models and adjustment for maternal HGB levels yielded highly consistent effect estimates (Supplementary Tables 6–10). These findings suggest that gestational O_3_ exposure may induce MIA through systemic inflammatory responses, with mid-to-late pregnancy representing a critical exposure window.

**Table 1:** Trimester-specific associations between ambient O_3_ exposure and increased risks of hematological parameter abnormalities

	OR	95% CI	*p*		OR	95% CI	*p*
**WBC**							
Pre-T1	0.994	0.982–1.007	0.391	T3	0.990	0.978–1.003	0.136
T1	1.011	0.998–1.024	0.092	T2-T3	0.993	0.974–1.012	0.474
T2	1.004	0.990–1.018	0.568	T1-T3	1.021	0.976–1.069	0.371
**Neu**							
Pre-T1	0.998	0.986–1.011	0.807	T3	1.016	1.004–1.029	0.012*
T1	0.984	0.972–0.996	0.012	T2-T3	1.020	1.001–1.039	0.044*
T2	1.001	0.988–1.014	0.887	T1-T3	1.003	0.959–1.048	0.908
**Lym**							
Pre-T1	0.995	0.977–1.013	0.563	T3	0.986	0.967–1.004	0.127
T1	1.010	0.992–1.029	0.282	T2-T3	0.993	0.965–1.021	0.607
T2	1.001	0.999–1.003	0.374	T1-T3	1.016	0.952–1.085	0.629
**Mon**							
Pre-T1	1.005	0.992–1.018	0.458	T3	1.022	1.008–1.035	0.002**
T1	0.982	0.969–0.995	0.006	T2-T3	1.021	1.000–1.041	0.048*
T2	0.996	0.982–1.010	0.573	T1-T3	0.995	0.949–1.042	0.819
**Eos**							
Pre-T1	1.111	0.974–1.283	0.127	T3	0.979	0.853–1.120	0.761
T1	1.036	0.908–1.187	0.602	T2-T3	0.859	0.678–1.058	0.172
T2	0.885	0.749–1.024	0.118	T1-T3	0.693	0.419–1.109	0.135
**Bas**							
Pre-T1	1.017	0.949–1.089	0.632	T3	1.005	0.936–1.079	0.886
T1	1.009	0.940–1.082	0.812	T2-T3	0.976	0.876–1.086	0.652
T2	0.971	0.901–1.046	0.442	T1-T3	0.949	0.741–1.217	0.677
**FIB**							
Pre-T1	1.045	1.026–1.065	<0.001***	T3	1.030	1.010–1.050	0.003**
T1	0.983	0.964–1.002	0.078	T2-T3	0.990	0.961–1.019	0.498
T2	0.959	0.940–0.979	<0.001	T1-T3	0.897	0.838–0.960	0.002
**HGB**							
T3	1.030	1.012–1.048	0.001**				

Adjusted odds ratios (ORs) with 95% confidence intervals (CIs) were calculated for abnormalities in hematological parameters—including white blood cells (WBC), neutrophils (Neu), lymphocytes (Lym), monocytes (Mon), eosinophils (Eos), basophils (Bas), fibrinogen (FIB), and hemoglobin (HGB)—associated with each 10 μg/m^3^ increase in ambient O_3_ exposure, with covariate adjustments for maternal age, pre-pregnancy body mass index (BMI), education level, occupation, family income per person, folic acid supplementation, smoking, drinking, gravidity, parity, and previous adverse pregnancy.

Pregnancy stage definitions: Pre-T1: 12 weeks before pregnancy; T1: First trimester (weeks 1–12); T2: Second trimester (weeks 13–27); T3: Third trimester (weeks 28 until delivery); T2-T3: Mid-to-late pregnancy period (weeks 13 until delivery); T1-T3: Entire pregnancy period.

*indicates statistically significant risk factors (OR > 1, * *p* < 0.05, ** *p* < 0.01, *** *p* < 0.001).

**Fig. 1:**
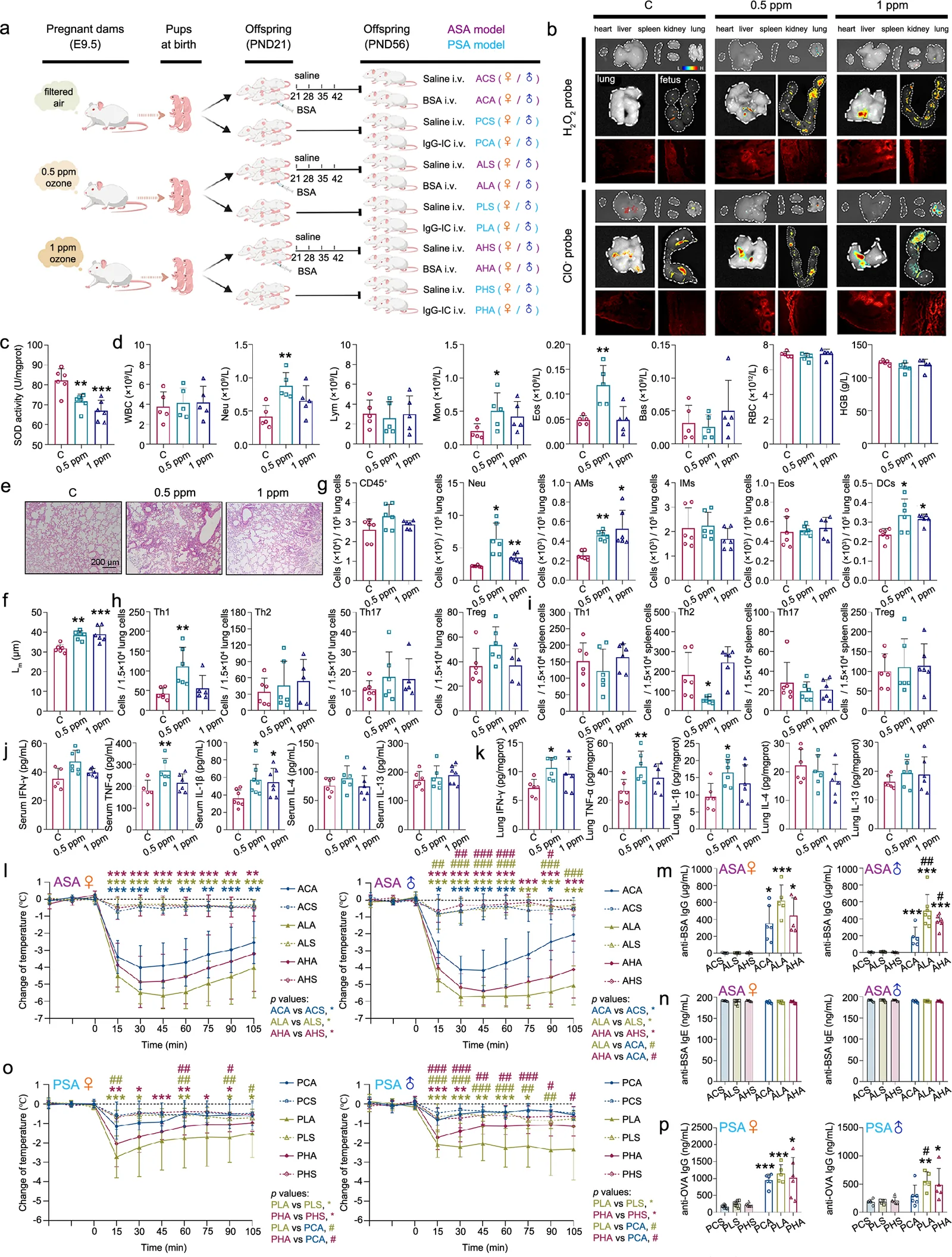
O_3_-induced maternal immune activation (MIA) increases offspring susceptibility to IgG-mediated anaphylaxis. **a** Experimental design of gestational O_3_ exposure and the subsequent offspring allergy models (created using BioGDP.com). **b** Maternal oxidative stress at E18.5 via fluorescence imaging using H_2_O_2_ and ClO^-^ probes. **c** SOD activity in maternal lungs (*n* = 6 mice/group). **d** Complete blood count (CBC) of maternal blood (*n* = 5 mice/group). **e**, **f** Representative H&E staining (scale bar, 200 μm) and mean linear intercept (L_m_) of maternal lungs (*n* = 6 mice/group). **g** Inflammatory cell counts in maternal lungs (*n* = 6 mice/group). T-cell subsets in maternal lungs (**h**) and spleen (**i**) (*n* = 6 mice/group; *n* = 5 mice for 1 ppm group in (**h**)). Cytokine levels in maternal serum (**j**) and lungs (**k**) (for (**j**), *n* = 5, 6, 7 mice/group with individual data points; *n* = 6 mice/group for (**k**)). Body temperature changes (**l**), serum anti-BSA IgG (**m**), and anti-BSA IgE (**n**) in ASA-challenged offspring (for (**l**), *n* = 11, 15, 10, 10, 14, 9 for females and *n* = 10, 10, 10, 8, 10, 11 for males; for (**m**), *n* = 5, 6, 6, 6, 5, 5 for females and *n* = 6, 6, 6, 5, 7, 6 for males; for (**n**), *n* = 6 mice/group; ordered by ACS, ALS, AHS, ACA, ALA, AHA). Body temperature changes (**o**) and serum anti-OVA IgG (**p**) in PSA-challenged offspring (for (**o**), *n* = 12, 14, 9, 11, 12, 10 for females and *n* = 9, 11, 9, 11, 11, 12 for males; for (**p**), *n* = 8, 7, 6, 6, 5, 6 for females and *n* = 6, 5, 5, 6, 5, 5 for males; ordered by PCS, PLS, PHS, PCA, PLA, PHA). Data are mean ± SD, analyzed by one-way ANOVA or Kruskal–Wallis test. In (**c**–**k**), *p < 0.05, **p < 0.01, ***p < 0.001 vs. control. In (**l**–**p**), *p < 0.05, **p < 0.01, ***p < 0.001 for challenge vs. saline; ^#^*p* < 0.05, ^##^*p* < 0.01, ^###^*p* < 0.001 for O_3_ vs. air allergy groups.

Given that mid-gestation coincides with fetal immune system development, we further established a gestational MIA model via O_3_ exposure from E9.5 to evaluate the impact of MIA on allergic responses in adult offspring (Fig. 1a). Maternal exposure to 0.5 ppm O_3_ induced transient weight loss on day 1, whereas 1 ppm O_3_ resulted in sustained weight loss and reduced dietary intake, with no significant differences in litter size or fetal weight among groups (Supplementary Fig. 2 and Supplementary Fig. 3). To assess systemic oxidative stress induced by O_3_ exposure, H_2_O_2_ and ClO^-^ probes were employed to quantify endogenous H_2_O_2_ and ClO^-^ levels in maternal organs at E18.5. As shown in Fig. 1b, O_3_-exposed dams exhibited significantly elevated pulmonary H_2_O_2_ and ClO^-^ levels compared to controls, whereas fetal and placental oxidative stress remained relatively lower, underscoring the heightened vulnerability of the maternal lung. Lung superoxide dismutase (SOD) activity declined to 0.87-fold and 0.81-fold of control levels following exposure to 0.5 ppm and 1 ppm O_3_, respectively (Fig. 1c). Given the well-established link between oxidative stress and inflammation, excessive H_2_O_2_ and HClO were likely drivers of inflammatory responses. Consistent with systemic inflammation, complete blood count analysis revealed significant increases in neutrophils, monocytes, and eosinophils in the 0.5 ppm exposure group compared to controls (Fig. 1d). Histopathological analysis revealed exacerbated pulmonary inflammation in O_3_-exposed dams, characterized by bronchial immune cell infiltration and airway mucosal thickening (Fig. 1e, f). Further characterization of maternal inflammation and lymphocyte subsets revealed that, in alignment with the observed inflammatory phenotype, both the 0.5 ppm and 1 ppm groups exhibited increased infiltration of neutrophils, alveolar macrophages, and dendritic cells, with the 0.5 ppm group likely associated with an expansion of pro-inflammatory Th1 (CD4^+^interferon-gamma (IFN)-γ^+^) cells (Fig. 1g, h). Additionally, splenic Th2 (CD4^+^ interleukin (‌IL)−4^+^) cells in dams exposed to 0.5 ppm O_3_ were reduced to 30% of control levels, further supporting the presence of a pro-inflammatory T cell profile (Fig. 1i). Consistent with this skewed T cell profile, assessment of inflammatory cytokines in maternal serum and lung homogenates at E18.5 showed that concentrations of the pro-inflammatory cytokines IFN-γ, TNF-α, and IL-1β were significantly elevated in the 0.5 ppm exposure group compared to controls. Notably, with the exception of a significant increase in serum IL-1β, these pro-inflammatory cytokines did not exhibit a similarly widespread elevation in the 1 ppm exposure group (Fig. 1j, k). Collectively, these results indicate that oxidative stress-induced immune activation was particularly pronounced following 0.5 ppm O_3_ exposure.

### O_3_-induced MIA increases susceptibility to IgG-mediated anaphylaxis in adult modeling offspring

To determine whether MIA increases the susceptibility of adult offspring to IgG-mediated anaphylaxis, both active systemic anaphylaxis (ASA) and passive systemic anaphylaxis (PSA) models were employed. These IgG-mediated anaphylaxis, driven by FcγR signaling, closely resemble allergic manifestations in humans^25^. In the ASA model, core body temperature measurements confirmed the successful establishment of IgG-mediated anaphylaxis models, as antigen-challenged offspring exhibited significantly greater temperature reductions compared to their respective solvent controls, regardless of maternal air or O_3_ exposure (ACA vs. ACS, ALA vs. ALS, AHA vs. AHS). Notably, in male offspring, prolonged hypothermia persisted in both ALA and AHA groups for 60 min post-challenge, with a maximum core body temperature decline of 6.27 °C in the ALA group, which failed to recover even after 105 min (Fig. 1l). Consistently, anti-bovine serum albumin (BSA) IgG levels in male offspring from the ALA and AHA groups were elevated 2.63-fold and 1.97-fold, respectively, compared to the ACA group (Fig. 1m). In contrast, no significant differences in anti-BSA IgE levels were observed among the groups (Fig. 1n). Similarly, following intravenous administration of an identical dose of OVA-IgG immune complexes, post-metabolic circulating anti-ovalbumin (OVA) IgG levels measured post-injection were 1.91-fold higher in the PLA group than in the PCA group (Fig. 1o, p), suggesting altered IgG-IC pharmacokinetics in these offspring. This might result from the remodeling of immune effector functions during embryonic development. Together, these results collectively suggest that MIA-exposed offspring exhibit heightened allergic sensitivity, with male offspring from the 0.5 ppm O_3_ exposure group being particularly susceptible.

### MIA enhances allergic lung inflammation in adult modeling offspring

**Fig. 2:**
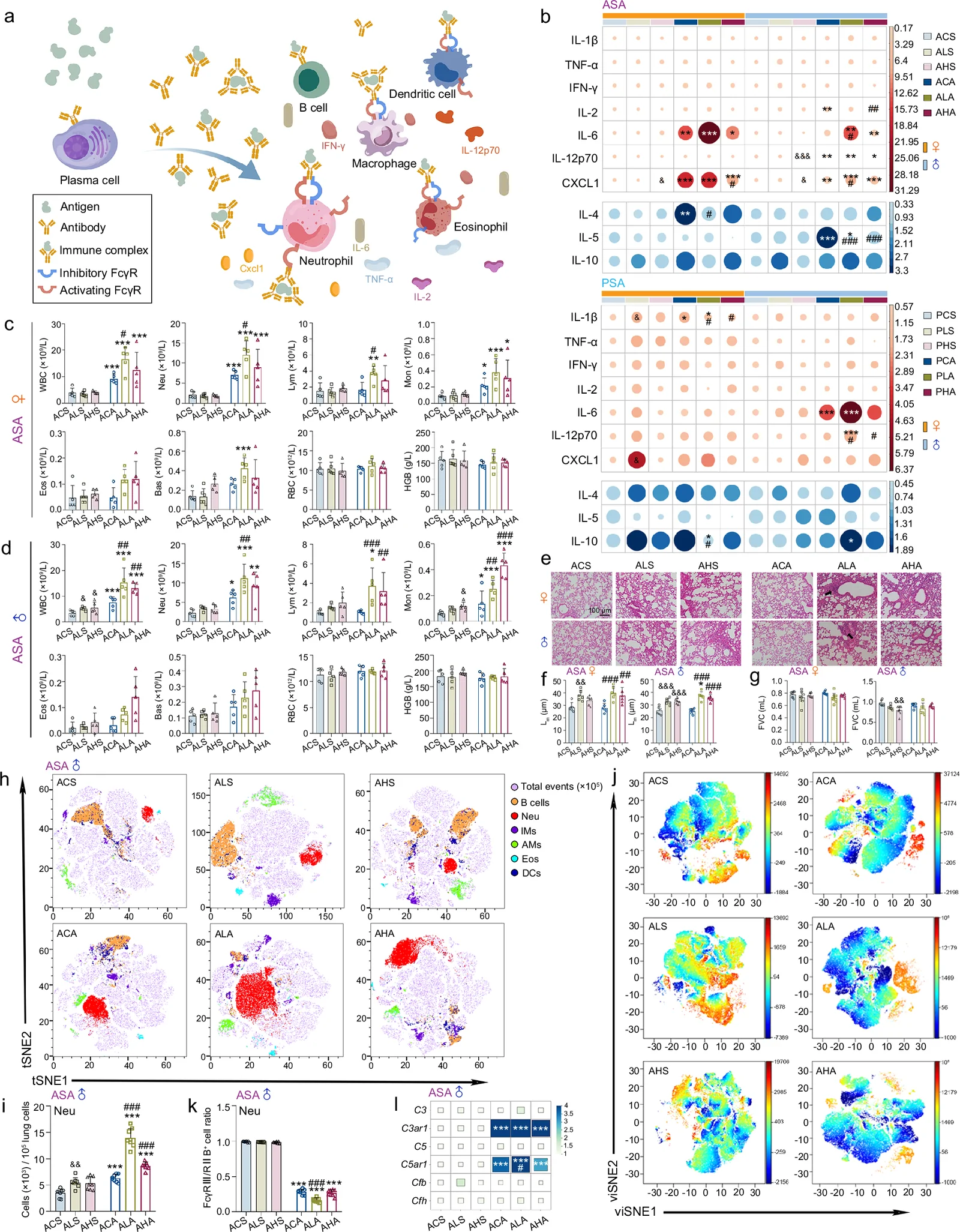
Inflammatory phenotypes induced by MIA in offspring mice with IgG-mediated anaphylaxis. **a** Regulatory mechanism of IgG-mediated immune responses. Allergen-IgG immune complexes engage activating or inhibitory Fcγ receptors (FcγRs) on effector cells, triggering anaphylaxis and promoting proinflammatory cytokine release (created using BioGDP.com). **b** Serum inflammatory cytokine profiles in ASA- and PSA-challenged offspring (*n* = 5–9 mice/group). CBC analysis in the ASA model (female (**c**) and male (**d**)) (*n* = 5, 6, 5, 5, 5, 5 for females and *n* = 5, 5, 5, 5, 5, 5 for males; ordered by ACS, ALS, AHS, ACA, ALA, AHA). **e**–**g** Pulmonary pathology and function in ASA models: representative H&E staining (black arrows indicate inflammatory foci; scale bar, 100 μm) (**e**), L_m_ (**f**), and forced vital capacity (FVC) (**g**) (for (**f**), *n* = 6 mice/group; for (**g**), *n* = 8, 8, 6, 7, 9, 6 for females and *n* = 6, 6, 7, 6, 6, 6 for males; ordered by ACS, ALS, AHS, ACA, ALA, and AHA groups). **h** t-SNE analysis of lung inflammatory cells in male ASA offspring. **i** Quantification of lung neutrophils in male ASA offspring (*n* = 8 mice/group). viSNE analysis of FcγRIII/FcγRIIB⁺ lung cells (**j**) and their neutrophil subset proportions (**k**) in male ASA offspring (*n* = 8 mice/group). **l** mRNA expression of complement-related genes in male ASA offspring lungs (*n* = 5–6 mice/group). Data are mean ± SD, analyzed by one-way ANOVA or Kruskal–Wallis test. *p < 0.05, **p < 0.01, ***p < 0.001 (ACA vs. ACS, ALA vs. ALS, AHA vs. AHS; PCA vs. PCS, PLA vs. PLS); ^#^*p* < 0.05, ^##^*p* < 0.01, ^###^*p* < 0.001 (ALA vs. ACA, AHA vs^.^ ACA; PLA vs. PCA, PHA vs. PCA); ^&^*p* < 0.05, ^& &^*p* < 0.01, ^&&&^*p* < 0.001 (ALS vs. ACS, AHS vs. ACS; PLS vs. PCS).

Anaphylaxis is frequently accompanied by inflammation, with cytokines playing a pivotal role in orchestrating immune responses and preventing excessive inflammatory damage (Fig. 2a). To evaluate cytokine alterations, serum profiling was conducted using Luminex. MIA exposure significantly increased IL-6 levels in male offspring from the ALA group, reaching 6.49-fold higher than ACA controls. Moreover, neutrophil chemoattractant CXCL1 was markedly upregulated, with a 2.09-fold increase in ALA males. In contrast, IL-4 levels in female offspring and IL-5 levels in male offspring from the ALA group were significantly reduced (Fig. 2b, Supplementary Fig. 4), indicating cytokine dysregulation and a disrupted Th1/Th2 balance in allergic offspring. Consistent with these cytokine alterations, complete blood count analysis revealed elevated leukocyte and differential counts (including neutrophils, lymphocytes, and monocytes) in both the ALA and AHA groups, further supporting a systemic inflammatory phenotype in MIA‑exposed offspring (Fig. 2c, d). Histopathological analysis of ASA model offspring revealed exacerbated pulmonary inflammation, characterized by inflammatory cell infiltration, alveolar wall destruction, and cavity fusion (Fig. 2e). These structural changes were accompanied by alveolar simplification (increased mean linear intercept, L_m_) and airway thickening (Fig. 2f, g and Supplementary Fig. 5). Similar pathological alterations were observed in PSA model offspring, including dysregulated inflammatory mediators and structural lung damage (Fig. 2b, Supplementary Figs. 6–9). These results suggest that oxidative stress-induced maternal immune alterations exert persistent effects on offspring, leading to cytokine imbalances and pulmonary injury, particularly males from 0.5 ppm O_3_-induced MIA.

To evaluate the inflammatory response in the lungs, flow cytometry was utilized to quantify immune cell populations involved in experimental anaphylaxis^28^. Allergen sensitization preferentially induced sustained neutrophil recruitment and activation in male offspring, with neutrophil levels in the ALA group increasing to 2.21-fold of the corresponding control (Fig. 2h, i and Supplementary Fig. 10). As key mediators of IgG-driven anaphylaxis, neutrophils are activated by deposited IgG-ICs, amplifying pre-existing inflammatory responses^31^. This mechanistic cascade further explains the heightened inflammation observed in the ALA group. FcγRIII serves as the primary receptor in IgG-mediated allergic responses, enabling immune cells to engage IgG-ICs and initiate downstream signaling. This response ability is believed to be regulated by the co-expressed inhibitory receptor FcγRⅡB^32^. To assess whether IgG-ICs accumulation in the lungs was dependent on FcγR-mediated immune cell activation, FcγRIII/FcγRIIB expression levels were examined. Compared to allergen-naive offspring, allergen-exposed mice exhibited a marked reduction in FcγRIII/FcγRIIB^+^ neutrophils, alveolar macrophages, interstitial macrophages, eosinophils, and dendritic cells (Supplementary Fig. 11). Notably, the downregulation of FcγRIII on neutrophils serves as a sensitive indicator of IgG-mediated allergic severity. Upon allergen challenge, FcγRIII/FcγRIIB expression on neutrophils in male offspring from MIA-exposed dams was significantly reduced compared to controls, with the ratio declining to 58.5% of ACA group levels in the ALA group (Fig. 2j, k). Given that IgG-ICs engagement with FcγR triggers complement activation, key complement components were assessed. Male offspring in the ALA group showed upregulated expression of complement component 5a receptor (*C5ar*) *1*, indicative of complement overactivation and exacerbated inflammatory responses (Fig. 2l and Supplementary Fig. 12). In the PSA model, neutrophils remained the predominant recruited cell type, with selective induction observed in the PLA group (Supplementary Fig. 13). These results collectively suggest that O_3_-induced MIA enhances susceptibility to IgG-mediated anaphylaxis in adult offspring by disrupting neutrophil recruitment and FcγRIII-mediated immune complex engagement, with male offspring exhibiting more pronounced symptoms.

### MIA alters maternal-fetal transmission of IgG with different glycosylation and reprograms inflammatory responses in juvenile offspring

**Fig. 3:**
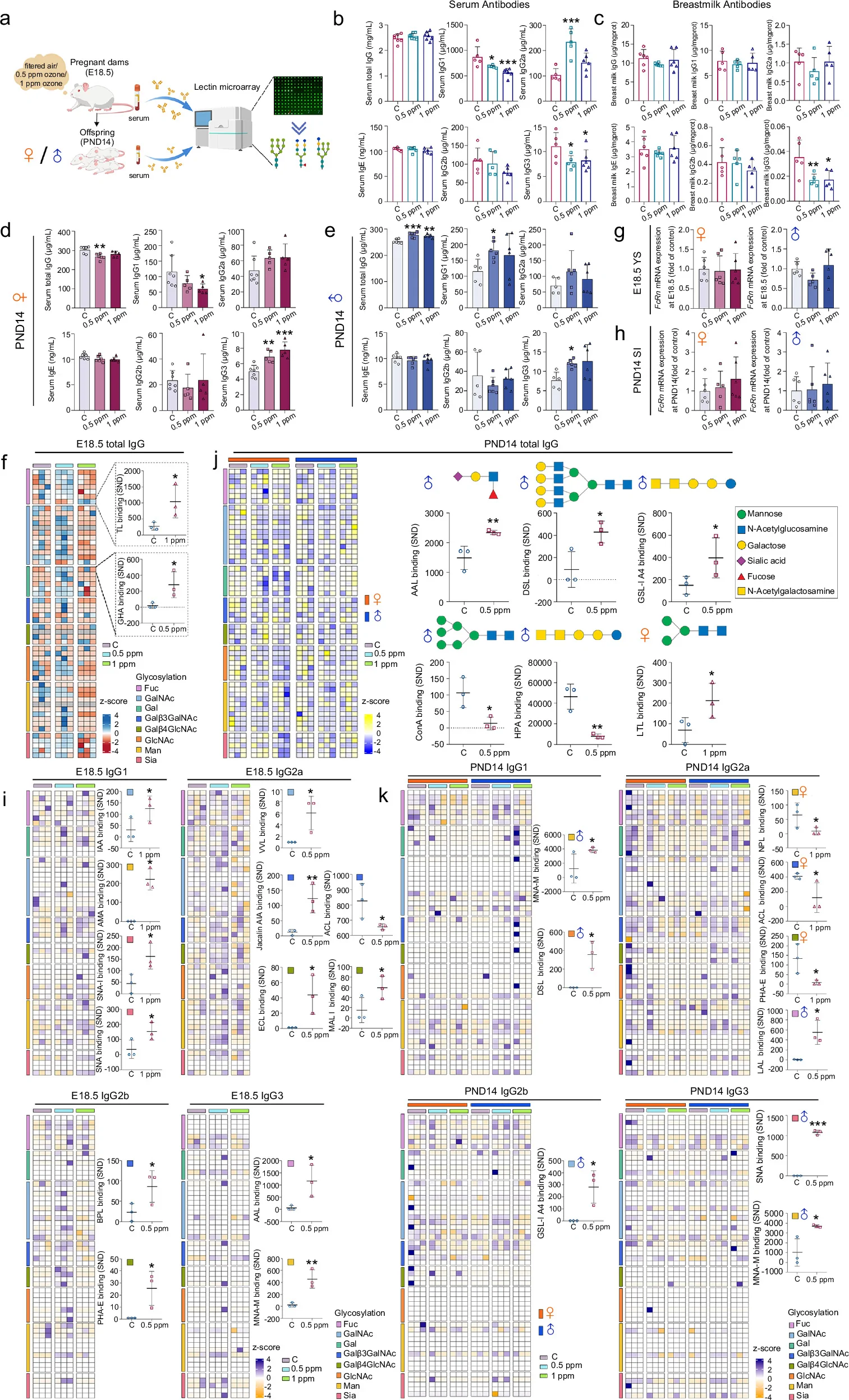
MIA disrupts maternal-fetal IgG transfer and reprograms neonatal IgG glycosylation profiles. **a** Lectin microarray analysis of IgG glycosylation profiles in maternal serum (E18.5) and offspring serum (PND14) at the end of maternal antibody transmission process (created using BioGDP.com). All samples were analyzed individually without pooling. Antibody levels (total IgG, IgG1, IgG2a, IgG2b, IgG3, and IgE) in maternal serum (**b**), breastmilk at PND14 (**c**), female offspring at PND14 (**d**), and male offspring at PND14 (**e**) (for (**b**), *n* = 7 for total IgG, *n* = 6 for IgE, and *n* = 5, 5, 6 for IgG subclasses; for (**c**), *n* = 6 for total IgG/IgE and *n* = 5 for IgG subclasses; for (**d**), *n* = 6, 6, 5 for total IgG, *n* = 6 for IgE, and *n* = 7, 5, 5 for IgG subclasses; for (**e**), *n* = 6, 6, 5 for total IgG, *n* = 6 for IgE, and *n* = 5, 6, 6 for IgG subclasses; ordered by control, 0.5 ppm, and 1 ppm groups). **f** Maternal serum IgG glycosylation profiles at E18.5. *FcRn* mRNA expression in yolk sacs (YS) at E18.5 (**g**) and neonatal small intestine (SI) at PND14 (**h**) (for (**g**), *n* = 6, 6, 6 for females and *n* = 5, 5, 6 for males; for (**h**), *n* = 6, 5, 6 for females and *n* = 6, 6, 6 for males; ordered by control, 0.5 ppm, and 1 ppm groups). **i** IgG subclass glycosylation profiles in maternal serum at E18.5. **j** Offspring serum IgG glycosylation profiles at PND14, showing representative structures with the highest binding affinity. **k** Offspring IgG subclass glycosylation profiles at PND14. For (**f**, **i**–**k**), the dot plots display differential lectin binding relative to corresponding controls (*n* = 3 mice/group). SND represents the signal strength detected by the lectin microarray assay. Data are mean ± SD, analyzed by one‑way ANOVA or Kruskal–Wallis test for (**b**–**e**) and (**g**, **h**), and by one‑tailed Student’s *t* test for (**f**) and (**i**–**k**). *p < 0.05, **p < 0.01, and ***p < 0.001 compared with the corresponding control group.

We further investigated whether IgG-mediated anaphylaxis susceptibility in offspring could be traced back to MIA during gestation (Fig. 3a). Maternal IgG crosses the yolk sac-placenta barrier via FcRn binding during late mouse gestation and continues to transfer postnatally through intestinal FcRn-mediated uptake from milk until PND14, thereby establishing neonatal humoral immunity before autonomous antibody production commences^33^. Notably, the level and glycosylation status of maternal IgG significantly influence its binding kinetics and affinity for FcRn^34^. We first assessed the impact of MIA on maternal and neonatal IgG levels. Total maternal IgG levels at E18.5 remained unchanged in MIA dams, but a significant rearrangement of the IgG subclass composition was uncovered. Specifically, IgG1 and IgG3 levels were significantly reduced in both the 0.5 and 1 ppm O_3_ induced MIA groups, while IgG2a levels were elevated in the 0.5 ppm group (Fig. 3b). This reconfigured maternal antibody profile was transmitted to the offspring, manifesting as sex-specific alterations in neonatal IgG levels: female offspring exposed to O_3_ exhibited decreased serum IgG at the completion of maternal antibody transfer (PND14), while male offspring showed an elevation in IgG levels compared to controls (Fig. 3d, e). Subclass analysis confirmed that the elevated total IgG in males was predominantly driven by an expansion of IgG1, whereas the reduction in females was associated with a decrease in IgG1. Notably, we did not detect significant differences in IgG levels in breast milk (Fig. 3c).

To explore the underlying mechanism, we next analyzed maternal IgG glycosylation. As illustrated in Fig. 3f, the glycan structures of maternal IgG were classified into multiple clusters. Detailed fluorescence analysis revealed that total IgG from MIA dams exhibited significantly stronger binding to GHA (0.5 ppm) and TL (1 ppm) lectins, indicating increased galactose and fucose relative levels relative to controls, respectively. We hypothesized that these glycosylation changes might affect FcRn-mediated transfer. To test this directly, we performed triple immunofluorescence co-staining of yolk sac tissue at E18.5 using lectins (GHA/TL) to track glycan-altered IgG, together with antibodies against total IgG and FcRn. The results showed that IgG carrying these specific glycan modifications exhibited higher colocalization with FcRn in yolk sacs of male fetuses compared to females, providing evidence that MIA-induced glycosylation remodeling differentially regulates IgG-FcRn interaction at the maternal-fetal interface (Supplementary Fig. 14). The unchanged expression of FcRn in the maternal yolk sac and offspring intestines (Fig. 3g, h) further supported that altered transfer efficiency was mediated by IgG glycosylation rather than receptor abundance.

Subclass-specific analysis revealed that in the 0.5 ppm O_3_-induced MIA group, the pro-galactosylation phenotype was primarily driven by elevations of Galβ4GlcNAc (bound by lectins ECL and MAL I) and Galβ3GalNAc (Jacalin AIA) on IgG2a, alongside increases in the same structures on IgG2b (PHA-E and BPL). In contrast, the 1 ppm group induced a distinct pattern, characterized by elevated sialylation (SNA and SNA-I), mannosylation (AMA), and GalNAc (IAA) specifically on the IgG1 subclass (Fig. 3i). Thus, while total maternal antibody levels remained unchanged, the altered abundance of IgG1 and IgG2a/b subclasses, together with their distinct glycosylation profiles, shaped the differences in the functional propensity transferred to offspring. At PND14, male offspring from the 0.5 ppm O_3_-induced MIA group exhibited marked glycosylation differences in total IgG. Specifically, the abundances of Fucα6GlcNAc (AAL), GlcNAc (DSL), and α-GalNAc (GSL-IA4) were significantly elevated, with levels reaching 1.58-, 4.63-, and 2.66-fold higher than those in the corresponding control groups. In contrast, glycans such as αMan and αGlc (ConA) and GalNAc (HPA) were substantially reduced, present at only 12.6% and 17.1% of control levels, respectively (Fig. 3j). Crucially, subclass-specific profiling demonstrated that this pro-inflammatory landscape was distributed across different subclasses: the elevated GlcNAc (DSL) was precisely localized to the expanded IgG1 pool, while the increased GalNAc (GSL-IA4) was enriched on IgG2b. These pro-inflammatory modifications, combined with the expansion of the IgG1 subclass, collectively drove the pro-inflammatory state in male offspring; meanwhile, an increase in fucosylation (LAL) with anti-inflammatory potential was observed on the effector subclass IgG2a. Furthermore, female offspring from the MIA (1 ppm O_3_) group displayed a predominant fucosylation signature in total IgG characterized by αFuc (LTL). Supporting a robust anti-inflammatory bias, subclass profiling in females revealed a significant reduction in pro-inflammatory mannose (NPL) structures specifically on the IgG2a subclass (Fig. 3k).

**Fig. 4:**
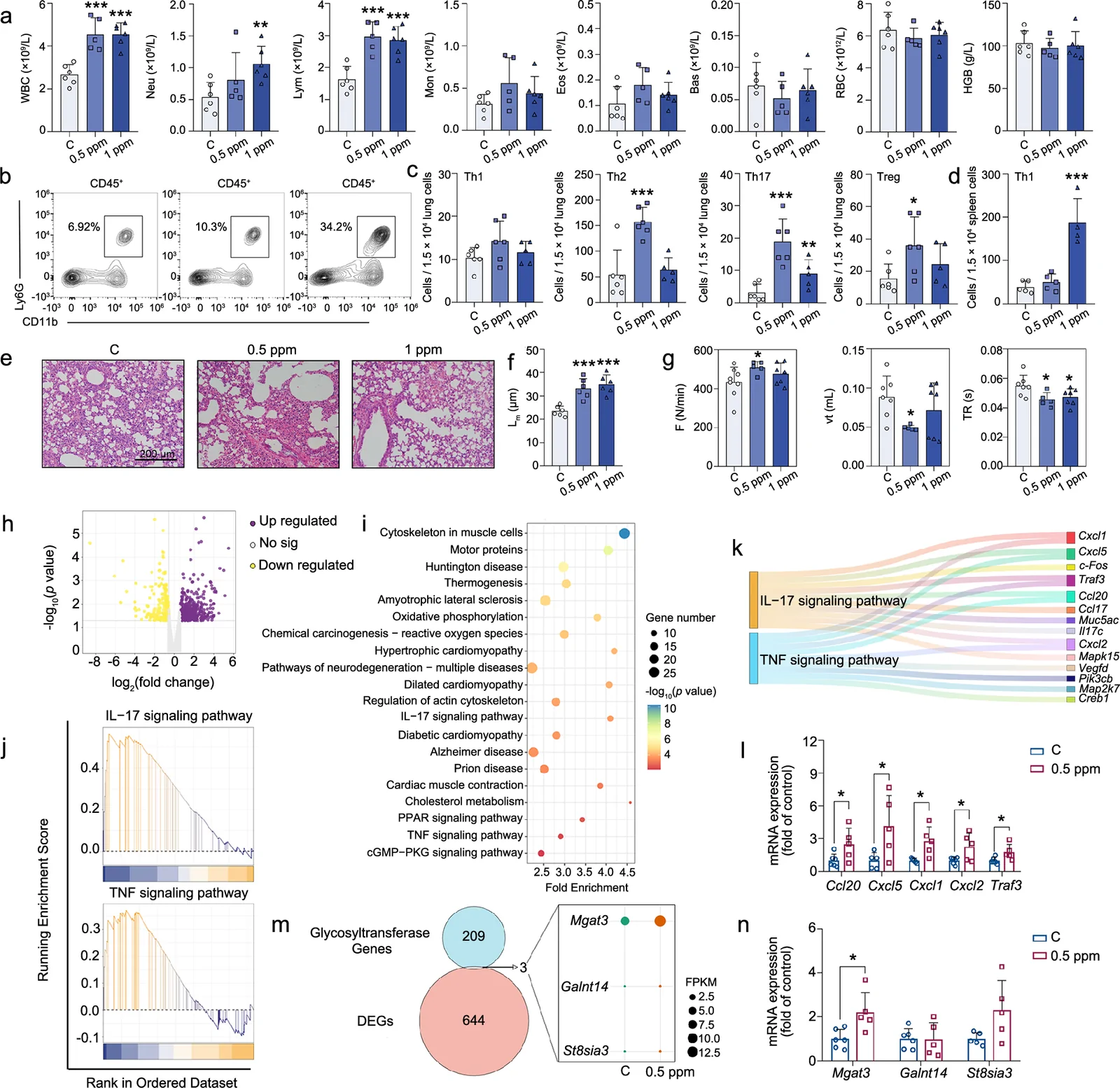
Altered IgG glycosylation affects inflammatory phenotypes in juvenile male offspring (PND14). **a** CBC analysis in male offspring (*n* = 6, 5, 6, ordered by control, 0.5 ppm, and 1 ppm groups). **b** Representative flow cytometric plots of lung neutrophils in male offspring. **c** T-cell subsets in male offspring lungs (*n* = 6, 6, 5, ordered by control, 0.5 ppm, and 1 ppm groups). **d** Th1 cell counts in male offspring spleens (*n* = 5, 5, 4, ordered by control, 0.5 ppm, and 1 ppm groups). Representative H&E staining (scale bar, 200 μm) (**e**) and L_m_ (**f**) in male offspring lung tissues (*n* = 6 mice/group). **g** Pulmonary function (respiratory frequency (F), tidal volume (vt), and total respiratory cycle time (TR)) analysis of male offspring (for (**f**), *n* = 8, 5, 7; for vt and TR, *n* = 7, 5, 7; ordered by control, 0.5 ppm, and 1 ppm groups). **h** Volcano plot of lung mRNA expression in male offspring (offspring from 0.5 ppm O_3_-induced MIA vs. controls) analyzed by two-sided exact tests, showing significantly upregulated (purple), downregulated (yellow), and nonsignificant genes (gray) (*n* = 3 mice/group). **i** Top 20 enriched KEGG pathways from lung differentially expressed genes (DEGs) calculated via one-sided hypergeometric tests. **j** GSEA showing the activation of IL-17 and TNF signaling pathways. **k** DEGs overlapping IL-17 and TNF signaling pathways. **l** qPCR validation of five DEGs involved in both IL-17 and TNF signaling pathways (for *Ccl20*, *Cxcl2*, and *Traf3*, *n* = 6, 5; for *Cxcl5* and *Cxcl1*, *n* = 5, 5; ordered by control and 0.5 ppm groups). **m** Intersection of 647 DEGs with 212 glycosyltransferase genes, identifying three hub genes. **n** qPCR validation of the three glycosyltransferase genes (for *Mgat3* and *Galnt14*, *n* = 6, 5; for *St8sia3*, *n* = 5, 5; ordered by control and 0.5 ppm groups). Data are mean ± SD. analyzed by one‑way ANOVA for (**a**–**d**) and (**f**, **g**), and by two‑tailed Student’s *t* test for (**l**) and (**n**). *p < 0.05, **p < 0.01, and ***p < 0.001 compared with the corresponding control group.

To investigate the consequences of altered IgG glycosylation profiles, inflammatory cell populations in serum and lung tissues of offspring at PND14 were examined. Significant elevations in white blood cells, lymphocytes, and neutrophils were observed in MIA male offspring, accompanied by increased neutrophil infiltration in the lungs (Fig. 4a, b). Building on these observations, the impact of glycosylated IgG on T cell priming and immune modulation was further assessed. Male offspring with altered IgG glycosylation profiles (from 0.5 ppm O_3_-induced MIA) exhibited concurrent activation of pulmonary Th17, Th2, and Treg cells, while those from the 1 ppm O_3_-induced MIA group demonstrated only enhanced pulmonary Th17 and splenic Th1 cell activation, supporting the hypothesis that maternally derived pro- and anti-inflammatory glycosylated IgG synergistically shape the immune priming phenotypes in offspring (Fig. 4c, d). Additionally, the accumulation of inflammatory signaling exacerbated lung injury in juvenile offspring, characterized by alveolar enlargement, thinning of alveolar walls, and a significant increase in L_m_ (Fig. 4e, f). These structural alterations were accompanied by functional impairments, including reduced tidal volume (vt), shortened total respiratory cycle time (TR), and increased respiratory frequency (F) (Fig. 4g). Similar, though milder, trends were observed in female offspring (Supplementary Figs. 15–17).

To further elucidate the genetic regulation underlying IgG glycosylation-mediated immune priming effects, lung transcriptomes from PND14 male offspring, from 0.5 ppm O_3_-induced MIA as an example were analyzed. A total of 647 differentially expressed genes (DEGs) were identified (Fig. 4h). Kyoto Encyclopedia of Genes and Genomes (KEGG) and Gene Set Enrichment Analysis (GSEA) enrichment analyses indicated a transcriptional predisposition towards IL-17 signaling in male MIA offspring, with concurrent activation of the TNF signaling pathway (Fig. 4i–k). Quantitative real-time PCR (qPCR) validation confirmed the upregulation of inflammation-related genes, including C-C motif chemokine ligand (*Ccl*) *20*, C-X-C motif chemokine ligand (*Cxcl*) *1*, *Cxcl2*, *Cxcl5*, and TNF receptor-associated factor (*Traf*) *3* (Fig. 4l). We subsequently extracted 212 glycosyltransferase-encoding genes and intersected them with DEGs, revealing upregulation of glycosyltransferase genes associated with N-acetylglucosamine glycosylation, sialylation, and N-acetylgalactosamine glycosylation, such as mannoside acetylglucosaminyltransferase (*Mgat*) *3*, ST8 alpha-N-acetyl-neuraminide alpha-2,8-sialyltransferase (*St8sia*) *3*, and polypeptide N-acetylgalactosaminyltransferase (*Galnt*) *14* (Fig. 4m, n). Notably, elevated *Mgat3* expression correlated with enhanced bisecting N-acetylglucosamine modifications in the IgG glycosylation profiles. These early glycan rearrangement events likely initiated the sensitized immune phenotypes observed in offspring.

### MIA shapes glycosylation-related inflammatory signals in adult offspring

**Fig. 5:**
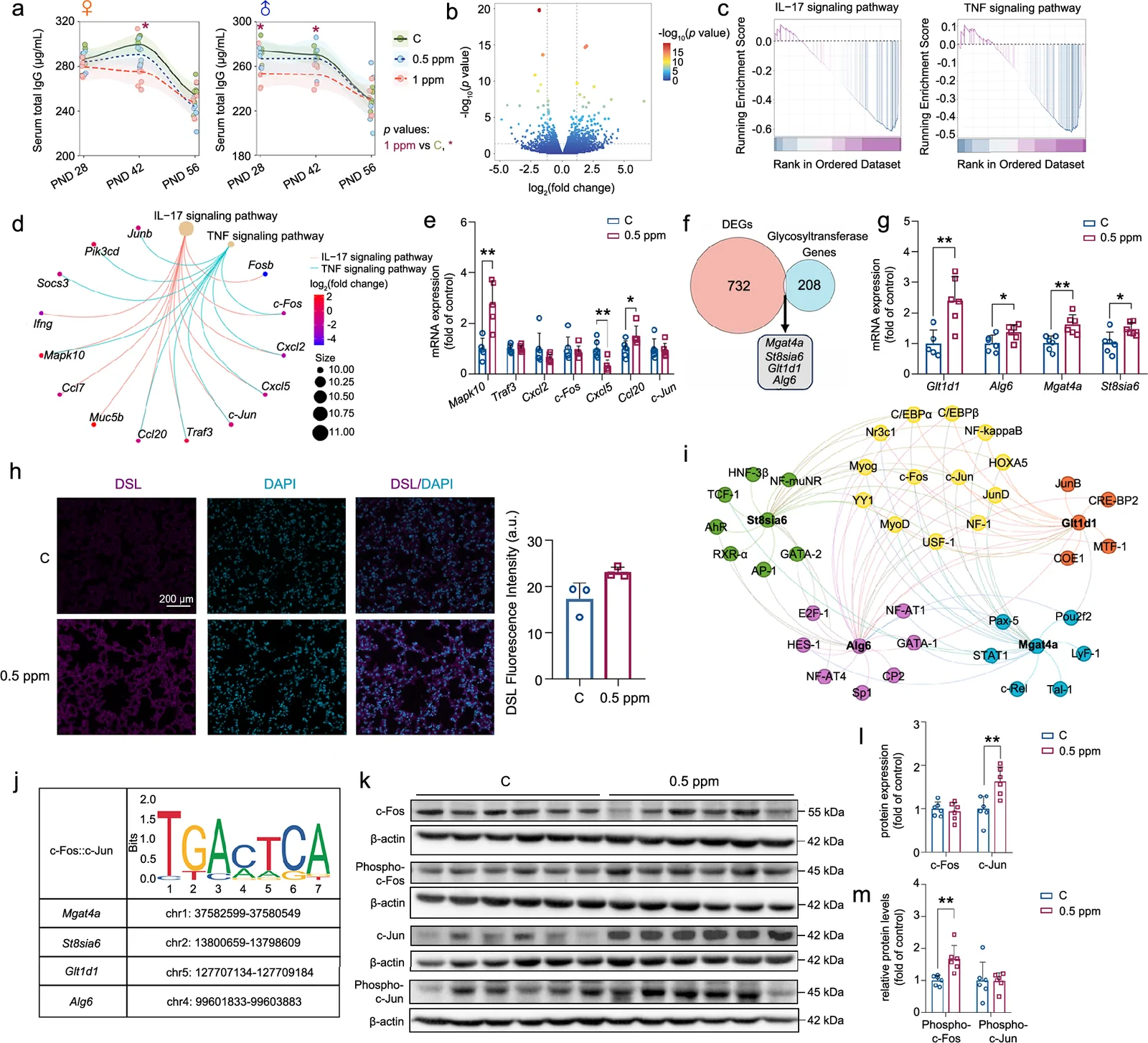
Inflammatory mediators transcriptionally regulate glycosylation-related genes in adult male offspring (PND56) following MIA. **a** Persistent serum IgG levels in female (*n* = 6 mice/group) and male offspring (*n* = 5 mice/group) at PND28, PND42, and PND56. **b** Volcano plot of lung mRNA expression in male offspring at PND56 (offspring from 0.5 ppm O_3_-induced MIA vs. controls) analyzed by two-sided exact tests (*n* = 3 mice/group). **c** GSEA of IL-17 and TNF signaling pathway. **d** DEGs overlapping IL-17 and TNF signaling pathways. **e** qPCR validation of seven DEGs involved in both IL-17 and TNF signaling pathways (for *Traf3*, *Cxcl2*, *c-Fos*, *Cxcl5*, and *c-Jun*, *n* = 6, 6; for *Ccl20*, *n* = 6, 5; for *Mapk10*, *n* = 5, 5; ordered by control and 0.5 ppm groups). **f** Intersection of 736 DEGs with 212 glycosyltransferase-encoding genes, identifying four hub genes. **g** qPCR validation of the four glycosyltransferase genes (for *Alg6*, *Mgat4a*, and *St8sia6*, *n* = 6, 6; for *Glt1d1*, *n* = 5, 6; ordered by control and 0.5 ppm groups). **h** FITC-DSL lectin staining (magenta) of lung sections from male offspring (PND56, offspring from 0.5 ppm O_3_-induced MIA vs. controls), counterstained with DAPI (cyan). (Left: Representative pseudo-colored images, scale bar, 200 μm. Right: Quantified DSL fluorescence intensity 6 random fields/sample) (*n* = 3 mice/group). **i** PROMO-predicted transcription factor (TF) network regulating hub genes. Gephi visualization highlighted 13 TFs (yellow nodes) co-regulating all 4 hub genes. **j** JASPAR prediction of c-Fos/c-Jun binding sites on four glycosyltransferase genes. Immunoblot images (**k**) and expression quantification (**l**, **m**) of c-Fos, phospho-c-Fos, c-Jun, and phospho-c-Jun protein levels in male offspring lungs (*n* = 6 mice/group). Data are mean ± SD, analyzed by one‑way ANOVA for (**a**), and by two‑tailed Student’s *t* test or Mann–Whitney U test for (**e**, **g**, **h**, **l**, **m**). *p < 0.05, **p < 0.01, and ***p < 0.001 compared with the corresponding control group.

Longitudinal tracking of IgG levels in offspring after the initiation of autonomous IgG synthesis post-PND14 revealed distinct patterns. Male MIA offspring exposed to 1 ppm O_3_ exhibited reduced IgG levels until normalization at PND56, whereas no significant differences were observed in the 0.5 ppm MIA group (Fig. 5a). Concurrently, longitudinal glycosylation profiling showed a dynamic shift in IgG glycans from PND14 to PND42 in the 0.5 ppm‑exposed males, characterized by decreased core fucosylation and increased galactosylation/sialylation, while specific pro‑inflammatory GlcNAc modifications were retained (Supplementary Fig. 18). Consequently, the lung transcriptome of adult male offspring with differentially glycosylated IgG (from 0.5 ppm O_3_-induced MIA) was analyzed to assess whether sustained alterations in IgG glycosylation exerted persistent regulatory effects on pulmonary gene expression networks. A total of 501 upregulated and 235 downregulated DEGs were identified (Fig. 5b). KEGG enrichment analysis revealed two primary inflammatory pathways, the IL-17 and TNF signaling pathways (Supplementary Fig. 19), both of which were significantly downregulated as indicated by GSEA (Fig. 5c–e). These results further support the notion that excessive immune activation persisted until the completion of maternal antibody transfer in juvenile MIA offspring, however, with the activation of the offspring’s autonomous immune regulatory mechanisms, the inflammatory response was compensatorily mitigated. Notably, this prolonged immune regulation could impact glycosylation pathways. To thoroughly analyze the transcriptomic features of glycosylation in MIA offspring, genes encoding glycosyltransferases were screened among the DEGs. Upregulation of *Mgat4a*, *St8sia6*, glycosyltransferase 1 domain containing 1 (*Glt1d1*), and ALG6 alpha-1,3-glucosyltransferase (*Alg6*) was observed in MIA offspring (Fig. 5f, g), implicating enhanced glycan sialylation and N-acetylglucosamine modifications. Consistently, DSL fluorescence showed a trend toward increased intensity for N-acetylglucosamine glycans in MIA offspring (*p* = 0.052), aligning with PND14 observations (Fig. 5h).

To further explore the transcriptional regulation of glycosyltransferase genes, transcription factors (TFs) were identified via PROMO and analyzed for correlations. A total of 13 TFs were found to regulate all four glycosyltransferase genes. Notably, the core TFs identified in the TF-gene network were inflammation-related regulators such as nuclear factor (NF) kappa B, JunD proto-oncogene (JunD, AP-1 transcription factor subunit), CCAAT/enhancer binding protein alpha (C/EBPα), and C/EBPβ (Fig. 5i), consistent with previous evidence suggesting that inflammation plays a pivotal role in glycosylation activation^35^. Among these TFs, c-Jun and c-Fos were not only identified as DEGs but also involved in the IL-17/TNF signaling pathways (Fig. 5j). The transcriptional activity of Jun proto-oncogene (c-Jun, AP-1 transcription factor subunit) and Fos proto-oncogene (c-Fos, AP-1 transcription factor subunit) is regulated by phosphorylation induced by various kinases. These results indicate that significantly elevated expression of mitogen-activated protein kinase (*Mapk*) *10* (JNK family), potentially leading to the phosphorylation of downstream substrates c-Jun and c-Fos. Consistent with expectations, western blotting confirmed that total protein levels of c-Jun were significantly upregulated in MIA offspring. Although total protein expression of c-Fos did not exhibit significant changes, its phosphorylation level was 1.67-fold higher than the control group (Fig. 5k–m). Collectively, these findings suggest that while MIA offspring exhibit restored inflammatory responses in adulthood, the regulation of IgG glycosylation by inflammatory mediators acting as TFs remains sustained.

### Altered *Mgat4a*-mediated GlcNAc glycosylation is associated with enhanced anaphylaxis susceptibility in modeling offspring

**Fig. 6:**
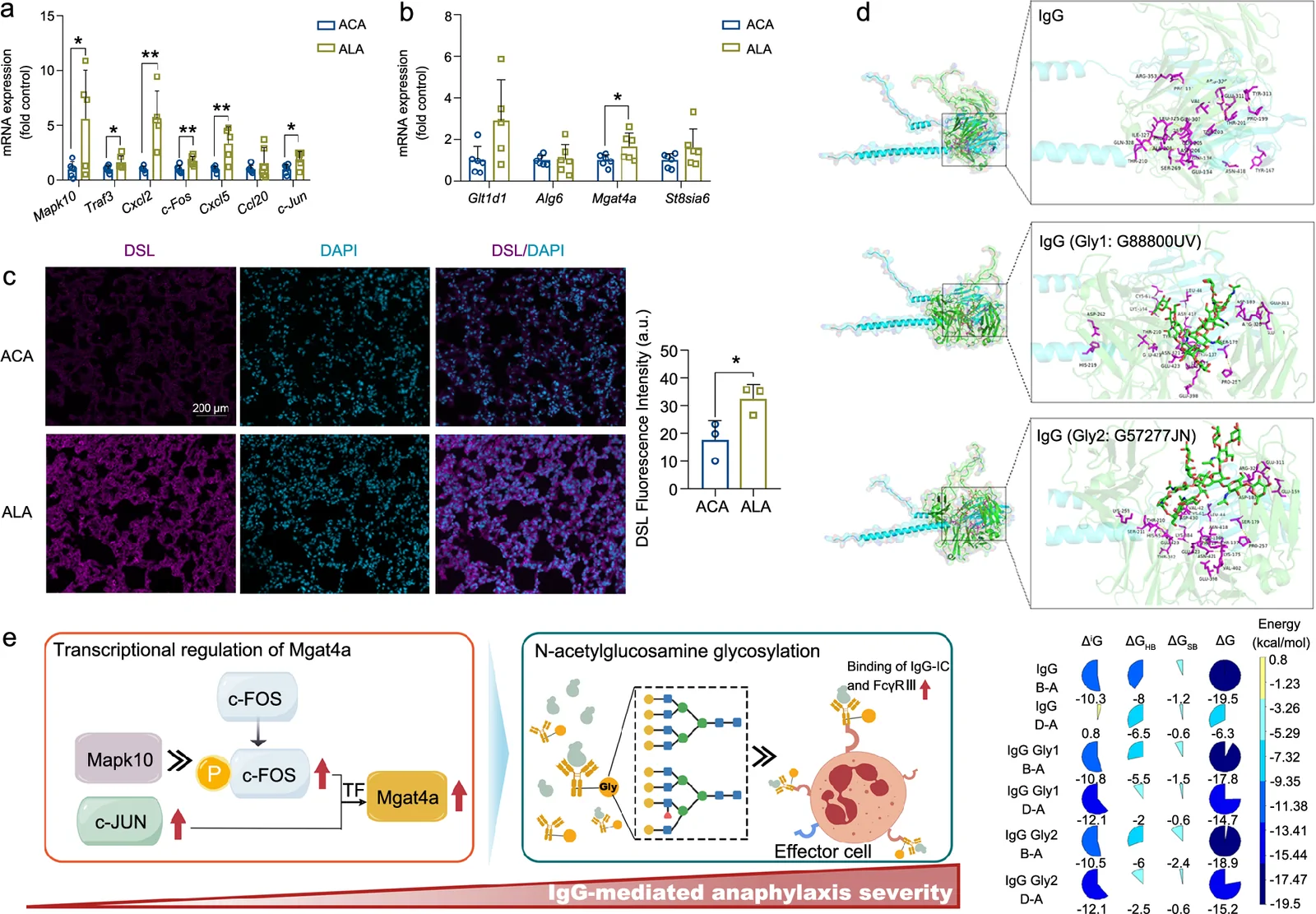
The c-Fos/c-Jun-*Mgat4a* axis may drive IgG GlcNAc glycosylation associated with IgG-mediated anaphylaxis in MIA male offspring. **a** qPCR validation of seven IL-17/TNF pathway-associated DEGs in lung of modeling male offspring (for *Traf3*, *c-Fos*, *Ccl20*, and *c-Jun*, *n* = 6, 6; for *Cxcl5* and *Mapk10*, *n* = 6, 5; for Cxcl2, *n* = 5, 5; ordered by ACA and ALA groups). **b** Expression of four hub glycosyltransferase genes in lung of modeling male offspring (for *Mgat4a*, *St8sia6*, and *Alg6*, *n* = 6, 6; for *Glt1d1*, *n* = 6, 5; ordered by ACA and ALA groups). **c** FITC-DSL lectin staining (magenta) of lung sections from modeling male offspring (ACA and ALA groups), counterstained with DAPI (cyan). (Left: Representative pseudo-colored images, scale bar, 200 μm. Right: Quantified DSL fluorescence intensity 6 random fields/sample) (*n* = 3 mice/group). **d** Molecular docking results of unmodified and GlcNAc-modified IgG with FcγRIII using GRAMM. G88800UV and G57277JN represent glycan structures corresponding to DSL in the GlycoShape database. The top-ranked complex from the docking was analyzed using PDBePISA for interface analysis, with a heatmap displaying their binding free energies. Chain A is FcγRIII, while Chains B and D are the two heavy chains of the IgG Fc domain responsible for binding to the FcγR receptor. Yellow sectors represent positive binding free energy values, while blue sectors represent negative binding free energy values. The numerical values beneath the heatmap represent the corresponding binding free energy (ΔG) values in kcal/mol. **e** A model illustrating the role of enhanced IgG N-acetylglucosamine glycosylation via c-Fos/c-Jun-*Mgat4a* signaling and its increased binding affinity with FcγRIII in increasing susceptibility to IgG-mediated anaphylaxis in MIA male offspring. The elements in this figure were created using BioGDP.com. Data are mean ± SD, analyzed by two-tailed Student’s *t* test or Mann–Whitney U test. *p < 0.05 and **p < 0.01 com*p*ared with the corresponding control group.

The role of upregulated glycosyltransferase gene expression in enhancing IgG-mediated anaphylaxis susceptibility in offspring was further investigated. qPCR analysis revealed significant upregulation of IL-17/TNF pathway genes (*Mapk10*, *Traf3*, *Cxcl2*, *c-Fos*, *Cxcl5*, and *c-Jun*) in the ALA group (Fig. 6a), with a marked increase in *Mgat4a* expression (1.65-fold higher than ACA; Fig. 6b). MGAT4A, a type II transmembrane glycosyltransferase, facilitates N-acetylglucosamine glycosylation of IgG^36^. To assess whether *Mgat4a* influences glycan modulation in ALA-susceptible male mice, DSL lectin staining revealed elevated GlcNAc levels in ALA lung tissue, with fluorescence intensity 1.84-fold higher than in the ACA group (Fig. 6c). To explore whether the heightened allergy susceptibility observed in MIA offspring within the ALA group was linked to by specific glycosylation patterns, molecular docking analysis was conducted to investigate the binding interactions between unmodified or GlcNAc-modified IgG and FcγRⅢ. GlcNAc-modified IgG exhibited lower binding free energies (ΔG = −14.7 kcal/mol for IgG Gly1 chain D with FcγRⅢ; ΔG = −15.2 kcal/mol for IgG Gly2 chain D with FcγRⅢ) than its unmodified counterpart (ΔG = −6.3 kcal/mol for IgG chain D with FcγRⅢ) when interacting with FcγRⅢ (Fig. 6d), suggesting a stronger binding affinity and more stable complex formation between GlcNAc-modified IgG and FcγRⅢ. These results suggest that the severe IgG-mediated anaphylaxis observed in male mice from the ALA group may be associated with *Mgat4a*-mediated N-acetylglucosamine glycosylation and a consequent enhancement in IgG’s ability to activate FcγRIII (Fig. 6e).

### Cross-fostering validates the dual-route transmission of allergy programming via glycosylated IgG

**Fig. 7:**
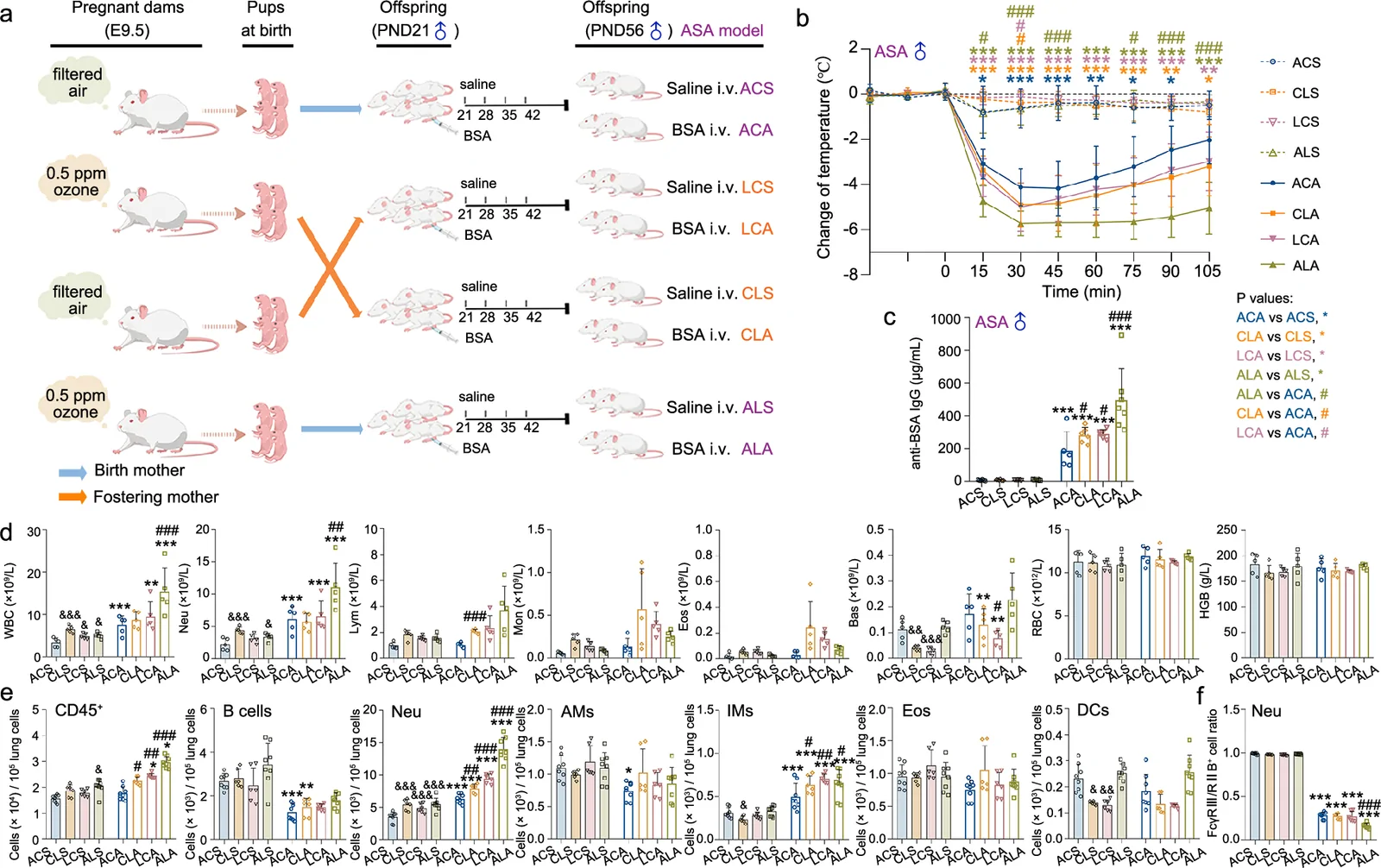
Allergic programming is transmitted via either *in utero* or lactational exposure. **a** Experimental design of cross-fostering within the 0.5 ppm O_3_ exposure paradigm and the subsequent offspring allergy models (created using BioGDP.com). **b** Body temperature changes in ASA-challenged male offspring from the cross-fostering design (*n* = 10, 8, 10, 10, 8, 8, 9, 10, ordered by ACS, CLS, LCS, ALS, ACA, CLA, LCA, and ALA groups). **c** Serum anti-BSA IgG levels in ASA-challenged male offspring from the cross-fostering design (*n* = 6, 6, 6, 6, 5, 6, 6, 7, ordered by ACS, CLS, LCS, ALS, ACA, CLA, LCA, and ALA groups). **d** CBC analysis of ASA-challenged male offspring from the cross-fostering design (*n* = 5 mice/group). **e** Inflammatory cell counts in ASA-challenged male offspring lungs from the cross-fostering design (*n* = 8, 6, 6, 8, 8, 6, 6, 8, ordered by ACS, CLS, LCS, ALS, ACA, CLA, LCA, and ALA groups). **f** FcγRIII/FcγRIIB⁺ lung neutrophil subset proportions in ASA-challenged male offspring from the cross-fostering design (*n* = 8, 6, 6, 8, 8, 6, 6, 8, ordered by ACS, CLS, LCS, ALS, ACA, CLA, LCA, and ALA groups). Data are mean ± SD, analyzed by one‑way ANOVA or Kruskal–Wallis test. *p < 0.05, **p < 0.01, ***p < 0.001 (ACA vs. ACS, CLA vs. CLS, LCA vs. LCS, ALA vs. ALS); ^#^*p* < 0.05, ^##^*p* < 0.01, ^###^*p* < 0.001 (CLA vs. ACA, LCA vs^.^ ACA, ALA vs. ACA); ^&^*p* <0.05, ^&&^*p* < 0.01, ^&&&^*p* < 0.001 (CLS vs. ACS, LCS vs. ACS, ALS vs. ACS).

Having observed that male offspring from the 0.5 ppm O_3_-exposed dams (ALA group) exhibited enhanced IgG-mediated anaphylaxis, which was associated with increased *Mgat4a*-mediated GlcNAc modification and enhanced IgG-FcγRIII interaction, we sought to determine through which physiological route(s) this glycosylation-mediated programming is transmitted. Specifically, we asked whether the effect was imprinted primarily during gestation via the yolk sac, or postnatally via breast milk. To directly address this, we performed a cross-fostering experiment within the 0.5 ppm exposure paradigm (Fig. 7a). When subjected to allergic challenge in adulthood, direct comparison of the single‑exposure groups revealed that both CLA and LCA pups developed significant and broadly comparable allergic sensitization relative to ACA groups. Specifically, both groups exhibited marked hypothermia 30 min after sensitization, along with elevated anti-BSA IgG levels and increased infiltration of immune cells (neutrophils and interstitial macrophages) in the lungs, with no significant difference between CLA and LCA groups in these core metrics (Fig. 7b–e). In contrast to the ACA group, we did not observe significant downregulation of FcγRIII/FcγRIIB expression on neutrophils in the CLA and LCA groups, whereas a marked downregulation was evident in the ALA group (Fig. 7f). Consistent with an additive effect, the ALA offspring exhibited the most severe allergic phenotype. The results demonstrate that either gestational or lactational exposure alone is sufficient to program lasting allergic susceptibility in offspring. The effect of lactational-only (LCA) exposure points to a milk-borne factor, while the comparable efficacy of in utero-only (CLA) exposure confirms prenatal transmission. These findings indicate that the glycosylated maternal IgG can act as a functional carrier relayed through both the yolk sac and breast milk pathways, serving as a key intergenerational transmitter of environmental risk across multiple developmental windows.

## Discussion

**Fig. 8:**
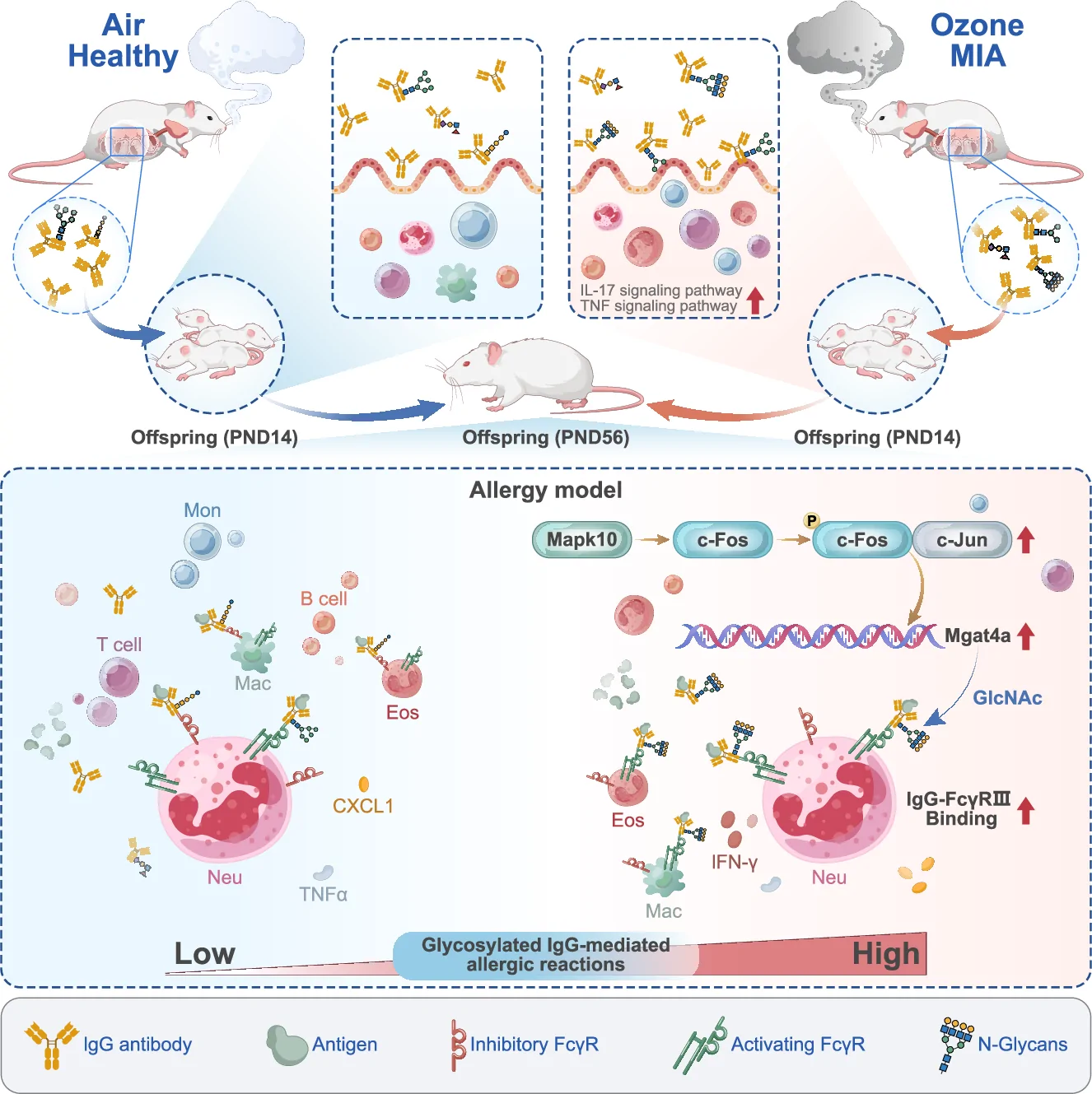
Maternal O_3_ exposure-induced MIA is associated with exacerbated IgG-mediated anaphylaxis in adult offspring, involving rearrangement of IgG glycosylation and modulation of inflammatory pathways. Maternal O_3_ exposure-induced MIA disrupts maternal-fetal IgG transfer and reprograms the IgG glycosylation profile in juvenile offspring (PND 14). This perturbation preferentially activates IL-17/TNF signaling in neonatal males. While this pathway becomes compensatorily attenuated in adult offspring (PND56) with the maturation of autonomous immune regulation, its downstream transcription factors c-Jun/c-Fos directly activated the glycosyltransferase gene *Mgat4a* to sustain GlcNAc glycosylation. This modification is associated with enhanced IgG binding affinity to FcγRIII on effector cells, which correlates with exacerbated inflammatory responses and more severe IgG-mediated anaphylaxis, particularly in male mice. The elements in this figure were created using BioGDP.com.

Extensive research underscores the pivotal role of maternal IgG in neonatal immune programming and the prevention of adult allergic diseases. Epidemiological data consistently link O_3_-induced MIA during pregnancy to an increased risk of allergy in offspring. Despite its clinical relevance, the molecular mechanisms underlying this association remain unclear. Our study identifies the mechanisms through which O_3_-induced MIA promotes IgG-mediated anaphylaxis susceptibility in offspring, emphasizing the critical roles of glycosylated IgG and inflammatory factor-mediated transcriptional regulation (Fig. 8).

First, epidemiological evidence from our cohort study indicates that exposure to O_3_ during the third trimester of pregnancy is associated with enhanced maternal inflammatory activation. This finding is consistent with previous research on PM_2.5_, suggesting that the third trimester represents a critical window during which environmental exposures influence maternal immunity. Interestingly, PM_2.5_ exposure during the early to mid-stages of pregnancy was linked to reduced levels of inflammatory cytokines^37^, a trend that parallels our observations concerning the effects of O_3_ exposure in the first trimester. These stage-specific findings highlight the dynamic nature of gestational immune programming. This changes from an immune-tolerant state in early gestation to a highly controlled, physiologically regulated state of activation in late gestation^38^. Additionally, while this study has controlled for major confounding factors through analysis, potential bias from unmeasured variables may still exist. Further measurement and analysis of relevant variables are required in future research.

Gestational homeostasis is essential for normal pregnancy, and MIA disrupts immune tolerance through cytokine release and inflammatory dysregulation. In this study, maternal O_3_ exposure triggered a significant oxidative stress response, leading to MIA. Key evidence included substantial recruitment of inflammatory cells in the lungs of model dams, particularly at 0.5 ppm O_3_. Under this exposure condition, the immune system exhibited a clear Th cell shift, which directly modulated the antibody response of B cells. It is well documented that IFN-γ secreted by Th1 cells promotes the production of IgG2a, while Th2-derived IL-4 drives the generation of IgG1^39^. Consistent with this classical regulatory paradigm, we observed a significant increase in IgG2a and a notable decrease in IgG1 levels in the 0.5 ppm exposure group at E18.5, aligning with the Th1-biased immune state in this group. Furthermore, this distinct immune environment also guided a specific remodeling of antibody glycosylation. In the 0.5 ppm group, galactosylated structures were specifically enriched in the effector subclasses IgG2a and IgG2b. In contrast, in the 1.0 ppm group, which exhibited a different immune response profile, anti-inflammatory sialylation was selectively loaded onto the IgG1 subclass.

Pregnancy is a critical period for fetal immune maturation, with maternal IgG playing an essential role in programming neonatal immunity and long-term immunomodulation^40^. For instance, maternal IgG, both natural and vaccine-induced, is transferred through the placenta, providing passive protection for ~15 weeks postnatally before infant IgG production commences^41,42^. Similar results have been observed in mouse models investigating the roles of microbe-specific and antigen-specific IgG^17,43^. Our data further suggest that MIA can alter IgG transfer. Supporting this, a UK cohort study found that SARS-CoV-2 infection during pregnancy reduced the placental transfer of protective antibodies, indicating that maternal inflammatory states can disrupt antibody delivery^44^. We further observed that the functionally biased IgG glycosylation patterns established in maternal serum persist from gestation into the lactation period, which may be facilitated by altered binding dynamics to the FcRn. In offspring at PND14, maternally derived IgG retained its specific glycosylation features and exhibited marked sexual dimorphism: total IgG levels were elevated in male offspring but decreased in females. Notably, male offspring from the 0.5 ppm O_3_-induced MIA group showed the most significant IgG glycosylation alterations, including increased N-acetylglucosamine and core fucosylation along with reduced mannosylation. This male-specific susceptibility may be partly rooted in a sexually dimorphic immune baseline, where an inherently more reactive innate immune system in males could predispose them to mount stronger responses to triggers such as maternal glycosylated IgG^45^. Subclass analysis revealed that the increase of total IgG in males is primarily driven by expansion of IgGl, on which pro-inflammatory GlcNAc modifications were predominantly enriched, whereas anti-inflammatory fucosylation was observed on IgG2a. This phenomenon finds a plausible explanation within the theoretical framework of subclass-specific IgG transport. Classical studies have confirmed that IgG transport in mice exhibits distinct subclass specificity: IgG2a and IgG2b are primarily transferred prenatally via the yolk sac pathway, whereas IgG1 is predominantly delivered postnatally through breast milk^46,47^. Building on this, our results indicate that in dams subjected to 0.5 ppm O_3_-induced MIA, galactosylated IgG2a and IgG2b antibodies are predominantly transported via the yolk sac. Conversely, IgG1, which is primarily transmitted postnatally via breast milk, exhibits enrichment of pro-inflammatory GlcNAc glycosylation. This indicates that MIA reprograms the glycosylation state of IgG1, thereby induce pro-inflammatory trend. These findings are consistent with established research: elevated N-acetylglucosamine has been linked to atherosclerosis through IgG-driven pro-inflammatory enhancement^48^. Reduced core fucosylation amplifies FcγRIII-mediated inflammation in dengue, while high-mannose IgG correlates with melanoma disease activity^49,50^. This intricate regulatory network suggests a synergistic interplay between the IgG glycosylation state and the inherent subclass-specific transport pathways, thereby constitutes a temporally precise regulatory framework, collectively enabling “selective transfer” based on immunological demands^51^.

Notably, this glycosylation signature continued to evolve during subsequent development. The first two months after birth constitute a critical window for immune system maturation, during which the glycosylation markers carried by IgG and its subclasses may significantly shape the establishment of full immune competence^15^. Studies in children with respiratory infections suggest that glycosylated IgG may serve as a key regulatory axis for humoral immune modulation during childhood^52^. The different glycosylation pattern of the IgG subclasses in the 0.5 ppm group we discussed above likely reflects an attempt by the organism to establish immune balance during early immune priming, which also helps explain the concurrent activation of both pro‑inflammatory Th17 and anti‑inflammatory Th2 cells observed in the lungs at PND14. Transcriptomic analysis revealed activation of the IL-17/TNF pathways and upregulated *Mgat3* expression, potentially priming N-acetylglucosamine regulation^53^. Our longitudinal profiling between PND28 and PND56, interpreted in light of the 21-day IgG half‑life during the transition from declining maternal antibodies to increasing endogenous IgG production^15^, revealed that as offspring’s own antibody synthesis rose, the overall glycan profile shifted toward reduced fucosylation and increased sialylation, while specific pro‑inflammatory GlcNAc modifications were retained. Lung transcriptomes from male adult offspring (0.5 ppm O_3_-induced MIA) revealed suppressed IL-17/TNF pathways. The downregulation of these pathways is intriguing from a trained immunity perspective, potentially reflecting adaptive and compensatory immune adjustments in response to early environmental stimuli. Conversely, 1 ppm O_3_-exposed males exhibited marked pro-inflammatory polarization at PND14, with enhanced Th17 differentiation and neutrophil recruitment to the lungs. Glycosyltransferases, predominantly localized in the endoplasmic reticulum (ER) and Golgi apparatus, may be compromised by excess reactive oxygen species (ROS)-induced ER stress, limiting IgG functional diversity^54^. This may explain the absence of IgG glycoform differences in male offspring from 1 ppm O_3_-induced MIA. Persistent IgG deficiencies in these offspring until PND56 could result from long-term disruptions in immune homeostasis and antibody production due to early-life hyperinflammation, rather than glycosylation-mediated regulation. These findings collectively underscore the dual role of maternally derived glycosylated IgG: providing passive immunity while programming long-term immune trajectories through fetal immune development.

Research indicates that extensive IgG glycosylation increases functional diversity^44^, which suggests that persistent changes in IgG glycosylation originated from maternal-fetal IgG transportation may be associated with increased susceptibility to diseases in adult offspring. However, current understanding remains limited, particularly those associated with anaphylaxis characterized by hypothermia and reduced consciousness^25^. In the present study, male offspring from 0.5 ppm O_3_-induced MIA exhibited pronounced hypothermia, elevated anti-BSA IgG, increased IL-6/CXCL1, and decreased IL-5, validating that neonatal IgG glycosylation alterations and inflammatory dysregulation induced by maternal oxidative stress during MIA drive abnormalities in MIA offspring. This finding aligns with recent experimental evidence. This pathway holds significant relevance in clinical scenarios involving substantial systemic antigen exposure, such as reactions to drug or therapeutic antibody infusions^27^. Beyond these high-dose scenarios, growing experimental evidence supports that IgG-mediated systemic anaphylaxis can occur at antigen doses lower than conventionally recognized thresholds (e.g., as low as 0.008 mg antigen and 0.04 mg antibody)^55^. The key mechanism involves IgG-antigen complexes engaging Fcγ receptors (e.g., FcγRIII), which activates effector cells including neutrophils and macrophages and promotes the release of inflammatory mediators such as platelet-activating factor (PAF)^56^. Such IgG immune complex-triggered responses have been demonstrated in populations with specific food allergies, such as to lipid transfer protein (LTP)-rich foods, and are also pathophysiologically relevant to a range of conditions including acute lung injury (ALI), acute respiratory distress syndrome (ARDS), and rheumatoid arthritis^57–59^. We have demonstrated that male offspring from O_3_-exposed dams (the ALA group) exhibit a significant increase in neutrophils—key responder cells in anaphylaxis—accompanied by markedly altered FcγRIII expression levels on these cells, findings that directly corroborate this antibody-myeloid receptor interaction mechanism. For females, these differences are less pronounced than in males. In addition, the structural features of the pulmonary microvasculature provide a foundation for the localized aggregation of IgG immune complexes^28^. Consistent with the inflammatory phenotype, MIA male offspring exhibited pulmonary pathological damage such as increased mean alveolar airspace size, indicating impaired gas exchange^60^. These findings suggest that differentially glycosylated IgG transferred from maternal to the male or female offspring contribute to different anaphylaxis later in life, particularly upon allergen exposure.

Inflammatory responses drive the dysregulated expression of glycosyltransferases through transcriptional control by pro-inflammatory cytokines, emerging as a critical factor in altered IgG glycosylation under pathological conditions^61^. Consistent with our findings, mouse studies indicate that offspring of infected dams gain enhanced immunity via epithelial modulation but face an increased risk of colitis^62^. In MIA offspring, allergen exposure co-activates IL-17/TNF pathways (*Mapk10*, *Traf3*, *Cxcl2*, *Cxcl5*, *c-Fos*, and *c-Jun*), upregulating glycosyltransferase genes (*Mgat4a*) through c-Fos/c-Jun-mediated transcription, highlighting the role of pro-inflammatory responses in orchestrating IgG glycosylation remodeling. c-Fos typically forms heterodimers with Jun proteins (c-Jun/JunB/JunD), creating the Activator Protein 1 (AP-1) transcription factor complex, regulated by MAPK signaling. Within this pathway, the MAPK cascade can phosphorylate both c-Fos and c-Jun, enhancing their transcriptional activity^63^. Our data indicate that *Mapk10* upregulation enhances c-Fos phosphorylation, activating downstream regulatory pathways. Numerous studies have demonstrated the significant role of inflammatory factors in regulating glycosyltransferase genes. TNF has been shown to downregulate ST6 beta-galactoside alpha-2,6-sialyltransferase (ST6Gal) 1, and its regulated desialylation modification exacerbates fibroblast inflammation^64^. IL-6/IL-8 upregulates fucosyltransferase (FUT) 11/ST3GAL6, increasing mucin fucosylation/sialylation in patients with cystic fibrosis^65^. *Mgat4a*, part of the N-acetylglucosaminyltransferase family, encodes enzymes that transfer GlcNAc to core mannose residues. O_3_ (0.5 ppm)-primed MIA enhanced ASA-triggered *Mgat4a* upregulation in males, alongside amplified DSL lectin signals indicative of GlcNAc rearrangement. Recent studies have highlighted that MGAT4A-mediated GLUT1 glycosylation drives endometrial cancer progression^36^. Notably, FcγRⅢ plays a primary role as an effector in the activation of antibody-dependent cellular cytotoxicity (ADCC) by effector cells^66^. Evidence from prior studies using surface plasmon resonance has confirmed that GlcNAc-modified IgG binds more strongly to the activating receptor FcγRⅢ^67^. These findings are further corroborated by studies utilizing glycoengineered antibodies and in vitro glycan remodeling platforms, with matrix-assisted laser desorption/ionization (MALDI)-mass spectrometer (MS) analysis confirming that the enhanced effector function directly correlates with bisecting GlcNAc addition^68,69^. These collective observations provide strong support for our finding that *Mgat4a*-regulated GlcNAc glycosylated IgG exhibited a stronger binding affinity to FcγRⅢ compared to unmodified IgG, an enhancement that correlates with increased IgG-mediated anaphylaxis susceptibility in male adult offspring (from 0.5 ppm O_3_-induced MIA). Interestingly, this GlcNAc pattern matched neonatal IgG upon maternal transfer completion (PND14), emphasizing a critical feedback loop for postnatal immune homeostasis. However, species-specific differences in IgG glycosylation and the current lack of methods to generate mouse IgG with the specific bisecting GlcNAc modification limit our ability to directly validate the contribution of GlcNAc-modified IgG–FcγRⅢ binding to allergic susceptibility in mouse models. Therefore, further research to develop species-specific glycoengineering tools is essential.

While the primary focus of this study is on the role of IgG glycosylation modifications in O_3_ exposure‑affected maternal-fetal immune transfer, we have not excluded other plausible parallel mechanisms. For instance, previous studies have indicated that O_3_ can interact with heme iron and disrupt iron homeostasis, thereby potentially affecting HGB levels. In our cohort study, we did observe a significant association between O_3_ exposure in the third trimester and an increased risk of anemia, although adjusting for HGB as a covariate did not materially alter our primary findings. From a physiological perspective, the substantially increased iron demand during late pregnancy may render maternal iron metabolism more susceptible to O_3_-induced oxidative stress and inflammation^70^. This also suggests that gestational O_3_ exposure may directly affect maternal health through disruption of iron metabolism, and future studies could further explore the underlying mechanisms of this pathway and its potential interactions with immune effects. Other maternal factors—including alterations in gut microbiota composition (which can vertically shape the offspring microbiome) and intrauterine epigenetic modifications—may independently or synergistically influence offspring immunity^71^. In addition, in contrast to IgG, the transfer efficiency of maternal IgE to offspring via the placenta or breast milk is remarkably low, with its limited transport believed to depend primarily on the formation of complexes with IgG rather than on direct transfer—further underscoring the dominant role of maternal IgG in neonatal immune programming^72^. Whether glycosylation of maternal IgE might also fine‑tune its interaction with IgG and thereby influence the formation and transport of IgE–IgG immune complexes represents an interesting direction for future research into the potential functions of antibodies at very low concentrations. Overall, the present study demonstrated that MIA-induced maternal IgG glycosylation rearrangement provides a complementary perspective on the mechanisms that may operate during critical windows of developmental immune transfer following prenatal exposure.

Our findings suggest that the enhanced IgG-mediated anaphylaxis observed in offspring following maternal O_3_ exposure may be linked to MIA-induced alterations in neonatal IgG glycosylation. These alterations, through modulation of inflammatory pathways and *Mgat4a*-mediated GlcNAc glycosylation, shape immune remodeling in the offspring, accompanied by enhanced IgG binding to FcγRIII on effector cells. This reveals a potential pathway for investigating oxidative stress-induced MIA-related allergic responses in humans, emphasizing the importance of early-life immune programming. A notable implication is that mitigating gestational oxidative stress and monitoring neonatal IgG glycosylation profiles could serve as controllable strategies for preventing allergic diseases. However, several limitations require further investigation: studies employing IgG-deficient or FcRn-deficient dams would be valuable to establish definitive causal evidence for the role of glycosylated maternal IgG in this intergenerational transmission; due to current technical limitations in isolating specific IgG glycoforms, studies generating GlcNAc-modified mouse IgG are needed to directly test its contribution to IgG-mediated anaphylaxis susceptibility in vivo; and population-based studies are needed to validate the allergic relevance of MIA-driven offspring IgG glycosylation rearrangement observed in mouse models.

## Methods

### Participants in the retrospective study

This retrospective cohort study analyzed birth records of 10,188 mother-infant pairs collected from Bethune Hospital (Taiyuan, Shanxi Province) between January 1, 2018, and December 31, 2020. Initial exclusions comprised non-permanent residents, non-singleton births, pregnancies with incomplete information, non-live births, pregnancies with gestational age <28 or > 42 weeks, and maternal ages <20 years. Subsequent exclusions eliminated infants with congenital malformations, births with implausible anthropometric data, and mothers diagnosed with gestational comorbidities or pre-existing chronic conditions. The final analytic cohort included 5299 eligible pregnancies. A detailed participant selection flowchart is provided in Supplementary Fig. 1. Demographic and socioeconomic characteristics derived from birth records are summarized in Supplementary Table 1. The study protocol received ethical approval from the Ethics Committee of Shanxi Bethune Hospital (Approval No. YXLL-KY-2021-016), with exemption from informed consent.

### Exposure assessment in the retrospective study

Participant residential addresses obtained from birth records were converted into latitude and longitude geographic coordinates and spatially matched with the China Air Quality ReAnalysis dataset (CAQRA) to retrieve daily ambient pollutant exposure concentrations (PM_2.5_, PM_10_, NO_2_, O_3_ [calculated as the daily maximum 8-h average O_3_ concentration], and SO_2_) for all enrolled mothers from 12 weeks preconception to delivery. Building upon conventional approaches, exposure windows were stratified as: preconception (12 weeks before pregnancy), first trimester (weeks 1–12), second trimester (weeks 13–27), third trimester (weeks 28 to delivery), mid-to-late pregnancy (weeks 13 to delivery), and the entire pregnancy period. Exposure levels for corresponding gestational periods were derived by averaging daily exposure concentrations.

### Statistical analysis in the retrospective study

Hematological reference ranges followed the Chinese Health Industry Standard (WS/T 405-2012) for blood cell analysis^73^. Hematological abnormalities were defined as values exceeding or falling below clinical reference ranges for white blood cells (WBC: 3.5–9.5 × 10^9^/L), neutrophils (Neu: 1.8–6.3 × 10^9^/L), lymphocytes (Lym: 1.1–3.2 × 10^9^/L), monocytes (Mon: 0.1–0.6 × 10^9^/L), eosinophils (Eos: 0.02–0.52 × 10^9^/L), basophils (Bas: 0–0.06 × 10^9^/L), and fibrinogen (FIB: 2.38–4.98 g/L). HGB abnormality was defined as levels below 110 g/L. Based on this criterion, women were stratified into normal and abnormal HGB groups, and descriptive statistics (mean ± SD) were calculated for each group. These dichotomous outcomes were determined using standardized laboratory protocols. Gestational age (weeks) was calculated from the last menstrual period and confirmed by ultrasound. Descriptive statistics summarized demographic characteristics. Associations between categorical variables (considered biologically relevant to hematological parameters or potential confounding effects) and hematological abnormalities were analyzed using χ² tests (or Fisher’s exact test for small-frequency cases). Logistic regression models estimated adjusted odds ratios (ORs) and 95% confidence intervals (CIs) for hematological abnormalities associated with each 10 μg/m^3^ increase in trimester-specific air pollutants exposure, adjusting for maternal age (<28, 28–35, >35 years), pre-pregnancy BMI (<18.5, 18.5–24, >24 kg/m^2^), education (college degree: yes/no), occupation (employed or unemployed), monthly family income per person (≤4000, >4000 yuan), smoking (yes/no), drinking (yes/no), folic acid supplementation (yes/no), gravidity (≤1, 2, ≥3), parity (≤1, 2, ≥3), and previous adverse pregnancy (yes/no). To assess the robustness of our findings, we further performed multi-pollutant regression analyses adjusting for PM_2.5_, PM_10_, SO_2_, and NO_2_, as well as models adjusting for HGB levels. Analyses were performed using R (v: 4.4.2), with statistical significance set at *p* < 0.05.

### Animal protocol

All animal procedures were approved by the Committee of Scientific Research at Shanxi University (CSRSX; Approval No. SXULL2021018) and conducted in accordance with institutional guidelines. BALB/c mice (7- to 8-week-old, Beijing Vital River Laboratory Animal Technology Co., Ltd.) were acclimatized for one week under controlled conditions (12-h light/dark cycle, 22 ± 2 °C, 50% relative humidity) with ad libitum access to food and water. For timed pregnancy, male and female mice were co-housed at a 1:1 ratio, and vaginal plug detection was used to designate embryonic day 0.5 (E0.5). Pregnant mice were randomly assigned to control or O_3_-exposed groups (0.5 or 1 ppm O_3_, 4 h/day). O_3_ exposure began at E9.5, a critical window for fetal immune system development, with O_3_ generated via ultraviolet light irradiation and precisely controlled by mass flow controllers, continuously monitored using calibrated O_3_ analyzers (TONGLIN OZONE). Exposure concentrations were chosen based on the following criteria: (1) the U.S. National Ambient Air Quality Standard (NAAQS) 8-h average limit (0.07 ppm) and China’s Grade I/II 1-h standards (0.075 ppm/0.093 ppm); (2) epidemiological observations of peak O_3_ levels (0.2–0.3 ppm) in heavily polluted regions; and (3) interspecies dose extrapolation (4–5 times higher mouse pulmonary deposition vs. humans, corresponding to human-equivalent exposures of 0.1 and 0.2 ppm, respectively, as validated in prior toxicokinetic research)^74–76^. Maternal weight, food intake, and clinical status were monitored daily. Dams were individually housed until weaning of the offspring at postnatal day 21 (PND21). Post-weaning, pups were group-housed by sex for subsequent experimental procedures. For the cross-fostering procedure, pups were reciprocally exchanged between nursing dams within 12 h of birth. As described in previous studies^13^, mice were humanely euthanized by cervical dislocation under deep isoflurane anesthesia (using an ABS small animal anesthesia system from Shanghai Yuyan Instruments Co., Ltd.), and neonates were sacrificed by decapitation. Death was confirmed by cessation of cardiac and respiratory activity prior to any subsequent analyses.

### Tissue collection and phenotypic assessment

At E18.5, pregnant dams were humanely euthanized for multilevel analyses. Blood was collected for complete blood count (CBC) profiling, ELISA quantification, and lectin microarray assays. Lung and spleen tissues were harvested for superoxide dismutase (SOD) activity measurement, histopathological evaluation, and flow cytometry analysis. All samples were processed and analyzed individually (no pooling across animals) to maintain biological independence. Uterine horns were excised and dissected to harvest embryos and yolk sacs, with fetal counts recorded. Individual fetuses were transferred to ice-cold saline for gross morphological characterization. In addition, body weight, sex identification by gonadal morphology, crown-rump length (CRL), and placental morphometrics (weight, diameter, and thickness) were recorded. The remaining pregnant dams were allowed to deliver naturally, with parturition dates and neonatal viability tracked. At PND14, 28, 42, and 56, blood samples from male and female pups were collected for ELISA. A subset of offspring was anesthetized at PND14 and PND56 for pulmonary function testing, followed by necropsy, during which lung and spleen tissues were collected for histopathological, molecular, and immunological analyses. Additionally, PND56 mice were used to establish a systemic IgG-Mediated Anaphylaxis model. To minimize litter effects, offspring samples were systematically randomized across biological replicates from distinct litters.

### Systemic IgG-mediated anaphylaxis modeling

Based on well‑characterized protocols^28^, active and passive systemic anaphylaxis models were established in offspring from dams exposed to O_3_ or filtered air during gestation. For the active systemic anaphylaxis (ASA) models, male and female offspring received weekly intraperitoneal injections of 200 μg bovine serum albumin (BSA, purity ≥96%, Solarbio, Beijing, China, dissolved in saline) starting at PND21. Fourteen days post-final immunization (at 8 weeks of age), ASA was induced by intravenous administration of 500 μg BSA via the tail vein. Experimental cohorts were designated as ACA (offspring from air-exposed dams + BSA), ALA (offspring from 0.5 ppm O_3_-exposed dams + BSA), and AHA (offspring from 1 ppm O_3_-exposed dams + BSA), with saline-treated controls (ACS: offspring from air-exposed dams + saline; ALS: offspring from 0.5 ppm O_3_ + saline; AHS: offspring from 1 ppm O_3_ + saline). To quantitatively evaluate the severity of systemic anaphylaxis, core body temperature was continuously monitored at 15-min intervals for 4 h post-antigen challenge using a digital thermometer. During the establishment of the ASA model, in addition to following the standard protocol, we also implemented a cross-fostering experimental design to investigate the respective roles of different exposure windows. Newborn pups were reciprocally exchanged between control dams and dams subjected to 0.5 ppm O_3_ exposure within 12 h after birth. All male offspring from these cross‑fostered groups were then evaluated, starting from PND21, using the identical ASA sensitization and challenge protocol described above, resulting in the following four experimental groups: ACS/ACA (control pups nursed by control dams, challenged with saline or BSA, respectively); ALS/ALA (exposed pups nursed by 0.5 ppm O_3_‑exposed dams, challenged with saline or BSA, respectively); CLS/CLA (exposed pups nursed by control dams, challenged with saline or BSA, respectively); and LCS/LCA (control pups nursed by 0.5 ppm O₃‑exposed dams, challenged with saline or BSA, respectively). For the passive systemic anaphylaxis (PSA) models, IgG immune complexes (IgG-ICs) were generated by pre-incubating 90 μg OVA-specific IgG1 (Clone F2-3.58, purity >95%, BioXcell, West Lebanon, NH, USA) with 30 μg ovalbumin (OVA) (Sigma-Aldrich, purity ≥98%, St. Louis, MO, USA) at 37 °C for 30 min. At 8 weeks of age, offspring were intravenously injected with preformed IgG-ICs. Cohorts were labeled PCA (offspring from air-exposed dams + IgG-ICs), PLA (offspring from 0.5 ppm O_3_ + IgG-ICs), PHA (offspring from 1 ppm O_3_ + IgG-ICs), and saline-treated controls (PCS: offspring from air-exposed dams + saline; PLS: offspring from 0.5 ppm O_3_ + saline; PHS: offspring from 1 ppm O_3_ + saline). Thermometric monitoring followed identical protocols. Any animal exhibiting loss of righting reflex, severe respiratory distress, or signs of circulatory failure was euthanized immediately to avoid unnecessary suffering. After challenge, mice underwent pulmonary function assessments followed by necropsy. Lung tissue samples were collected for comprehensive histopathological evaluation and molecular characterization, while serum was simultaneously collected for immunological profiling.

### Histopathological evaluation

Left lungs were inflation-fixed with 4% paraformaldehyde for 24 h, paraffin-embedded, and sectioned at 5–6 μm thickness for hematoxylin and eosin (H&E) staining. Morphometric analyses were performed on three randomly selected sections per biological replicate. The mean linear intercept (L_m_), a stereological index of alveolar airspace enlargement, was quantified using a grid-based method: parallel test lines of defined length (d) were systematically overlaid on ten non-overlapping fields per section. The number of intercepts intersecting alveolar surface [I(A)] and endpoints contacting airspaces [P(asp)] were counted, with L_m_ calculated as L_m_ = 2·k·d·P(asp)/I(A) (k = 1/2)^77^. Bronchiolar remodeling was assessed by measuring the total cross-sectional area (μm^2^), luminal area (μm^2^), and internal perimeter (μm) of airways. Medial area was derived by subtracting luminal area from the total area, and medial thickness was calculated as medial area/internal perimeter^78^.

### Quantification of antibodies in serum and milk

For serum collection, whole blood was allowed to clot at room temperature for 3–4 h, followed by centrifugation at 900 × *g* for 15 min to isolate serum, which was stored at −80 °C until analysis. For milk collection, as previously described^18^, dams were separated from their pups for 3 h on PND14. To induce milk letdown, each dam was injected intraperitoneally with 250 μL of oxytocin (2 IU diluted in 1 mL PBS; Shanghai Yuanye Bio-Technology Co., Ltd., China). After 5 min, dams were anesthetized with isoflurane and gently massaged for milk collection. The milk was diluted 1:3 in PBS and centrifuged at 21,000 × *g* for 15 min. The fat layer was removed, and the supernatant was transferred to a new tube for a second centrifugation under the same conditions. The final, defatted milk supernatant was stored at −80 °C.

Total IgG levels and total IgE levels in serum and milk were quantified using a commercial enzyme-linked immunosorbent assay (ELISA) kit (Cat. No. LV30268 for IgG; Cat. No. LV30255 for IgE; Animalunion Biotechnology Co., Ltd, Shanghai, China). Concentrations of IgG subclasses (IgG1, IgG2a, IgG2b, and IgG3) were determined using corresponding mouse IgG subclass ELISA kits (Cat. No. EM1152 for IgG1; Cat. No. EM1153 for IgG2a; Cat. No. EM2086 for IgG2b; Cat. No. EM1154 for IgG3; Fine Biotech Co., Ltd., Wuhan, China). The total protein concentration in milk was determined using the Bradford assay (Solarbio, Beijing, China) with BSA standards, and the antibody concentrations were subsequently normalized to this value and are presented as the amount of antibody per milligram of total protein.

For allergen-specific IgG and IgE profiling, serum from model mice was analyzed using anti-OVA IgG (Cat. No. LV31260, Animalunion Biotechnology Co., Ltd., Shanghai, China), anti-BSA IgG (Cat. No. EM2527, Fine Biotech Co., Ltd., Wuhan, China), and anti-BSA IgE (Cat. No. EM2289, Fine Biotech Co., Ltd., Wuhan, China) ELISA kits, respectively. All assays were conducted in strict accordance with the respective manufacturers’ protocols.

### Cytokine quantification by Luminex

Serum cytokine levels (IFN-γ, IL-1β, IL-2, IL-4, IL-5, IL-6, CXCL1, IL-10, IL-12p70, and TNF-α) in PSA and ASA model mice were quantified using a Luminex-based multiplex assay (Proinflammatory Panel 1, BIO-RAD). Detailed information could be found in Supplementary Text 1.

### Flow cytometry analysis

Mouse lung tissues were dissociated in HBSS (Hanks’ Balanced Salt Solution, Solarbio, Beijing, China) containing 0.5 mg/mL collagenase type IA (Sigma-Aldrich, St. Louis, MO, USA) and 20 μg/mL DNase I (Sigma-Aldrich, St. Louis, MO, USA) by mincing, followed by incubation at 37 °C for 30 min. Splenic tissues were mechanically dissociated by grinding. Single-cell suspensions were generated by filtering through a 70 μm cell strainer, subjected to red blood cell lysis (Solarbio, Beijing, China), and resuspended in PBS supplemented with 2% FBS (Dcell, Shanghai, China). Cell viability (>95% live cells) was confirmed via trypan blue exclusion assay, and aliquots were prepared for staining. For innate immune profiling, cells were stained with the following surface antibody panel: CD45-eFluor506 (eBioscience; 0.5 μg/10^6^ cells), Ly6C-eFluor450 (eBioscience; 0.25 μg/10^6^ cells), Ly6G-FITC (eBioscience; 0.5 μg/10^6^ cells), I-A/I-E-PE (eBioscience; 0.02 μg/10^6^ cells), CD11b-PE-Cyanine7 (eBioscience; 0.125 μg/10^6^ cells), Siglec F-eFluor660 (eBioscience; 0.125 μg/10^6^ cells), F4/80-Alexa Fluor700 (eBioscience; 0.25 μg/10^6^ cells), CD11c-APC-eFluor780 (eBioscience; 0.5 μg/10^6^ cells), and CD16/32-PerCP-Cyanine5.5 (BioLegend; 0.25 μg/10^6^ cells). For T cell subset analysis, single-cell suspensions were stimulated with Cell Stimulation Cocktail (PMA/ionomycin/brefeldin A/monensin, 500X, eBioscience) in complete RPMI 1640 medium (Servicebio Technology Co., Ltd., Wuhan, China) for 5 h at 37 °C under 5% CO_2_. Cells were blocked with Fc Receptor Blocker (eBioscience; 1:100 dilution) for 15 min, stained with LIVE/DEAD Fixable Viability Dye-eFluor450 (eBioscience; 1:1000 dilution) on ice for 30 min, and surface-labeled with: CD3-FITC (eBioscience; 0.25 μg/10^6^ cells), CD4-eFluor506 (eBioscience; 0.5 μg/10^6^ cells), CD25-eFluor660 (eBioscience; 0.125 μg/10^6^ cells). Intracellular staining was performed using the Foxp3/Transcription Factor Staining Buffer Set (eBioscience) with IFN-γ-PerCP-Cyanine5.5 (eBioscience; 0.25 μg/10^6^ cells), IL-4-PE-Cyanine7 (eBioscience; 0.25 μg/10^6^ cells), IL-17A-Brilliant Violet711 (eBioscience; 0.25 μg/10^6^ cells), Foxp3-PE (eBioscience; 1 μg/10^6^ cells). Data were acquired on an Attune NxT Acoustic Focusing Cytometer (Thermo Fisher Scientific, USA) and analyzed using FlowJo (v: 10.8.1) with gating strategies detailed in Supplementary Fig. 20. Fluorescence-minus-one (FMO) controls were shown in Supplementary Fig. 21. Dimensionality reduction (t-SNE) and viSNE analysis (Cytobank) were employed to resolve immune subsets. Antibody details are summarized in Supplementary Table 11.

### Pulmonary function assessment

Invasive pulmonary function analysis was performed on PSA and ASA model mice as previously described, using the AniRes2005 system (v: 3.5, Beijing Bestlab Technology Co., Ltd.)^79^. To mitigate invasive procedures in immature lungs, unrestrained whole-body plethysmography (WBP) was conducted on neonatal pups at PND14 using the Tow-Int Tech system (Tower International Technology, Shanghai, China). Detailed parameters are provided in Supplementary Text 1.

### Superoxide dismutase (SOD) activity assay

Lung tissues from dams at E18.5 were processed for SOD activity quantification following manufacturer protocols (Nanjing Jiancheng Bioengineering Institute, Nanjing, China). Detailed information could be found in Supplementary Text 1.

### Measurement of inflammatory cytokines in lung tissue and serum

Approximately 30 mg of lung tissue was homogenized on ice in pre‑cooled phosphate‑buffered saline (PBS, 0.1 M, pH 7.4). The homogenate was centrifuged at 1200 × *g* for 10 min at 4 °C, and the supernatant was collected. The concentrations of inflammatory cytokines (IFN‑γ, TNF‑α, IL‑1β, IL‑4, and IL‑13) in lung and serum were measured using corresponding mouse ELISA kits (Cat. No. LV30253 for IFN‑γ; Cat. No. LV30536 for TNF‑α; Cat. No. LV30300 for IL‑1β; Cat. No. LV30323 for IL‑4; Cat. No. LV30278 for IL‑13; Animalunion Biotechnology Co., Ltd, Shanghai, China) according to the manufacturer’s instructions. Total protein concentration in the lung supernatant was determined via Bradford assay (Solarbio, Beijing, China) using BSA standards. Cytokine levels in lung tissue were normalized to total protein content.

### Reactive oxygen species (ROS) probe detection

At E18.5, pregnant dams were anesthetized with intraperitoneal sodium pentobarbital (50 mg/kg) and administered ROS-specific probes via bilateral intranasal instillation (15 μL per nostril, totaling 30 μL). Probe solutions were prepared as follows: H_2_O_2_-sensitive probe in dimethyl sulfoxide (DMSO) and ClO^-^-sensitive probe in dimethylformamide (DMF), both at a final concentration of 300 μM. Thirty minutes post-administration, major organs (heart, liver, spleen, lung, kidney) and embryonic tissues were rapidly dissected, rinsed in ice-cold PBS (pH 7.4), and subjected to ex vivo imaging. Multispectral fluorescence imaging was conducted using a Maestro EX system (PerkinElmer, Inc.) with 523 nm excitation and a 560 nm longpass emission filter, ensuring tissue processing within 10 min to maintain oxidative stress marker integrity. Fluorescence signals were quantified using Maestro (v: 3.0) software. Subsequently, lung and placental tissues were cryosectioned at 10 μm thickness (Leica CM1950 Cryostat, Leica Microsystems) and imaged with a fluorescence microscope (IX73, Olympus, Japan).

### Hematological and biochemical profiling

Whole blood was collected from isoflurane-anesthetized mice via retro-orbital plexus puncture using sterile glass capillary tubes (200 μL per animal) and immediately transferred to pre-chilled EDTA-coated Microtainer tubes, followed by 8–10 gentle inversions to ensure anticoagulant mixing. CBCs were performed using a Mindray BC-5000vet veterinary automated hematology analyzer (Servicebio Technology Co., Ltd., Wuhan, China), which quantified leukocyte differentials (WBC, neutrophils, lymphocytes, monocytes, eosinophils, and basophils) as well as red blood cell (RBC) count and hemoglobin (HGB) concentration.

### Western blot analysis

Western blot analysis of lung tissues (40 mg) was performed to quantify protein levels of c-Fos, c-Jun, and their phosphorylated forms (phospho-c-Fos, phospho-c-Jun), with β-actin as the loading control. Detailed experimental protocols are provided in Supplementary Text 1.

### RNA isolation and quantitative real-time PCR

Total RNA was isolated from lung, yolk sacs, and intestinal tissues using TRIzol Reagent (TaKaRa Bio. Inc., Shiga, Japan) following the manufacturer’s protocol. Total RNA was reverse-transcribed into cDNA using the PrimeScript RT Reagent Kit (Cat. No. RR047A, TaKaRa Bio. Inc.). qPCR reactions were performed in 20 μL volumes containing 10 μL SYBR Green (Mei5 Biotechnology, Beijing, China), 0.5 μM forward/reverse primers (sequences in Supplementary Table 12), 2 μL cDNA template, and nuclease-free water. Amplification was performed on a 7500 Real-Time PCR System (Thermo Fisher Scientific, USA). Relative gene expression was calculated using the ΔΔCt method, normalized to glyceraldehyde-3-phosphate dehydrogenase (*Gapdh*) as the endogenous control. Data were expressed as fold changes relative to matched control.

### Lectin microarray profiling of IgG glycosylation

Serum IgG (both total IgG and its subclasses IgG1, IgG2a, IgG2b, and IgG3) glycosylation profiling was conducted by Guangzhou BCBIO Biotechnology Co., Ltd. using custom lectin microarrays. Pre-coated chips were equilibrated at 4 °C for 30 min, followed by a 3-hour blocking step at 4 °C. After three washes with PBST (0.05% Tween-20) and air-drying, 200 μL of diluted serum IgG (1:10 in incubation buffer) was applied to each array in containment frames and incubated overnight at 4 °C. After hybridization, unbound IgG was removed by sequential PBST washes, followed by a 1-h incubation with Cy3-conjugated goat anti-mouse IgG cross-adsorbed secondary antibody under light-protected conditions at room temperature. Fluorescent signals were acquired using a LuxScan 10K-A microarray scanner (CapitalBio Corporation, China) at 5 μm resolution, with the photomultiplier tube (PMT) gain set to 700 and laser power set to 75%. Each group consisted of three biological replicate samples. For each biological sample, lectin binding signals were measured in triplicate on the microarray. The lectin microarray data were processed using GenePix Pro 6.0 to extract the foreground signal intensity (F532, representing the median signal foreground value at 532 nm channel) and background signal intensity (B532, the median signal background value at 532 nm channel) for all lectin microarray spots. For the extracted data, background correction was performed to eliminate signal non-uniformity caused by inconsistent background values among different lectin spots by calculating the foreground-to-background difference (F–B) for each lectin spot. To eliminate errors from nonspecific lectin signals, the F–B values of blank controls (control) were subtracted from the F–B values of each lectin spot, and the SND was defined based on this calculation. For each sample, the mean value of technical replicate spots was calculated for each lectin. Differentially reactive lectins between biological samples were identified using threshold criteria (fold change > 1.2, *p* < 0.05). The glycan recognition specificity information of lectins in the lectin microarray is provided in Supplementary Table 13.

### Transcriptome sequencing and analysis

Lung tissues from offspring at PND14 and PND56 were processed for RNA sequencing by Shanghai Biotechnology Corporation (Shanghai, China) (see Supplementary Text 1 for details). Differential gene expression analysis was performed using the edgeR package (v: 4.4.2) in R (v: 4.4.2) under RStudio with thresholds of |log_2_ (fold change)| > log_2_1.5 and *p* < 0.05. Functional enrichment of DEGs was assessed through Kyoto Encyclopedia of KEGG pathway analysis using the clusterProfiler package (v: 4.14.3) in R, with significance defined as *p* < 0.05. GSEA was conducted to identify pathway alterations, with visualization performed using R and the online platform https://www.bioinformatics.com.cn.

### Integration of lectin specificity with transcriptomic pathways

To investigate the regulatory interplay between lectin-binding glycans and transcriptional networks, glycosyltransferase genes were systematically curated from the HUGO Gene Nomenclature Committee (HGNC) Glycosyltransferase Subfamily Database (https://www.genenames.org/data/genegroup/#!/group/424, last accessed: 11/06/24). Hub genes were identified as the intersection of glycosyltransferase-encoding genes and DEGs. Putative transcription factors (TFs) regulating these hub genes were predicted using PROMO (http://alggen.lsi.upc.es/cgi-bin/promo_v3/promo/promoinit.cgi?dirDB=TF_8.3, last accessed: 11/11/24) with stringent parameters: species restricted to *Mus musculus*, matrix dissimilarity rate ≤5%, and a conservation filter (phylogenetic distance <0.85). TF binding site predictions were validated against the JASPAR 2024 database (https://jaspar.elixir.no/, last accessed: 12/20/24). The interaction network between hub genes and TFs was visualized and analyzed using Gephi software.

### FITC-conjugated DSL immunofluorescence staining

Glycosylation patterns in lung tissue were analyzed using fluorescein isothiocyanate (FITC)-conjugated Datura stramonium lectin (DSL, Vector Laboratories, FL-1181-2). Detailed experimental protocols are provided in Supplementary Text 1. Fluorescence intensity was quantified across six random fields per sample using ImageJ.

### Immunofluorescence staining for FcRn, IgG, and lectins (GHA/TL) in yolk sac tissue

To examine the spatial association between the neonatal FcRn, IgG, and exposure‑specific glycosylation signatures at the maternal‑fetal interface, uterine horns were excised and embryos were collected at E18.5. Fetal sex was determined by dissection and morphological identification of gonads (uterus/ovaries or testes), and the corresponding yolk sac–placenta tissue was collected separately by sex, triple‑label immunofluorescence was performed on yolk sac tissue. Tissue sections were stained with anti‑FcRn primary antibody (2.5 μg/mL; Boster Biological Technology, Wuhan, China) followed by a Cy3‑conjugated secondary antibody (10 μg/mL; Boster Biological Technology), total mouse IgG was detected using a Fluoro 647‑conjugated goat anti‑mouse IgG (10 μg/mL; Boster Biological Technology), and glycans were labeled with FITC‑conjugated GHA lectin (0.5 ppm O_3_ group, 10 μg/mL; EY Laboratories, San Mateo, CA, USA) or TL lectin (1 ppm O_3_ group, 10 μg/mL; EY Laboratories). Detailed protocols are provided in Supplementary Text 1. Fluorescence signals were captured with an Axio Observer 7 fluorescence microscope and analyzed for co‑localization and intensity using ImageJ Fiji.

### Molecular docking analysis

To investigate the structural impact of IgG glycosylation on FcγRIII binding, molecular docking analysis was performed. The mouse FcγRIII homology model was generated using Swiss-Model (https://swissmodel.expasy.org, last accessed: 01/02/25) with default parameters. The IgG crystal structure (PDB:1IGT) was processed in PyMOL (v: 2.5.5) to remove glycan chains (residues 1–9 from E/F chains), creating a deglycosylated model. DSL lectin-specific glycans (GlyTouCan IDs: G42830YF, G86157UQ, G75236EF) were selected via the Glycosmos Portal (https://glycosmos.org, last accessed: 01/04/25) and computationally integrated into the N-glycosylation site of the deglycosylated IgG model using GlycoShape’s Re-Glyco module (https://glycoshape.org/reglyco, last accessed: 01/04/25). The glycan structures with GlyTouCan IDs G42830YF, G86157UQ, and G75236EF were matched to the structures in the GlycoShape database with IDs G88800UV, G57277JN, and G56655CC, respectively, producing glycosylated variants (the glycan structure with ID G56655CC cannot generate IgG glycosylated variants in the Re-Glyco module). Fc domains (B/D chains, residues 224–447) were isolated by PyMOL chain segmentation for docking. Protein-protein docking was performed using GRAMM (https://gramm.compbio.ku.edu, last accessed: 01/04/25), with FcγRIII as the receptor and IgG-Fc structures as ligands. The top complex (lowest ΔG) from each group was selected for interfacial analysis via PDBePISA (https://www.ebi.ac.uk/pdbe/pisa, last accessed: 01/04/25). ΔG, solvation free energy gain upon interface formation (Δ^i^G), hydrogen bond contribution to protein binding free energy (ΔG_HB_), and salt bridge contribution to protein binding free energy (ΔG_SB_) were quantified. Molecular structures were visualized using PyMOL.

### Statistical analysis

Data are presented as mean ± standard deviation (SD). Normality was assessed using the Shapiro–Wilk test, and homogeneity of variances was verified using Levene’s test. For multi-group analyses, one-way ANOVA was performed. When both normality and homogeneity of variances assumptions were satisfied, Fisher’s Least Significant Difference (LSD) test was applied for post-hoc pairwise comparisons. For analyses involving only three groups where comparisons were exclusively made between each exposure group and the control group, the LSD test was used. For multi-group comparisons in the allergy challenge model, the Benjamini–Hochberg false discovery rate (FDR) procedure was applied to the raw *p*-values obtained from the LSD tests to control for multiple testing. When homogeneity of variances was violated after data transformation, Tamhane’s T2 test was used for post-hoc pairwise comparisons. When data were not normally distributed, data transformations were first attempted. If the transformed data met the assumptions of normality and homoscedasticity, the parametric procedures described above were applied. If, after transformation, the data remained non-normal, non-parametric methods were employed, assessing overall group differences using the Kruskal–Wallis test followed by Dunn’s post-hoc test for pairwise comparisons. As with the parametric analyses, multiplicity was controlled using the Benjamini–Hochberg FDR procedure. For two-group comparisons, the Student’s *t* test was applied when both normality and homogeneity of variances assumptions were satisfied; when variances were unequal but normality held, Welch’s *t* test was used. For data that did not follow a normal distribution, the Mann–Whitney U test was performed. All analyses were performed using SPSS (v: 26.0), and statistical significance was defined as *p* < 0.05. Data visualization was performed using  GraphPad Prism (v: 8.0.1) and R (v: 4.4.2). Schematic illustrations were created using BioGDP.com^80^.

### Reporting summary

Further information on research design is available in the Nature Portfolio Reporting Summary linked to this article.

## Supplementary information

Supplementary Information

Reporting Summary

Transparent Peer Review file

## Source data

Source Data

## Data Availability

The RNA sequencing data generated in this study have been deposited in the Gene Expression Omnibus (GEO) database under accession code GSE296733. The cohort and demographic data are available under restricted access for safeguarding participant privacy and ethical confidentiality; access can be obtained by submitting a reasonable academic research request to the corresponding author within a reasonable timeframe and executing a formal Data Use Agreement. All other data are available in the article and its Supplementary files or from the corresponding author upon request. Source data are provided with this paper.
