# Supplementary material for: Elevated ozone activates maternal immunity to drive offspring IgG-mediated anaphylaxis through IgG glycosylation alterations
Source: Nat Commun. 2026 Sep 23;17:11063. doi: 10.1038/s41467-026-77756-x (PMC13691101; doi:10.1038/s41467-026-77756-x)
Supplement: Supplementary file 1 [file 41467_2026_77756_MOESM1_ESM.pdf]

Supplementary Materials

Elevated Ozone Activates Maternal Immunity to Drive Offspring IgG-Mediated Anaphylaxis through IgG Glycosylation Alterations

Xiaoyun Wu[1], Zhihua Gong[2], Yuchai Tian[1], Xiaomin Liang[1], Mudan Wu[3], Huizhen Zhu[1], Jiyue Zhang[1], Yangcheng Hu[1], Bin Li[1], Pannan Li[2], Xiaotong Ji[4*], Huifeng Yue[1*], Nan Sang[1*]

* Corresponding author: Xiaotong Ji, Huifeng Yue, Nan Sang

E-mail: jixiaotong@sxmu.edu.cn; yuehuifeng@sxu.edu.cn; sangnan@sxu.edu.cn

This PDF file includes:

Methods

Figures S1 to S21

Tables S1 to S13

Contents

Text S1. Methods

Figure. S1. Flowchart of participant inclusion and exclusion criteria for the retrospective study.

Figure. S2. Effects of O_3 exposure on gestational parameters in pregnant dams.

Figure. S3. Effects of O_3 exposure on fetal and placental parameters at E18.5.

Figure. S4. Serum inflammatory cytokine profiles in the ASA model offspring.

Figure. S5. Pathological assessment of MIA-induced airway remodeling in the ASA model offspring.

Figure. S6. Serum inflammatory cytokine profiles in the PSA model offspring.

Figure. S7. CBC analysis in the PSA model.

Figure. S8. Pathological assessment of MIA-induced pulmonary alterations in the PSA model offspring.

Figure. S9. MIA-induced pulmonary functional impairment in the PSA model offspring.

Figure. S10. t-SNE analysis of lung inflammatory cells in female ASA offspring.

Figure. S11. Assessment of FcγRIII/FcγRIIB$^+$ cell ratios in pulmonary inflammatory cell subsets of ASA model offspring.

Figure. S12. mRNA expression of complement-related genes in female ASA offspring lungs.

Figure. S13. t-SNE analysis of lung inflammatory cells in PSA model offspring.

Figure. S14. Co-localization and quantification of FcRn, IgG, and differential lectin staining in E18.5 mouse yolk sacs.

Figure. S15. CBC analysis in female MIA offspring at PND14.

Figure. S16. MIA-driven pulmonary immune cell alterations in offspring at PND14.

Figure. S17. MIA-driven pulmonary pathophysiological alterations in female offspring at PND14.

Figure. S18. IgG glycosylation profiles and differential lectin binding in male offspring at PND28 and PND42.

Figure. S19. Top 20 KEGG pathways enriched in DEGs from male offspring lungs at PND56.

Figure. S20. Gating strategy for the identification of inflammatory cell and T cell subsets.

Figure. S21. Representative fluorescence-minus-one (FMO) control histograms for flow cytometry gating.

Table S1. Characteristics of the participants in the study (n = 5,299).

Table S2. Trimester-specific associations between ambient $PM_{2.5}$ exposure and increased risks of hematological parameter abnormalities.

Table S3. Trimester-specific associations between ambient PM_{10} exposure and increased risks of hematological parameter abnormalities.

Table S4. Trimester-specific associations between ambient NO_2 exposure and increased risks of hematological parameter abnormalities.

Table S5. Trimester-specific associations between ambient SO_2 exposure and increased risks of hematological parameter abnormalities.

Table S6. Associations of trimester-specific O_3 exposure with risks of hematological parameter abnormalities: Single-pollutant and $PM_{2.5}$ adjusted models

Table S7. Associations of trimester-specific O_3 exposure with risks of hematological parameter abnormalities: Single-pollutant and NO_2 adjusted models

Table S8. Associations of trimester-specific O_3 exposure with risks of hematological parameter abnormalities: Single-pollutant and SO_2 adjusted models

Table S9. Associations of trimester-specific O_3 exposure with risks of hematological parameter abnormalities: Single-pollutant and PM_{10} adjusted models

Table S10. Associations of trimester-specific O_3 exposure with risks of hematological parameter abnormalities: Base model and HGB adjusted model.

Table S11. Antibodies and dyes used in flow cytometry analysis.

Table S12. Primers used for qPCR analysis.

Table S13. Lectin and glycan identity in the microarray.

Supplementary Text

Text S1. Methods

Cytokine quantification by Luminex

Serum samples (50 μL/well) were mixed 1:4 with assay buffer, then loaded into 96-well plates containing pre-dispensed antibody-conjugated magnetic beads. Serially diluted standards and quality controls were run in parallel. Plates were sealed and incubated at 25°C with orbital shaking (100 ×g) for 1 hour, followed by three automated wash cycles using 100 μL/well wash buffer. Detection antibodies (50 μL/well) were then added, and plates were incubated under the same conditions for 1 h. After additional washes, 50 μL/well of phycoerythrin (PE)-conjugated streptavidin was added for 30 minutes. Following a final wash, 150 μL/well Bio-Plex sheath fluid was added, and the samples were immediately analyzed on a Luminex X-200 system. Raw fluorescence signals were processed using Milliplex Analyst software (v: 5.1, MilliporeSigma), with blank controls (assay buffer only) and spike-in recombinant cytokine controls used to validate assay specificity and sensitivity. Analyte concentrations were reported as absolute values (pg/mL). The entire Luminex assay process was performed by Shanghai LabEx Biotech Co., LTD. Prior to downstream statistical evaluation, batch effect correction was applied to the quantified cytokine levels using a control-based median-centering alignment approach.

Pulmonary Function Assessment

For invasive lung function analysis in PSA and ASA model mice, anesthetized

animals (intraperitoneal sodium pentobarbital, 50 mg/kg) underwent tracheostomy with insertion of an 18-gauge tracheal cannula connected to the AniRes2005 system (v: 3.5, Beijing Bestlab Technology Co., Ltd.). Ventilator parameters were set to respiratory rate 90 breaths/min, inspiration/expiration ratio 1:1.5, tidal volume 5 mL/kg, and positive end-expiratory pressure 8 cm H_2O (0.78 kPa). Key spirometric parameters — including forced vital capacity (FVC), forced expiratory volume in 0.1 second (FEV0.1), and maximal mid-expiratory flow (MMF) — were continuously monitored in real-time through system-integrated (AniRes Suite), with respiratory waveforms monitored to exclude artifactual signals induced by airway secretions or mechanical noise. Five consecutive measurements per mouse were averaged for statistical analysis.

To mitigate invasive procedures in immature lungs, unrestrained whole-body plethysmography (WBP) was performed on PND14 neonatal pups using a Tow-Int Tech system (Tower International Technology, Shanghai, China). Conscious mice were acclimatized for 2 minutes in a transparent sealed chamber (200 mL volume) with constant airflow (500 mL/minute). Respiratory waveforms were continuously monitored via high-sensitivity pressure transducers over a 10-minute stable recording period. Key parameters including inspiratory time (TI), expiratory time (TE), peak inspiratory flow (PIF), peak expiratory flow (PEF), respiratory frequency (F), tidal volume (vt), minute ventilation (mv), expiratory flow at 50% tidal volume (EF50), enhanced pause (Penh), alveolar ventilation (AV), total respiratory cycle time (TR), and ratio of peak expiratory flow (Rpef) were extracted using TowerPleth software.

Superoxide Dismutase (SOD) Activity Assay

30 mg of lung tissue was homogenized on ice in pre-chilled phosphate-buffered saline (PBS, 0.1 M, pH 7.4) and centrifuged at 1200 ×g for 10 minutes at 4°C. The supernatant was diluted 40-fold, and 20 μL aliquots were mixed with 220 μL reaction mixture in 96-well plates. After 20 minutes of incubation at 37°C protected from light, absorbance at 450 nm was measured using a microplate reader. SOD activity was calculated using the inhibition rate method. Total protein concentration was determined via Bradford assay (Solarbio, Beijing, China) with BSA standards. Final SOD activity values were normalized to protein content and expressed as units per milligram of protein (U/mg prot).

Western Blot Analysis

Lung tissues (40 mg) were flash-frozen in liquid nitrogen and homogenized in ice-cold RIPA lysis buffer (Epizyme, Shanghai, China) supplemented with 1% protease and phosphatase inhibitor cocktail (Epizyme, Shanghai, China). After 30 minutes of ice-cold lysis, lysates were centrifuged at 11400 ×g for 15 minutes at 4°C, and supernatants were quantified via Bradford assay with BSA standards. Equal protein loads (36 μg) were resolved on 10% SDS-PAGE gels and transferred to 0.45 μm PVDF membranes (Merck Millipore). Membranes were blocked with 3% BSA in TBST for 1 hour at room temperature, followed by overnight incubation at 4°C with primary antibodies: anti-c-Fos (rabbit monoclonal, clone EPR21930-238, Cat. No. ab222699, Abcam; 0.228 μg/mL), anti-c-Jun (rabbit monoclonal, clone EP693Y, Cat. No. ab40766, Abcam; 0.102 μg/mL), anti-phospho-c-Fos (rabbit polyclonal, Cat. No. bs-12910R, Bioss; 1 μg/mL), anti-phospho-c-Jun (rabbit monoclonal, clone IGA-10, Cat. No. BM4845,

Boster; 0.5 μg/mL), and β-actin (rabbit polyclonal, Cat. No. bs-0061R, Bioss; 0.2 μg/mL). After TBST washes, membranes were incubated with HRP-conjugated goat anti-rabbit secondary antibody (Cat. No. bs-0295G-HRP, Bioss; 0.2 μg/mL) for 1 hour at room temperature. Chemiluminescent signals were developed using ultrasensitive ECL chemiluminescence kit (Cat. No. SW134, Seven, Beijing, China) and imaged on a Bio-Rad Gel Imager System (Bio-Rad, USA). Band intensities were normalized to β-actin and quantified using Fiji/ImageJ.

RNA sequencing

Total RNA was extracted using the MJzol Animal RNA Isolation Kit (Majorbio), purified with RNAClean XP Kit (Beckman Coulter) and RNase-Free DNase Set (QIAGEN), and quantified via Agilent 2100 Bioanalyzer. Libraries were constructed through fragmentation, cDNA synthesis, end repair, and Illumina adapter ligation, followed by size selection using an Agilent 4200 TapeStation (Agilent Technologies). Qualified libraries were sequenced on an Illumina NovaSeq6000 platform with paired-end 150 bp reads (PE150). Clean reads were aligned to the mouse reference genome (GRCm38) via HISAT2 v2.0.4 with default parameters. Gene expression levels were quantified as fragments per kilobase of exon per million mapped reads (FPKM).

FITC-Conjugated DSL Immunofluorescence Staining

Lung sections were deparaffinized with xylene gradients, rehydrated through an ethanol series, and subjected to antigen retrieval in sodium citrate buffer at 95°C for 15 minutes. After permeabilization, endogenous peroxidase blocking, and serum blocking,

sections were incubated overnight at 4°C with FITC-DSL (10 μg/mL in PBS) under light-protected conditions. Unbound lectin was removed by three PBS washes, and nuclei were counterstained with DAPI (Boster Biological Technology, Wuhan, China) for 10 minutes. Sections were mounted with an anti-fade mounting medium (Boster Biological Technology, Wuhan, China) and imaged using an Axio Imager M2 fluorescence microscope (Zeiss, Jena, Germany) with FITC and DAPI filter sets.

Immunofluorescence Staining for FcRn, IgG, and Lectins (GHA/TL) in Yolk Sac Tissue

At E18.5, uterine horns were excised and embryos were collected. Fetal sex was determined by dissection and morphological identification of gonads (uterus/ovaries or testes). The corresponding yolk sac–placenta tissue was collected separately by sex and fixed overnight in 4% paraformaldehyde. Tissues were then paraffin-embedded and sectioned at 4–5 μm thickness. Prior to staining, sections were deparaffinized in xylene and rehydrated through a graded ethanol series, and subjected to antigen retrieval in sodium citrate buffer at 95°C for 15 minutes. After permeabilization, endogenous peroxidase blocking, and serum blocking, sections were incubated overnight at 4 °C in the dark with a primary rabbit anti-FcRn antibody (2.5 μg/mL; Boster Biological Technology, Wuhan, China). Following rewarming and three washes with PBS, sections were co-incubated for 1 hour at room temperature in the dark with a Cy3-conjugated goat anti-rabbit IgG secondary antibody (10 μg/mL; Boster Biological Technology) and an Fluoro 647-conjugated goat anti-mouse IgG secondary antibody (10 μg/mL; Boster Biological Technology). To detect specific glycosylation, after PBS washing, sections

from the control and 0.5 ppm O_3 exposure groups were incubated for 2 hours at room temperature in the dark with FITC-labeled GHA lectin (10 µg/mL; EY Laboratories, San Mateo, CA, USA). Sections from the control and 1 ppm O_3 exposure groups were incubated under the same conditions with FITC-labeled TL lectin (10 µg/mL; EY Laboratories). Following another wash step, nuclei were counterstained with DAPI (Boster Biological Technology) for 10 minutes. Stained sections were mounted with anti-fade mounting medium (Boster Biological Technology) and imaged using an Axio Observer 7 fluorescence microscope (Zeiss, Jena, Germany) equipped with standard FITC, DAPI, Cy3, and Alexa Fluor 647 filter sets.

Supplementary Figures

Supplementary Fig. 1

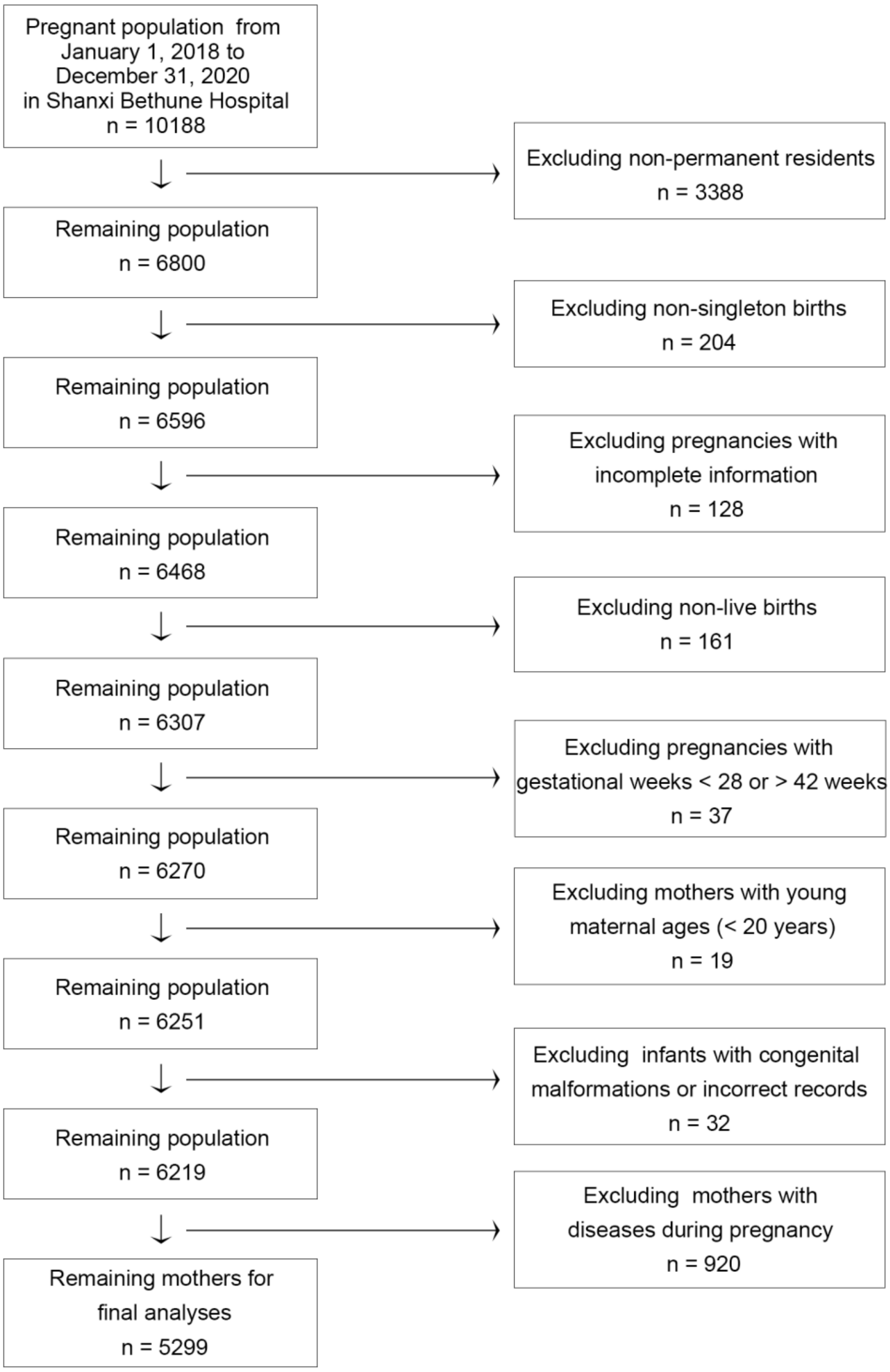

Figure S1. Flowchart of participant inclusion and exclusion criteria for the retrospective study.

Supplementary Fig. 2

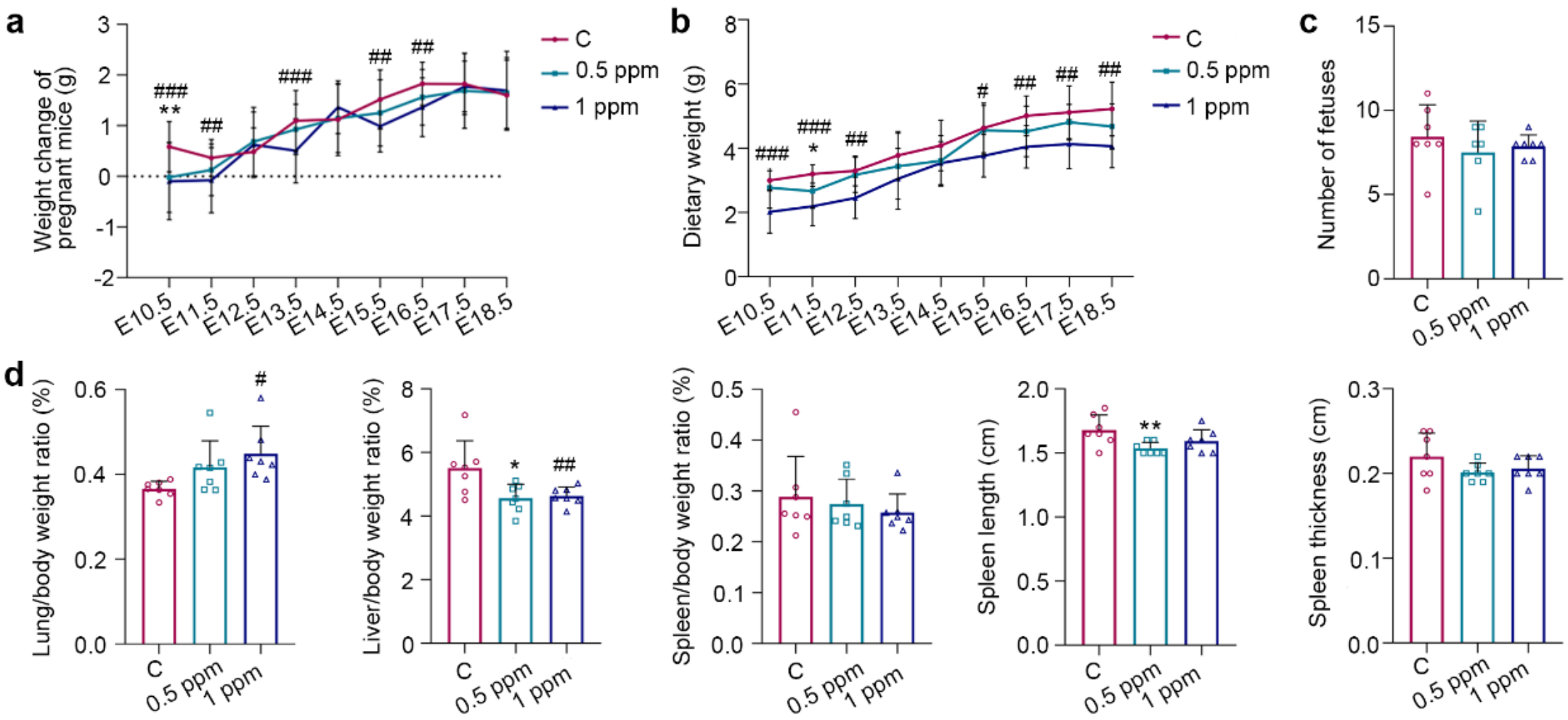

Figure S2. Effects of O_3 exposure on gestational parameters in pregnant dams. a Body weight changes in pregnant dams post-O_3 exposure (n = 24, 26, 25; ordered by control, 0.5 ppm, and 1 ppm groups). **b** Dietary alterations post-O_3 exposure (n = 10, 11, 11; ordered by control, 0.5 ppm, and 1 ppm groups). **c-d** Impacts of O_3 exposure on **(c)** litter size and **(d)** organ indices, including lung/body weight ratio, liver/body weight ratio, spleen/body weight ratio, spleen length, and spleen thickness (for litter size, n = 7, 6, 7; for organ indices, n = 7, 7, 7; ordered by control, 0.5 ppm, and 1 ppm groups). Data are mean ± SD, analyzed by one-way ANOVA or Kruskal-Wallis test. *$p < 0.05$, **$p < 0.01$ (0.5 ppm O_3 vs. control); $^{\#}p < 0.05$, $^{\#\#}p < 0.01$, $^{\#\#\#}p < 0.001$ (1 ppm O_3 vs. control).

Supplementary Fig. 3

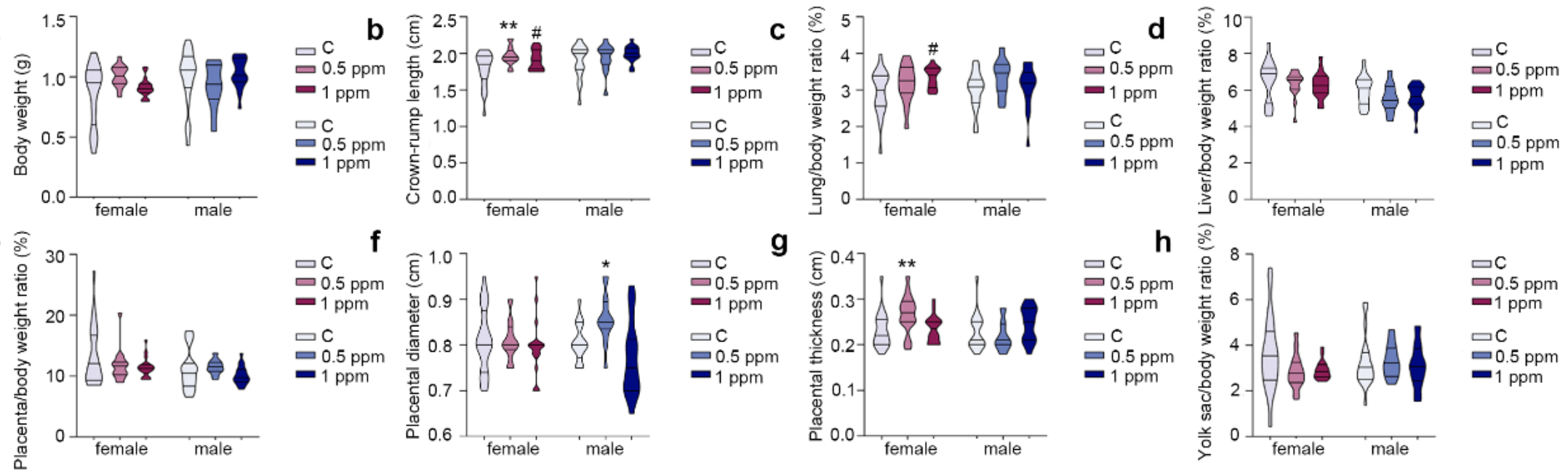

Figure S3. Effects of O_3 exposure on fetal and placental parameters at E18.5. a-h Quantitative measurements of fetal body weight **(a)**, crown-rump length **(b)**, fetal lung/body weight ratio **(c)**, fetal liver/body weight ratio **(d)**, placenta/body weight ratio **(e)**, placental diameter **(f)**, placental thickness **(g)**, and yolk sac/body weight ratio **(h)** (for **a–e** and **h**, n = 21, 15, 18 for females and n = 19, 11, 18 for males; for **f** and **g**, n = 21, 17, 18 for females and n = 18, 13, 18 for males; ordered by control, 0.5 ppm, and 1 ppm groups). Data are mean ± SD, analyzed by one-way ANOVA or Kruskal-Wallis test. *$p < 0.05$, **$p < 0.01$ (0.5 ppm O_3 vs. control); $^{\#}p < 0.05$ (1 ppm O_3 vs. control).

Supplementary Fig. 4

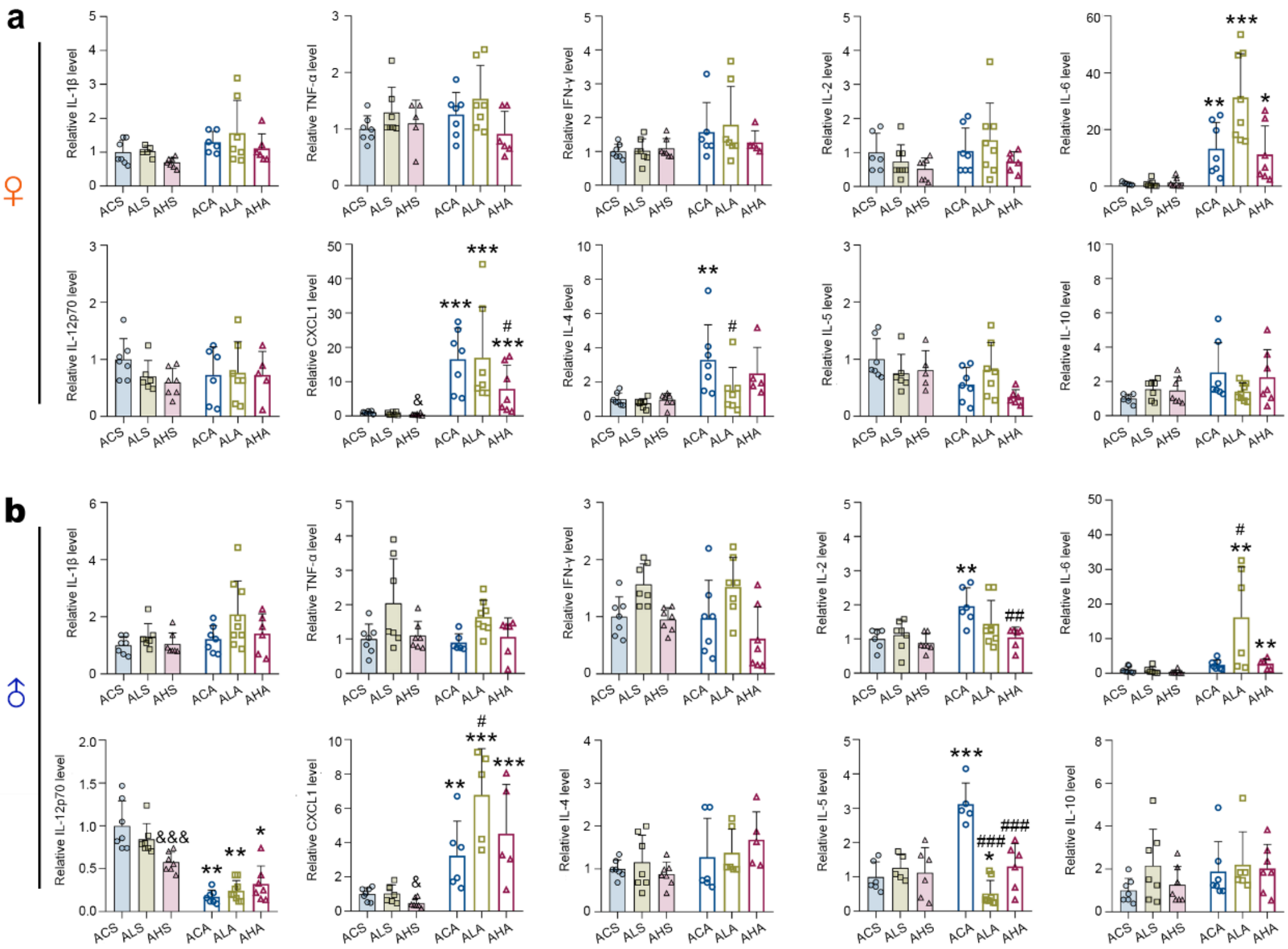

Figure S4. Serum inflammatory cytokine profiles in the ASA model offspring. a, b Quantitative analysis of multiple inflammatory cytokines in female **(a)** and male **(b)** offspring (n = 5-9 mice/group across different cytokines). Data are normalized to the ACS group and shown as mean ± SD, analyzed by one-way ANOVA or Kruskal-Wallis test. *$p < 0.05$, **$p < 0.01$, ***$p < 0.001$ (ACA vs. ACS, ALA vs. ALS, AHA vs. AHS); $^{\#}p < 0.05$, $^{\#\#\#}p < 0.001$ (ALA vs. ACA, AHA vs. ACA); $^{\&}p < 0.05$, $^{\&\&\&}p < 0.001$ (AHS vs. ACS).

Supplementary Fig. 5

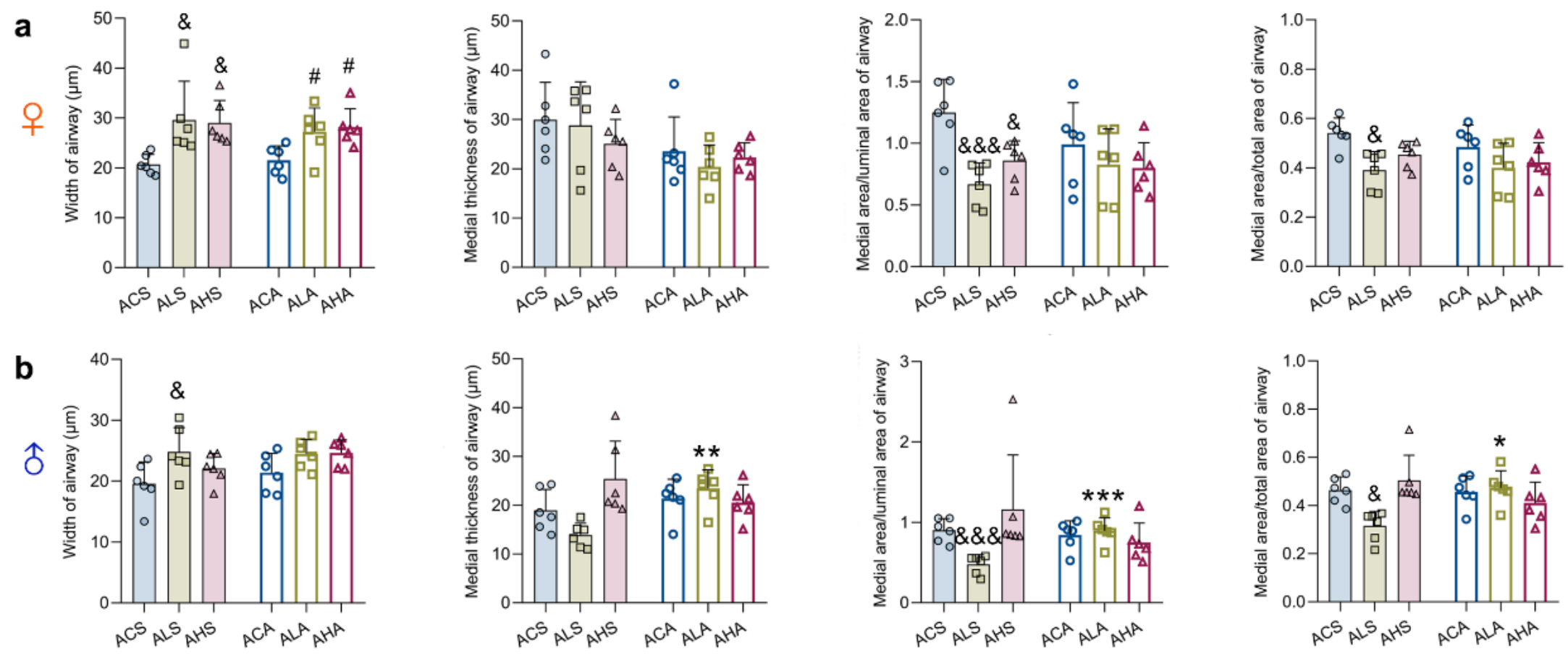

Figure S5. Pathological assessment of MIA-induced airway remodeling in the ASA model offspring. a, b Quantitative measurements of airway remodeling metrics, including width of airway, medial thickness of airway, medial area/luminal area of airway, and medial area/total area of airway in female **(a)** and male **(b)** offspring (n = 6 mice/group). Data are mean ± SD, analyzed by one-way ANOVA or Kruskal-Wallis test. *$p < 0.05$, **$p < 0.01$, ***$p < 0.001$ (ALA vs. ALS); $^{\#}p < 0.05$ (ALA vs. ACA, AHA vs. ACA); $^{\&}p < 0.05$, $^{\&\&\&}p < 0.001$ (ALS vs. ACS, AHS vs. ACS).

Supplementary Fig. 6

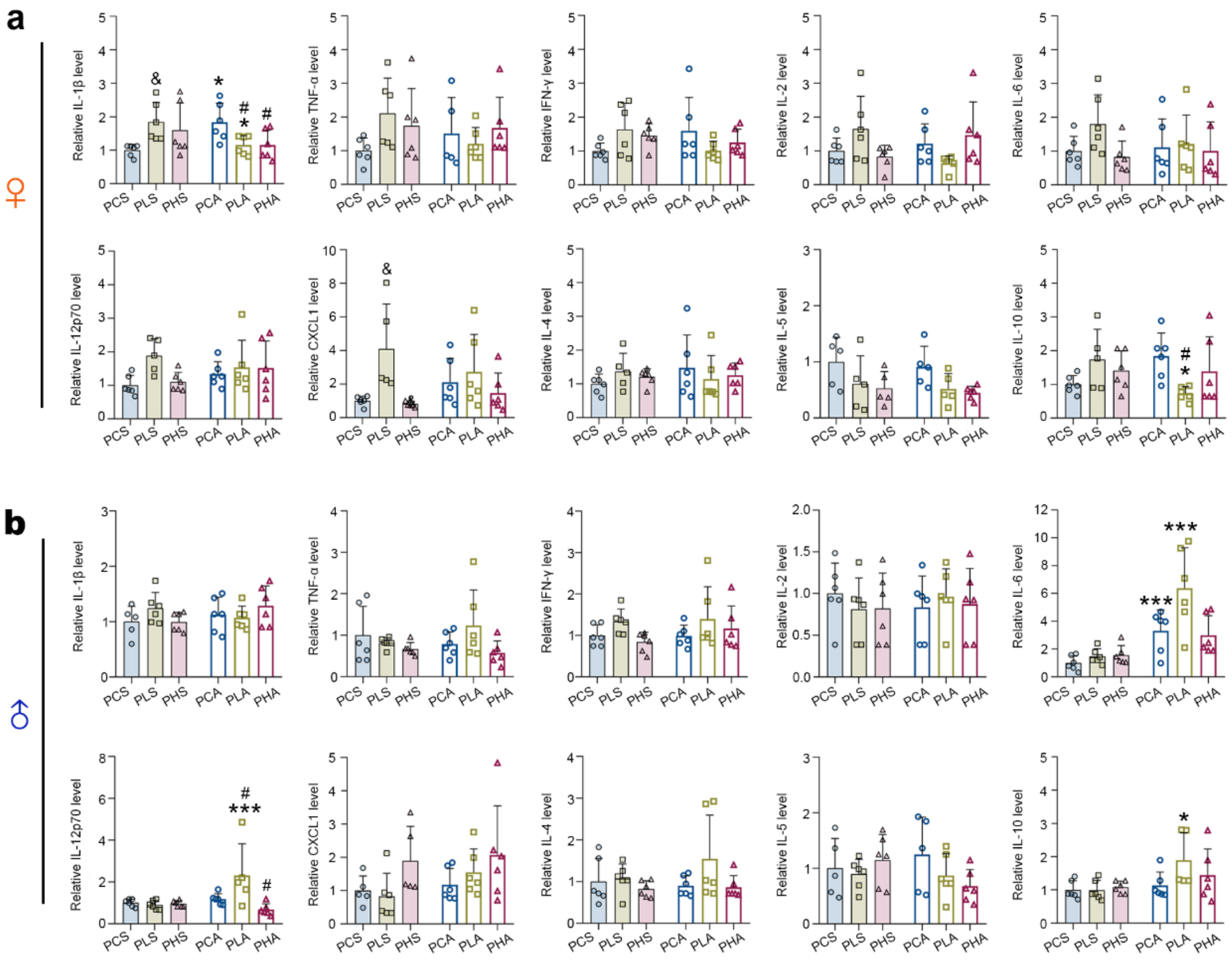

Figure S6. Serum inflammatory cytokine profiles in the PSA model offspring. a, b Quantitative analysis of multiple inflammatory cytokines in female (**a**) and male (**b**) offspring (n = 5-6 mice/group across different cytokines). Data are normalized to the PCS group and shown as mean ± SD, analyzed by one-way ANOVA or Kruskal-Wallis test. *$p < 0.05$, ***$p < 0.001$ (PCA vs. PCS, PLA vs. PLS); $^{\#}p < 0.05$ (PLA vs. PCA, PHA vs. PCA); $^{\&}p < 0.05$ (PLS vs. PCS).

Supplementary Fig. 7

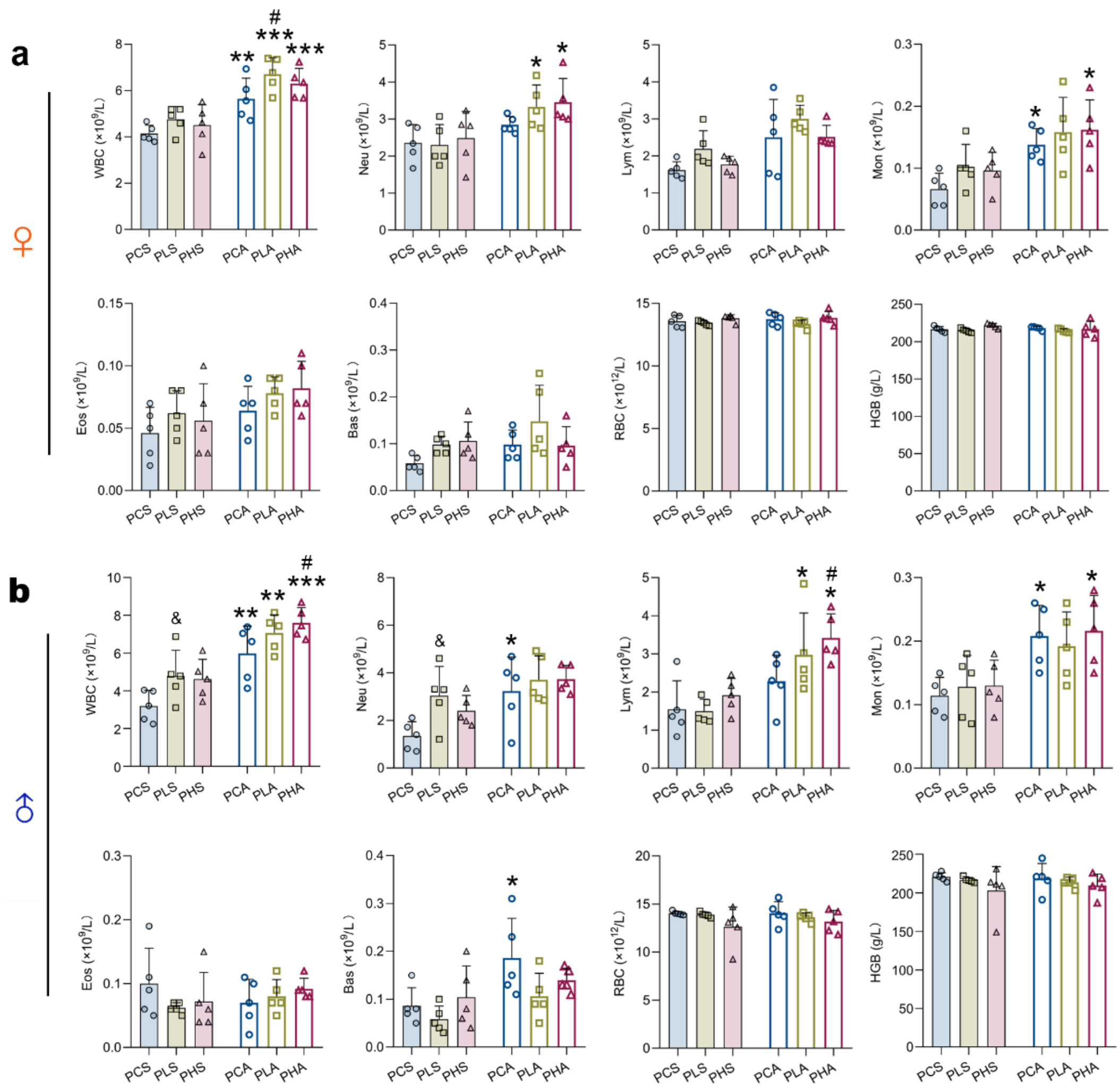

Figure S7. CBC analysis in the PSA model. a, b Quantitative measurements of CBC parameters in female (**a**) and male (**b**) offspring (n = 5 mice/group). Data are mean ± SD, analyzed by one-way ANOVA or Kruskal-Wallis test. *$p < 0.05$, **$p < 0.01$, ***$p < 0.001$ (PCA vs. PCS, PLA vs. PLS, PHA vs. PHS); #$p < 0.05$ (PLA vs. PCA, PHA vs. PCA).

Supplementary Fig. 8

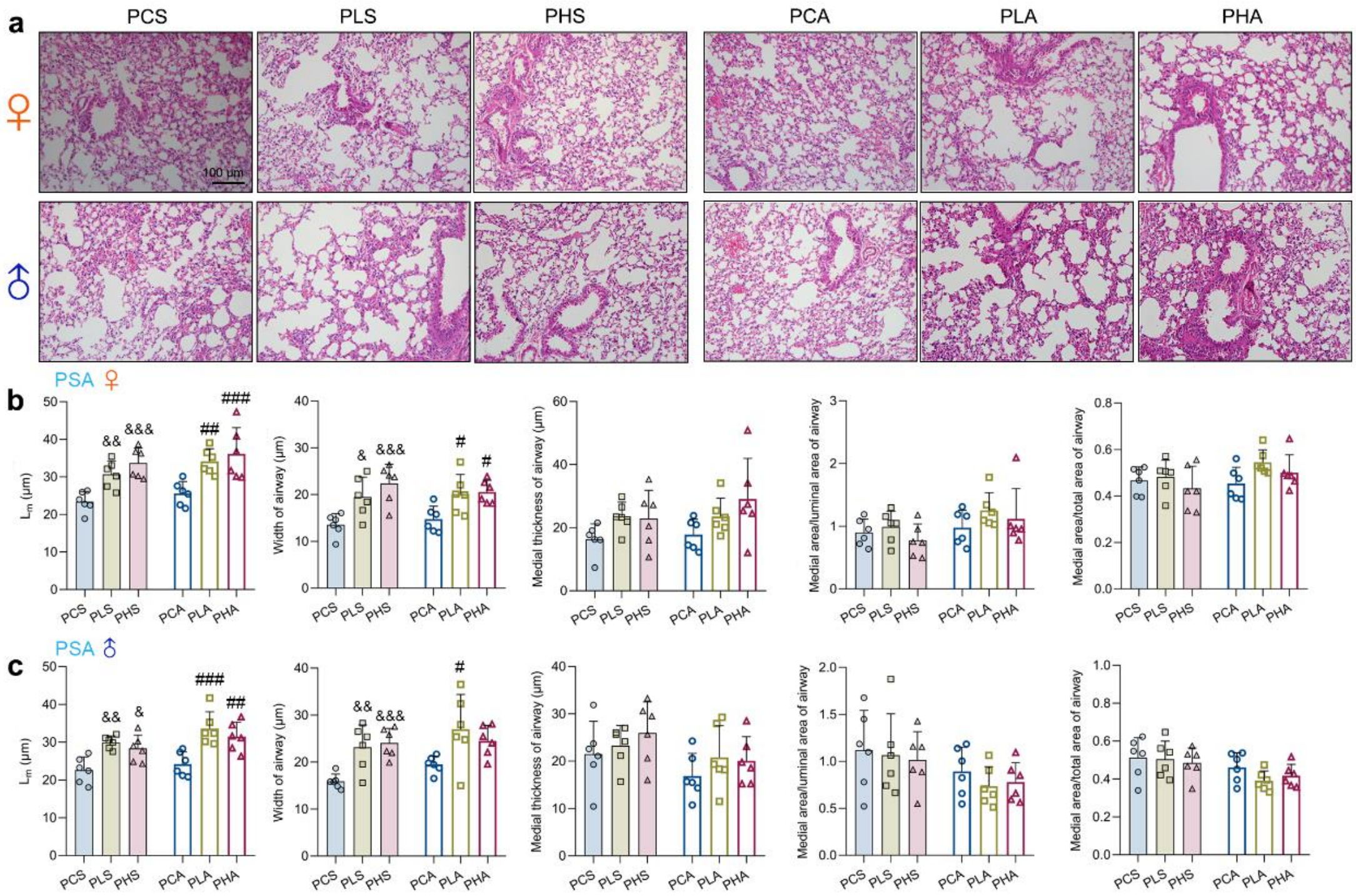

Figure S8. Pathological assessment of MIA-induced pulmonary alterations in the PSA model offspring. **a** Representative H&E-stained lung sections from female and male PSA model offspring, scale bar, 100 μm. **b, c** Quantitative measurements of airway remodeling parameters in female **(b)** and male **(c)** offspring (n = 6 mice/group). Data are mean ± SD, analyzed by one-way ANOVA or Kruskal-Wallis test. $^{\&}p < 0.05$, $^{\&\&}p < 0.01$, $^{\&\&\&}p < 0.001$ (PLS vs. PCS, PHS vs. PCS); $^{\#}p < 0.05$, $^{\#\#}p < 0.01$, $^{\#\#\#}p < 0.001$ (PLA vs. PCA, PHA vs. PCA).

Supplementary Fig. 9

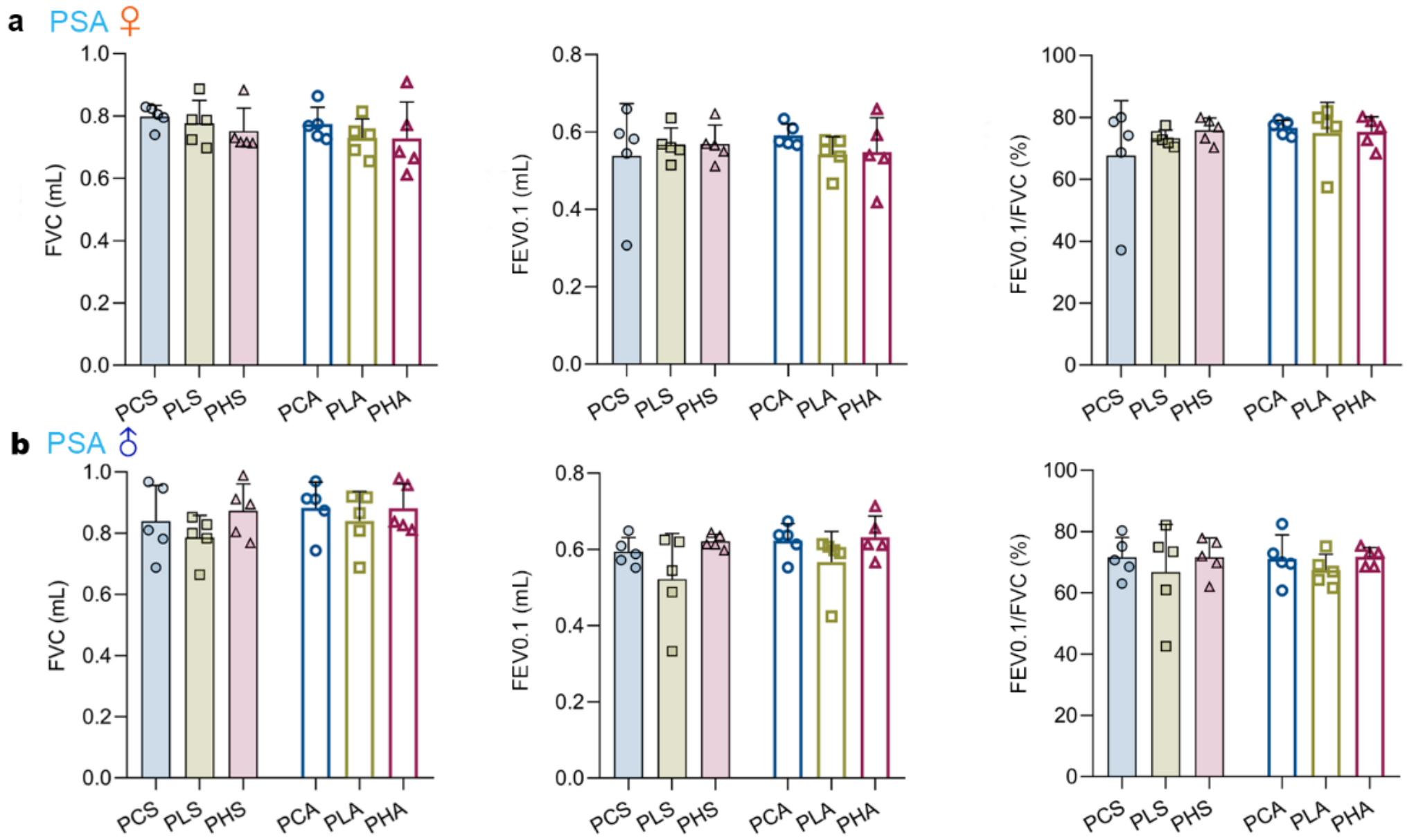

Figure S9. MIA-induced pulmonary functional impairment in the PSA model offspring. a, b Quantitative measurements of pulmonary function parameters (forced vital capacity (FVC), forced expiratory volume in 0.1 second (FEV0.1), and FEV0.1/FVC) in female (**a**) and male (**b**) offspring (n = 5 mice/group). Data are mean ± SD, analyzed by one-way ANOVA or Kruskal-Wallis test.

Supplementary Fig. 10

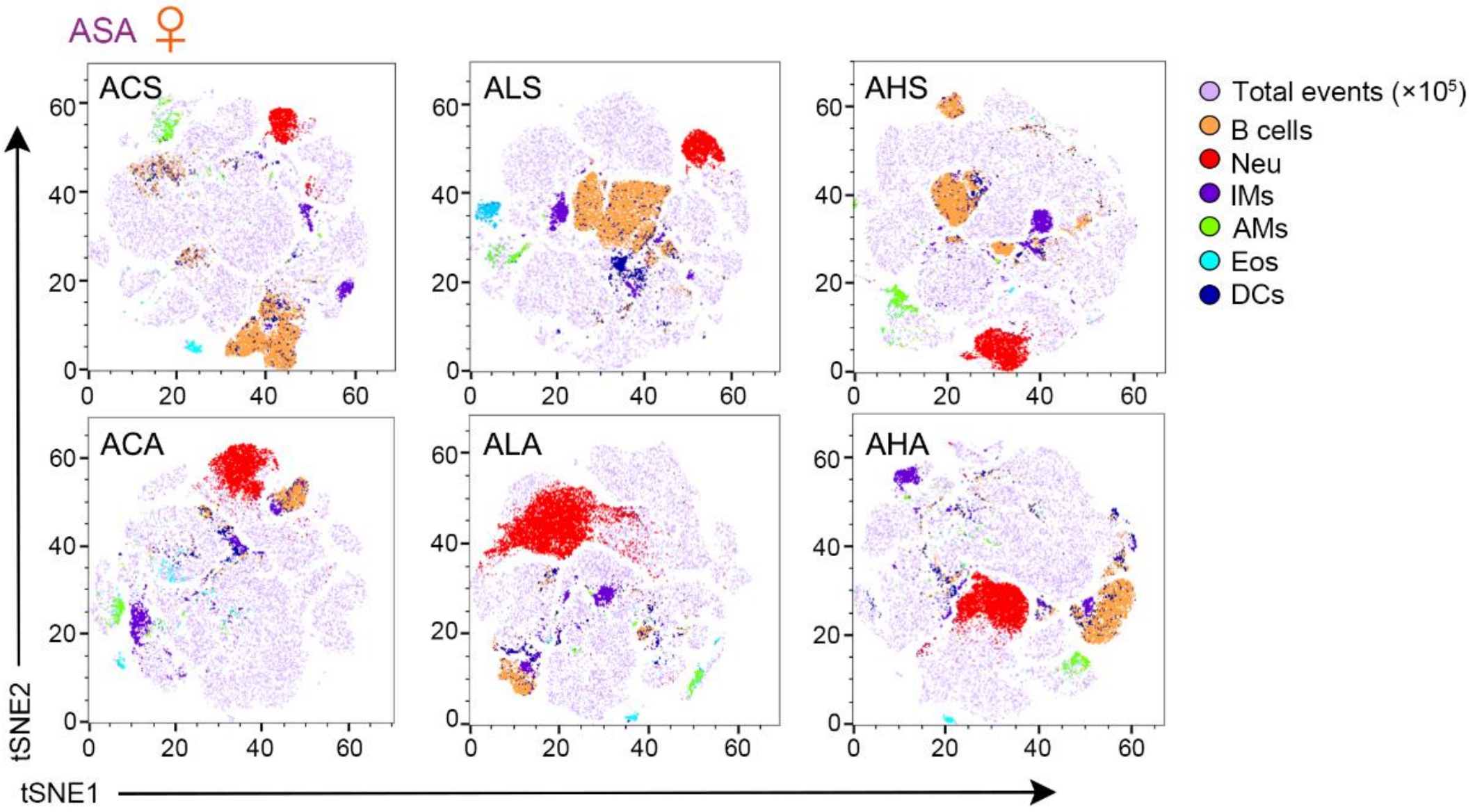

Figure S10. t-SNE analysis of lung inflammatory cells in female ASA offspring.

Supplementary Fig. 11

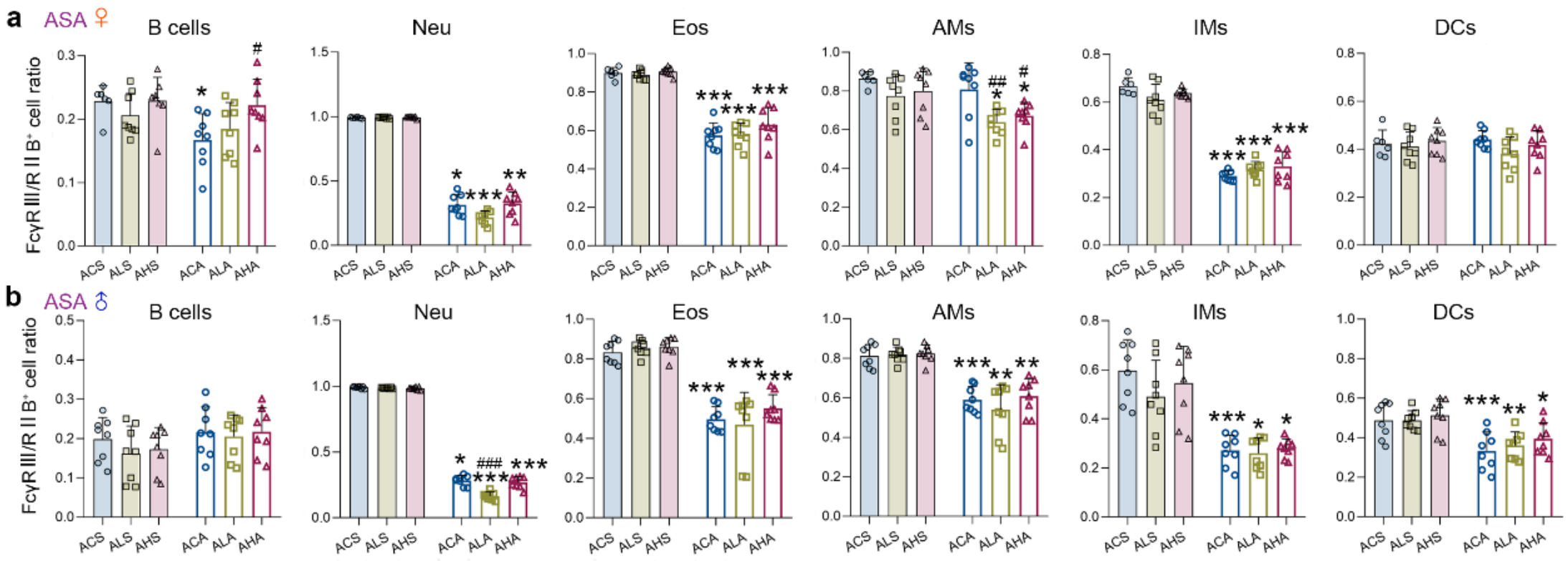

Figure S11. Assessment of FcγRIII/FcγRIIB⁺ cell ratios in pulmonary inflammatory cell subsets of ASA model offspring. a, b Quantitative analysis of cell ratios in female **(a)** and male **(b)** (n = 6, 8, 8, 8, 8, 8 for females and n = 8, 8, 8, 8, 8, 8 for males; ordered by ACS, ALS, AHS, ACA, ALA, and AHA groups). Data are mean ± SD, analyzed by one-way ANOVA or Kruskal-Wallis test. *$p < 0.05$, **$p < 0.01$, ***$p < 0.001$ (ACA vs. ACS, ALA vs. ALS, AHA vs. AHS); #$p < 0.05$, ##$p < 0.01$, ###$p < 0.001$ (ALA vs. ACA, AHA vs. ACA).

Supplementary Fig. 12

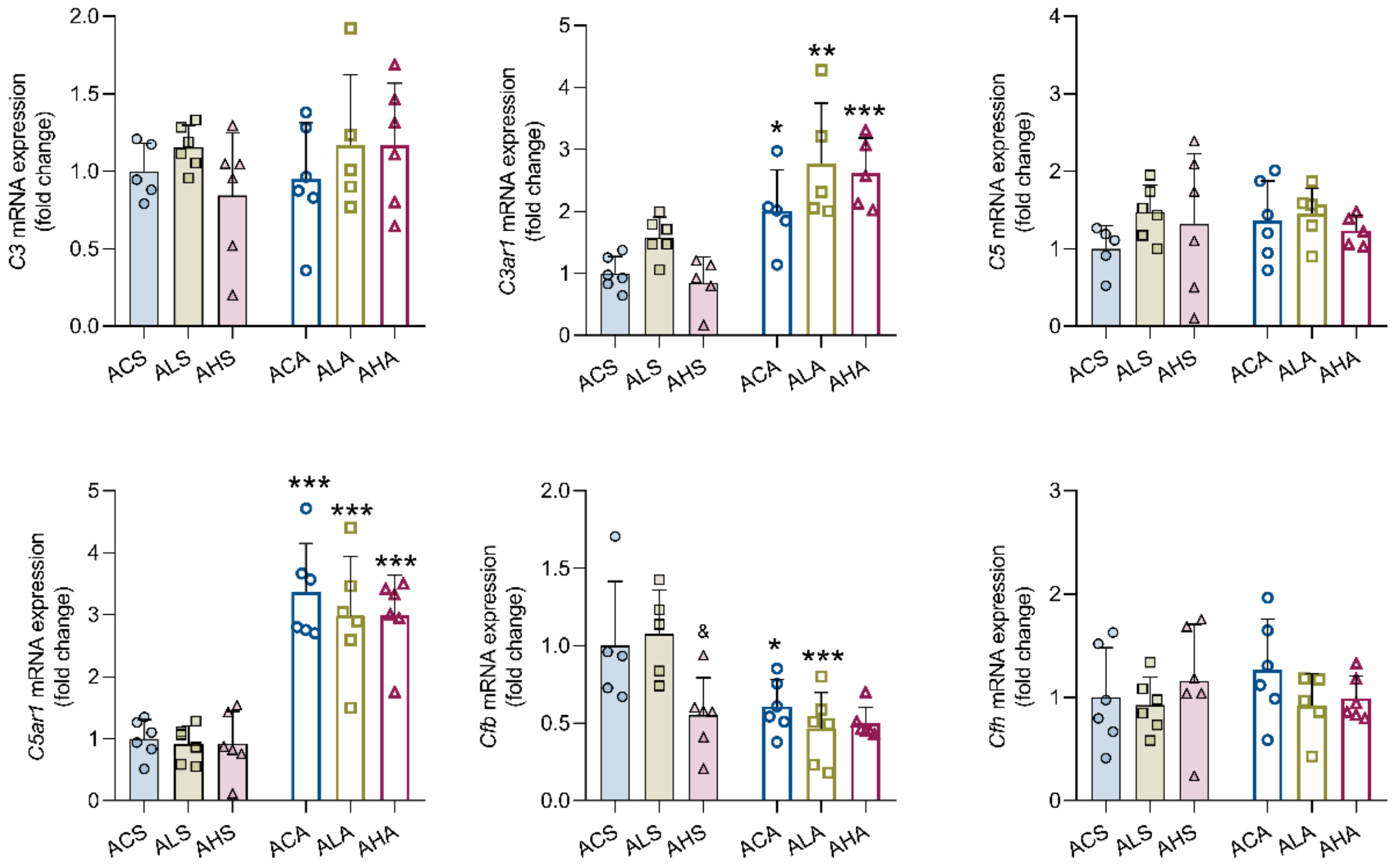

Figure S12. mRNA expression of complement-related genes in female ASA offspring lungs. Quantitative analysis of complement-related gene expression (n = 5-6 mice/group). Data are mean ± SD, analyzed by one-way ANOVA or Kruskal-Wallis test. **p* < 0.05, ***p* < 0.01, ****p* < 0.001 (ACA vs. ACS, ALA vs. ALS, AHA vs. AHS); &*p* < 0.05 (AHS vs. ACS).

Supplementary Fig. 13

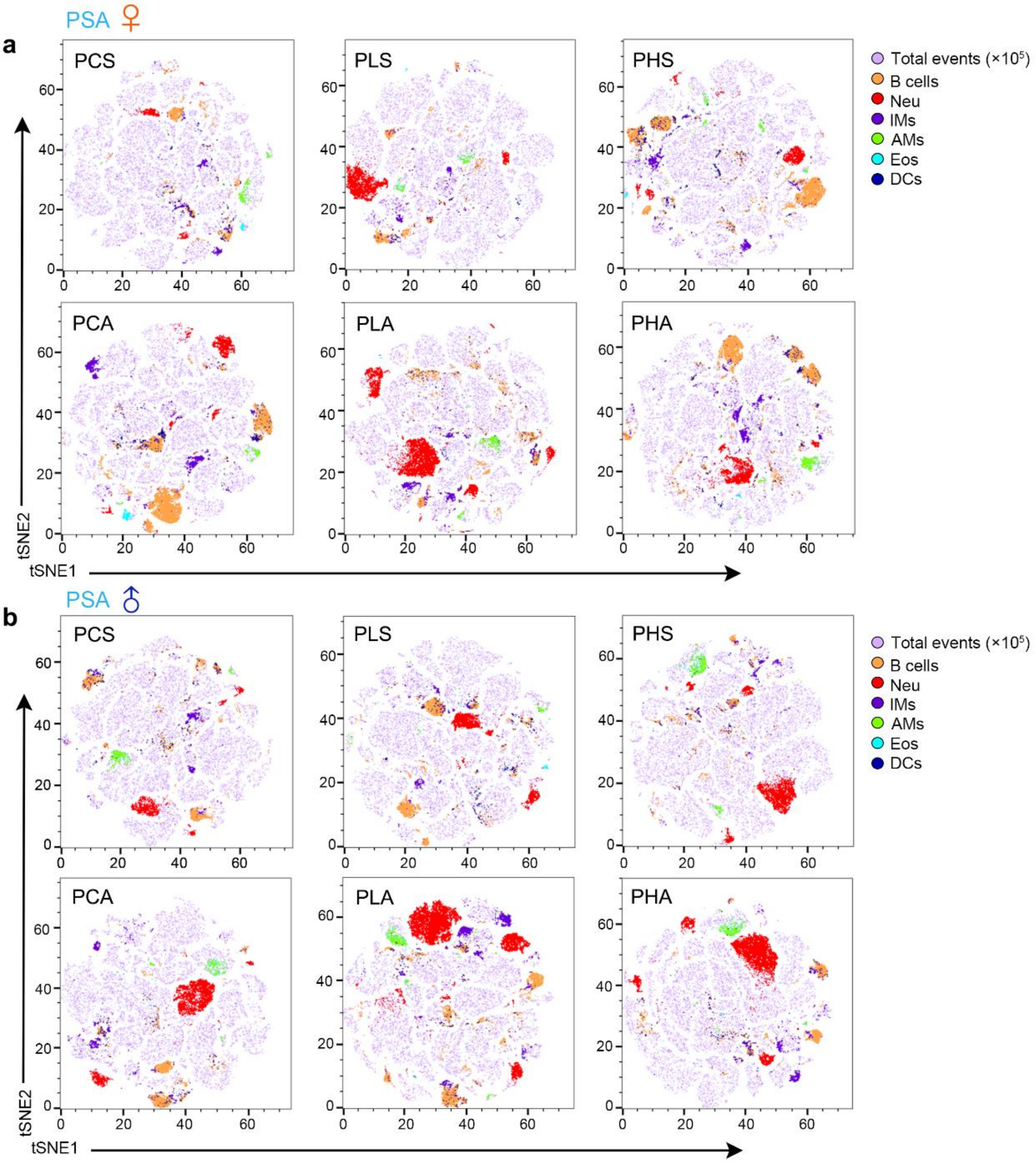

Figure S13. t-SNE analysis of lung inflammatory cells in PSA model offspring. a, b Visualization of lung inflammatory cell clusters in female **(a)** and male **(b)** offspring.

Supplementary Fig. 14

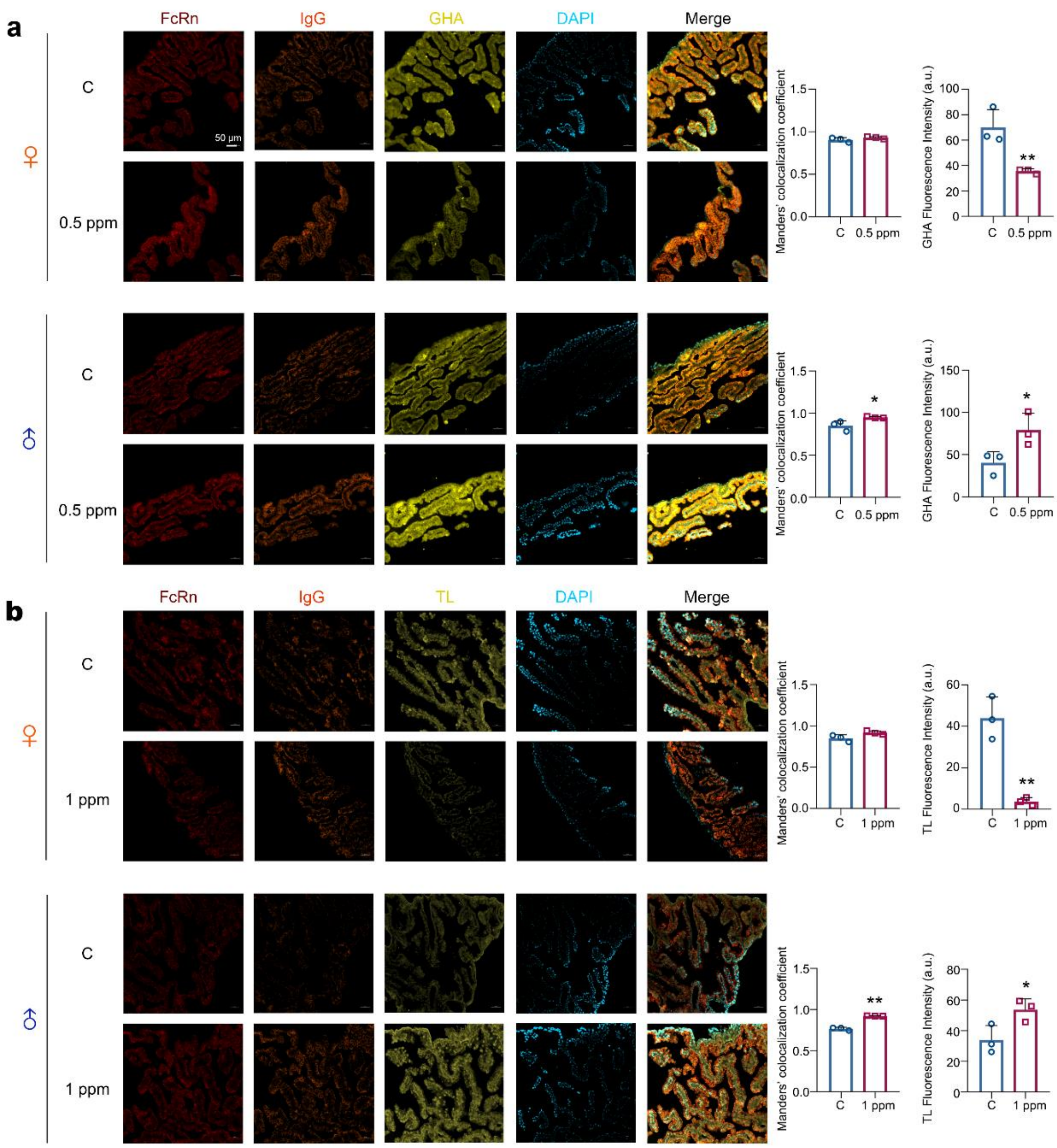

Figure S14. Co-localization and quantification of FcRn, IgG, and differential lectin staining in E18.5 mouse yolk sacs. a Left: Representative immunofluorescence images of yolk sac sections from control and 0.5 ppm O_3-exposed groups at E18.5, stained for FcRn (red), IgG (orange), and FITC-conjugated GHA lectin (yellow), with DAPI counterstain (cyan), scale bar, 50 μm. Right: Quantification of the FcRn & IgG Manders' colocalization coefficient and the fluorescence intensity of GHA (6 random

fields per sample). Top panels show female fetal yolk sacs; bottom panels show male fetal yolk sacs (n = 3 mice/group). **b** Left: Representative images of yolk sac sections from control and 1 ppm O_3-exposed groups at E18.5, stained for FcRn (red), IgG (orange), and FITC-conjugated TL lectin (yellow), with DAPI (cyan), scale bar, 50 μm. Right: Quantification of FcRn & IgG Manders' colocalization coefficient and TL fluorescence intensity (6 random fields per sample). Top: female; bottom: male (n = 3 mice/group). Data are mean ± SD, analyzed by two-tailed Student's t-test. *$p < 0.05$ and **$p < 0.01$ compared with the corresponding control group.

Supplementary Fig. 15

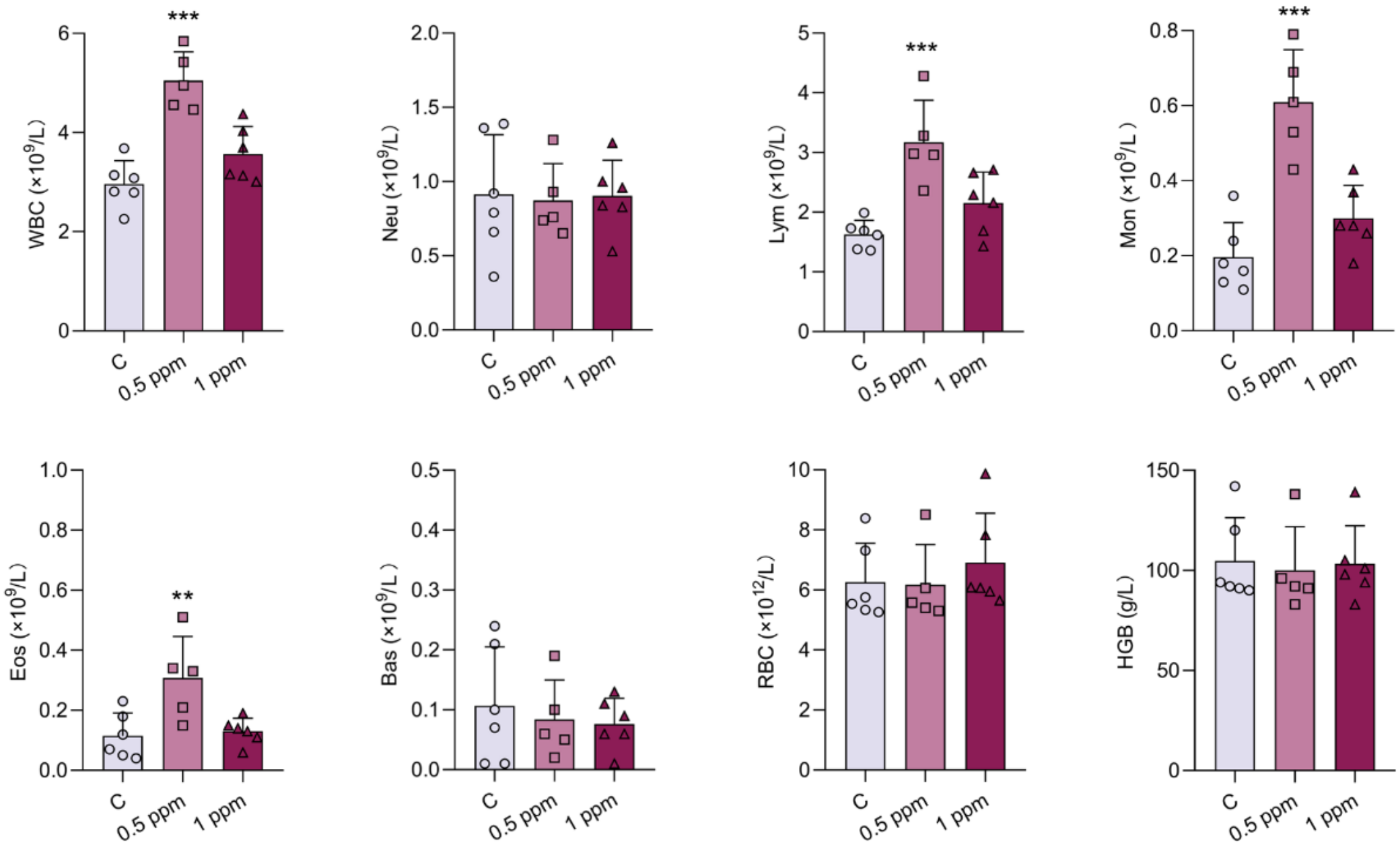

Figure S15. CBC analysis in female MIA offspring at PND14. Quantitative measurements of CBC parameters (n = 6, 5, 6; ordered by control, 0.5 ppm, and 1 ppm groups). Data are mean ± SD, analyzed by one-way ANOVA or Kruskal-Wallis test. **$p < 0.01$, ***$p < 0.001$ compared with the corresponding control group.

Supplementary Fig. 16

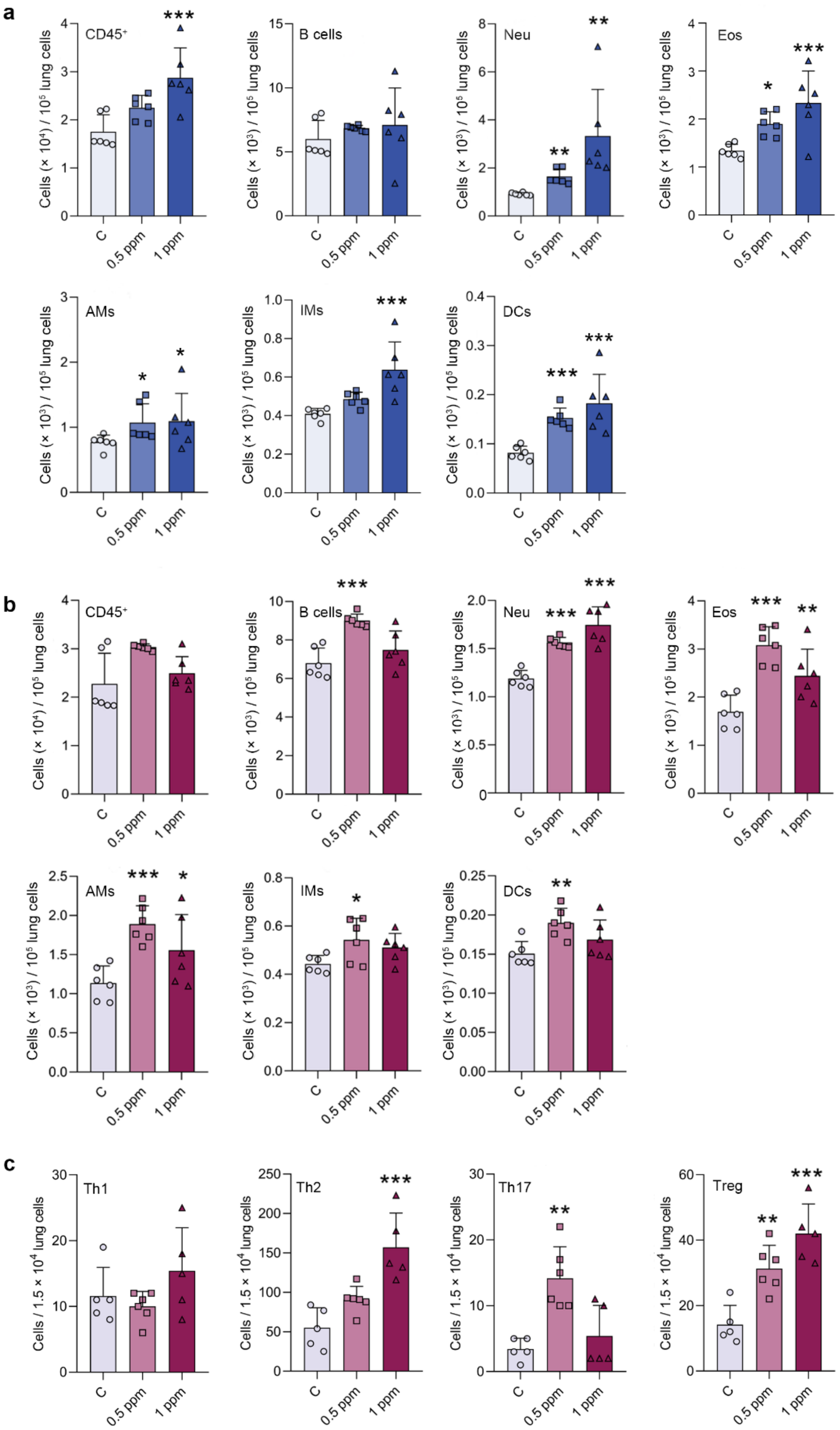

Figure S16. MIA-driven pulmonary immune cell alterations in offspring at PND14.

a, b Quantitative analysis of lung inflammatory cell subsets in male **(a)** and female (**b**) offspring at PND14 (n = 6 mice/group). **c** Quantitative analysis of lung T-cell subsets in female offspring at PND14 (n = 5, 6, 5; ordered by control, 0.5 ppm, and 1 ppm groups). Data are mean ± SD, analyzed by one-way ANOVA or Kruskal-Wallis test. *$p < 0.05$, **$p < 0.01$, and ***$p < 0.001$ compared with the corresponding control group.

Supplementary Fig. 17

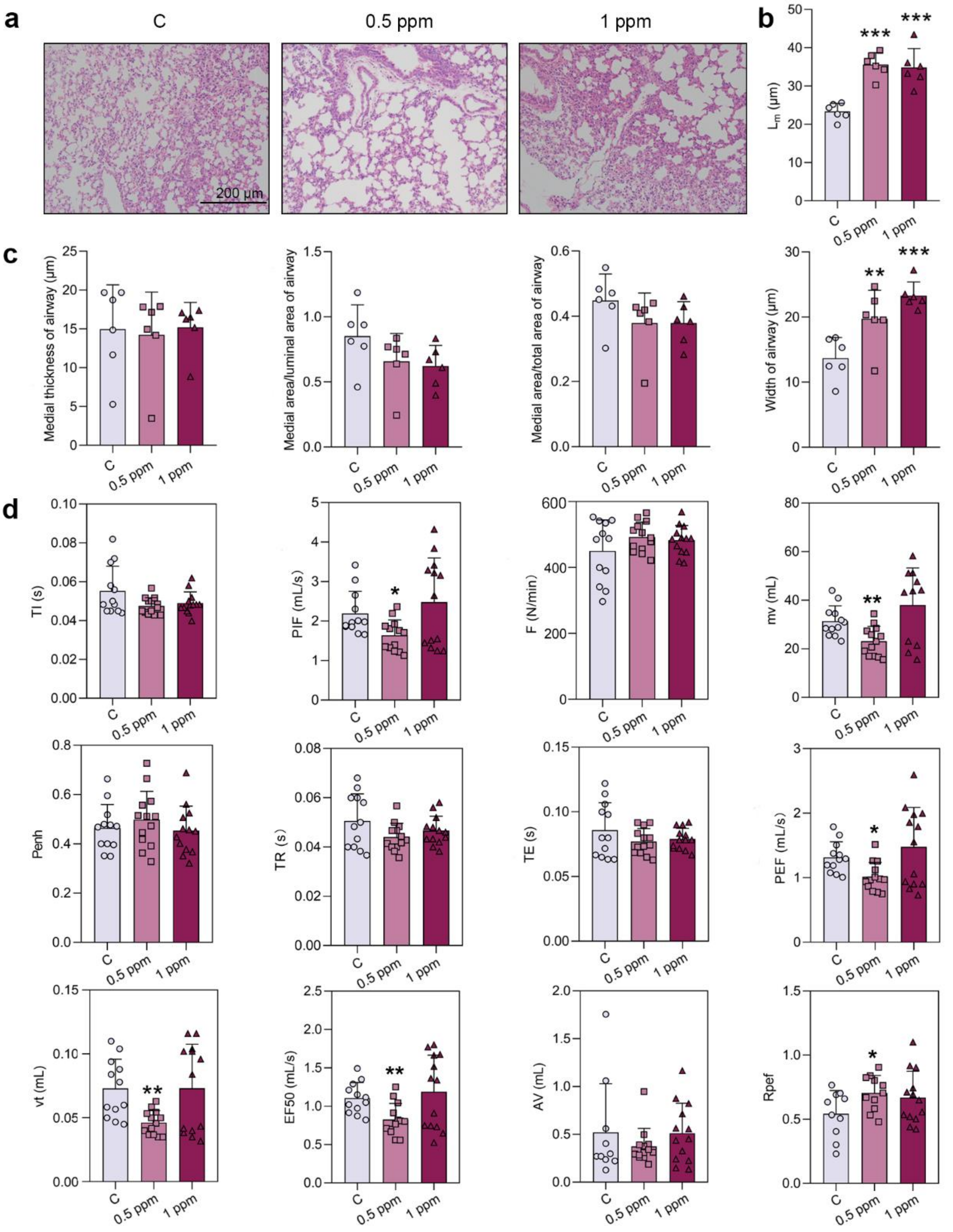

Figure S17. MIA-driven pulmonary pathophysiological alterations in female offspring at PND14. a, b Representative H&E-stained lung sections **(a)** and L_m analysis **(b)** of female offspring lungs, scale bar, 200 μm (n = 6 mice/group). **c**

Quantitative analysis of airway remodeling metrics (n = 6 mice/group). **d** Pulmonary functional parameters: inspiratory time (TI), peak inspiratory flow (PIF), respiratory frequency (F), minute ventilation (mv), enhanced pause (Penh), total respiratory cycle time (TR), expiratory time (TE), peak expiratory flow (PEF), tidal volume (vt), expiratory flow at 50% tidal volume (EF50), alveolar ventilation (AV), and ratio of peak expiratory flow (Rpef) (n = 10-13 mice/group). Data are mean ± SD, analyzed by one-way ANOVA or Kruskal-Wallis test. *$p < 0.05$, **$p < 0.01$, and ***$p < 0.001$ compared with the corresponding control group.

Supplementary Fig. 18

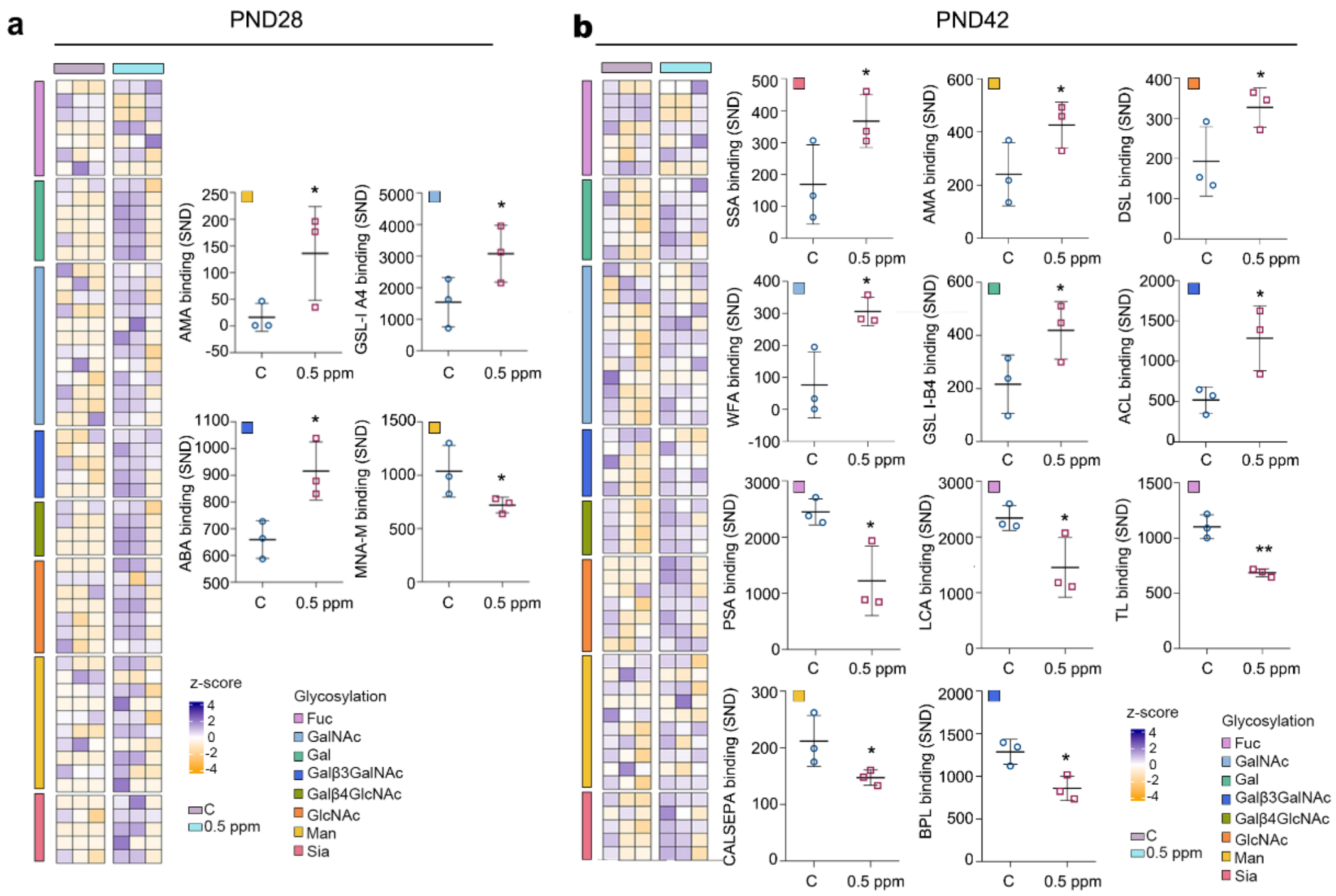

Figure S18. IgG glycosylation profiles and differential lectin binding in male offspring at PND28 and PND42. a, b Serum IgG glycosylation profiles and significantly altered lectin binding patterns in male offspring at PND28 **(a)** and PND42 (**b**), comparing those born to dams exposed to 0.5 ppm O_3 during pregnancy with offspring from control dams (n = 3 mice/group). Data are mean ± SD, analyzed by one-tailed Student's t-test. *$p < 0.05$ and **$p < 0.01$ compared with the corresponding control group.

Supplementary Fig. 19

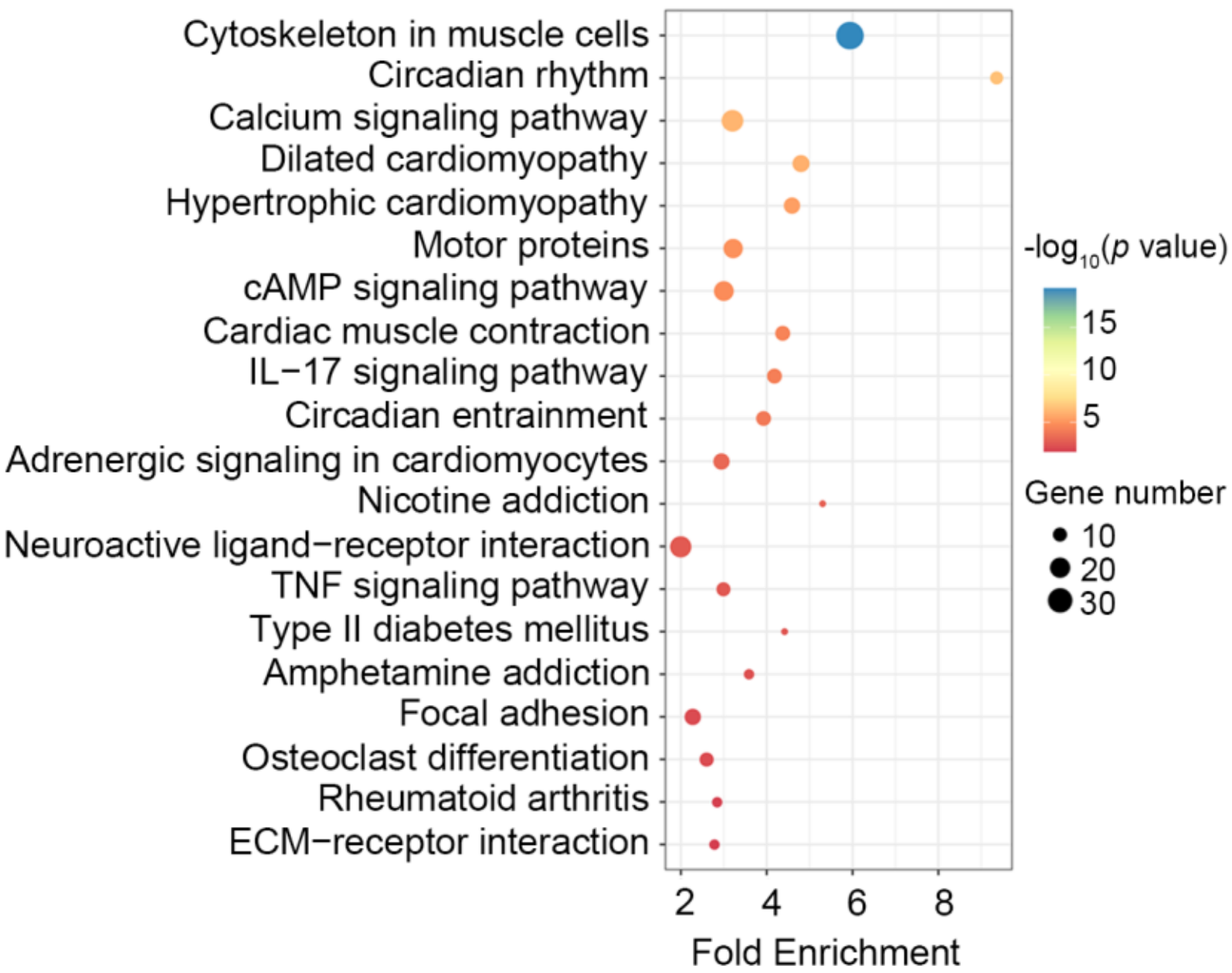

Figure S19. Top 20 KEGG pathways enriched in DEGs from male offspring lungs at PND56.

Supplementary Fig. 20

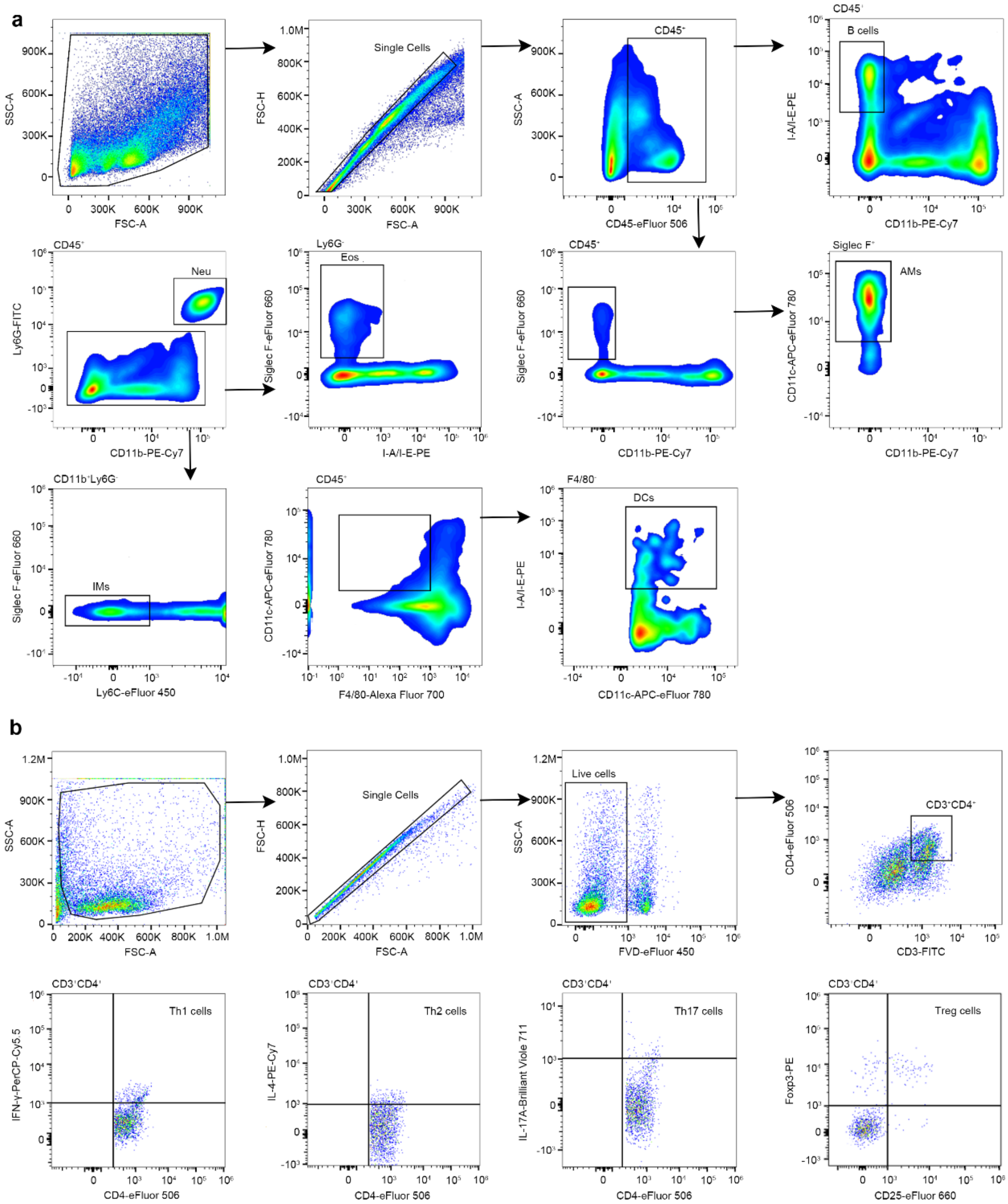

Figure S20. Gating strategy for the identification of inflammatory cell and T cell subsets. a Gating strategy for identifying B cells, neutrophils (Neu), eosinophils (Eos), alveolar macrophages (AMs), interstitial macrophages (IMs), and dendritic cells (DCs). **b** Gating strategy for T-cell subsets (Th1, Th2, Th17, and Treg). Live single cells were

sequentially gated using forward scatter (FSC) and side scatter (SSC), followed by doublet exclusion.

Supplementary Fig. 21

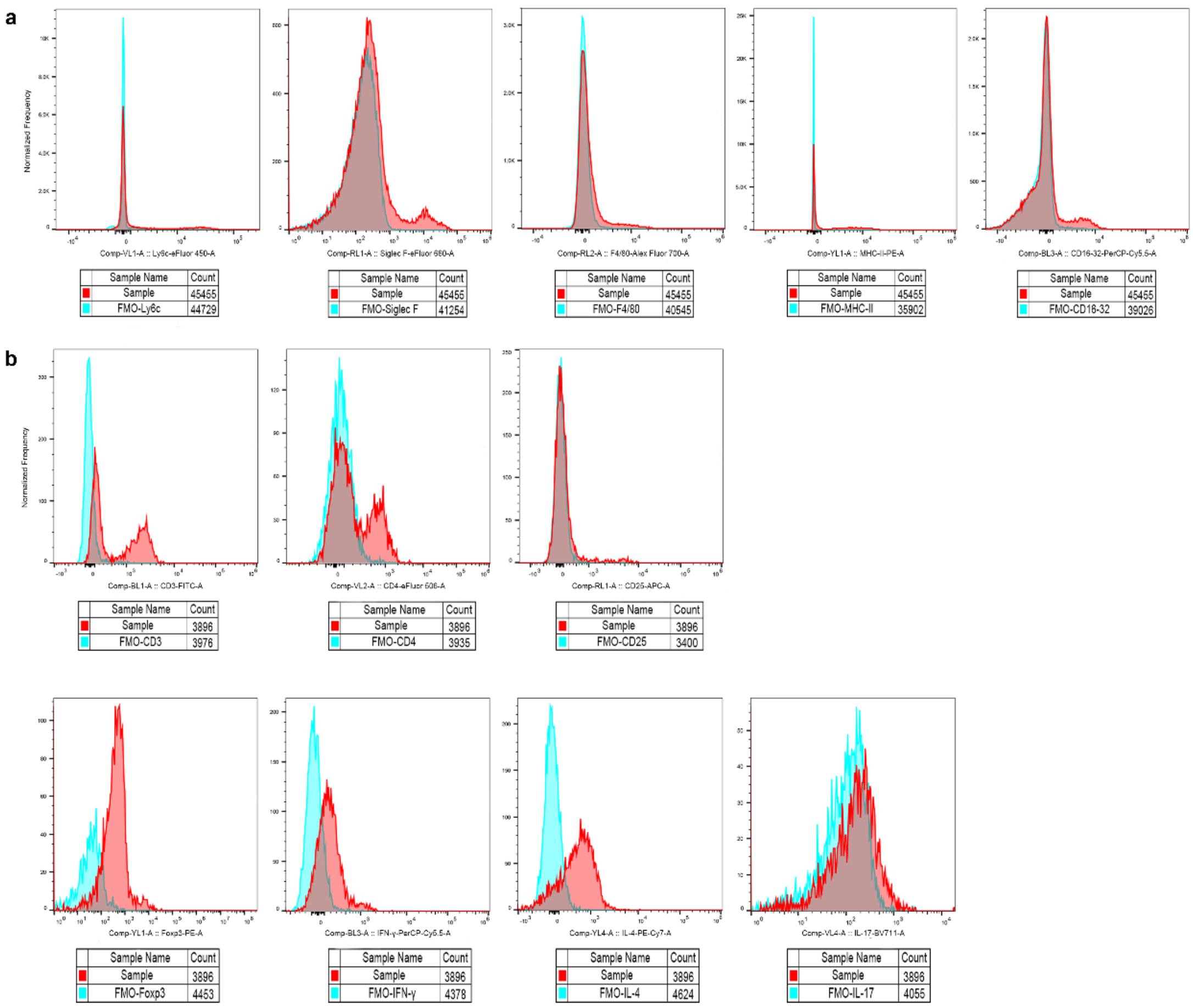

Figure S21. Representative fluorescence-minus-one (FMO) control histograms for flow cytometry gating. a Histograms for inflammatory cell markers: Ly6c, Siglec-F, F4/80, MHC-II, and CD16/32. **b** Histograms for T cell markers: CD3, CD4, CD25, IFN-γ, Foxp3, IL-4, and IL-17.

SUPPLEMENTARY TABLES

Supplementary Table 1. Characteristics of the participants in the study (n = 5,299).

Covariates	WBC（10^9/L）			Neu（10^9/L）			Lym（10^9/L）			Mon（10^9/L）		
	Normal（n = 3267）	Abnormal（n = 2032)	*p*	Normal（n = 2930）	Abnormal（n = 2369)	*p*	Normal（n = 4604）	Abnormal（n = 695)	*p*	Normal（n = 3555）	Abnormal（n = 1744)	*p*
**Maternal age (years)**			0.72			0.05			0.05			0.72
< 28	778	493		738	533		1098	173		841	430	
28-35	2187	1364		1941	1610		3107	444		2394	1157	
> 35	302	175		251	226		399	78		320	157	
**Pre-pregnancy BMI (kg/m²)**			1.00			0.41			0.58			0.84
< 18.5	487	303		443	347		680	110		537	253	
18.5-24	2167	1347		1921	1593		3051	463		2350	1164	
> 24	613	382		566	429		873	122		668	327	
**University degree**			0.22			0.12			0.54			0.63
Yes	2648	1618		2336	1930		3713	553		2869	1397	
No	619	414		594	439		891	142		686	347	
**Employment**			0.89			0.71			0.67			0.84
Yes	2022	1253		1818	1457		2851	424		2201	1074	
No	1245	779		1112	912		1753	271		1354	670	
**Family income per person (yuan/month)**			0.40			0.35			0.91			0.77
≤ 4000	1621	1033		1485	1169		2304	350		1775	879	

> 4000	1646	999		1445	1200		2300	345		1780	865	
**Folic acid supplementation**			0.79			0.64			0.14			0.49
Yes	3106	1936		2792	2250		4389	653		3377	1665	
No	161	96		138	119		215	42		178	79	
**Smoking**			0.56			1.00			0.34			1.00
Yes	1	2		2	1		2	1		2	1	
No	3266	2030		2928	2368		4602	694		3553	1743	
**Drinking**			0.38			1.00			0.51			1.00
Yes	2	3		3	2		4	1		4	1	
No	3265	2029		2927	2367		4600	694		3551	1743	
**Gravidity**			0.06			0.34			0.18			0.81
≤ 1	1590	1052		1484	1158		2274	368		1764	878	
2	1084	615		916	783		1495	204		1150	549	
> 3	593	365		530	428		835	123		641	317	
**Parity**			0.03			0.49			0.16			0.52
≤ 1	2086	1365		1927	1524		2976	475		2297	1154	
2	1096	626		932	790		1517	205		1171	551	
> 3	85	41		71	55		111	15		87	39	
**Previous adverse pregnancy**			0.99			0.42			0.92			0.71
Yes	254	159		220	193		360	53		281	132	
No	3013	1873		2710	2176		4244	642		3274	1612	

377

Covariates	Eos（10^9/L）			Bas（10^9/L）			FIB（g/L）		
	Normal（n = 5287）	Abnormal（n = 12)	*p*	Normal（n = 5257）	Abnormal（n = 42)	*p*	Normal（n = 4998）	Abnormal（n = 301)	*p*
**Maternal age (years)**			0.52			0.51			0.13
< 28	1269	2		1263	8		1103	168	
28-35	3543	8		3519	32		3145	406	
> 35	475	2		475	2		412	65	
**Pre-pregnancy BMI (kg/m²)**			0.02			0.47			0.54
< 18.5	785	5		781	9		704	86	
18.5-24	3510	4		3489	25		3082	432	
> 24	992	3		987	8		874	121	
**University degree**			0.48			0.79			1.00
Yes	4255	11		4231	35		3752	514	
No	1032	1		1026	7		908	125	
**Employment**			0.39			1.00			0.22
Yes	3269	6		3249	26		2878	397	
No	2018	6		2008	16		1782	242	
**Family income per person (yuan/month)**			1.00			0.27			0.61
≤ 4000	2648	6		2637	17		2349	305	
> 4000	2639	6		2620	25		2311	334	
**Folic acid supplementation**			0.45			0.72			0.77
Yes	5031	11		5001	41		4436	606	

No	256	1		256	1		224	33	
**Smoking**			1.00			1.00			1.00
Yes	3	0		3	0		3	0	
No	5284	12		5254	42		4657	639	
**Drinking**			1.00			1.00			1.00
Yes	5	0		5	0		5	0	
No	5282	12		5252	42		4655	639	
**Gravidity**			0.86			0.32			0.30
≤ 1	2635	7		2620	22		2321	321	
2	1695	4		1683	16		1508	191	
> 3	957	1		954	4		831	127	
**Parity**			0.52			0.77			0.18
≤ 1	3441	10		3422	29		3024	427	
2	1720	2		1710	12		1530	192	
> 3	126	0		125	1		106	20	
**Previous adverse pregnancy**			0.62			1.00			0.91
Yes	413	0		410	3		362	51	
No	4874	12		4847	39		4298	588	

Statistical associations between categorical variables and hematological abnormalities were analyzed using two-sided χ^2 tests (or Fisher's exact test for small-frequency cases). Statistical significance is defined as $p < 0.05$.

Supplementary Table 2. Trimester-specific associations between ambient $PM_{2.5}$ exposure and increased risks of hematological parameter abnormalities.

	OR	95% CI	*p*		OR	95% CI	*p*
**WBC**							
Pre-T1	0.974	0.943 to 1.007	0.121	T3	0.982	0.952 to 1.012	0.237
T1	1.010	0.977 to 1.044	0.557	T2-T3	0.996	0.953 to 1.042	0.874
T2	1.016	0.984 to 1.049	0.317	T1-T3	1.010	0.939 to 1.086	0.795
**Neu**							
Pre-T1	1.020	0.988 to 1.054	0.219	T3	1.007	0.977 to 1.038	0.647
T1	1.004	0.972 to 1.037	0.806	T2-T3	0.996	0.954 to 1.040	0.861
T2	0.988	0.958 to 1.020	0.467	T1-T3	1.000	0.931 to 1.073	0.991
**Lym**							
Pre-T1	1.009	0.962 to 1.058	0.702	T3	1.023	0.979 to 1.069	0.310
T1	0.984	0.938 to 1.032	0.501	T2-T3	1.030	0.966 to 1.099	0.360
T2	1.006	0.961 to 1.053	0.795	T1-T3	1.027	0.925 to 1.142	0.614
**Mon**							
Pre-T1	0.996	0.963 to 1.031	0.826	T3	0.984	0.953 to 1.016	0.318
T1	1.012	0.978 to 1.047	0.488	T2-T3	0.974	0.930 to 1.020	0.263
T2	0.990	0.958 to 1.024	0.566	T1-T3	0.973	0.902 to 1.049	0.475
**Eos**							
Pre-T1	0.977	0.685 to 1.358	0.891	T3	1.029	0.747 to 1.395	0.855
T1	0.796	0.518 to 1.139	0.247	T2-T3	1.237	0.782 to 2.020	0.373
T2	1.194	0.864 to 1.661	0.279	T1-T3	1.049	0.496 to 2.274	0.902
**Bas**							
Pre-T1	1.092	0.915 to 1.299	0.324	T3	0.898	0.747 to 1.066	0.234
T1	0.854	0.699 to 1.028	0.106	T2-T3	0.821	0.640 to 1.046	0.114
T2	0.908	0.753 to 1.083	0.293	T1-T3	0.561	0.381 to 0.827	0.003
**FIB**							
Pre-T1	1.033	0.983 to 1.084	0.195	T3	1.039	0.993 to 1.088	0.097
T1	1.088	1.037 to 1.142	0.001***	T2-T3	1.125	1.053 to 1.203	0.001***
T2	1.082	1.032 to 1.134	0.001***	T1-T3	1.434	1.283 to 1.605	< 0.001***

Adjusted odds ratios (ORs) with 95% confidence intervals (CIs) were calculated for abnormalities in hematological parameters associated with each 10 μg/m^3 increase in ambient $PM_{2.5}$ exposure, with covariate adjustments for maternal age, pre-pregnancy body mass index (BMI), education level, occupation, family income per person, folic acid supplementation, smoking, drinking, gravidity, parity, and previous adverse

pregnancy.

Pregnancy stage definitions: Pre-T1: 12 weeks before pregnancy; T1: First trimester (weeks 1-12); T2: Second trimester (weeks 13-27); T3: Third trimester (week 28 until delivery); T2-T3: Mid-to-late pregnancy period (weeks 13 until delivery); T1-T3: Entire pregnancy period.

p values are derived from two-sided Wald tests, and * indicates statistically significant risk factors (OR > 1, ***$p < 0.001$).

Supplementary Table 3. Trimester-specific associations between ambient PM_{10} exposure and increased risks of hematological parameter abnormalities.

	OR	95% CI	*p*		OR	95% CI	*p*
**WBC**							
Pre-T1	1.002	0.984 to 1.019	0.865	T3	1.012	0.995 to 1.028	0.160
T1	1.000	0.983 to 1.018	0.990	T2-T3	1.016	0.995 to 1.037	0.135
T2	1.008	0.992 to 1.025	0.330	T1-T3	1.017	0.991 to 1.044	0.212
**Neu**							
Pre-T1	0.997	0.980to 1.014	0.704	T3	0.978	0.963 to 0.994	0.007
T1	1.005	0.988 to 1.022	0.574	T2-T3	0.979	0.959 to 0.999	0.037
T2	0.995	0.979 to 1.011	0.544	T1-T3	0.981	0.956 to 1.006	0.140
**Lym**							
Pre-T1	1.000	0.975 to 1.025	0.985	T3	0.997	0.974 to 1.021	0.791
T1	0.983	0.959 to 1.008	0.191	T2-T3	0.979	0.950 to 1.008	0.158
T2	0.976	0.953 to 0.999	0.046	T1-T3	0.965	0.930 to 1.002	0.067
**Mon**							
Pre-T1	0.985	0.967 to 1.003	0.092	T3	0.989	0.973 to 1.006	0.208
T1	1.005	0.987 to 1.024	0.572	T2-T3	0.997	0.975 to 1.018	0.753
T2	1.007	0.990 to 1.024	0.473	T1-T3	1.000	0.974 to 1.027	0.987
**Eos**							
Pre-T1	0.942	0.785 to 1.126	0.513	T3	0.995	0.836 to 1.176	0.952
T1	0.880	0.719 to 1.057	0.188	T2-T3	0.991	0.797 to 1.225	0.933
T2	0.994	0.835 to 1.178	0.942	T1-T3	0.905	0.688 to 1.181	0.468
**Bas**							
Pre-T1	0.975	0.886 to 1.073	0.596	T3	0.937	0.852 to 1.026	0.166
T1	0.951	0.861 to 1.047	0.309	T2-T3	0.966	0.861 to 1.081	0.546
T2	1.021	0.932 to 1.119	0.657	T1-T3	0.929	0.804 to 1.071	0.312
**FIB**							
Pre-T1	1.019	0.993 to 1.046	0.158	T3	1.074	1.049 to 1.101	< 0.001***
T1	1.078	1.050 to 1.107	< 0.001***	T2-T3	1.154	1.118 to 1.191	< 0.001***
T2	1.118	1.090 to 1.147	< 0.001***	T1-T3	1.234	1.186 to 1.285	< 0.001***

Adjusted odds ratios (ORs) with 95% confidence intervals (CIs) were calculated for abnormalities in hematological parameters associated with each 10 μg/m^3 increase in ambient PM_{10} exposure, with covariate adjustments for maternal age, pre-pregnancy body mass index (BMI), education level, occupation, family income per person, folic acid supplementation, smoking, drinking, gravidity, parity, and previous adverse

pregnancy.

Pregnancy stage definitions: Pre-T1: 12 weeks before pregnancy; T1: First trimester (weeks 1-12); T2: Second trimester (weeks 13-27); T3: Third trimester (week 28 until delivery); T2-T3: Mid-to-late pregnancy period (weeks 13 until delivery); T1-T3: Entire pregnancy period.

p values are derived from two-sided Wald tests, and * indicates statistically significant risk factors (OR > 1, ***$p < 0.001$).

Supplementary Table 4. Trimester-specific associations between ambient NO_2 exposure and increased risks of hematological parameter abnormalities.

	OR	95% CI	*p*		OR	95% CI	*p*
**WBC**							
Pre-T1	1.016	0.957 to 1.078	0.613	T3	1.017	0.959 to 1.078	0.575
T1	0.980	0.924 to 1.040	0.511	T2-T3	0.998	0.927 to 1.075	0.966
T2	0.981	0.926 to 1.041	0.530	T1-T3	0.983	0.898 to 1.076	0.711
**Neu**							
Pre-T1	0.995	0.938 to 1.055	0.857	T3	0.928	0.877 to 0.983	0.011
T1	1.017	0.960 to 1.077	0.576	T2-T3	0.935	0.870 to 1.005	0.069
T2	0.990	0.935 to 1.049	0.741	T1-T3	0.948	0.868 to 1.035	0.232
**Lym**							
Pre-T1	1.006	0.923 to 1.097	0.897	T3	1.030	0.947 to 1.121	0.488
T1	0.937	0.861 to 1.020	0.133	T2-T3	0.951	0.857 to 1.058	0.355
T2	0.912	0.839 to 0.992	0.031	T1-T3	0.907	0.801 to 1.031	0.129
**Mon**							
Pre-T1	0.991	0.932 to 1.055	0.781	T3	0.939	0.884 to 0.997	0.040
T1	1.080	1.016 to 1.148	0.014*	T2-T3	0.944	0.875 to 1.019	0.140
T2	0.991	0.933 to 1.053	0.781	T1-T3	1.002	0.913 to 1.101	0.960
**Eos**							
Pre-T1	0.744	0.419 to 1.361	0.323	T3	1.154	0.635 to 2.176	0.647
T1	1.080	0.599 to 2.014	0.803	T2-T3	1.604	0.719 to 4.049	0.286
T2	1.494	0.804 to 2.949	0.223	T1-T3	1.775	0.656 to 6.105	0.321
**Bas**							
Pre-T1	0.989	0.719 to 1.379	0.948	T3	0.932	0.681 to 1.286	0.666
T1	0.956	0.697 to 1.324	0.784	T2-T3	1.077	0.724 to 1.651	0.725
T2	1.017	0.984 to 1.051	0.325	T1-T3	1.038	0.653 to 1.775	0.884
**FIB**							
Pre-T1	1.029	0.941 to 1.126	0.529	T3	1.068	0.978 to 1.166	0.142
T1	1.184	1.083 to 1.296	< 0.001***	T2-T3	1.269	1.130 to 1.427	< 0.001***
T2	1.250	1.143 to 1.369	< 0.001***	T1-T3	1.499	1.288 to 1.755	< 0.001***

Adjusted odds ratios (ORs) with 95% confidence intervals (CIs) were calculated for abnormalities in hematological parameters associated with each 10 $\mu g/m^3$ increase in ambient NO_2 exposure, with covariate adjustments for maternal age, pre-pregnancy body mass index (BMI), education level, occupation, family income per person, folic acid supplementation, smoking, drinking, gravidity, parity, and previous adverse

pregnancy.

Pregnancy stage definitions: Pre-T1: 12 weeks before pregnancy; T1: First trimester (weeks 1-12); T2: Second trimester (weeks 13-27); T3: Third trimester (week 28 until delivery); T2-T3: Mid-to-late pregnancy period (weeks 13 until delivery); T1-T3: Entire pregnancy period.

p values are derived from two-sided Wald tests, and * indicates statistically significant risk factors ($OR > 1$, *$p < 0.05$, ***$p < 0.001$).

Supplementary Table 5. Trimester-specific associations between ambient SO_2 exposure and increased risks of hematological parameter abnormalities.

SO_2	OR	95% CI	*p*		OR	95% CI	*p*
**WBC**							
Pre-T1	1.014	0.988 to 1.040	0.289	T3	1.002	0.999 to 1.005	0.151
T1	0.995	0.966 to 1.025	0.745	T2-T3	1.024	0.985 to 1.065	0.223
T2	1.007	0.978 to 1.036	0.647	T1-T3	1.022	0.972 to 1.074	0.393
**Neu**							
Pre-T1	0.978	0.954 to 1.003	0.082	T3	0.996	0.993 to 0.999	0.012
T1	1.018	0.989 to 1.047	0.226	T2-T3	0.951	0.915 to 0.988	0.011
T2	0.979	0.952 to 1.008	0.151	T1-T3	0.963	0.917 to 1.012	0.136
**Lym**							
Pre-T1	1.009	0.973 to 1.046	0.619	T3	1.000	0.995 to 1.004	0.990
T1	0.960	0.919 to 1.002	0.067	T2-T3	0.972	0.917 to 1.029	0.335
T2	0.970	0.929 to 1.012	0.159	T1-T3	0.931	0.864 to 1.003	0.061
**Mon**							
Pre-T1	0.947	0.922 to 0.973	< 0.001	T3	0.996	0.993 to 0.999	0.015
T1	1.001	0.971 to 1.031	0.953	T2-T3	0.980	0.941 to 1.021	0.341
T2	1.013	0.983 to 1.044	0.391	T1-T3	0.980	0.930 to 1.032	0.436
**Eos**							
Pre-T1	0.958	0.690 to 1.227	0.766	T3	1.009	0.976 to 1.037	0.567
T1	0.826	0.523 to 1.155	0.335	T2-T3	1.049	0.675 to 1.506	0.813
T2	0.973	0.685 to 1.284	0.860	T1-T3	0.906	0.488 to 1.498	0.726
**Bas**							
Pre-T1	0.946	0.804 to 1.089	0.471	T3	1.004	0.987 to 1.020	0.619
T1	0.996	0.838 to 1.160	0.959	T2-T3	1.047	0.842 to 1.274	0.660
T2	1.015	0.861 to 1.176	0.848	T1-T3	1.049	0.791 to 1.356	0.728
**FIB**							
Pre-T1	1.020	0.983 to 1.058	0.285	T3	1.006	1.002 to 1.011	0.005**
T1	1.147	1.103 to 1.193	< 0.001***	T2-T3	1.241	1.177 to 1.308	< 0.001***
T2	1.199	1.154 to 1.246	< 0.001***	T1-T3	1.446	1.352 to 1.547	< 0.001***

Adjusted odds ratios (ORs) with 95% confidence intervals (CIs) were calculated for abnormalities in hematological parameters associated with each 10 μg/m^3 increase in ambient SO_2 exposure, with covariate adjustments for maternal age, pre-pregnancy body mass index (BMI), education level, occupation, family income per person, folic acid supplementation, smoking, drinking, gravidity, parity, and previous adverse

pregnancy.

Pregnancy stage definitions: Pre-T1: 12 weeks before pregnancy; T1: First trimester (weeks 1-12); T2: Second trimester (weeks 13-27); T3: Third trimester (week 28 until delivery); T2-T3: Mid-to-late pregnancy period (weeks 13 until delivery); T1-T3: Entire pregnancy period.

p values are derived from two-sided Wald tests, and * indicates statistically significant risk factors (OR > 1, **$p < 0.01$, ***$p < 0.001$).

Supplementary Table 6. Associations of trimester-specific O_3 exposure with risks of hematological parameter abnormalities: Single-pollutant and $PM_{2.5}$ adjusted models.

	Single-pollutant model			$PM_{2.5}$-adjusted model		
	OR	95% CI	*p*	OR	95% CI	*p*
**T3**						
WBC	0.990	0.978 to 1.003	0.136	0.987	0.974 to 1.001	0.064
Neu	1.016	1.004 to 1.029	0.012*	1.018	1.005 to 1.032	0.006**
Lym	0.986	0.967 to 1.004	0.127	0.987	0.968 to 1.006	0.190
Mon	1.022	1.008 to 1.035	0.002**	1.021	1.007 to 1.035	0.003**
Eos	0.979	0.853 to 1.120	0.761	0.981	0.850 to 1.127	0.791
Bas	1.005	0.936 to 1.079	0.886	0.994	0.924 to 1.070	0.873
FIB	1.030	1.010 to 1.050	0.003**	1.036	1.016 to 1.057	< 0.001***
**T2-T3**						
WBC	0.993	0.974 to 1.012	0.474	0.992	0.973 to 1.012	0.446
Neu	1.020	1.001 to 1.039	0.044*	1.020	1.001 to 1.040	0.044*
Lym	0.993	0.965 to 1.021	0.607	0.995	0.967 to 1.024	0.732
Mon	1.021	1.000 to 1.041	0.048*	1.019	0.998 to 1.040	0.073
Eos	0.859	0.678 to 1.058	0.172	0.871	0.685 to 1.077	0.222
Bas	0.976	0.876 to 1.086	0.652	0.957	0.858 to 1.035	0.430
FIB	0.990	0.961 to 1.019	0.498	1.000	0.971 to 1.030	0.995

Adjusted odds ratios (ORs) with 95% confidence intervals (CIs) were calculated for abnormalities in hematological parameters associated with each 10 μg/m^3 increase in ambient O_3 exposure. Analyses were performed using single-pollutant models and models additionally adjusted for $PM_{2.5}$ exposure, with covariate adjustments for maternal age, pre-pregnancy body mass index (BMI), education level, occupation,

family income per person, folic acid supplementation, smoking, drinking, gravidity, parity, and previous adverse pregnancy.

Pregnancy stage definitions: T3: Third trimester (week 28 until delivery); T2-T3: Mid-to-late pregnancy period (weeks 13 until delivery).

p values are derived from two-sided Wald tests, and * indicates statistically significant risk factors ($OR > 1$, *$p < 0.05$, **$p < 0.01$, ***$p < 0.001$).

Supplementary Table 7. Associations of trimester-specific O_3 exposure with risks of hematological parameter abnormalities: Single-pollutant and NO_2 adjusted models.

	Single-pollutant model			NO_2-adjusted model		
	OR	95% CI	*p*	OR	95% CI	*p*
**T3**						
WBC	0.990	0.978 to 1.003	0.136	0.985	0.966 to 1.004	0.111
Neu	1.016	1.004 to 1.029	0.012*	1.009	0.991 to 1.028	0.335
Lym	0.986	0.967 to 1.004	0.127	0.979	0.952 to 1.006	0.132
Mon	1.022	1.008 to 1.035	0.002**	1.025	1.005 to 1.045	0.016*
Eos	0.979	0.853 to 1.120	0.761	1.005	0.823 to 1.225	0.958
Bas	1.005	0.936 to 1.079	0.886	0.987	0.891 to 1.095	0.805
FIB	1.030	1.010 to 1.050	0.003**	1.094	1.063 to 1.127	< 0.001***
**T2-T3**						
WBC	0.993	0.974 to 1.012	0.474	0.987	0.961 to 1.013	0.317
Neu	1.020	1.001 to 1.039	0.044*	1.014	0.988 to 1.041	0.283
Lym	0.993	0.965 to 1.021	0.607	0.972	0.936 to 1.009	0.134
Mon	1.021	1.000 to 1.041	0.048*	1.019	0.991 to 1.047	0.182
Eos	0.859	0.678 to 1.058	0.172	0.875	0.650 to 1.176	0.370
Bas	0.976	0.876 to 1.086	0.652	0.979	0.849 to 1.136	0.773
FIB	0.990	0.961 to 1.019	0.498	1.067	1.023 to 1.114	0.003**

Adjusted odds ratios (ORs) with 95% confidence intervals (CIs) were calculated for abnormalities in hematological parameters associated with each 10 μg/m^3 increase in ambient O_3 exposure. Analyses were performed using single-pollutant models and models additionally adjusted for NO_2 exposure, with covariate adjustments for maternal age, pre-pregnancy body mass index (BMI), education level, occupation, family income

per person, folic acid supplementation, smoking, drinking, gravidity, parity, and previous adverse pregnancy.

Pregnancy stage definitions: T3: Third trimester (week 28 until delivery); T2-T3: Mid-to-late pregnancy period (weeks 13 until delivery).

p values are derived from two-sided Wald tests, and * indicates statistically significant risk factors (OR > 1, $^{*}p < 0.05$, $^{**}p < 0.01$, $^{***}p < 0.001$).

Supplementary Table 8. Associations of trimester-specific O_3 exposure with risks of hematological parameter abnormalities: Single-pollutant and SO_2 adjusted models.

	Single-pollutant model			SO_2-adjusted model		
	OR	95% CI	*p*	OR	95% CI	*p*
**T3**						
WBC	0.990	0.978 to 1.003	0.136	0.993	0.976 to 1.012	0.491
Neu	1.016	1.004 to 1.029	0.012*	1.010	0.992 to 1.028	0.280
Lym	0.986	0.967 to 1.004	0.127	0.972	0.947 to 0.997	0.033
Mon	1.022	1.008 to 1.035	0.002**	1.020	1.000 to 1.038	0.043*
Eos	0.979	0.853 to 1.120	0.761	1.010	0.837 to 1.214	0.916
Bas	1.005	0.936 to 1.079	0.886	1.033	0.937 to 1.140	0.508
FIB	1.030	1.010 to 1.050	0.003**	1.095	1.066 to 1.125	< 0.001***
**T2-T3**						
WBC	0.993	0.974 to 1.012	0.474	0.999	0.976 to 1.023	0.943
Neu	1.020	1.001 to 1.039	0.044*	1.009	0.986 to 1.032	0.453
Lym	0.993	0.965 to 1.021	0.607	0.979	0.947 to 1.012	0.218
Mon	1.021	1.000 to 1.041	0.048*	1.022	0.997 to 1.046	0.082
Eos	0.859	0.678 to 1.058	0.172	0.826	0.631 to 1.055	0.140
Bas	0.976	0.876 to 1.086	0.652	0.983	0.866 to 1.118	0.797
FIB	0.990	0.961 to 1.019	0.498	1.076	1.039 to 1.115	< 0.001***

Adjusted odds ratios (ORs) with 95% confidence intervals (CIs) were calculated for abnormalities in hematological parameters associated with each 10 μg/m^3 increase in ambient O_3 exposure. Analyses were performed using single-pollutant models and models additionally adjusted for SO_2 exposure, with covariate adjustments for maternal age, pre-pregnancy body mass index (BMI), education level, occupation, family income

per person, folic acid supplementation, smoking, drinking, gravidity, parity, and previous adverse pregnancy.

Pregnancy stage definitions: T3: Third trimester (week 28 until delivery); T2-T3: Mid-to-late pregnancy period (weeks 13 until delivery).

p values are derived from two-sided Wald tests, and * indicates statistically significant risk factors (OR > 1, *$p < 0.05$, **$p < 0.01$, ***$p < 0.001$).

Supplementary Table 9. Associations of trimester-specific O_3 exposure with risks of hematological parameter abnormalities: Single-pollutant and PM_{10} adjusted models.

	Single-pollutant model			PM_{10}-adjusted model		
	OR	95% CI	*p*	OR	95% CI	*p*
**T3**						
WBC	0.990	0.978 to 1.003	0.136	0.993	0.979 to 1.007	0.338
Neu	1.016	1.004 to 1.029	0.012*	1.010	0.996 to 1.025	0.146
Lym	0.986	0.967 to 1.004	0.127	0.980	0.960 to 1.001	0.065
Mon	1.022	1.008 to 1.035	0.002**	1.022	1.007 to 1.038	0.004**
Eos	0.979	0.853 to 1.120	0.761	0.972	0.830 to 1.127	0.710
Bas	1.005	0.936 to 1.079	0.886	0.976	0.920 to 1.059	0.566
FIB	1.030	1.010 to 1.050	0.003**	1.068	1.046 to 1.091	< 0.001***
**T2-T3**						
WBC	0.993	0.974 to 1.012	0.474	0.996	0.977 to 1.017	0.732
Neu	1.020	1.001 to 1.039	0.044*	1.016	0.996 to 1.036	0.127
Lym	0.993	0.965 to 1.021	0.607	0.987	0.958 to 1.016	0.365
Mon	1.021	1.000 to 1.041	0.048*	1.021	1.000 to 1.043	0.049*
Eos	0.859	0.678 to 1.058	0.172	0.848	0.668 to 1.052	0.149
Bas	0.976	0.876 to 1.086	0.652	0.965	0.863 to 1.078	0.528
FIB	0.990	0.961 to 1.019	0.498	1.028	0.997 to 1.060	0.082

Adjusted odds ratios (ORs) with 95% confidence intervals (CIs) were calculated for abnormalities in hematological parameters associated with each 10 μg/m^3 increase in ambient O_3 exposure. Analyses were performed using single-pollutant models and models additionally adjusted for PM_{10} exposure, with covariate adjustments for maternal age, pre-pregnancy body mass index (BMI), education level, occupation,

family income per person, folic acid supplementation, smoking, drinking, gravidity, parity, and previous adverse pregnancy.

Pregnancy stage definitions: T3: Third trimester (week 28 until delivery); T2-T3: Mid-to-late pregnancy period (weeks 13 until delivery).

p values are derived from two-sided Wald tests, and * indicates statistically significant risk factors (OR > 1, *$p < 0.05$, **$p < 0.01$, ***$p < 0.001$).

Supplementary Table 10. Associations of trimester-specific O_3 exposure with risks of hematological parameter abnormalities: Base model and HGB adjusted model.

	Base model			HGB-adjusted model		
	OR	95% CI	*p*	OR	95% CI	*p*
**T3**						
WBC	0.990	0.978 to 1.003	0.136	0.991	0.978 to 1.004	0.172
Neu	1.016	1.004 to 1.029	0.012*	1.015	1.003 to 1.028	0.018*
Lym	0.986	0.967 to 1.004	0.127	0.985	0.966 to 1.003	0.101
Mon	1.022	1.008 to 1.035	0.002**	1.022	1.008 to 1.036	0.001**
Eos	0.979	0.853 to 1.120	0.761	0.981	0.855 to 1.121	0.781
Bas	1.005	0.936 to 1.079	0.886	1.002	0.933 to 1.076	0.953
FIB	1.030	1.010 to 1.050	0.003**	1.030	1.011 to 1.050	0.003**
**T2-T3**						
WBC	0.993	0.974 to 1.012	0.474	0.993	0.973 to 1.012	0.457
Neu	1.020	1.001 to 1.039	0.044*	1.020	1.001 to 1.040	0.040*
Lym	0.993	0.965 to 1.021	0.607	0.993	0.965 to 1.021	0.627
Mon	1.021	1.000 to 1.041	0.048*	1.021	1.000 to 1.041	0.049*
Eos	0.859	0.678 to 1.058	0.172	0.857	0.677 to 1.056	0.165
Bas	0.976	0.876 to 1.086	0.652	0.977	0.877 to 1.087	0.672
FIB	0.990	0.961 to 1.019	0.498	0.990	0.961 to 1.019	0.485

Adjusted odds ratios (ORs) with 95% confidence intervals (CIs) were calculated for abnormalities in hematological parameters associated with each 10 μg/m^3 increase in ambient O_3 exposure. Analyses were performed using base models and HGB adjusted model, with covariate adjustments for maternal age, pre-pregnancy body mass index (BMI), education level, occupation, family income per person, folic acid

supplementation, smoking, drinking, gravidity, parity, and previous adverse pregnancy.

Pregnancy stage definitions: T3: Third trimester (week 28 until delivery); T2-T3: Mid-to-late pregnancy period (weeks 13 until delivery).

p values are derived from two-sided Wald tests, and * indicates statistically significant risk factors (OR > 1, *$p < 0.05$, **$p < 0.01$).

Supplementary Table 11. Antibodies and dyes used in flow cytometry analysis.

Antibody	Catalog number	Company
CD45-eFluor506, clone 30-F11	69-0451-82	eBioscience
Ly6C-eFluor450, clone HK1.4	48-5932-82	eBioscience
Ly6G-FITC, clone 1A8	11-9668-82	eBioscience
I-A/I-E-PE, clone M5/114.15.2	12-5321-82	eBioscience
CD11b-PE-Cyanine7, clone M1/70	25-0112-82	eBioscience
Siglec F-eFluor660, clone 1RNM44N	50-1702-82	eBioscience
F4/80-Alexa Fluor700, clone BM8	56-4801-82	eBioscience
CD11c-APC-eFluor780, clone N418	47-0114-82	eBioscience
CD16/32-PerCP-Cyanine5.5, clone 93	101323	BioLegend
CD3-FITC, clone 17A2	11-0032-82	eBioscience
CD4-eFluor506, clone RM4-5	69-0042-82	eBioscience
CD25-eFluor660, clone eBio7D4	50-0252-82	eBioscience
IFN-γ-PerCP-Cyanine5.5, clone XMG1.2	45-7311-82	eBioscience
IL-4-PE-Cyanine7, clone 11B11	25-7041-82	eBioscience
IL-17A-Brilliant Violet711, clone eBio17B7	407-7177-82	eBioscience
Foxp3-PE, clone FJK-16s	12-5773-82	eBioscience
CD16/CD32 Monoclonal Antibody, clone 93	16-0161-82	eBioscience

Supplementary Table 12. Primers used for qPCR analysis.

Genes	Species	Forward primer	Reverse primer
C3	Mouse	TCGGCATAGAGAAGAGGCAAGAG	CGGTTGTTGAAGGCAGCATAGG
C3ar1	Mouse	GACACCGCTGATGGCAATAACC	ATTCTGAAGACGATGAGGCTGTAAC
C5	Mouse	CTGCCTGACTCACTAACGACTTG	TCCTCGCACAACAGAATATGGTATG
C5ar1	Mouse	ACATCTGCTACACCTTCCTCCTG	GACCACAGCCATCACCACTTTG
Cfb	Mouse	AGAGCCTTCCTGCCAAGATTCC	TCTTCCTCTTCTGCTGTTCTCCTG
Cfh	Mouse	ACGAACGCTCAATCTGTATCAATGG	TTGGAATCTGCGGTGGAGGAG
FcRn	Mouse	TGGAAGGAGCCGCCGTCTATG	GAAGGAGAAAGCAGCACAGGTCAG
Ccl20	Mouse	AGGCAGAAGCAAGCAACTACGAC	ATCGGCCATCTGTCTTGTGAAACC
Cxcl5	Mouse	ATCCCCAGCGGTTCCATCTCG	CGTTGCGGCTATGACTGAGGAAG
Cxcl1	Mouse	CTGGGATTCACCTCAAGAACATC	CAGGGTCAAGGCAAGCCTC
Cxcl2	Mouse	GACAGAAGTCATAGCCACTCTCAAG	TCAGTTAGCCTTGCCTTTGTTCAG
Traf3	Mouse	ATGCTCCGAAACAACGAGTCCAAG	GCTCTTCATGCTGTCCGCTTCC
Mapk10	Mouse	GGAGGTAATGAACTCAGAAGAGAAGAC	CAGACGAGGATGGAGGGAGAC
c-Fos	Mouse	AAGACCGTGTCAGGAGGCAGAG	TTGAGAAGGGGCAGGGTGAAGG
c-Jun	Mouse	CTACGCCAACCTCAGCAACTTC	TGCGGCTGCGAGGGAAAG
Mgat3	Mouse	GCCACAAGGTGCTCTATGTCTTCC	CGCAGGTAGTCATCCGCAATCC
Galnt14	Mouse	TCCGCAGTGTGTTAAACCGAACC	GACCAGACCTTGCCGCTCATTG
St8sia3	Mouse	TTGTACGGTTTCTGGCCCTTTGG	TGGTGAGACTCCTGCCACTTGG
Glt1d1	Mouse	AGGGAAGTGAAAGAGCGGGTGAG	TCGGAGACGGAGCTGTTGACC
Alg6	Mouse	CCCTGTTGACCGTGGACTGTTTG	CTATCTGGATGTGGCGAGGCAAAG
Mgat4a	Mouse	GACTGGCTGCTGGACCACATTC	CTGGAAGAGGGAGGGTCGGAAG
St8sia6	Mouse	TCCTCCTGCGTATGCTCTGGTG	GCTGTTCCTGGTGCGTGGTAC
Gapdh	Mouse	TGGTCCAGGGTTTCTTACTC	GTTGTCTCCTGCGACTTCA

Supplementary Table 13. Lectin and glycan identity in the microarray.

Glycoform	Lectin	Lectin Full Name
Fuc	LTL	Lotus tetragonolobus lectin
	PSA	Pisum sativum agglutinin
	LCA	Lens Culinaris Agglutinin
	UEA I	Ulex europaeus agglutinin I
	AAL	Aleuria aurantia lectin
	LAL	Laburnum anagyroides Lectin (Gold Chain)
	TL	Tulipa sp. Lectin (Tulip)
Sia	MAA	Maackia amurensis Lectin
	SNA, EBL	Sambucus nigra lectin
	SSA	Salvia sclarea
	MAL II	Maackia amurensis lectin II
	SNA-I	Sambucus nigra (Elderberry Bark)
Man	NPL, NPA	Narcissus pseudonarcissus (daffodil) lectin
	ConA	Con A Lectin
	GNL	Galanthus nivalis (snowdrop) lectin
	HHL, AL	Hippeastrum hybrid lectin
	CALSEPA	Calystega sepiem Lectin (Hedge Bindweed Rhizomes)
	AMA	Arum maculatum Lectin (Lords and Ladies)
	MNA-M	Morniga M Lectin (black elderberry)
	VFA	Vicia fava Lectin (Fava Bean)
	VVA mannose	Vicia villosa Lectin (Hairy Vetch, Mannose Specific)
	ASA	Allium sativum Lectin (Garlic)
GlcNAc	GSL II, BSL II	Griffonia (Bandeiraea) simplicifolia lectin II
	LEL, TL	Lycopersicon esculentum lectin

	STL, PL	Solanum tuberosum lectin
	UDA	Urtica dioica Lectin (Stinging Nettle)
	PWM, PWA	Phytolacca americana Lectin (Pokeweed)
	WGA	Triticum vulgare Lectin (Wheat Germ)
	DSL	Datura stramonium lectin
GalNAc	HPA	Helix pomation Lectin (Snail)
	VVL, VVA	Vicia villosa lectin
	DBA	Dolichos biflorus agglutinin
	SBA	Glycine max Lectin (Soybean)
	PTL I, WBA I	Psophocarpus tetragonolobus lectin I
	WFA, WFL	Wisteria floribunda lectin
	CSA	Cytisus sessilifolius Lectin (Portugal Broom)
	Black bean crude	Phaseolus vulgaris sp. Lectin
	GSL-IA4	Pure Griffonia simplicifolia Lectin
	IRA	lris hybrid Lectin (Dutch Iris)
	IAA	Iberis amara Lectin
	HMA	Homarus americanus Lectin (Lobster)
Gal	GHA	Glechoma hederacea Lectin (ground ivy)
	MNA-G	Morniga G Lectin (black elderberry)
	RCA I, RCA120	Ricinus communis agglutinin I
	GSL I-B4, BSL I-B4	GSL I - isolectin B4
	EEL	Euonymus europaeus lectin
	PNA	Arachis hypogaea Lectin (Peanut)
Galβ3GalNAc	BPL	Bauhinia Purpurea Lectin
	ABA	Agaricus bisporus Lectin (Mushroom)
	Jacalin， AIA	Jacalin

	ACL, ACA	Amaranthus caudatus lectin
	MPL	Maclura pomifera lectin
Galβ4GlcNAc	PHA-L	Phaseolus vulgaris Leucoagglutinin
	PHA-E	Phaseolus vulgaris Erythroagglutinin
	ECL	Erythrina cristagalli Lectin
	MAL I	Maackia amurensis lectin I
