# Supplementary material for: Elevated ozone activates maternal immunity to drive offspring IgG-mediated anaphylaxis through IgG glycosylation alterations
Source: Nat Commun. 2026 Sep 23;17:11063. doi: 10.1038/s41467-026-77756-x (PMC13691101; doi:10.1038/s41467-026-77756-x)
Supplement: Supplementary file 2 [file 41467_2026_77756_MOESM2_ESM.pdf]

nature portfolio

Corresponding author(s): Huifeng Yue

Last updated by author(s): Aug 14, 2026

Reporting Summary

Nature Portfolio wishes to improve the reproducibility of the work that we publish. This form provides structure for consistency and transparency in reporting. For further information on Nature Portfolio policies, see our Editorial Policies and the Editorial Policy Checklist.

Statistics

For all statistical analyses, confirm that the following items are present in the figure legend, table legend, main text, or Methods section.

n/a	Confirmed	
☐	☒	The exact sample size (n) for each experimental group/condition, given as a discrete number and unit of measurement
☐	☒	A statement on whether measurements were taken from distinct samples or whether the same sample was measured repeatedly
☐	☒	The statistical test(s) used AND whether they are one- or two-sided<br>*Only common tests should be described solely by name; describe more complex techniques in the Methods section.*
☐	☒	A description of all covariates tested
☐	☒	A description of any assumptions or corrections, such as tests of normality and adjustment for multiple comparisons
☐	☒	A full description of the statistical parameters including central tendency (e.g. means) or other basic estimates (e.g. regression coefficient) AND variation (e.g. standard deviation) or associated estimates of uncertainty (e.g. confidence intervals)
☐	☒	For null hypothesis testing, the test statistic (e.g. F, t, r) with confidence intervals, effect sizes, degrees of freedom and P value noted<br>*Give P values as exact values whenever suitable.*
☒	☐	For Bayesian analysis, information on the choice of priors and Markov chain Monte Carlo settings
☐	☒	For hierarchical and complex designs, identification of the appropriate level for tests and full reporting of outcomes
☐	☒	Estimates of effect sizes (e.g. Cohen's d, Pearson's r), indicating how they were calculated

Our web collection on statistics for biologists contains articles on many of the points above.

Software and code

Policy information about availability of computer code

Data collection	Attune™ Cytometric software
Data analysis	FlowJo (v: 10.8.1) ; R (v: 4.4.2); edgeR (v: 4.4.2); clusterProfiler package (v: 4.14.3) ; GraphPad Prism (v: 8.0.1) ; stats (v: 4.4.2); rcompanion (v: 2.5.0); epiR (v:2.0.80); broom (v:1.0.7); dplyr (v:1.1.4); ggplot2 (v: 3.5.1); library(tidyverse); ComplexHeatmap (v: 2.22.0); Gephi (v:0.10.1); ImageJ (v: 1.50i); corrplot (v: 0.95); PyMOL (v: 2.5.5)

For manuscripts utilizing custom algorithms or software that are central to the research but not yet described in published literature, software must be made available to editors and reviewers. We strongly encourage code deposition in a community repository (e.g. GitHub). See the Nature Portfolio guidelines for submitting code & software for further information.

Data

Policy information about availability of data

All manuscripts must include a data availability statement. This statement should provide the following information, where applicable:

- Accession codes, unique identifiers, or web links for publicly available datasets
- A description of any restrictions on data availability
- For clinical datasets or third party data, please ensure that the statement adheres to our policy

The RNA sequencing data generated in this study have been deposited in the Gene Expression Omnibus (GEO) database under accession code GSE296733 (https://www.ncbi.nlm.nih.gov/geo/query/acc.cgi?acc=GSE296733). The cohort and demographic data are available under restricted access for safeguarding participant privacy and ethical confidentiality; access can be obtained by submitting a reasonable academic research request to the corresponding author within a reasonable

timeframe and executing a formal Data Use Agreement. All other data generated in this study are provided in the Source Data file.

Research involving human participants, their data, or biological material

Policy information about studies with human participants or human data. See also policy information about sex, gender (identity/presentation), and sexual orientation and race, ethnicity and racism.

Reporting on sex and gender	This retrospective cohort study analyzed data from 10,188 pregnant women collected at Bethune Hospital (Taiyuan, Shanxi Province) between January 1, 2018, and December 31, 2020. No sex and gender-based analyses were conducted as part of this investigation.
Reporting on race, ethnicity, or other socially relevant groupings	After adjusting for potential confounding variables including maternal age, pre-pregnancy BMI, education, occupation, monthly family income per person, smoking, dringking, folic acid supplementation, gravidity, parity, and previous adverse pregnancy, logistic regression models were employed to calculate the adjusted odds ratios (ORs) and 95% confidence intervals (CIs) for hematological abnormalities associated with a per 10 μg/m³ increase in gestational period-specific air pollutant exposure.
Population characteristics	In this retrospective cohort study, the demographic and clinical characteristics of participants were described as follows: maternal age (<28 years, n = 1,271; 28–35 years, n = 3,551; >35 years, n = 477), pre-pregnancy BMI (<18.5 kg/m², n = 790; 18.5–24 kg/m², n = 3,514; >24 kg/m², n = 995), education (college degree: yes, n = 4,266; no, n = 1,033), occupation (employed, n = 3,275; unemployed, n = 2,024), monthly family income per capita (≤4,000 yuan, n = 2,654; >4,000 yuan, n = 2,645), smoking status (yes, n = 3; no, n = 5,296), alcohol consumption (yes, n = 5; no, n = 5,294), folic acid supplementation (yes, n = 5,042; no, n = 257), gravidity (≤1, n = 2,642; 2, n = 1,699; ≥3, n = 958), parity (≤1, n = 3,451; 2, n = 1,722; ≥3, n = 126), and history of adverse pregnancy outcomes (yes, n = 413; no, n = 4,886).
Recruitment	Participants in this retrospective cohort were derived from 10,188 mother-infant pairs whose birth records were collected at Bethune Hospital (Taiyuan, Shanxi Province) between January 1, 2018, and December 31, 2020. Informed consent for this study was exempted due to its retrospective design and the use of anonymized clinical data.
Ethics oversight	In this retrospective study, after submitting the clinical research protocol, safety measures, emergency response plan, and ethical review application, the study received ethical approval from the Ethics Committee of Shanxi Bethune Hospital (Approval No. YXLL-KY-2021-016), with exemption from informed consent.

Note that full information on the approval of the study protocol must also be provided in the manuscript.

Field-specific reporting

Please select the one below that is the best fit for your research. If you are not sure, read the appropriate sections before making your selection.

☐ Life sciences ☐ Behavioural & social sciences ☒ Ecological, evolutionary & environmental sciences

For a reference copy of the document with all sections, see nature.com/documents/nr-reporting-summary-flat.pdf

Ecological, evolutionary & environmental sciences study design

All studies must disclose on these points even when the disclosure is negative.

Study description	This study employs quantitative analytical methods to investigate the impact of prenatal ozone (O3) exposure on offspring allergy susceptibility and its underlying mechanisms. Treatment factors include prenatal O3 exposure (an environmental oxidative stress inducer), with a focus on its interactions with maternal immune activation (MIA), offspring IgG glycosylation modifications and inflammatory mediators. The design structure integrates a human retrospective cohort (stratified data analysis of 5,299 pregnancies) and an animal experimental model (nested design: O3-exposed dams followed by observation of allergic phenotypes in offspring). Experimental units in the human study are defined as individual pregnancies, while in the animal model, they comprise dam-offspring pairs. Replication is ensured through a large human cohort and comparative group analyses in animal experiments (each group including control groups), thereby guaranteeing the robustness of the findings.
Research sample	This study integrates multi-dimensional analyses of human cohort data and animal models. Human samples were derived from a retrospective cohort, encompassing 5,299 pregnancies. Data included maternal exposure levels to atmospheric pollutants across distinct gestational windows, maternal hematological indices, and population descriptive characteristics. The data were sourced from the electronic medical record system of Bethune Hospital (Taiyuan, Shanxi Province). Exclusion criteria comprised non-permanent residents, non-singleton deliveries, incomplete medical records, non-live births, pregnancies with gestational age <28 weeks or >42 weeks, maternal age <20 years, infants with congenital malformations, unreliable birth anthropometric data, and mothers diagnosed with pregnancy complications. Animal experimental samples utilized BALB/c strain mice (Mus musculus). Female mice aged 7–8 weeks were exposed to controlled concentrations of O3 (0.5 ppm or 1 ppm, 4 hours/day) during mid-to-late gestation, while control groups received filtered air. Offspring were stratified by sex (male vs. female) and monitored longitudinally until adulthood (8 weeks of age). For animal experiments, biological replicates were incorporated into the analyses to ensure statistical rigor.
Sampling strategy	In the human retrospective cohort study, participants were selected from permanent resident pregnant women in Taiyuan City, Shanxi Province, using the electronic medical record system of Shanxi Bethune Hospital. The cohort was stratified by gestational windows to capture temporal heterogeneity in exposure. Exclusion criteria comprised non-permanent residents, non-singleton deliveries, incomplete medical records, non-live births, pregnancies with gestational age <28 weeks or >42 weeks, maternal age <20 years, infants with congenital malformations, unreliable birth anthropometric data, and mothers diagnosed with pregnancy

complications. A total of 5,299 pregnancies were ultimately included. For the animal experiments, a completely randomized design was employed. Pregnant BALB/c female mice (7–8 weeks old) in mid-to-late gestation were randomly assigned to ozone (O_3) exposure groups (0.5 ppm or 1 ppm, 4 hours/day) or a filtered air control group. Offspring were stratified by sex, to minimize litter effects, fetal samples were systematically randomized across biological replicates from different litters, ensuring both adequate sample size and randomization integrity.

Data collection	Data were collected by researchers using standardized forms and entered into electronic databases in real time. The recording process included periodic cross-checks and dual verification (clinical parameters were cross-verified by healthcare professionals, while experimental data were independently confirmed by laboratory technicians) to ensure completeness and accuracy. Key variables (O3 exposure levels, biospecimen test results) were acquired through automated instruments or laboratory-standardized protocols.
Timing and spatial scale	Human cohort data were collected from January 1, 2018, to December 31, 2020. Participant residential addresses obtained from birth records were converted into latitude and longitude geographic coordinates and spatially matched with the China Air Quality ReAnalysis dataset (CAQRA) to retrieve daily ambient pollutant exposure concentrations for all enrolled mothers from 12 weeks preconception to delivery. Animal experimental data were collected between January 2023 and December 2025, with experiments conducted in a standardized laboratory environment (temperature: 22 ± 2°C, humidity: 50 ± 5%) to minimize environmental interference.
Data exclusions	No data were excluded from the analysis.
Reproducibility	All samples in the human cohort were derived from independent pregnant women, and all animal studies incorporated biological replicates.
Randomization	In the animal experiments, pregnant BALB/c female mice (7–8 weeks old) were assigned to different treatment groups (O3 exposure groups: 0.5 ppm, 1 ppm; control group: filtered air) via a completely randomized design. Post-randomization verification confirmed homogeneity in baseline physiological parameters (body weight and food intake) across groups, thereby eliminating potential confounding effects. To minimize litter effects, fetal samples were systematically distributed across biological replicates from different litters. For the human cohort, as a non-interventional study, participants were not actively grouped; instead, exposure levels were categorized based on retrospective environmental monitoring data. Covariate were rigorously controlled in statistical models to reduce confounding bias.
Blinding	In the animal experiments, data analysis was performed by independent researchers under blinded conditions (group allocation concealed) to minimize subjective bias. For the human retrospective cohort, exposure concentration relied on objective environmental monitoring data, and researchers remained blinded to outcome associations during the analysis phase, ensuring the objectivity of statistical inferences.

Did the study involve field work? ☐ Yes ☒ No

Reporting for specific materials, systems and methods

We require information from authors about some types of materials, experimental systems and methods used in many studies. Here, indicate whether each material, system or method listed is relevant to your study. If you are not sure if a list item applies to your research, read the appropriate section before selecting a response.

Materials & experimental systems

n/a	Involved in the study	
☐	☒	Antibodies
☒	☐	Eukaryotic cell lines
☒	☐	Palaeontology and archaeology
☐	☒	Animals and other organisms
☒	☐	Clinical data
☒	☐	Dual use research of concern
☒	☐	Plants

Methods

n/a	Involved in the study	
☒	☐	ChIP-seq
☐	☒	Flow cytometry
☒	☐	MRI-based neuroimaging

Antibodies

Antibodies used

1. CD45-eFluor™506, clone 30-F11, 69-0451-82, eBioscience;
2. Ly6C-eFluor™450, clone HK1.4, 48-5932-82, eBioscience;
3. Ly6G-FITC, clone 1A8, 11-9668-82, eBioscience;
4. I-A/I-E-PE, clone M5/114.15.2, 12-5321-82, eBioscience;
5. CD11b-PE-Cyanine7, clone M1/70, 25-0112-82, eBioscience;
6. Siglec F-eFluor™660, clone 1RNM44N, 50-1702-82, eBioscience;
7. F4/80-Alexa Fluor™700, clone BM8, 56-4801-82, eBioscience;
8. CD11c-APC-eFluor™780, clone N418, 47-0114-82, eBioscience;
9. CD16/32-PerCP-Cyanine5.5, clone 93, 101323, BioLegend;
10. CD3-FITC, clone 17A2, 11-0032-82, eBioscience;

11. CD4-eFluor™506, clone RM4-5, 69-0042-82, eBioscience;
12. CD25-eFluor™660, clone eBio7D4, 50-0252-82, eBioscience;
13. IFN-γ-PerCP-Cyanine5.5, clone XMG1.2, 45-7311-82, eBioscience;
14. IL-4-PE-Cyanine7, clone 11B11, 25-7041-82, eBioscience;
15. IL-17A-Brilliant Violet™711, clone eBio17B7, 407-7177-82, eBioscience;
16. Foxp3-PE, clone FJK-16s, 12-5773-82, eBioscience;
17. CD16/CD32 Monoclonal Antibody, clone 93, 16-0161-82, eBioscience;
18. Anti-c-Fos, ab222699, Abcam;
19. Anti-c-Jun, ab40766, Abcam;
20. Phospho-c-Fos (Ser362) Rabbit pAb, bs-12910R, Bioss;
21. Anti-c-Jun (Phospho-S63) antibody, clone IGA-10, BM4845, Boster;
22. Anti-β-actin, bs-0061R, Bioss.
23. Anti-FCRN antibody, PB0911, Boster;
24. Cy3 conjugated affinipure goat anti-rabbit IgG, BA1032, Boster;
25. Fluoro 647 conjugated affinipure goat anti-mouse IgG, BA1151, Boster.

Validation

1. https://www.thermofisher.cn/cn/zh/antibody/product/CD45-Antibody-clone-30-F11-Monoclonal/69-0451-82;
2. https://www.thermofisher.cn/cn/zh/antibody/product/Ly-6C-Antibody-clone-HK1-4-Monoclonal/48-5932-82;
3. https://www.thermofisher.cn/cn/zh/antibody/product/Ly-6G-Antibody-clone-1A8-Ly6g-Monoclonal/11-9668-82;
4. https://www.thermofisher.cn/cn/zh/antibody/product/MHC-Class-II-I-A-I-E-Antibody-clone-M5-114-15-2-Monoclonal/12-5321-82;
5. https://www.thermofisher.cn/cn/zh/antibody/product/CD11b-Antibody-clone-M1-70-Monoclonal/25-0112-82;
6. https://www.thermofisher.cn/cn/zh/antibody/product/CD170-Siglec-F-Antibody-clone-1RNM44N-Monoclonal/50-1702-82;
7. https://www.thermofisher.cn/cn/zh/antibody/product/F4-80-Antibody-clone-BM8-Monoclonal/56-4801-82;
8. https://www.thermofisher.cn/cn/zh/antibody/product/CD11c-Antibody-clone-N418-Monoclonal/47-0114-82;
9. https://www.biolegend.com/en-us/products/percp-cyanine5-5-anti-mouse-cd16-32-antibody-6165;
10. https://www.thermofisher.cn/cn/zh/antibody/product/CD3-Antibody-clone-17A2-Monoclonal/11-0032-82;
11. https://www.thermofisher.cn/cn/zh/antibody/product/CD4-Antibody-clone-RM4-5-Monoclonal/69-0042-82;
12. https://www.thermofisher.cn/cn/zh/antibody/product/CD25-Antibody-clone-eBio7D4-7D4-Monoclonal/50-0252-82;
13. https://www.thermofisher.cn/cn/zh/antibody/product/IFN-gamma-Antibody-clone-XMG1-2-Monoclonal/45-7311-82;
14. https://www.thermofisher.cn/cn/zh/antibody/product/IL-4-Antibody-clone-11B11-Monoclonal/25-7041-82;
15. https://www.thermofisher.cn/cn/zh/antibody/product/IL-17A-Antibody-clone-eBio17B7-Monoclonal/407-7177-82;
16. https://www.thermofisher.cn/cn/zh/antibody/product/FOXP3-Antibody-clone-FJK-16s-Monoclonal/12-5773-82;
17. https://www.thermofisher.cn/cn/zh/antibody/product/CD16-CD32-Antibody-clone-93-Monoclonal/16-0161-82;
18. https://www.abcam.cn/products/primary-antibodies/c-fos-antibody-epr21930-238-ab222699;
19. https://www.abcam.cn/products/primary-antibodies/c-jun-antibody-ep693y-ab40766;
20. http://www.bioss.com.cn/prolook_03.asp?id=AF08169606016195&pro37=1;
21. https://www.boster.com/index/products/productsDetail?goods_sn=BM4845;
22. http://www.bioss.com.cn/prolook_03.asp?id=AF68169668816960&pro37=1.
23. https://www.boster.com/index/products/productsDetail?goods_sn=PB0911;
24. https://www.boster.com/index/products/productsDetail?goods_sn=BA1032;
25. https://www.boster.com/index/products/productsDetail?goods_sn=BA1151.

Animals and other research organisms

Policy information about studies involving animals; ARRIVE guidelines recommended for reporting animal research, and Sex and Gender in Research

Laboratory animals

BALB/c mice (7- to 8-week-old, Beijing Vital River Laboratory Animal Technology Co., Ltd.) were acclimatized under controlled conditions (12-hour light/dark cycle, 22 ± 2°C, 50% relative humidity) with ad libitum access to food and water.

Wild animals

N/A

Reporting on sex

This study incorporated sex-based evaluations, revealing notable differences in IgG glycosylation profiles among juvenile male offspring derived from dams exposed to 0.5 ppm ozone-induced MIA. Furthermore, adult male offspring consistently demonstrated heightened allergic susceptibility compared to females.

Field-collected samples

This study did not involve field-collected samples.

Ethics oversight

All animal procedures were approved by the Committee of Scientific Research at Shanxi University (CSRSX; Approval No. SXULL2021018) and conducted in accordance with institutional guidelines.

Note that full information on the approval of the study protocol must also be provided in the manuscript.

Plants

Seed stocks	N/A
Novel plant genotypes	N/A
Authentication	N/A

Flow Cytometry

Plots

Confirm that:

- [x] The axis labels state the marker and fluorochrome used (e.g. CD4-FITC).
- [x] The axis scales are clearly visible. Include numbers along axes only for bottom left plot of group (a 'group' is an analysis of identical markers).
- [x] All plots are contour plots with outliers or pseudocolor plots.
- [x] A numerical value for number of cells or percentage (with statistics) is provided.

Methodology

Sample preparation	Murine lung tissues were dissociated in HBSS (Hanks' Balanced Salt Solution, Solarbio, Beijing, China) containing 0.5 mg/mL collagenase type IA (Sigma-Aldrich, St. Louis, MO, USA) and 20 µg/mL DNase I (Sigma-Aldrich, St. Louis, MO, USA) by mincing, followed by incubation at 37°C for 30 minutes. Single-cell suspensions were generated by filtering through a 70 µm cell strainer, subjected to red blood cell lysis (Solarbio, Beijing, China), and resuspended in PBS supplemented with 2% FBS (Dcell, Shanghai, China). Cell viability (> 95% live cells) was confirmed via trypan blue exclusion assay, and aliquots were prepared for staining.
Instrument	Attune™ NxT Acoustic Focusing Cytometer (Thermo Fisher Scientific, USA)
Software	Samples were acquired using the Attune™ Cytometric software, and data was analyzed using FlowJo (v: 10.8.1) software.
Cell population abundance	Flow cytometry analysis in this study utilized multiparametric gating strategies incorporating forward scatter (FSC), side scatter (SSC), and lineage-specific surface markers to delineate target immune cell subpopulations. No cell sorting was performed in this study.
Gating strategy	For innate immune cells, following exclusion of dead cells and doublets, populations were defined as follows: neutrophils (CD45+CD11b+Ly6G+), eosinophils (CD45+Ly6G−Siglec F+I-A/I-E−), alveolar macrophages (CD45+CD11b−Siglec F+CD11c+), interstitial macrophages (CD45+CD11b+Ly6G−Siglec F−Ly6C−), B cells (CD45+CD11b−I-A/I-E+), and dendritic cells (CD45 +F4/80−CD11c+I-A/I-E+). For T-cell subsets, after excluding doublets and dead cells, populations were identified as: Th1 cells (CD3+CD4+IFN-γ+), Th2 cells (CD3+CD4+IL-4+), Th17 cells (CD3+CD4+IL-17A+), and Treg cells (CD3+CD4+CD25+Foxp3+). Gating strategies are detailed in the Supplementary Information of the manuscript.

- [x] Tick this box to confirm that a figure exemplifying the gating strategy is provided in the Supplementary Information.
