# Supplementary material for: Elevated ozone activates maternal immunity to drive offspring IgG-mediated anaphylaxis through IgG glycosylation alterations
Source: Nat Commun. 2026 Sep 23;17:11063. doi: 10.1038/s41467-026-77756-x (PMC13691101; doi:10.1038/s41467-026-77756-x)
Supplement: Supplementary file 3 [file 41467_2026_77756_MOESM3_ESM.pdf]

nature portfolio

Peer Review File

Elevated Ozone Activates Maternal Immunity to Drive Offspring IgG-Mediated Anaphylaxis through IgG Glycosylation Rearrangement

Corresponding Author: Professor Huifeng Yue

Parts of this Peer Review File have been redacted as indicated to remove third-party material.

This file contains all reviewer reports in order by version, followed by all author rebuttals in order by version.

Version 0:

Reviewer comments:

Reviewer #1

(Remarks to the Author)
Recommendation: Major Revision

Summary
This manuscript explores how prenatal exposure to ozone (O_3) induces maternal immune activation (MIA), reprograms IgG glycosylation, and subsequently enhances allergic susceptibility in offspring—particularly males. The authors use a robust combination of human cohort data, mouse models, lectin microarrays, transcriptomics, and molecular docking to establish a mechanistic link between environmental oxidative stress and immune imprinting.

Strengths
1. Multidisciplinary Integration: The authors successfully bridge epidemiology, immunology, and glycobiology in a coherent framework.
2. Mechanistic Depth: Identification of the c-Fos/c-Jun–Mgat4a axis as an key regulatory pathway is novel and biologically plausible.
3. Sex-specific Analysis: The sex-dependent effects provide important nuance often missing in developmental immune studies.
4. Robust Animal Models: The use of both active and passive systemic anaphylaxis models enhances translational relevance.

Major Concerns
1. Causality and Transfer Efficiency:
- Issue: While glycosylation changes in maternal IgG are noted, the manuscript insufficiently addresses whether these modifications directly impair IgG transplacental transfer or alter neonatal FcRn binding.
- Revision: Include experimental validation (e.g., FcRn-IgG binding affinity assays or ex vivo placental IgG transfer models).
2. Confounding in Human Cohort:
- Issue: The retrospective cohort analysis lacks adjustment for maternal comorbidities and environmental confounders.
- Revision: Clarify in the methods if sensitivity analyses were conducted or explicitly state as a limitation.
3. Immunoglobulin Subclass Specificity:
- Issue: IgG is analyzed in bulk, but subclass-specific glycosylation could exhibit distinct effects.
- Revision: Consider measuring subclass-specific IgG glycoforms or discuss this as a limitation.
4. Mechanistic Validation of IgG-FcγRIII Interaction:
- Issue: Molecular docking results are predictive but lack experimental confirmation.
- Revision: Recommend including future plans for in vitro binding assays in the discussion.

Minor Revisions (indications and suggestion)

Line 36 Mgat3-regulated IgG N-acetylglucosamine (GlcNAc)

Mgat3-mediated addition of bisecting N-acetylglucosamine (GlcNAc) to IgG

Line 60 remain substantially underestimated
are likely underestimated in terms of immunological impact

Lines 84–85 heightened FcγRIIIa affinity, exacerbating...
increased FcγRIIIa affinity, thereby exacerbating...

Line 89 function of their immune system development and functional programming of their immune system

Line 319 shift IgG towards an anti-inflammatory function potentially confer an anti-inflammatory bias to IgG effector functions

Line 420 These results indicate that... Collectively, these findings suggest that...

Line 579 influence offspring's intrinsic IgG-mediated... modulate intrinsic IgG-mediated effector functions in offspring

Figures and Data Quality
- Figure 1–6: Data are compelling; axis labeling in lectin microarrays should clarify fluorescence units and glycan identity.
- Fig. 6d: Provide binding free energy (ΔG) values numerically in main text.
- Supplementary Figures: Consider merging key figures (e.g., Supplementary Figs. 4, 6, and 9) into the main text.

Suggested References
- Martinez D. R. et al., Cell, 2022 — IgG glycoengineering and placental transfer.
- Bouman A. et al., Clin Dev Immunol, 2005 — sex differences in immunity.

Overall Assessment
This study are innovative and potentially impactful, but the causal chain between maternal O_3 exposure, IgG glycosylation, FcRn transfer, and offspring allergic phenotypes requires additional clarification or careful caveating. With the recommended revisions, this manuscript would make a valuable contribution to our understanding of how prenatal environmental insults shape immune ontogeny and allergic risk via antibody-mediated pathways.

Reviewer #2

(Remarks to the Author)
This is an interesting studies, that addresses the impact of o3, which is associated with an increased mortality, and focused on deciphering the role of glycosylation in regulating the immune response.

Major issues
However, ozone largely effect the organism, with beside altering glycan structures, also critically interacts with the body's iron metabolism, which further facilitate inflammation.

- It is thus essential, to include in the logistic regression hemoglobin-data at each pregnancy stage, but also in the mouse data (hgb should be included, with a hgb<11 in pregnant women considered anemic).
- Further, what is really missing is an in vitro model where bisecting N-acetylglucosamine (GlcNAc) modifications in IgG glycosylation profiles are generated and are subsequently given to mice to assess their direct contribution to anaphylaxis.
- Several studies have consistently shown that male mice tend to develop greater ozone-induced AHR compared to female mice. Consequently, elevated maternal IgG cannot be really linked to the observed greater susceptibility. – please rephrase, discuss
- Differences in Th1/Th2 profile, please provide specific IgE-data
- All graphs should also depict single values as points

Fig 1
-difference between f and g (y-axis is wrong, as it probably should mean spleen
-Please show in every graphical presentations single points
-Also as less Th2 cells are seen and less ag-presentation is suggested, please show antigen-specific IgE-levels from the different mice groups.
Please detail better the schematic the immunization/exposure strategy of ASA /PSA and extra panel detailing the sensitization model and in bigger letters would be welcomed
- Please write in the figure legend, which statististical test were employed in which graph.

Figure 2, rbc-values, hemoglobin-values per group and sex, RBC count of the different pups should be added
- Please make the graphical presentation of b as in d and only of those cytokines that are significant, as here better visual comparison can be made .
- Please elaborate the results in regard to O3 exposure, rather than male/female
- No difference can be seen in lung inflammation between ALA and AHA in regard to H&E staining- please explain in the result section
- How was the -T-SNE anaylsis conducted, which perplexity and step size was used?
- please interpret the two B cell cluster in AHS

Figure 3
- Please indicate in each graph, whether this is maternal IgG or from the pups, please show single points, if a pool was measured, please indicate in text and figure legend
- Add hemoglobin-levels of mice exposed to saline/0.5 and 1 ppm O3
- Please show sugar-binding not only by gender, but also by o3 exposure

Figure 4
- Please add hb-values per 03 exposure
- - please show individual points in all graphs
- - why is the 1ppm O3 missing now in figure 4e, h
Figure 6
- while the different glycosylation surely has an impact, enhanced allergic reaction is not shown in any experiments, this should be shown using in vitro generated antibodies and monitoring for anaphylaxis, otherwise this is only speculation. However, inflammation seems to be facilitated

Minor
• Please explain the abbreviation, if possible write the whole word and do not use abbrevitations

• Statistical test employed should be stated in the figure legends and shown in the figure with * for p<0.05, **p<0.01 and so on, but the specific p-Values between the different groups should not be in the figure legend

Reviewer #3

(Remarks to the Author)
The manuscript entitled “Elevated ozone activates maternal immunity to drive offspring allergy through IgG glycosylation reprogramming” aimed to rely prenatal oxidative stress during pregnancy induced by exposure to ozone, to offspring immunity – notably propensity to develop allergy. The authors postulate the effects resulted from modification of IgG glycosylation in early life.
While timely and of scientific interest, the study presented many issues and impreciseness that doesn’t support its acceptance.

Major concerns
First, in my opinion, the causality between IgG glycosylation and immune response in the progeny is not demonstrated. Actually, we cannot ascertain that it is altered immunity in offspring that results in modification of IgG glycosylation profiles, and not the reverse as stated by authors: oxidative stress induced by ozone may induce various effects in mothers then progeny, for example altering mother microbiota composition (and then that of the progeny) and epigenetic mark in tissues and cells in utero, all of which may alter immunity in offspring independently of IgG glycosylation. Moreover, exposure has a great impact on weight and dietary intake of mothers, which are stress impacting in utero development and functions maturation (as exemplified in many parameters in supplementary figures S2 and S3 – on the opposite to the authors statement lines 138-139).
For example, were adoptions tested to address the respective role of in utero vs lactation (and then potentially gly-IgG transfer through yolk sac vs milk) phases in the effects observed in progeny? What would be the results using IgG deficient dams or FcRn deficient mice? Concerning IgG transfer through yolk sac, it concerns only IgG2 and IgG3 whereas IgG1 are only transfer through breast milk (pioneer studies form Morshed et al., 1993 & Grey et al., 1971)? Could you please discuss your results in front of that knowledge?
Moreover, isotype-specific glycosylation is not explored at all despite studies demonstrating that glycosylation and its functional effects are different for IgG1 and IgG3 versus IgG2 in mice (Barrientos et al., 2020), as observed in humans (Sneed et al., 2025). A more precise and deep analysis of sub-isotype glycosylation appear necessary to draw conclusions.
In addition, IgG-dependent anaphylaxis is more relevant for high doses of circulating antigens, whereas IgE-dependent anaphylaxis is the mechanism involved at low dose of antigen and at mucosal sites, i.e. food or respiratory allergic reactions. Indeed, as clearly reviewed in Reber et al (cited as ref 22 by authors), IgG-mediated anaphylaxis requires a much larger dose of antigen than does IgE-mediated anaphylaxis, and systemic anaphylaxis also requires systemic absorption of ingested antigen. Such conditions could be encountered in the case of anaphylaxis occurring in response to infusion of large quantities of a drug or a therapeutic monoclonal antibodies. In that context, the rationale of the present study, and the animal models used, does not seem relevant and transposable to human. Moreover, induction of severe systemic anaphylaxis remain, in my opinion, ethically criticizing (2 out 8 males mice in the ALA group exhibited signs of anaphylactic shock and loss of vital functions; decline of core temperature superior to 5°C): what were the limit points and were those mice sacrificed to limit suffering?
In the same line, what about IgE transfer through milk and their glycosylation?
It should have been interesting to present and to discuss the role anti-inflammatory role of terminal galactose/sialic acid vs the pro-inflammatory role of agalactosylated IgG, and the natural evolution from agalactosylated to galactosylated/sialylated after weaning in mice. For example, this decrease of pro-inflammatory profile parallels the transition form innate passive immunity to endogenous production of IgG, and the corresponding timeline (also considering half-life of mother transferred IgG). In my opinion, glycosylation and its specificity is not deeply present and discussed in regards to the results and discussion.
There is also some issues in experimental section, and one of the major concern is also the number of mice per groups – that are far too low in my opinion, even in a reduction context of animal use for science. As an example, n=3 for flow cytometry analysis (fig 1, 2 and 4), cytokines, or lung histology and functions (fig 2 and S7)…., and 3-8 mice are shown in allergy models, with variable numbers between groups and figures, so it’s difficult to assess number of mice in each

experiments/results. Figures should be provided as individual point for each mouse and median. Moreover, how were managed the littermates for the allergy models? How many mothers and litters were considered and were litters mixed within exposure groups?
Considering result from human cohort, we can also observe that O3 exposure during T1 has a significant anti-inflammatory effect (table 1) – i.e. the opposite effect of exposure in mid-to-late pregnancy: this has to be presented and discussed
I'm not convinced by the conclusion "collectively, … whereas 1 ppm O3" appear to elicit immunosuppressive effects" (lines 158-161). In my opinion, cytometry results presented in figure 1 did not allow to affirm such a conclusion. Moreover, were cytokines assessed in dams lung tissue or blood?
How do you explain differences in anti-Ova IgG levels between groups in the passive anaphylaxis model, since same doses of anti-IgG OVA should have been administered to mice! (lines 193 and following)?
Moreover, if I correctly understand, each lectin probe included triplicate technical replicate (lines 883—884), meaning that figure f and g show results from these technical replicates, and not that of individual mice with n=3. You can not perform statistical analysis in those conditions and draw conclusion that glycosylation patterns form dams and males/females offspring are different (all the more considering the high heterogeneity within technical triplicates!).
Were IgG concentrations ang glycosylation assessed in milk from mothers at PND14?

Minor points
Introduction:
Revise sentences lines 56-59 and add ref
Lines 70-72: please specify at what PND transfer of IgG from mother to infant via milk end (both in human and mice).
Lines 72; please clarify the sentence "IgG play a fundamental role in opsonizing innate immune cells… to enhance their function."
Methods & results
Please provide a ref for the clinical reference ranges considered (lines 675-679), and the time of blood collection for mothers (at delivery??)
How euthanasia of pregnant dams was performed (please be more precise that "humanely euthanized")?
Please specify the number of mice of both sex at each time point in progeny (i.e. at PND14 and PND56 for pulmonary testing and tissue collection), the same for mice submitted to systemic allergic reaction model.
Please mention if samples from mice were treated individually or as pools (i.e. for flow cytometry analysis, ELISA, RT PCR, IgG-glycosylation assessment…)
I'm not convinced pentobarbital is the best anaesthesia to perform lung function assessment in mice?
What is the isotype of OVA-specific IgG used in the passive anaphylaxis model, and the purity of both IgG and BSA injected i.v.? (line 750)
Multiplex assay : There is an issue concerning the assay, with a confuse methodology mixing MSD analysis (solid phase) and Luminex (bead assay) one. This has to be clarified.
Please translate rpm to xg , antibodies dilution to exact quantities (e.g. µg/ml for western blot, or µg/10e cells for cytometry) to ensure reproducibility of experiments
Flow cytometry analysis: Please provide FMO and precise how many cells were acquired.
Figure 5h: for DSL fluorescence, C vs 0.5 ppm tend to be different, but this is not significant : please modify corresponding text (lines 412-413)

Version 1:

Reviewer comments:

Reviewer #1

(Remarks to the Author)
I have reviewed the revised version of the manuscript entitled "Elevated Ozone Activates Maternal Immunity to Drive Offspring Allergy through IgG Glycosylation Reprogramming" (Manuscript ID: NCOMMS-25-29897).

I am satisfied that the authors have carefully and thoughtfully addressed all of the concerns raised during the review process. The additional experiments and analyses meaningfully strengthen the study, particularly with respect to the proposed mechanistic link between prenatal ozone exposure, maternal IgG glycosylation, FcRn-mediated antibody transfer, and the sex-specific immune outcomes observed in the offspring. The expanded treatment of IgG subclass specificity, receptor interactions, and the human cohort analyses adds important clarity and depth to the work.

In its current form, the manuscript is clear, rigorous, and well balanced. It offers genuinely novel insight into how environmental exposures during pregnancy can influence immune development through antibody-mediated mechanisms, and it will be of broad interest to readers working in immunology, environmental health, and developmental biology.

I have no remaining concerns and believe the manuscript now meets the standards for publication in Nature Communications.

I therefore recommend acceptance of this manuscript.

Reviewer #2

(Remarks to the Author)
Thank you for the revised responses. I have three remaining concerns.

(1) Thank you for providing the unadjusted logistic regression models for haematological abnormalities. However, my comment regarding haemoglobin was not that HGB should be included as an adjustment variable in those models, but rather that it should be evaluated directly as an endpoint to determine whether ozone-exposure is associated with altered HGB in the last pregnancy stage (the only stage for which HGB appears available).
Please therefore add an analysis of HGB vs exposure (restricted to the last pregnancy stage), reporting group-wise HGB values (n and mean±SD or median[IQR]), an effect estimate with 95% CI, and the statistical test used. This will allow readers to see whether HGB changes with exposure. Any HGB-adjusted logistic model may be retained as a sensitivity analysis, but it does not replace reporting whether HGB differs by exposure
(2) Statistical reporting: The manuscript still lists omnibus tests (e.g., ANOVA) without specifying the post-hoc procedure. Please state which post-hoc test(s) were used, whether comparisons were versus control only or all pairwise, and how multiplicity was controlled. Where relevant, indicate assumption checks and the alternative tests used if assumptions were violated.
(3) Mechanistic support for IgG glycosylation: I understand the stated technical limitation preventing in vitro generation of defined IgG glycoforms. However, the current data remain largely associative. To strengthen the claim of a direct and meaningful contribution to anaphylaxis, it would be helpful to isolate/enrich the specific glycosylated IgG fraction from ozone-exposed mice and test its anaphylactic potential (or Fc-dependent effector activity) compared with an appropriate control fraction, with glycoform composition verified analytically and IgG dose/subclass matched. If such experiments are not feasible, the mechanistic claims should be tempered accordingly.
All other points appear adequately addressed.

Reviewer #3

(Remarks to the Author)
Dear authors,
Thanks for taking into consideration all points raised by the reviewers, and for carefully answering each remark - either by clarifying the text or by providing additional experiments.
Two points for the reviewed manuscript:
- I would really prefer the authors used "IgG-mediated anaphylaxis" instead of "allergic reaction"
- what is the rational for assessing anti-OVA IgE in the passive anaphylaxis model!!!! Results may correspond to non-specific signals and should be removed!

Version 2:

Reviewer comments:

Reviewer #2

(Remarks to the Author)
I have carefully reviewed the revised manuscript and the authors’ responses and am pleased to see that all of my comments have been satisfactorily addressed.

Open Access This Peer Review File is licensed under a Creative Commons Attribution 4.0 International License, which permits use, sharing, adaptation, distribution and reproduction in any medium or format, as long as you give appropriate credit to the original author(s) and the source, provide a link to the Creative Commons license, and indicate if changes were made.
In cases where reviewers are anonymous, credit should be given to 'Anonymous Referee' and the source.
The images or other third party material in this Peer Review File are included in the article's Creative Commons license, unless indicated otherwise in a credit line to the material. If material is not included in the article's Creative Commons license and your intended use is not permitted by statutory regulation or exceeds the permitted use, you will need to obtain permission directly from the copyright holder.
To view a copy of this license, visit https://creativecommons.org/licenses/by/4.0/

Title: Elevated Ozone Activates Maternal Immunity to Drive Offspring Allergy through IgG Glycosylation Reprogramming

The point-by-point response to the Reviewers' comments

Reviewer 1 Comments for the Author

Summary: This manuscript explores how prenatal exposure to ozone (O_3) induces maternal immune activation (MIA), reprograms IgG glycosylation, and subsequently enhances allergic susceptibility in offspring—particularly males. The authors use a robust combination of human cohort data, mouse models, lectin microarrays, transcriptomics, and molecular docking to establish a mechanistic link between environmental oxidative stress and immune imprinting.

Strengths: 1. Multidisciplinary Integration: The authors successfully bridge epidemiology, immunology, and glycobiology in a coherent framework. 2. Mechanistic Depth: Identification of the c-Fos/c-Jun Mgat4a axis as an key regulatory pathway is novel and biologically plausible. 3. Sex-specific Analysis: The sex-dependent effects provide important nuance often missing in developmental immune studies. 4. Robust Animal Models: The use of both active and passive systemic anaphylaxis models enhances translational relevance.

Authors:

We thank for reviewer's encouraging comments and helpful suggestions. We carefully addressed every comment by the reviewer.

Response to comment

Major Concerns:

Reviewer:

1. Causality and Transfer Efficiency: - Issue: While glycosylation changes in maternal IgG are noted, the manuscript insufficiently addresses whether these modifications directly impair IgG transplacental transfer or alter neonatal FcRn binding. - Revision: Include experimental validation (e.g., FcRn-IgG binding affinity assays or ex vivo placental IgG transfer models).

Authors:

We thank the reviewer for this helpful suggestion. We fully agree that investigating whether maternal IgG glycosylation alterations directly and functionally regulate its transplacental transfer efficiency is crucial for elucidating the core mechanism of our study. Following the reviewer's advises, we have supplemented a yolk sac staining experiment to address this issue in the revised manuscript.

As elaborated in the Introduction of our manuscript, maternal IgG is transferred to the fetus during mid-to-late gestation. In mice, the primary route is via the yolk sac barrier. This transfer is most active in late gestation, with embryonic day 18.5 (E18.5) representing the final critical window for fetal acquisition of maternal IgG before birth. Therefore, at this time point, we collected yolk sac tissues separately according to offspring sex. By performing triple immunofluorescence co-staining on these tissues for FcRn (the transfer receptor), total IgG, and specific lectins (the differential lectins

identified at E18.5 in IgG glycosylation analysis: GHA for the 0.5 ppm group and TL for the 1 ppm group (Fig. 3f in the revised manuscript)), we were able to directly visualize and quantitatively assess whether glycosylation-remodeled IgG interacts with the FcRn receptor within the native tissue microenvironment.

Quantitative results showed that in the yolk sacs of male fetuses exposed to 0.5 ppm, we observed a significant increase in both the Manders' colocalization coefficient for IgG with FcRn and the intensity of the GHA lectin signal. Analysis of individual samples within this group further indicated a positive correlation between the degree of glycosylation and receptor engagement (colocalization coefficient). Similarly, in the 1 ppm (TL-binding) exposure group, male fetuses exhibited significant increases in both the colocalization coefficient and the lectin signal intensity. Critically, correlation analysis confirmed a strong and statistically significant positive relationship ($\rho = 0.829$) between these two parameters. This consistent triad of findings—enhanced lectin signal, increased FcRn colocalization, and a positive correlation between them across two independent lectin markers—indicates that the exposure-induced IgG glycosylation modifications actively promote the binding of maternal IgG to the FcRn in the yolk sac. This enhanced interaction at the critical transfer interface directly explains the subsequent elevation of serum IgG levels observed in male offspring at PND14. However, in female offspring, exposure decreased the lectin signals (GHA $p = 0.050$; TL $p = 0.003$). Further, the overall relationship between the glycosylation marker and receptor colocalization differed from the clear positive association seen in males. This suggests that in the female fetal environment, the processing or trafficking of glycosylated IgG may involve distinct pathways or compensatory mechanisms, potentially leading to its deviation from the efficient FcRn-mediated transfer route. This altered tissue-level handling aligns with the ultimate physiological outcome of reduced serum IgG accumulation in females at PND14 (Fig 1 in the response to reviewers).

We also attempted to perform the FcRn-IgG binding affinity assays as suggested. However, after consulting with GLYCOGENE Pharmaceutical Co., Ltd. (Wuhan, China; specializes in antibody glycosylation modification), GlycanLink Biomedicine Co., Ltd. (Shanghai, China), and glycochemistry expert Prof. Feng Tang from the Shanghai Institute of Materia Medica (Shanghai, China), it was concluded that, due to the inherent complexity of synthesizing the mouse specific IgG glycoforms identified in our study in sufficient quantity and purity, we were unable to proceed with these *in vitro* binding experiments. Meanwhile, the primary route of mouse IgG crosses the placenta is via the yolk sac barrier, setting up an *ex vivo* mouse yolk sac transfer model presented significant technical challenges, which led us to prioritize the *in vivo* yolk sac approach that directly captures the physiological context.

These findings demonstrate that glycosylation modifications alter the binding of IgG to the FcRn receptor, which directly leads to differences in IgG transfer efficiency. We have incorporated these results into the revised manuscript (lines 361-367).

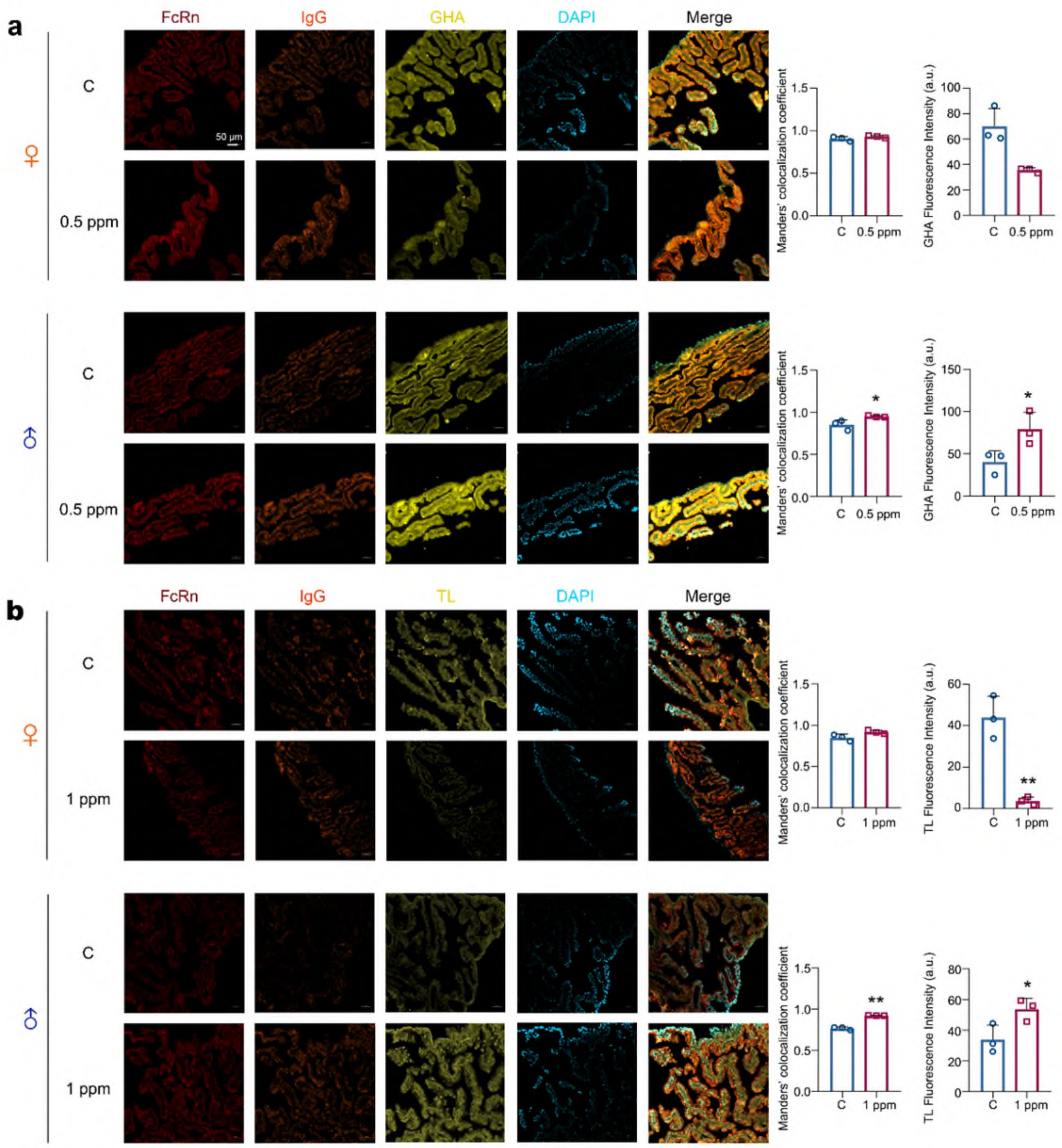

Fig. 1. Co-localization and quantification of FcRn, IgG, and differential lectin staining in E18.5 mouse yolk sacs. **a** Left: Representative immunofluorescence images of yolk sac sections from control and 0.5 ppm O_3-exposed groups at E18.5, stained for FcRn (red), IgG (orange), and FITC-conjugated GHA lectin (yellow), with DAPI counterstain (cyan), scale bar, 50 μm. Right: Quantification of the FcRn & IgG Manders' colocalization coefficient and the fluorescence intensity of GHA (6 random fields per sample). Top panels show female fetal yolk sacs; bottom panels show male fetal yolk sacs (n = 3). **b** Left: Representative images of yolk sac sections from control and 1 ppm O_3-exposed groups at E18.5, stained for FcRn (red), IgG (orange), and FITC-conjugated TL lectin (yellow), with DAPI (cyan), scale bar, 50 μm. Right: Quantification of FcRn & IgG Manders' colocalization coefficient and TL fluorescence intensity (6 random fields per sample). Top: female; bottom: male (n = 3). Data were presented as mean ± SD. Statistical analysis was performed by two-tailed Student's t-test. $^{*}p < 0.05$ and $^{**}p < 0.01$ compared with the corresponding control group.

Reviewer:

2. Confounding in Human Cohort: - Issue: The retrospective cohort analysis lacks adjustment for maternal comorbidities and environmental confounders. - Revision: Clarify in the methods if sensitivity analyses were conducted or explicitly state as a limitation.

Authors:

We sincerely thank the reviewer for the insightful recommendations on adjusting maternal comorbidities and environmental confounders in our retrospective cohort analysis. To address maternal comorbidities, our study implemented rigorous exclusion criteria that systematically eliminated mothers with gestational complications (including hypertensive disorders such as gestational hypertension and preeclampsia, gestational diabetes, and infections such as syphilis, HIV, hepatitis, rubella, HSV, CMV, influenza, and gonorrhea) as well as those with pre-existing chronic conditions (including chronic hypertension, diabetes, cardiovascular/cerebrovascular diseases, and malignancies). Specifically, 920 mothers meeting these exclusion criteria were removed during data screening, as detailed shown in Fig. S1 in the revised manuscript (Flowchart of participant inclusion and exclusion criteria for the retrospective study). We sincerely apologize for the ambiguity in our prior description and have revised the Methods section in the revised manuscript (lines 904-906) to clarify: "Subsequent exclusions eliminated infants with congenital malformations, births with implausible anthropometric data, and mothers diagnosed with gestational comorbidities or pre-existing chronic conditions."

To account for potential confounding from other concurrently present air pollutants, we conducted multi-pollutant regression analyses, adjusting for $PM_{2.5}$, NO_2, SO_2 and PM_{10} as a primary potential confounder to isolate the specific effects of O_3, respectively. Recognizing the window-specific associations identified in primary analyses, we conducted targeted analyses adjusting for $PM_{2.5}$, NO_2, SO_2 and PM_{10} exposures during critical exposure windows (mid-late gestation and the third trimester) to evaluate the robustness of O_3 effects, respectively. As shown in our results, after $PM_{2.5}$ adjustment, each 10 $\mu g/m^3$ increase in third-trimester O_3 exposure was associated with a 1.8% (95% CI: 1.005-1.032), 2.1% (95% CI: 1.007-1.035), and 3.6% (95% CI: 1.016-1.057) increased risk of neutrophil, monocyte, and fibrinogen abnormalities, respectively. NO_2 adjustment similarly showed persistent associations for monocyte (2.5%; 95% CI: 1.005-1.045) and fibrinogen (9.4%; 95% CI: 1.063-1.127) abnormalities. Similar results were observed after adjustment for SO_2 and PM_{10}, with the associations for O_3 generally remaining consistent with the original analysis (Tables 1-4 in the response to reviewers). These results from the additional sensitivity analyses have been included in the revised manuscript (lines 139-142).

While these exclusion strategies and analytical approaches mitigate key confounding from maternal comorbidities and coexisting air pollutants, we acknowledge potential residual confounding from unmeasured environmental or lifestyle factors. To address this limitation, we have added the following statement to the Discussion section (lines 688-691): "Additionally, while this study has controlled for major confounding factors through analysis, potential bias from unmeasured variables may still exist. Further measurement and analysis of relevant variables are

required in future research."

Table 1. Associations of trimester-specific O_3 exposure with risks of hematological parameter abnormalities: Single-pollutant and $PM_{2.5}$ adjusted models

	Single-pollutant model			$PM_{2.5}$-adjusted model		
	OR	95% CI	*p*	OR	95% CI	*p*
**T3**						
WBC	0.990	0.978 to 1.003	0.136	0.987	0.974 to 1.001	0.064
Neu	1.016	1.004 to 1.029	0.012*	1.018	1.005 to 1.032	0.006**
Lym	0.986	0.967 to 1.004	0.127	0.987	0.968 to 1.006	0.190
Mon	1.022	1.008 to 1.035	0.002**	1.021	1.007 to 1.035	0.003**
Eos	0.979	0.853 to 1.120	0.761	0.981	0.850 to 1.127	0.791
Bas	1.005	0.936 to 1.079	0.886	0.994	0.924 to 1.070	0.873
FIB	1.030	1.010 to 1.050	0.003**	1.036	1.016 to 1.057	< 0.001***
**T2-T3**						
WBC	0.993	0.974 to 1.012	0.474	0.992	0.973 to 1.012	0.446
Neu	1.020	1.001 to 1.039	0.044*	1.020	1.001 to 1.040	0.044*
Lym	0.993	0.965 to 1.021	0.607	0.995	0.967 to 1.024	0.732
Mon	1.021	1.000 to 1.041	0.048*	1.019	0.998 to 1.040	0.073
Eos	0.859	0.678 to 1.058	0.172	0.871	0.685 to 1.077	0.222
Bas	0.976	0.876 to 1.086	0.652	0.957	0.858 to 1.035	0.430
FIB	0.990	0.961 to 1.019	0.498	1.000	0.971 to 1.030	0.995

Note: * p < 0.05, ** p < 0.01, *** p < 0.001

Table 2. Associations of trimester-specific O_3 exposure with risks of hematological parameter abnormalities: Single-pollutant and NO_2 adjusted models

	Single-pollutant model			NO_2-adjusted model		
	OR	95% CI	*p*	OR	95% CI	*p*
**T3**						
WBC	0.990	0.978 to 1.003	0.136	0.985	0.966 to 1.004	0.111

Neu	1.016	1.004 to 1.029	0.012*	1.009	0.991 to 1.028	0.335
Lym	0.986	0.967 to 1.004	0.127	0.979	0.952 to 1.006	0.132
Mon	1.022	1.008 to 1.035	0.002**	1.025	1.005 to 1.045	0.016*
Eos	0.979	0.853 to 1.120	0.761	1.005	0.823 to 1.225	0.958
Bas	1.005	0.936 to 1.079	0.886	0.987	0.891 to 1.095	0.805
FIB	1.030	1.010 to 1.050	0.003**	1.094	1.063 to 1.127	< 0.001***
**T2-T3**						
WBC	0.993	0.974 to 1.012	0.474	0.987	0.961 to 1.013	0.317
Neu	1.020	1.001 to 1.039	0.044*	1.014	0.988 to 1.041	0.283
Lym	0.993	0.965 to 1.021	0.607	0.972	0.936 to 1.009	0.134
Mon	1.021	1.000 to 1.041	0.048*	1.019	0.991 to 1.047	0.182
Eos	0.859	0.678 to 1.058	0.172	0.875	0.650 to 1.176	0.370
Bas	0.976	0.876 to 1.086	0.652	0.979	0.849 to 1.136	0.773
FIB	0.990	0.961 to 1.019	0.498	1.067	1.023 to 1.114	0.003**

Note: * *p* < 0.05, ** *p* < 0.01, * *p* < 0.001**

Table 3. Associations of trimester-specific O_3 exposure with risks of hematological parameter abnormalities: Single-pollutant and SO_2 adjusted models

	Single-pollutant model			SO_2-adjusted model		
	OR	95% CI	*p*	OR	95% CI	*p*
**T3**						
WBC	0.990	0.978 to 1.003	0.136	0.993	0.976 to 1.012	0.491
Neu	1.016	1.004 to 1.029	0.012*	1.010	0.992 to 1.028	0.280
Lym	0.986	0.967 to 1.004	0.127	0.972	0.947 to 0.997	0.033
Mon	1.022	1.008 to 1.035	0.002**	1.020	1.000 to 1.038	0.043*
Eos	0.979	0.853 to 1.120	0.761	1.010	0.837 to 1.214	0.916
Bas	1.005	0.936 to 1.079	0.886	1.033	0.937 to 1.140	0.508
FIB	1.030	1.010 to 1.050	0.003**	1.095	1.066 to 1.125	< 0.001***

**T2-T3**						
WBC	0.993	0.974 to 1.012	0.474	0.999	0.976 to 1.023	0.943
Neu	1.020	1.001 to 1.039	0.044*	1.009	0.986 to 1.032	0.453
Lym	0.993	0.965 to 1.021	0.607	0.979	0.947 to 1.012	0.218
Mon	1.021	1.000 to 1.041	0.048*	1.022	0.997 to 1.046	0.082
Eos	0.859	0.678 to 1.058	0.172	0.826	0.631 to 1.055	0.140
Bas	0.976	0.876 to 1.086	0.652	0.983	0.866 to 1.118	0.797
FIB	0.990	0.961 to 1.019	0.498	1.076	1.039 to 1.115	< 0.001***

Note: * $p < 0.05$, ** $p < 0.01$, *** $p < 0.001$

Table 4. Associations of trimester-specific O_3 exposure with risks of hematological parameter abnormalities: Single-pollutant and PM_{10} adjusted models

	Single-pollutant model			PM_{10}-adjusted model		
	OR	95% CI	*p*	OR	95% CI	*p*
**T3**						
WBC	0.990	0.978 to 1.003	0.136	0.993	0.979 to 1.007	0.338
Neu	1.016	1.004 to 1.029	0.012*	1.010	0.996 to 1.025	0.146
Lym	0.986	0.967 to 1.004	0.127	0.980	0.960 to 1.001	0.065
Mon	1.022	1.008 to 1.035	0.002**	1.022	1.007 to 1.038	0.004**
Eos	0.979	0.853 to 1.120	0.761	0.972	0.830 to 1.127	0.710
Bas	1.005	0.936 to 1.079	0.886	0.976	0.920 to 1.059	0.566
FIB	1.030	1.010 to 1.050	0.003**	1.068	1.046 to 1.091	< 0.001***
**T2-T3**						
WBC	0.993	0.974 to 1.012	0.474	0.996	0.977 to 1.017	0.732
Neu	1.020	1.001 to 1.039	0.044*	1.016	0.996 to 1.036	0.127
Lym	0.993	0.965 to 1.021	0.607	0.987	0.958 to 1.016	0.365
Mon	1.021	1.000 to 1.041	0.048*	1.021	1.000 to 1.043	0.049*
Eos	0.859	0.678 to 1.058	0.172	0.848	0.668 to 1.052	0.149

Bas	0.976	0.876 to 1.086	0.652	0.965	0.863 to 1.078	0.528
FIB	0.990	0.961 to 1.019	0.498	1.028	0.997 to 1.060	0.082

Note: * $p < 0.05$, ** $p < 0.01$, *** $p < 0.001$

Reviewer:

3. Immunoglobulin Subclass Specificity: - Issue: IgG is analyzed in bulk, but subclass-specific glycosylation could exhibit distinct effects. - Revision: Consider measuring subclass-specific IgG glycoforms or discuss this as a limitation.

Authors:

We sincerely thank the reviewer for raising the critical point regarding subclass-specific IgG glycosylation. We agree that focusing solely on total IgG may obscure the distinct functional contributions of individual subclasses. In direct response to this suggestion, we performed comprehensive supplementary analyses to profile the concentrations and glycosylation patterns of both total IgG and its subclasses in maternal (E18.5) and offspring (PND14) sera (Fig. 2, 3 in the response to reviewers).

Our results revealed a significant redistribution of subclass abundance at E18.5 despite unchanged total IgG levels: IgG1 was downregulated in the O_3-exposed groups, IgG2a was specifically elevated in the 0.5 ppm group, while IgG3 was reduced. Crucially, lectin microarray analysis uncovered that each altered subclass acquired a distinct glycosylation signature. In the 0.5 ppm group, the overall increase in galactosylation (reflected by stronger binding to GHA) was driven by elevated levels of pro-inflammatory glycan motifs—specifically, increased galactosylation (e.g., Galβ4GlcNAc and Galβ3GalNAc) on the IgG2a and IgG2b subclasses. In contrast, the 1 ppm group induced a different pattern (overall increase in fucosylation reflected by stronger binding to TL), characterized by elevated sialylation (SNA, SNA-I binding) and mannosylation on the IgG1 subclass, which were modifications associated with immunoregulatory or anti-inflammatory functions.

This pre-conceptionally programmed maternal antibody "blueprint" was transmitted and manifested in the offspring in a remarkable sex-specific manner by PND14. Male offspring from the 0.5 ppm MIA group exhibited elevated total IgG, driven primarily by an expansion of IgG1. Glycosylation analysis showed that this expanded IgG1 pool in males was armed with pro-inflammatory modifications, such as increased GlcNAc (DSL binding), aligning with the total IgG profile. Concurrently, their IgG2a displayed enhanced fucosylation, providing anti-inflammatory balancing. Female offspring from the 1 ppm group presented a contrasting profile: reduced total IgG due to decreased IgG1, and a specific "disarming" of their IgG2a via a reduction in galactosylated structures (e.g., decreased binding to ACL (Galβ3GalNAc) and PHA-E (Galβ4GlcNAc)), resulting in a net anti-inflammatory or protective phenotype.

In response to the reviewers' suggestions, we have incorporated these detailed analyses of IgG subclasses (including both concentrations and glycosylation patterns) into the Results section of the revised manuscript (lines 370-378 and lines 385-396), and have added a discussion on the functional implications of subclass-specific glycosylation changes in the Discussion section:

1) "Under this exposure condition, the immune system exhibited a clear Th cell shift, which directly modulated the antibody response of B cells. It is well documented that IFN-γ secreted by Th1 cells promotes the production of IgG2a, while Th2-derived IL-4 drives the generation of IgG1. Consistent with this classical regulatory paradigm, we observed a significant increase in IgG2a and a notable decrease in IgG1 levels in the 0.5 ppm exposure group at E18.5, aligning with the Th1-biased immune state in this group. Furthermore, this distinct immune environment also guided a specific remodeling of antibody glycosylation. In the 0.5 ppm group, galactosylated structures were specifically enriched in the effector subclasses IgG2a and IgG2b. In contrast, in the 1.0 ppm group, which exhibited a different immune response profile, anti-inflammatory sialylation was selectively loaded onto the IgG1 subclass." (lines 696-707)

2) "We further observed that the functionally biased IgG glycosylation patterns established in maternal serum persist from gestation into the lactation period, which may be facilitated by altered binding dynamics to the FcRn. In offspring at PND14, maternally derived IgG retained its specific glycosylation features and exhibited marked sexual dimorphism: total IgG levels were elevated in male offspring but decreased in females. Notably, male offspring from the 0.5 ppm O_3-induced MIA group showed the most significant IgG glycosylation alterations, including increased N-acetylglucosamine and core fucosylation along with reduced mannosylation. Subclass analysis revealed that the increase of total IgG in males is primarily driven by expansion of IgGl, on which pro-inflammatory GlcNAc modifications were predominantly enriched, whereas anti-inflammatory fucosylation was observed on IgG2a. This phenomenon finds a plausible explanation within the theoretical framework of subclass-specific IgG transport. Classical studies have confirmed that IgG transport in mice exhibits distinct subclass specificity: IgG2a and IgG2b are primarily transferred prenatally via the yolk sac pathway, whereas IgG1 is predominantly delivered postnatally through breast milk. Building on this, our results indicate that in dams subjected to 0.5 ppm O_3-induced MIA, the galactosylated IgG2a and IgG2b antibodies are predominantly transported via the yolk sac. Conversely, IgG1, which is primarily transmitted postnatally via breast milk, exhibits enrichment of pro-inflammatory GlcNAc glycosylation. This indicates that MIA reprograms the glycosylation state of IgG1, thereby further reinforces this pro-inflammatory trend. These findings are consistent with established research: elevated N-acetylglucosamine has been linked to atherosclerosis through IgG-driven pro-inflammatory enhancement. Reduced core fucosylation amplifies FcγRIII-mediated inflammation in dengue, while high-mannose IgG correlates with melanoma disease activity. This intricate regulatory network suggests a synergistic interplay between the IgG glycosylation state and the inherent subclass-specific transport pathways, thereby constitutes a temporally precise regulatory framework, collectively enabling "selective transfer" based on immunological demands." (lines 717-748)

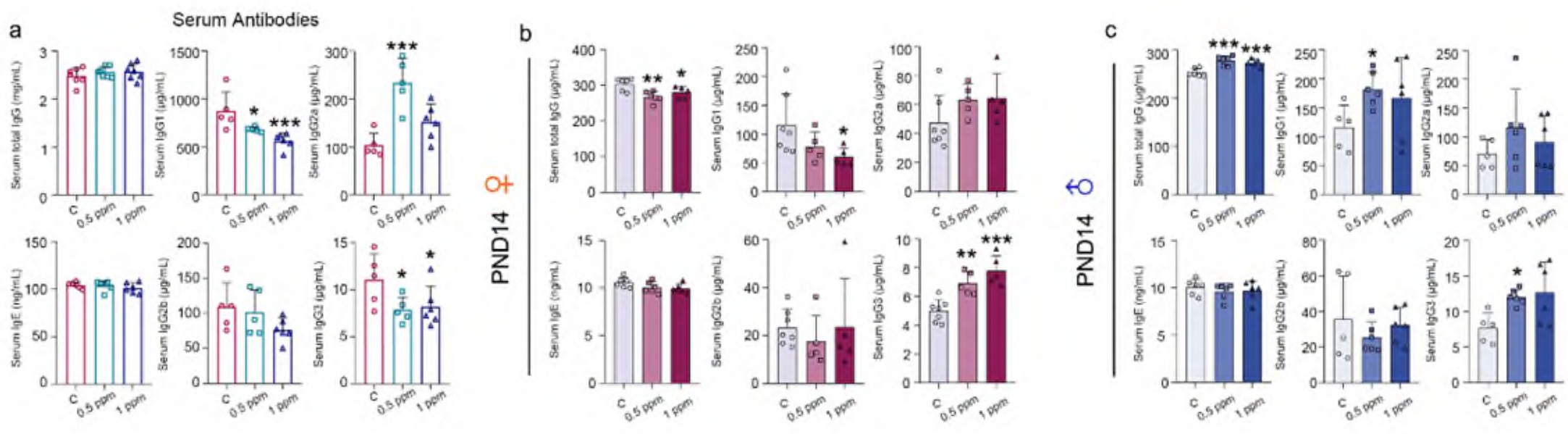

Fig. 2. Serum concentrations of total IgG and IgG subclasses (IgG1, IgG2a, IgG2b, and IgG3) in E18.5 dams **(a)**, PND14 female offspring **(b)**, and PND14 male offspring **(c)** (n = 5-6). Data were shown as the mean ± SD. Statistical analysis was performed by one-way ANOVA. *p < 0.05, **p < 0.01, and ***p < 0.001 compared with the corresponding control group.

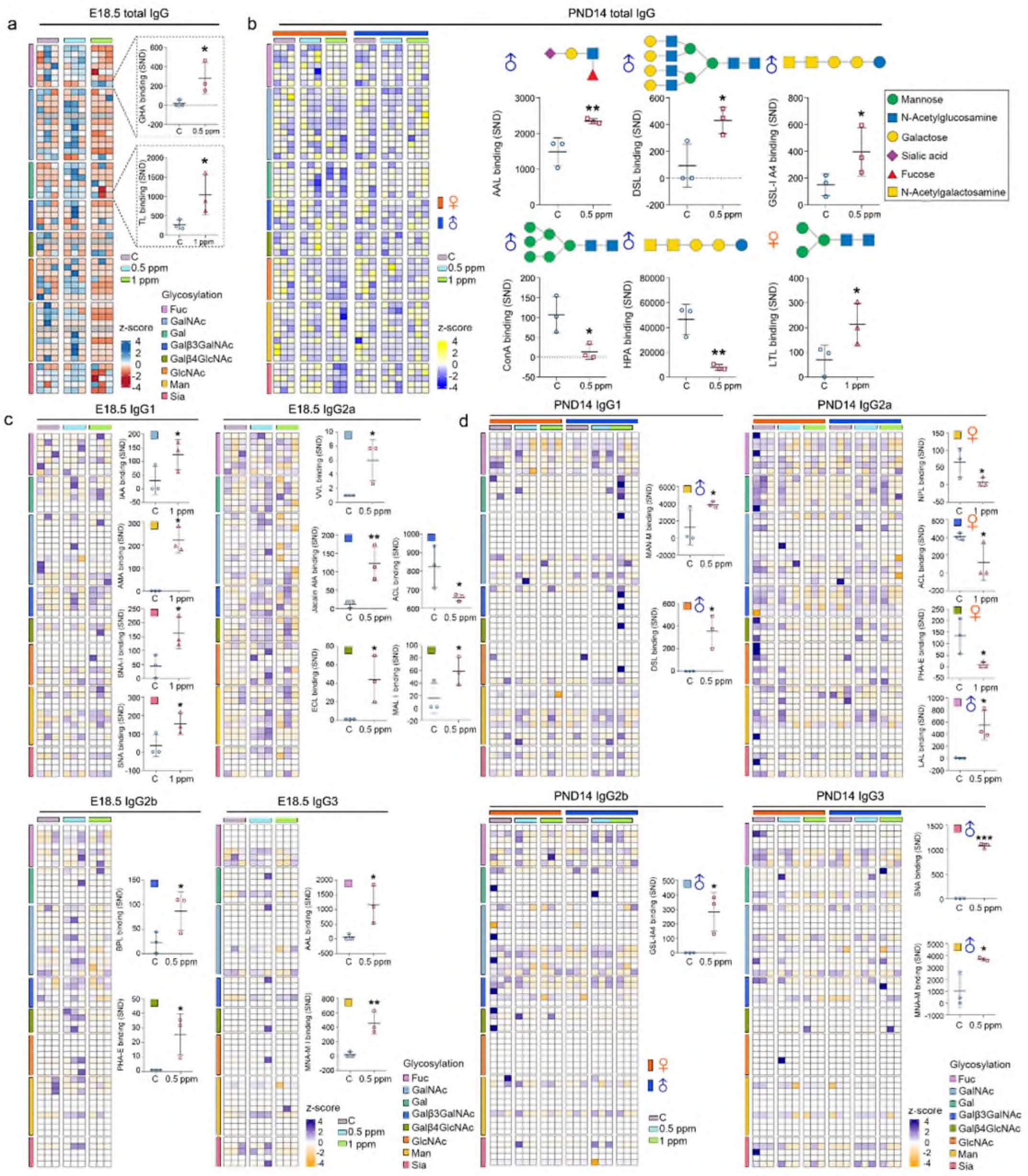

Fig. 3. IgG glycosylation profiles in maternal and offspring serum. **a** Glycosylation profile of total

IgG in maternal serum at E18.5. **b** Glycosylation profile of total IgG in serum of female and male offspring at PND14. **c** Subclass-specific glycosylation profiles (IgG1, IgG2a, IgG2b, and IgG3) in maternal serum at E18.5. **d** Subclass-specific glycosylation profiles (IgG1, IgG2a, IgG2b, and IgG3) in serum of female and male offspring at PND14. The dot plots display differential lectin binding in offspring from the 0.5 ppm O_3- and 1 ppm O_3-induced MIA groups relative to offspring from control dams (n = 3). Data were shown as the mean ± SD. Statistical analysis was performed by one-way ANOVA. *$p < 0.05$, **$p < 0.01$, and ***$p < 0.001$ compared with the corresponding control group.

Reviewer:

4. Mechanistic Validation of IgG-FcγRIII Interaction: - Issue: Molecular docking results are predictive but lack experimental confirmation. - Revision: Recommend including future plans for in vitro binding assays in the discussion.

Authors:

We sincerely appreciate the reviewers' valuable suggestions regarding experimental validation of our molecular docking results in future plans. Existing studies have robustly established the functional impact of GlcNAc modifications on enhancing IgG binding to FcγRIII in human systems. For instance, Zou et al. used chemoenzymatically synthesized IgG glycoforms and SPR analysis to demonstrate that the presence of bisecting GlcNAc strengthens affinity for FcγRIIIa, with minimal effect on the inhibitory receptor FcγRIIb[1]. Julian et al. further engineered cells to produce antibodies enriched with bisecting GlcNAc, which exhibited a 10–20-fold enhancement in antibody-dependent cellular cytotoxicity (ADCC) activity via increased FcγRIII engagement[2]. Moreover, an *in vitro* glycan remodeling platform confirmed that enzymatic introduction of bisecting GlcNAc into therapeutic antibodies increased ADCC by 10-fold, with MALDI-MS analysis directly correlating the enhanced function with the specific bisecting GlcNAc addition[3].

While these comprehensive studies strongly support our computational prediction that GlcNAc modifications enhance IgG-FcγRIII interaction, they were conducted in human antibody systems. We acknowledge the current technical limitation in experimentally validating this specific interaction in our mouse model. The key challenge lies in generating the precise bisecting GlcNAc glycoform on mouse IgG for *in vitro* binding assays. Established glycoengineering and chemoenzymatic methods are not readily applicable for installing this defined modification onto mouse IgG-Fc. This presents a specific obstacle for direct functional validation in our mouse system. To address this gap as reviewer suggested, we plan to explore and develop strategies in future work that are capable of generating mouse IgG with this specific glycosylation, which would enable definitive experimental validation of our computational models. We have included the following statement in the Discussion section (lines 840-856 of the revised manuscript): "Evidence from prior studies using surface plasmon resonance has confirmed that GlcNAc-modified IgG binds more strongly to the activating receptor FcγRIII. These findings are further corroborated by studies utilizing glycoengineered antibodies and *in vitro* glycan remodeling platforms, with MALDI-MS analysis confirming that the enhanced effector function directly correlates with bisecting GlcNAc addition. These collective observations provide strong support for our results

that Mgat4a-regulated GlcNAc glycosylated IgG exhibited a stronger binding affinity to FcγRIII compared to unmodified IgG, potentially amplifying allergy susceptibility in male adult offspring (from 0.5 ppm O_3-induced MIA). However, species-specific differences in IgG glycosylation and the current lack of methods to generate mouse IgG with the specific bisecting GlcNAc modification limit our ability to directly validate the contribution of GlcNAc-modified IgG–FcγRIII binding to allergic susceptibility in mouse models. Therefore, further research to develop species-specific glycoengineering tools is essential."

Reference:

1. Zou G, Ochiai H, Huang W, Yang Q, Li C, Wang LX. Chemoenzymatic synthesis and Fcγ receptor binding of homogeneous glycoforms of antibody Fc domain. Presence of a bisecting sugar moiety enhances the affinity of Fc to FcγIIIa receptor. J Am Chem Soc. 2011 Nov 23;133(46):18975-91.
2. Davies J, Jiang L, Pan LZ, LaBarre MJ, Anderson D, Reff M. Expression of GnTIII in a recombinant anti-CD20 CHO production cell line: Expression of antibodies with altered glycoforms leads to an increase in ADCC through higher affinity for FC gamma RIII. Biotechnol Bioeng. 2001 Aug 20;74(4):288-94.
3. Hodoniczky J, Zheng YZ, James DC. Control of recombinant monoclonal antibody effector functions by Fc N-glycan remodeling in vitro. Biotechnol Prog. 2005 Nov-Dec;21(6):1644-52.

Minor Revisions (indications and suggestion)

Reviewer:

Line 36 Mgat3-regulated IgG N-acetylglucosamine (GlcNAc) Mgat3-mediated addition of bisecting N-acetylglucosamine (GlcNAc) to IgG

Line 60 remain substantially underestimated are likely underestimated in terms of immunological impact

Lines 84–85 heightened FcγRIIIa affinity, exacerbating... increased FcγRIIIa affinity, thereby exacerbating…

Line 89 function of their immune system development and functional programming of their immune system

Line 319 shift IgG towards an anti-inflammatory function potentially confer an anti-inflammatory bias to IgG effector functions

Line 420 These results indicate that...Collectively, these findings suggest that...

Line 579 influence offspring's intrinsic IgG mediated...modulate intrinsic IgG-mediated effector functions in offspring

Authors:

We sincerely thank the reviewer for their meticulous evaluation of our manuscript and their constructive suggestions. Following the reviewer's valuable recommendations, we have carefully revised the text to improve clarity and precision as reviewer suggested.

Reviewer:

Figures and Data Quality

-Figure 1–6: Data are compelling; axis labeling in lectin microarrays should clarify

fluorescence units and glycan identity.

Authors:

We sincerely thank the reviewer for their valuable suggestion. We apologize for the lack of clarity in the representation of lectin microarray units in our original figures. Regarding the fluorescence signals were recorded from the lectin microarrays, they were acquired through the following analytical pipeline: after microarray scanning, all lectin spot signals were automatically extracted using GenePix Pro v6.0 software, which recorded the foreground signal intensity (F532, representing the median signal foreground value at 532 nm channel) and background signal intensity (B532, the median signal background value at 532 nm channel) for all lectin microarray spots in the array images. To correct for signal heterogeneity caused by background variations among different lectin spots within each array, we performed background correction by calculating the foreground-to-background difference (F – B) for each lectin spot. Furthermore, to eliminate errors from nonspecific binding, we subtracted the F – B values of blank controls from the sample F – B values. The final normalized signals were expressed as Signal-to-Noise Difference (SND), calculated as $[(F - B)_{sample} - (F - B)_{blank\ control}]$.

We have supplemented the explanation of the lectin microarray axis signal intensity in the legend of Fig. 3 in the revised manuscript (lines 420-423), stating: "SND represents the signal strength detected by the lectin microarray assay and was defined based on the lectin spot binding signal intensity, which was calculated as $[(F - B)_{sample} - (F - B)_{blank\ control}]$, where F and B denote the median foreground (532 nm) and background signal intensities, respectively." Additionally, we have improved the description in the Methods section under 'Lectin Microarray Profiling of IgG Glycosylation' of the revised manuscript (lines 1188-1197), which now reads: "The lectin microarray data were processed using GenePix Pro 6.0 to extract the foreground signal intensity (F532, representing the median signal foreground value at 532 nm channel) and background signal intensity (B532, the median signal background value at 532 nm channel) for all lectin microarray spots. For the extracted data, background correction was performed to eliminate signal non-uniformity caused by inconsistent background values among different lectin spots by calculating the foreground-to-background difference (F – B) for each lectin spot. To eliminate errors from nonspecific lectin signals, the F – B values of blank controls (control) were subtracted from the F – B values of each lectin spot, and the SND was defined based on this calculation."

Regarding the glycan identity in the lectin microarrays (Table 5 in the response to reviewers), we have provided a table in the supplementary material detailing the glycan recognition specificity of lectins in the lectin microarray, and we have added the following statement in the Methods section on lines 1201-1202 of the revised manuscript: "The glycan recognition specificity information of lectins in the lectin microarray is provided in Supplementary Table 13." Moreover, axis labeling in lectin microarrays have been clarified fluorescence units and glycan identity.

Table 5. Lectin and Glycan Identity in the Microarray.

Glycoform	Lectin	Lectin Full Name

Fuc	LTL	Lotus tetragonolobus lectin
	PSA	Pisum sativum agglutinin
	LCA	Lens Culinaris Agglutinin
	UEA I	Ulex europaeus agglutinin I
	AAL	Aleuria aurantia lectin
	LAL	Laburnum anagyroides Lectin (Gold Chain)
	TL	Tulipa sp. Lectin (Tulip)
Sia	MAA	Maackia amurensis Lectin
	SNA, EBL	Sambucus nigra lectin
	SSA	Salvia sclarea
	MAL II	Maackia amurensis lectin II
	SNA-I	Sambucus nigra (Elderberry Bark)
Man	NPL, NPA	Narcissus pseudonarcissus (daffodil) lectin
	ConA	Con A Lectin
	GNL	Galanthus nivalis (snowdrop) lectin
	HHL, AL	Hippeastrum hybrid lectin
	CALSEPA	Calystega sepiem Lectin (Hedge Bindweed Rhizomes)
	AMA	Arum maculatum Lectin (Lords and Ladies）
	MNA-M	Morniga M Lectin (black elderberry)
	VFA	Vicia fava Lectin (Fava Bean)
	VVA mannose	Vicia villosa Lectin (Hairy Vetch, Mannose Specific)
	ASA	Allium sativum Lectin (Garlic)
GlcNAc	GSL II, BSL II	Griffonia (Bandeiraea) simplicifolia lectin II
	LEL, TL	Lycopersicon esculentum lectin
	STL, PL	Solanum tuberosum lectin
	UDA	Urtica dioica Lectin (Stinging Nettle)
	PWM, PWA	Phytolacca americana Lectin (Pokeweed)
	WGA	Triticum vulgare Lectin (Wheat Germ)
	DSL	Datura stramonium lectin
GalNAc	HPA	Helix pomation Lectin (Snail)
	VVL, VVA	Vicia villosa lectin
	DBA	Dolichos biflorus agglutinin
	SBA	Glycine max Lectin (Soybean)
	PTL I, WBA I	Psophocarpus tetragonolobus lectin I
	WFA, WFL	Wisteria floribunda lectin
	CSA	Cytisus sessilifolius Lectin (Portugal Broom)
	Black bean crude	Phaseolus vulgaris sp. Lectin
	GSL-IA4	Pure Griffonia simplicifolia Lectin
	IRA	lris hybrid Lectin (Dutch Iris)
	IAA	Iberis amara Lectin
	HMA	Homarus americanus Lectin (Lobster)
Gal	GHA	Glechoma hederacea Lectin (ground ivy)
	MNA-G	Morniga G Lectin (black elderberry）
	RCA I, RCA120	Ricinus communis agglutinin I

	GSL I-B4, BSL I-B4	GSL I - isolectin B4
	EEL	Euonymus europaeus lectin
	PNA	Arachis hypogaea Lectin (Peanut)
Galβ3GalNAc	BPL	Bauhinia Purpurea Lectin
	ABA	Agaricus bisporus Lectin (Mushroom)
	Jacalin，AIA	Jacalin
	ACL, ACA	Amaranthus caudatus lectin
	MPL	Maclura pomifera lectin
Galβ4GlcNAc	PHA-L	Phaseolus vulgaris Leucoagglutinin
	PHA-E	Phaseolus vulgaris Erythroagglutinin
	ECL	Erythrina cristagalli Lectin
	MAL I	Maackia amurensis lectin I

Reviewer:

- Fig. 6d: Provide binding free energy (ΔG) values numerically in main text.

Authors:

We thank the reviewer for their suggestion and have incorporated the binding free energy (ΔG) values in the main text to beneath the corresponding interaction heatmap in Fig. 6d in the revised manuscript to provide quantitative support for our docking results. We also described as follows: "GlcNAc-modified IgG exhibited lower binding free energies (ΔG = -14.7 kcal/mol for IgG Gly1 chain D with FcγRIII; ΔG = -15.2 kcal/mol for IgG Gly2 chain D with FcγRIII) than its unmodified counterpart (ΔG = -6.3 kcal/mol for IgG chain D with FcγRIII) when interacting with FcγRIII" (lines 569-572 in the revised manuscript).

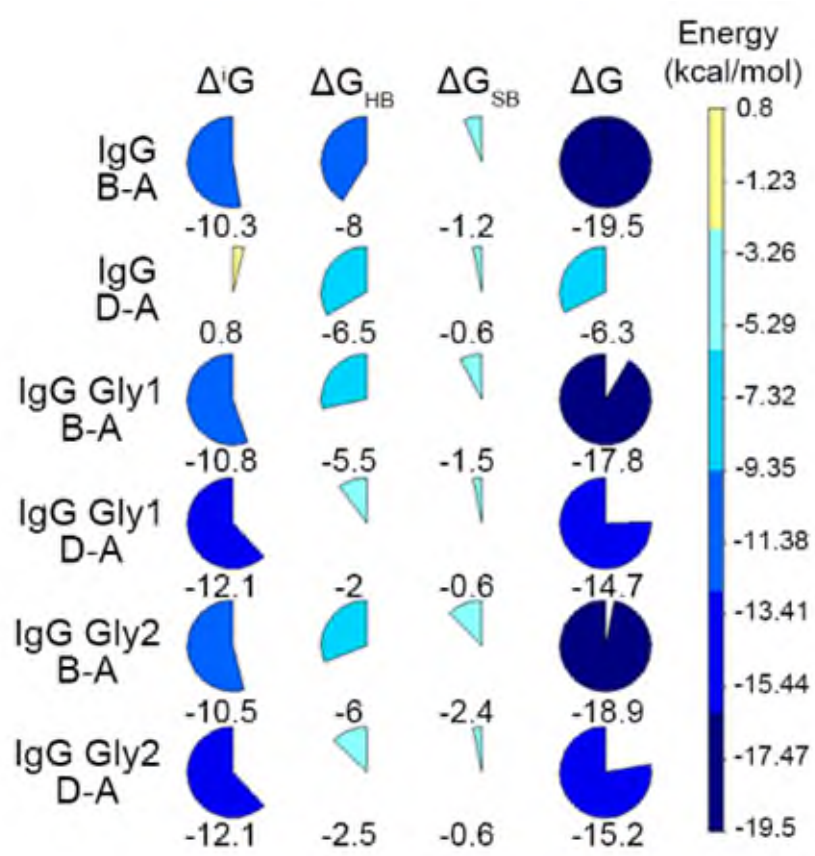

Fig. 4. Molecular docking results of unmodified and GlcNAc-modified IgG with FcγRIII using GRAMM. The heatmap displayed their binding free energies. Chain A is FcγRIII, while Chains B and D are the two heavy chains of the IgG Fc domain responsible for binding to the FcγR receptor. Yellow sectors represent positive binding free energy values, while blue sectors represent negative binding free energy values. The numerical values beneath the heatmap represent the corresponding binding free energy (ΔG) values in kcal/mol.

Reviewer:

- Supplementary Figures: Consider merging key figures (e.g., Supplementary Figs. 4,

6, and 9) into the main text.

Authors:

Thank you for the valuable suggestion. We have conducted a reassessment and reorganization of the figure layout throughout the manuscript. To optimize the length of the article, highlight the key findings, ensure the clarity of data presentation, and take into account input from other reviewers, we have now included the core result figures from the active anaphylaxis experiment in male mice in the main text, while retaining the detailed results from some female mice and the complete dataset from the passive anaphylaxis experiment in the supplementary materials.

Reviewer:

Suggested References- Martinez D. R. et al., Cell, 2022 — IgG glycoengineering and placental transfer. - Bouman A. et al., Clin Dev Immunol, 2005 — sex differences in immunity.

Authors:

We sincerely thank the reviewer for these valuable suggested references. We searched for these two references as thoroughly as possible, but could not find any articles that were completely consistent. However, we found the most relevant topics of the two authors. We have carefully read both articles and in the revised manuscript, we have incorporated the suggested citations in the Discussion section: "… This male-specific susceptibility may be partly rooted in a sexually dimorphic immune baseline, where an inherently more reactive innate immune system in males could predispose them to mount stronger responses to triggers such as maternal glycosylated IgG[1]"(lines 725-728). "…This intricate regulatory network suggests a synergistic interplay between the IgG glycosylation state and the inherent subclass-specific transport pathways, thereby constitutes a temporally precise regulatory framework, collectively enabling "selective transfer" based on immunological demands[2]"(lines 744-748).

Reference:

1. Bouman A, Schipper M, Heineman MJ, Faas MM. Gender difference in the non-specific and specific immune response in humans. Am J Reprod Immunol. 2004 Jul;52(1):19-26.
2. Martinez DR, Fong Y, Li SH, Yang F, Jennewein MF, Weiner JA, Harrell EA, Mangold JF, Goswami R, Seage GR 3rd, Alter G, Ackerman ME, Peng X, Fouda GG, Permar SR. Fc Characteristics Mediate Selective Placental Transfer of IgG in HIV-Infected Women. Cell. 2019 Jun 27;178(1):190-201.e11.

Reviewer:

Overall Assessment

This study are innovative and potentially impactful, but the causal chain between maternal O_3 exposure, IgG glycosylation, FcRn transfer, and offspring allergic phenotypes requires additional clarification or careful caveating. With the recommended revisions, this manuscript would make a valuable contribution to our understanding of how prenatal environmental insults shape immune ontogeny and allergic risk via antibody-mediated pathways.

Authors:

Thank you for your positive assessment of the innovation and potential impact of this study, as well as for your valuable suggestions regarding the mechanistic chain proposed in the manuscript. We fully agree that elucidating the causal relationships among maternal O_3 exposure, alterations in IgG glycosylation, FcRn-mediated antibody transfer, and offspring allergic phenotypes is crucial for strengthening the argument.

In response to your recommendations, we have conducted key additional experiments to provide more direct evidence. First, through *in situ* co-staining of FcRn, IgG, and glycosylation-specific lectins in yolk sac tissue at critical time windows, we have visually demonstrated their co-localization, thereby providing spatial evidence for how maternal exposure affects antibody transfer efficiency. Furthermore, we performed detailed analyses of the concentrations and glycosylation profiles of various IgG subclasses in both maternal and offspring serum. We have also made detailed modifications to the other concerns you raised. These precise modifications make the chain of evidence in this study clearer, namely that maternal O_3 exposure remodels the glycosylation patterns of antibody subclasses in the offspring, leading to their enhanced binding to FcγRIII, which further results in increased allergic susceptibility in the offspring. This has allowed us to observe more precisely the differential effects of O_3 exposure on specific antibody subpopulations and their glycosylation states, making the results more comprehensive.

We believe these supplementary analyses provide stronger experimental support for the proposed mechanistic pathway. Accordingly, we have revised the main text and discussion sections to make the argument clearer and more carefully articulated. We hope these modifications will further improve the completeness of the study. Once again, we sincerely appreciate your constructive feedback.

Reviewer 2 Comments for the Author

Reviewer:

This is an interesting studies, that addresses the impact of O_3, which is associated with an increased mortality, and focused on deciphering the role of glycosylation in regulating the immune response.

Authors:

We thank the reviewer for your helpful comments and suggestions. We are particularly encouraged that you found our study to be 'interesting'. We have carefully addressed all of the concerns raised and have incorporated the missing information in accordance with the reviewer's constructive guidance.

Response to comment

Major issues

Reviewer:

-However, ozone largely effect the organism, with beside altering glycan structures, also critically interacts with the body's iron metabolism, which further facilitate inflammation.

-It is thus essential, to include in the logistic regression hemoglobin-data at each pregnancy stage, but also in the mouse data (hgb should be included, with a hgb<11 in pregnant women considered anemic).

Authors:

We sincerely appreciate the reviewer's insightful observations regarding the multifaceted physiological impacts of O_3 exposure. We fully acknowledge that O_3 may potentially influence iron metabolism pathways, which could exacerbate inflammatory responses through mechanisms beyond glycan modification.

To thoroughly address this issue, we conducted additional analyses incorporating HGB data into our investigation. In our human cohort study, while comprehensive HGB measurements across all trimesters were unavailable due to clinical sampling limitations, we utilized available third-trimester hemoglobin data from pregnant women, which aligned with the critical exposure window identified in our research (Table 6 in the response to reviewers). We systematically included HGB data as a covariate in logistic regression models examining the association between prenatal O_3 exposure and abnormal hematological parameters, applying the World Health Organization's diagnostic threshold for gestational anemia (HGB concentration < 110 g/L) as the standard reference for our analysis[1] (Fig.5 in the response to reviewers). The comprehensive reanalysis demonstrated that even after adjusting for HGB levels, significant associations between O_3 exposure and hematological abnormalities remained robust during the mid-to-late pregnancy stages. Specifically, for each 10 $\mu g/m^3$ increase in O_3 concentration during the mid-to-late pregnancy period (T2-T3), we observed a 2.0% elevated risk of neutrophil abnormalities (95% CI: 1.001-1.040) and a 2.1% increased risk of monocyte abnormalities (95% CI: 1.000-1.041). Similarly, during the third trimester (T3), every 10 $\mu g/m^3$ O_3 increment was associated with a 1.5% higher risk of neutrophil abnormalities (95% CI: 1.003-1.028), a 2.2% increased risk of monocyte abnormalities (95% CI: 1.008-1.036), and a 3.0% greater risk of fibrinogen abnormalities (95% CI: 1.011-1.050). These additional analyses and results have now been included in the revised manuscript (lines 139-142). Importantly, these effect estimates showed remarkable consistency with our initial unadjusted models, and the HGB covariate itself did not significantly contribute to the model fit.

Furthermore, we examined the impact of gestational O_3 exposure on HGB levels in both pregnant dams and offspring in our mouse model. Comparative analysis revealed no significant differences in hemoglobin levels between O_3-exposed and control groups (Fig.6 in the response to reviewers). These consistent findings across species indicate that under our present exposure protocol, the pro-inflammatory effects of O_3 manifest predominantly through direct glycoconjugate interactions rather than significant disruption of iron metabolism. We have incorporated these key refinements throughout the manuscript, including: presenting the adjusted regression results for hemoglobin in the human cohort study along with comprehensive mouse hematological profiles in the revised Results section; and adding the following clarification in the Discussion (lines 857-880): "While the primary focus of this study is on the role of IgG glycosylation modifications in O_3 exposure-affected maternal–fetal immune transfer, we have not excluded other plausible parallel mechanisms. For instance, previous

studies have indicated that O_3 can interact with heme iron and disrupt iron homeostasis; however, adjustments in our human cohort data and assessments of hemoglobin levels in mice suggest that under our experimental conditions, dysregulation of iron metabolism is unlikely to be the main contributing factor. It has been reported that high-level O_3 exposure may theoretically disturb iron homeostasis, while at the environmentally relevant doses employed here, its inflammatory effects appear to be mediated more prominently through mechanisms such as oxidative stress and antibody glycosylation…Overall, the present study demonstrated that MIA-induced maternal IgG glycosylation reprogramming provides a complementary perspective on the mechanisms that may operate during critical windows of developmental immune transfer following prenatal exposure."

[editorial note: third party material redacted]

Fig. 5. World Health Organization (WHO)-recommended hemoglobin concentration thresholds for the diagnosis and severity assessment of anemia (2011) [World Health Organization, 2011].

Table 6. Associations of trimester-specific O_3 exposure with risks of hematological parameter abnormalities: Base model and HGB adjusted model

	Base model			HGB-adjusted model		
	OR	95% CI	*p*	OR	95% CI	*p*
**T3**						
WBC	0.990	0.978 to 1.003	0.136	0.991	0.978 to 1.004	0.172
Neu	1.016	1.004 to 1.029	0.012*	1.015	1.003 to 1.028	0.018*
Lym	0.986	0.967 to 1.004	0.127	0.985	0.966 to 1.003	0.101
Mon	1.022	1.008 to 1.035	0.002**	1.022	1.008 to 1.036	0.001**
Eos	0.979	0.853 to 1.120	0.761	0.981	0.855 to 1.121	0.781
Bas	1.005	0.936 to 1.079	0.886	1.002	0.933 to 1.076	0.953
FIB	1.030	1.010 to 1.050	0.003**	1.030	1.011 to 1.050	0.003**
**T2-T3**						
WBC	0.993	0.974 to 1.012	0.474	0.993	0.973 to 1.012	0.457
Neu	1.020	1.001 to 1.039	0.044*	1.020	1.001 to 1.040	0.040*
Lym	0.993	0.965 to 1.021	0.607	0.993	0.965 to 1.021	0.627
Mon	1.021	1.000 to 1.041	0.048*	1.021	1.000 to 1.041	0.049*

Eos	0.859	0.678 to 1.058	0.172	0.857	0.677 to 1.056	0.165
Bas	0.976	0.876 to 1.086	0.652	0.977	0.877 to 1.087	0.672
FIB	0.990	0.961 to 1.019	0.498	0.990	0.961 to 1.019	0.485

Note: * *p* < 0.05, ** *p* < 0.01

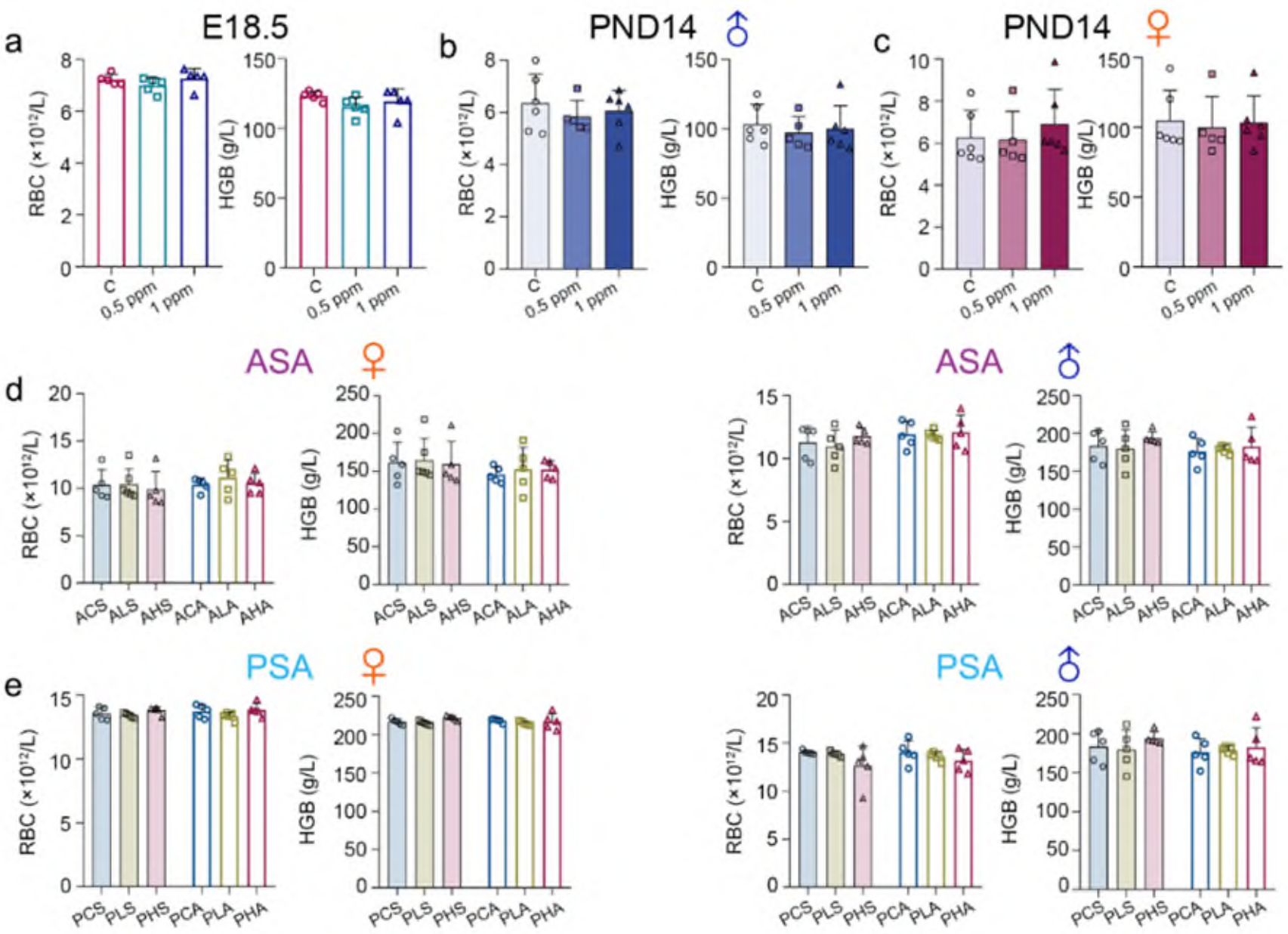

Fig. 6. Red blood cell (RBC) count and hemoglobin (HGB) concentration across experimental groups. **a** Levels in maternal mice at E18.5 (n = 6). **b** Levels in male offspring at PND14 (n = 5-6). **c** Levels in female offspring at PND14 (n = 5-6). **d** Levels in female and male mice following active anaphylaxis challenge (n = 5). **e** Levels in female and male mice following passive anaphylaxis challenge (n = 5). Data were presented as mean ± SD. Statistical analysis was performed by one-way ANOVA.

Reference:

1. World Health Organization. (2011). Haemoglobin concentrations for the diagnosis of anaemia and assessment of severity. World Health Organization. https://iris.who.int/handle/10665/85839

Reviewer:

-Further, what is really missing is an in vitro model where bisecting N-acetylglucosamine (GlcNAc) modifications in IgG glycosylation profiles are generated and are subsequently given to mice to assess their direct contribution to anaphylaxis.

Authors:

We sincerely thank the reviewer for raising this important point regarding the potential value of an *in vitro* glycoengineering and subsequent *in vivo* functional validation model. Indeed, our initial experimental design envisioned precisely such an approach; however, its implementation was hindered by the technical difficulty in generating the requisite mouse IgG glycoforms bearing the specific GlcNAc

modification. We appreciate the opportunity to discuss the existing evidence for this mechanism and the current technical challenges in implementing such a model in mouse systems.

Existing studies have robustly established the functional impact of GlcNAc modifications on enhancing IgG binding to FcγRIII in human systems. For instance, Zou et al. used chemoenzymatically synthesized IgG glycoforms and SPR analysis to demonstrate that the presence of bisecting GlcNAc strengthens affinity for FcγRIIIa, with minimal effect on the inhibitory receptor FcγRIIb[1]. Julian et al. further engineered cells to produce antibodies enriched with bisecting GlcNAc, which exhibited a 10–20-fold enhancement in ADCC activity via increased FcγRIII engagement[2]. Moreover, an *in vitro* glycan remodeling platform confirmed that enzymatic introduction of bisecting GlcNAc into therapeutic antibodies increased ADCC by 10-fold, with MALDI-MS analysis directly correlating the enhanced function with the specific bisecting GlcNAc addition[3].

While these comprehensive studies strongly support our computational prediction that GlcNAc modifications enhance FcγRIII binding, they were conducted in human antibody systems. We also attempted to generate the precise bisecting GlcNAc glycoform on mouse IgG for in vitro binding assays as suggested. However, after consulting with GLYCOGENE Pharmaceutical Co., Ltd. (Wuhan, China; specializes in antibody glycosylation modification), GlycanLink Biomedicine Co., Ltd. (Shanghai, China), and glycochemistry expert Prof. Feng Tang from the Shanghai Institute of Materia Medica (Shanghai, China), it was concluded that, established glycoengineering and chemoenzymatic methods are not readily applicable for installing this defined modification onto mouse IgG-Fc. We acknowledge the current technical limitation in experimentally validating this specific interaction in our mouse model. The key challenge lies in generating the precise bisecting GlcNAc glycoform on mouse IgG for *in vitro* binding assays. This presents a specific obstacle for direct functional validation in our mouse system. To address this gap as the reviewer suggested, we plan to explore and develop strategies in future work that are capable of generating mouse IgG with this specific glycosylation, which would enable definitive experimental validation of our computational models.

We have included the following statement in the Discussion section (lines 840-856 of the revised manuscript): "Evidence from prior studies using surface plasmon resonance has confirmed that GlcNAc-modified IgG binds more strongly to the activating receptor FcγRIII. These findings are further corroborated by studies utilizing glycoengineered antibodies and in vitro glycan remodeling platforms, with MALDI-MS analysis confirming that the enhanced effector function directly correlates with bisecting GlcNAc addition. These collective observations provide strong support for our results that Mgat4a-regulated GlcNAc glycosylated IgG exhibited a stronger binding affinity to FcγRIII compared to unmodified IgG, potentially amplifying allergy susceptibility in male adult offspring (from 0.5 ppm O_3-induced MIA). Interestingly, this GlcNAc pattern matched neonatal IgG upon maternal transfer completion (PND14), emphasizing a critical feedback loop for postnatal immune homeostasis. However, species-specific differences in IgG glycosylation and the current lack of methods to

generate mouse IgG with the specific bisecting GlcNAc modification limit our ability to directly validate the contribution of GlcNAc-modified IgG–FcγRIII binding to allergic susceptibility in mouse models. Therefore, further research to develop species-specific glycoengineering tools is essential."

Reference:

1. Zou G, Ochiai H, Huang W, Yang Q, Li C, Wang LX. Chemoenzymatic synthesis and Fcγ receptor binding of homogeneous glycoforms of antibody Fc domain. Presence of a bisecting sugar moiety enhances the affinity of Fc to FcγIIIa receptor. J Am Chem Soc. 2011 Nov 23;133(46):18975-91.
2. Davies J, Jiang L, Pan LZ, LaBarre MJ, Anderson D, Reff M. Expression of GnTIII in a recombinant anti-CD20 CHO production cell line: Expression of antibodies with altered glycoforms leads to an increase in ADCC through higher affinity for FC gamma RIII. Biotechnol Bioeng. 2001 Aug 20;74(4):288-94.
3. Hodoniczky J, Zheng YZ, James DC. Control of recombinant monoclonal antibody effector functions by Fc N-glycan remodeling in vitro. Biotechnol Prog. 2005 Nov-Dec;21(6):1644-52.

Reviewer:

-Several studies have consistently shown that male mice tend to develop greater ozone-induced AHR compared to female mice. Consequently, elevated maternal IgG cannot be really linked to the observed greater susceptibility.

-please rephrase, discuss

Authors:

We fully agree with the reviewer's observation that some studies have demonstrated male mice exhibit more pronounced O_3-induced airway hyperresponsiveness (AHR) compared to females[1-3].

The heightened allergic susceptibility in male offspring specifically correlates with enhanced FcγRIII binding affinity mediated by N-acetylglucosamine glycosylation of IgG, a modification detectable as early as postnatal day 14 (PND14) when maternal antibody transfer concludes. While maternal IgG levels remained unchanged across groups, the glycosylation profile - particularly increased FcγRIII-binding glycans - appears to drive this transgenerational effect. The transgenerational effect resulted in sexually dimorphic IgG glycan profiles in the offspring, which in turn led to differential allergic susceptibility. This epigenetic programming of offspring immunity through qualitative changes in maternal IgG represents a novel mechanism distinct from the direct O_3-induced AHR pathway in adults. Furthermore, AHR is a core manifestation and primary consequence of IgE-mediated allergic responses. However, in our established allergy model, we measured the levels of allergen-specific IgE and found no significant differences among groups (Fig.7 in the response to reviewers)**.** Our findings thus reveal an alternative axis of environmental exposure impacts, where maternal immune experience reshapes offspring allergic responses through antibody glycosylation.

We have added the following statement to the Discussion section (lines 786-814) to further clarify this point: "In the present study, male offspring from 0.5 ppm O_3-induced MIA exhibited pronounced hypothermia, elevated anti-BSA IgG, increased IL-

6/CXCL1, and decreased IL-5, validating that neonatal IgG glycosylation alterations and inflammatory dysregulation induced by maternal oxidative stress during MIA drive abnormalities in offspring…We have demonstrated that male offspring from O_3-exposed dams (the ALA group) exhibit a significant increase in neutrophils—key responder cells in allergic reactions—accompanied by markedly altered FcγRIII expression levels on these cells, findings that directly corroborate this antibody-myeloid receptor interaction mechanism. For females, these differences are less pronounced than in males…These findings suggest that differentially glycosylated IgG transferred from maternal to the male or female offspring contribute to different allergic reactions later in life, particularly upon allergen exposure."

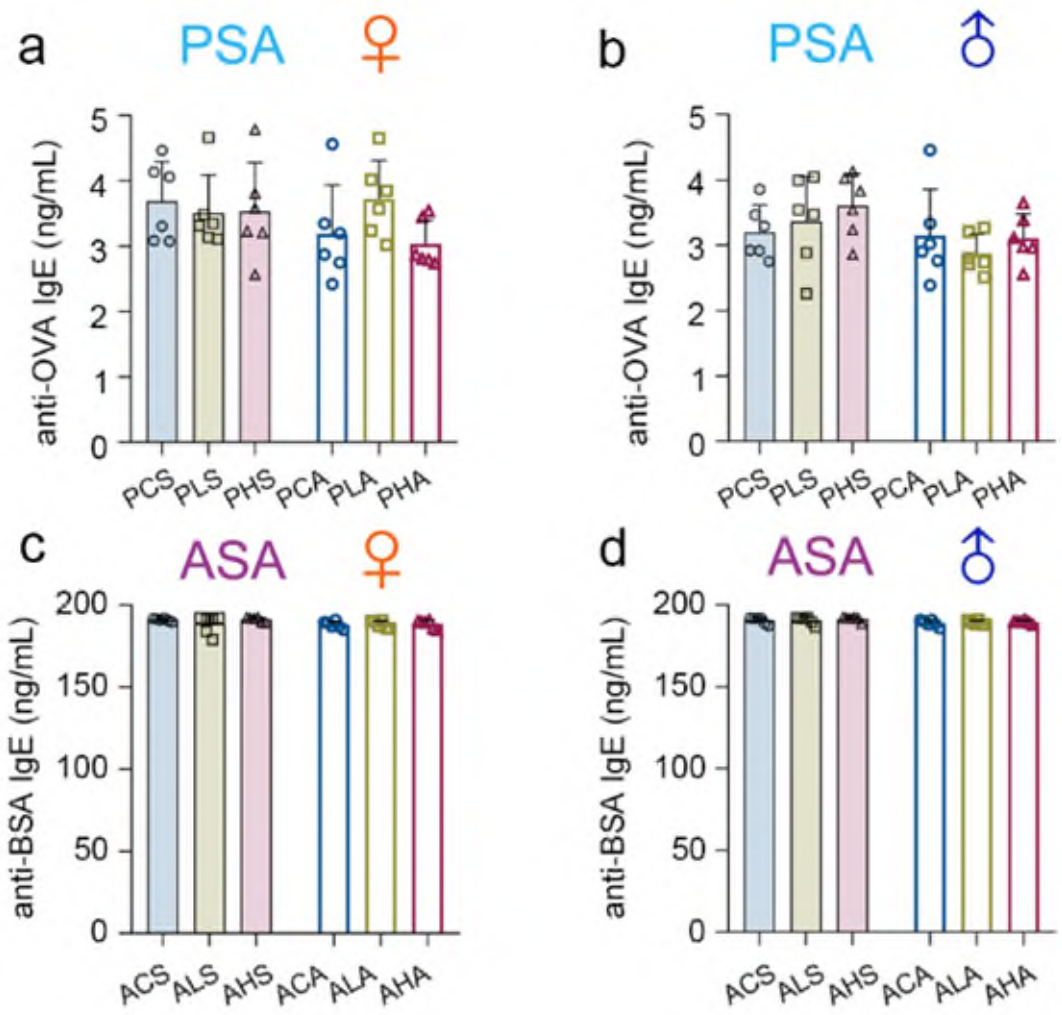

Fig. 7. Allergen-specific IgE levels in active and passive anaphylaxis models. **a** Anti-ovalbumin (OVA) IgE levels in female mice subjected to passive systemic anaphylaxis (PSA) (n = 6). **b** Anti-OVA IgE levels in male mice subjected to PSA (n = 6). **c** Anti-bovine serum albumin (BSA) IgE levels in female mice subjected to active systemic anaphylaxis (ASA) (n = 6). **d** Anti-BSA IgE levels in male mice subjected to ASA (n = 6). Data were presented as mean ± SD. Statistical analysis was performed by one-way ANOVA.

Reference:

1. Cho Y, Abu-Ali G, Tashiro H, Brown TA, Osgood RS, Kasahara DI, Huttenhower C, Shore SA. Sex Differences in Pulmonary Responses to Ozone in Mice. Role of the Microbiome. Am J Respir Cell Mol Biol. 2019 Feb;60(2):198-208.
2. Brown TA, Tashiro H, Kasahara DI, Cho Y, Shore SA. Early life microbiome perturbation alters pulmonary responses to ozone in male mice. Physiol Rep. 2020 Jan;8(2): e14290.
3. Birukova A, Cyphert-Daly J, Cumming RI, Yu YR, Gowdy KM, Que LG, Tighe RM. Sex Modifies Acute Ozone-Mediated Airway Physiologic Responses. Toxicol Sci. 2019 Jun 1;169(2):499-510.

Reviewer:

-Differences in Th1/Th2 profile, please provide specific IgE data.

Authors:

Thank you for your thoughtful comment. We have now included the specific IgE

data in the Results section of the revised manuscript. We found no significant difference in IgE levels between the control and exposure groups (Fig.8 in the response to reviewers).

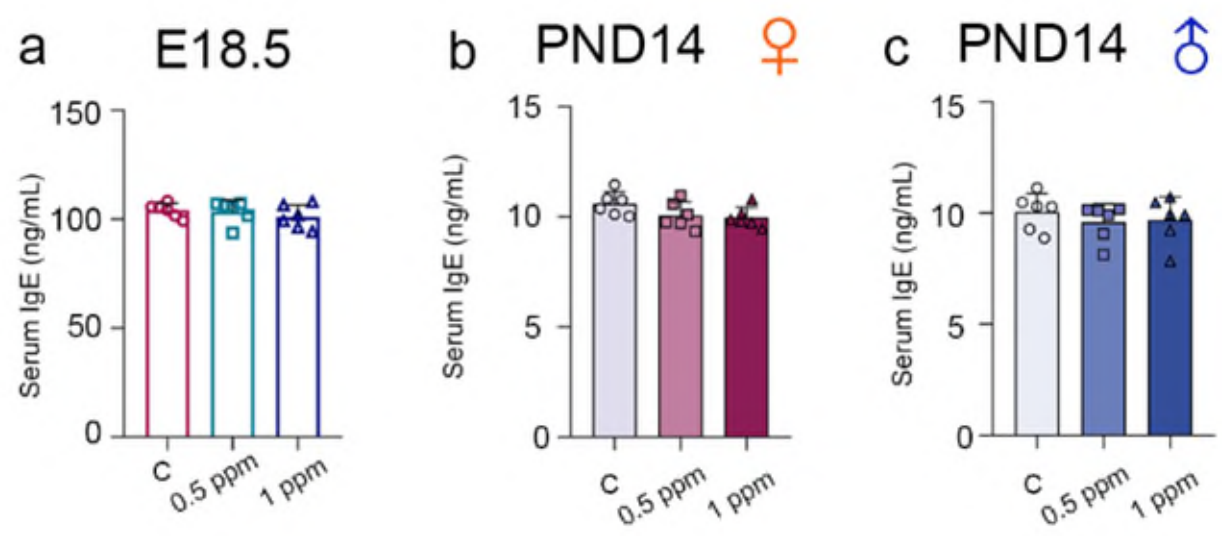

Fig. 8. Serum IgE concentrations in maternal and offspring mice. **a** Total IgE levels in maternal serum at E18.5 (n = 6). **b** Total IgE levels in serum of female offspring at PND14 (n = 6). **c** Total IgE levels in serum of male offspring at PND14 (n = 6). Data were presented as mean ± SD. Statistical analysis was performed by one-way ANOVA.

Reviewer:

-All graphs should also depict single values as points.

Authors:

We thank the reviewer for this careful suggestion. All graphs have been revised to depict individual data points in accordance with this recommendation.

Reviewer:

Figure 1:

-difference between f and g (y-axis is wrong, as it probably should mean spleen).

-Please show in every graphical presentations single points.

-Also as less Th2 cells are seen and less Ag-presentation is suggested, please show antigen-specific IgE-levels from the different mice groups.

-Please detail better the schematic the immunization/exposure strategy of ASA/PSA and extra panel detailing the sensitization model and in bigger letters would be welcomed

-Please write in the figure legend, which statististical test were employed in which graph.

Authors:

- We thank the reviewer for pointing out this error. We apologize for the oversight and have corrected the y-axis label in Fig.1g to accurately represent the number of T-cell subpopulations in the spleen.

- As requested, all graphical presentations now display individual data points.

- Thank you for your comment. In response, we have measured and included the antigen-specific IgE levels from the different mouse groups in the Results section of the revised manuscript. The results indicate no significant differences in antigen-specific IgE levels were observed among the groups (Fig.7 in the response to reviewers).

- Thank you for your suggestion regarding improving the schematic. In the revised manuscript, we have redrawn the figure to more clearly and illustrate the immunization/exposure strategy in detail for both active systemic anaphylaxis (ASA)

and passive systemic anaphylaxis (PSA) experiments. Additionally, we have increased the font size of all labels and descriptive text to ensure optimal readability at various sizes (Fig.9 in the response to reviewers). The updated schematic (Fig. 1) and its corresponding legend have been placed in the appropriate section of the manuscript.

- Thank you for pointing this out. In the revised manuscript, we have explicitly stated the specific statistical tests employed in the legend of every figure that presents comparative data.

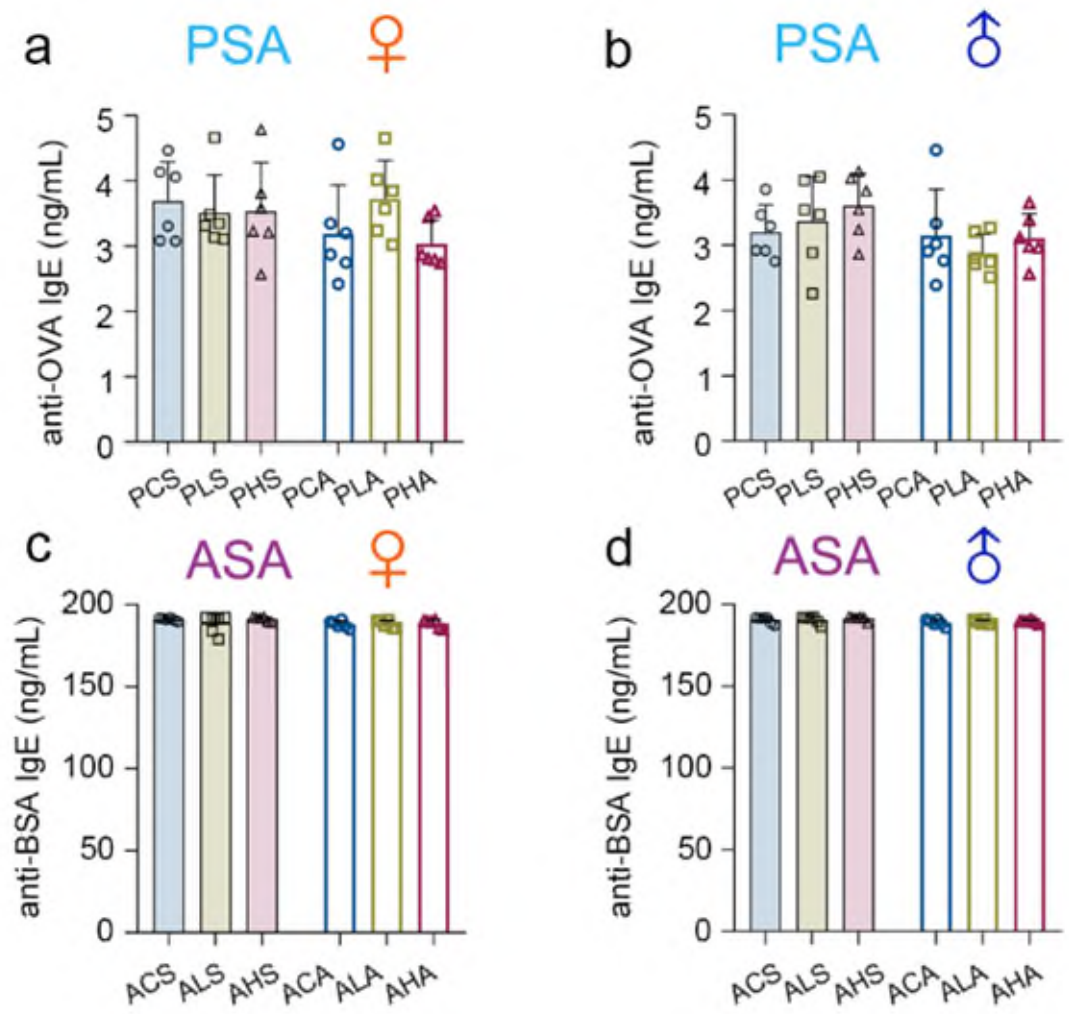

Fig. 7. Allergen-specific IgE levels in active and passive anaphylaxis models. **a** Anti-ovalbumin (OVA) IgE levels in female mice subjected to passive systemic anaphylaxis (PSA) (n = 6). **b** Anti-OVA IgE levels in male mice subjected to PSA (n = 6). **c** Anti-bovine serum albumin (BSA) IgE levels in female mice subjected to active systemic anaphylaxis (ASA) (n = 6). **d** Anti-BSA IgE levels in male mice subjected to ASA (n = 6). Data were presented as mean ± SD. Statistical analysis was performed by one-way ANOVA.

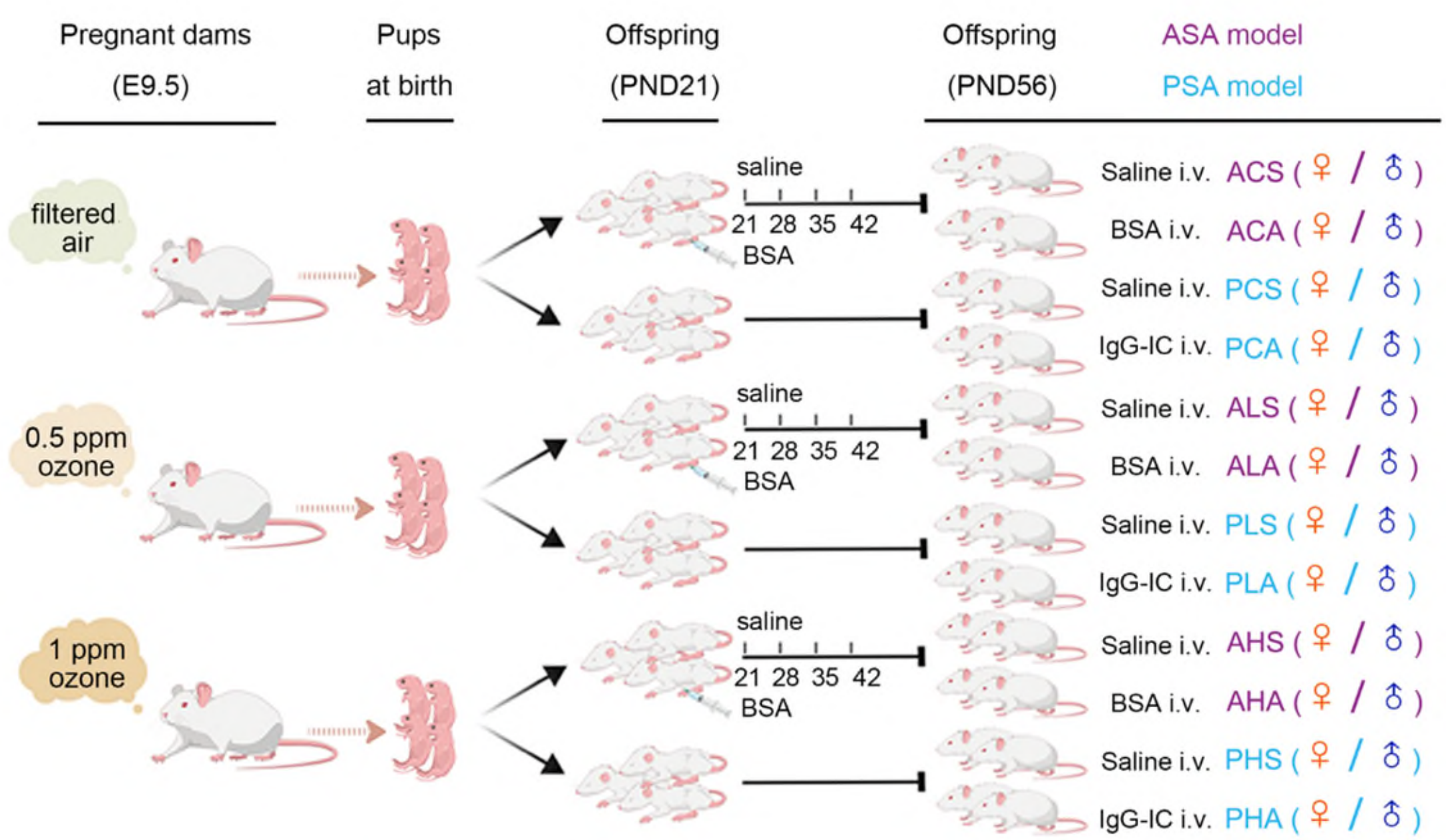

Fig. 9. Experimental design of MIA during gestation and offspring allergy models. Pregnant dams were exposed to filtered air (labeled as control (C) group) or O3 (0.5 ppm and 1 ppm) for 4 hours daily from gestational day 9.5 (E9.5) until parturition. Offspring were subjected to active systemic anaphylaxis (ASA) or passive systemic anaphylaxis (PSA) models. For ASA model, offspring received weekly intraperitoneal injections of bovine serum albumin (BSA) from postnatal day 21 (PND21) to PND42, followed by an intravenous (i.v.) BSA challenge at PND56. Experimental groups were designated as ACA (offspring from air-exposed dams + BSA), ALA (offspring from 0.5 ppm O_3-exposed dams + BSA), and AHA (offspring from 1 ppm O_3-exposed dams + BSA), with corresponding saline-treated controls (ACS, ALS, and AHS). For PSA model, offspring were intravenously (i.v.) injected with pre-formed ovalbumin-IgG immune complexes (IgG-IC) at PND56. Groups were labeled PCA (offspring from air-exposed dams + IgG-IC), PLA (offspring from 0.5 ppm O_3-exposed dams + IgG-IC), and PHA (offspring from 1 ppm O_3-exposed dams + IgG-IC), with corresponding saline controls (PCS, PLS, and PHS). Sex-specific analyses were labeled as female (♀) and male (♂). [Editorial note: The elements in this figure were Created with BioGDP.com. Jiang S, Li H, Zhang L, et al. Generic Diagramming Platform (GDP): a comprehensive database of high-quality biomedical graphics. Nucleic Acids Res. 2025;53(D1):D1670-D1676. doi:10.1093/nar/gkae973]

Reviewer:

Figure 2:

-RBC-values, hemoglobin-values per group and sex, RBC count of the different pups should be added.

-Please make the graphical presentation of b as in d and only of those cytokines that are significant, as here better visual comparison can be made.

-Please elaborate the results in regard to O_3 exposure, rather than male/female.

-No difference can be seen in lung inflammation between ALA and AHA in regard to H&E staining- please explain in the result section.

-How was the -T-SNE anaylsis conducted, which perplexity and step size was used?

-Please interpret the two B cell cluster in AHS.

Authors:

- We have supplemented the red blood cell (RBC) counts and hemoglobin values for each group, as well as the RBC counts of the different pups, in the revised manuscript (lines 313).

- Thank you for your suggestion. In order to comprehensively present the data and provide a clearer visual comparison of the statistically significant changes in cytokines, we have now supplemented the manuscript by adding bar graphs specifically for these cytokines in the supplementary materials.

- Thank you for your valuable comment. In the revised manuscript, we have reorganized the description of this section's results to center on the levels of prenatal O_3 exposure (i.e., comparisons among the ACA, ALA, and AHA groups). For any statistically significant sex differences identified, we have integrated them as supplementary explanations to the main effects of O_3.

- We acknowledge the reviewer's observation. While direct visual assessment of H&E-stained lung sections may not readily distinguish subtle inflammatory differences

between the ALA and AHA groups, our quantitative flow cytometry analysis revealed statistically significant variations in specific inflammatory cell populations, such as neutrophils. To improve clarity, we have replaced the H&E images in Fig. 2 with more representative micrographs, where arrows now clearly indicate inflammatory foci for easier comparison. Together with the complementary flow cytometry data, this updated presentation provides a comprehensive assessment of lung inflammation.
- We thank the reviewer for raising this question. In FlowJo, we selected the parameters for the t-SNE analysis based on the characteristics of our specific dataset (which contains approximately 100,000 cells) and computational practices for large-scale cytometry data. These parameters were configured to accurately capture the high-dimensional local relationships among immune cell subsets while also generating a stable, two-dimensional embedding. For this analysis, we employed the opt-SNE method, an optimized implementation in FlowJo based on the approach by Belkina et al., which intelligently manages the optimization process. We set the perplexity to 30. This value is suitable for our large-scale dataset; a higher perplexity emphasizes broader global data structure, whereas a lower value focuses on finer local neighborhoods. A setting of 30 provided a balance, effectively revealing the major immune populations without over-segmenting the data or over-smoaning meaningful biological differences. The learning rate (eta) was set to 7000, a value automatically suggested by the opt-SNE configuration based on our dataset size and perplexity. While this value is higher than the typical defaults for smaller datasets, it is appropriate and recommended for large cell counts to ensure efficient and effective convergence of the gradient descent algorithm within a manageable number of iterations (here, 1000). This combination, along with the use of the exact KNN algorithm (vantage point tree) and the Barnes-Hut gradient algorithm, was selected to ensure computational robustness and reproducibility.
- Thank you for your question regarding the two B cell clusters observed in the AHS sample. We appreciate the opportunity to clarify this observation. In our t-SNE analysis, the B cell population was pre-gated using the classical surface marker combination CD45+CD11b-I-A/I-E+, a standard strategy to identify the total B cell compartment. The visualization itself, however, is based on a high-dimensional space incorporating all measured parameters from our panel. While most samples showed a more continuous or loosely aggregated distribution for this gated population, the AHS sample presented a distinct spatial separation into two primary clusters within the t-SNE map. This bipartite distribution likely reflects underlying biological heterogeneity within the total B cell pool that is captured by the multidimensional data, even without dedicated subpopulation markers. The most plausible explanation is a divergence in activation or differentiation states. One cluster may correspond to a more naïve or resting B cell phenotype, while the other could represent a continuum of activated, memory, or early antibody-secreting cells. These states are known to differ in metrics like cell size, granularity, and the expression levels of various surface proteins, which would influence their positions in the t-SNE plot. The algorithm is sensitive to these subtle, multidimensional differences and can resolve them into visually distinct communities. It is important to note that we did not include specific markers for definitive B cell subset classification (e.g., CD19, CD27, CD38) in this particular panel. Therefore,

while the two-cluster structure suggests significant functional or developmental heterogeneity within the B cells of the AHS sample, we cannot ascribe definitive subset identities to them based on the current data. This intriguing pattern itself may indicate a unique property of the B cell response under the specific experimental conditions of the AHS sample. Future studies incorporating extended phenotyping panels would be valuable to precisely characterize these distinct clusters.

Reviewer:

Figure 3:

-Please indicate in each graph, whether this is maternal IgG or from the pups, please show single points, if a pool was measured, please indicate in text and figure legend.

-Add hemoglobin-levels of mice exposed to saline/0.5 and 1 ppm O_3.

-Please show sugar-binding not only by gender, but also by O_3 exposure.

Authors:

- We apologize for the inadequate presentation in our original figures. We have now revised relevant graphs to explicitly indicate the source of IgG (maternal or pup-derived) and have presented all data using individual data points. These necessary clarifications have been added to the figures and their corresponding legends in the revised manuscript.

- We have now included the hemoglobin levels of mice in the revised manuscript. HGB and RBC levels in pregnant dams exposed to control, 0.5 ppm, and 1 ppm O_3 have been included in Fig. 1c. The corresponding levels in offspring at PND14 are provided in Fig. 4a (males) and Supplementary Figure 15 (females). Furthermore, HGB levels measured in the active and passive anaphylaxis models are shown in Figure 2c (active and anaphylaxis models) and Supplementary Fig. 7 (passive anaphylaxis models). These data collectively demonstrate that the prenatal O_3 exposure at these concentrations did not induce significant alterations in systemic HGB levels.

- We greatly appreciate your insightful comments. In the Fig. 3 f-k of the revised manuscript, we used squares of different colors to indicate differences in O_3 exposure (i.e., C, 0.5 ppm, and 1 ppm), which is also shown in the figure labels, as shown in the figure below (Fig.10 in the response to reviewers).

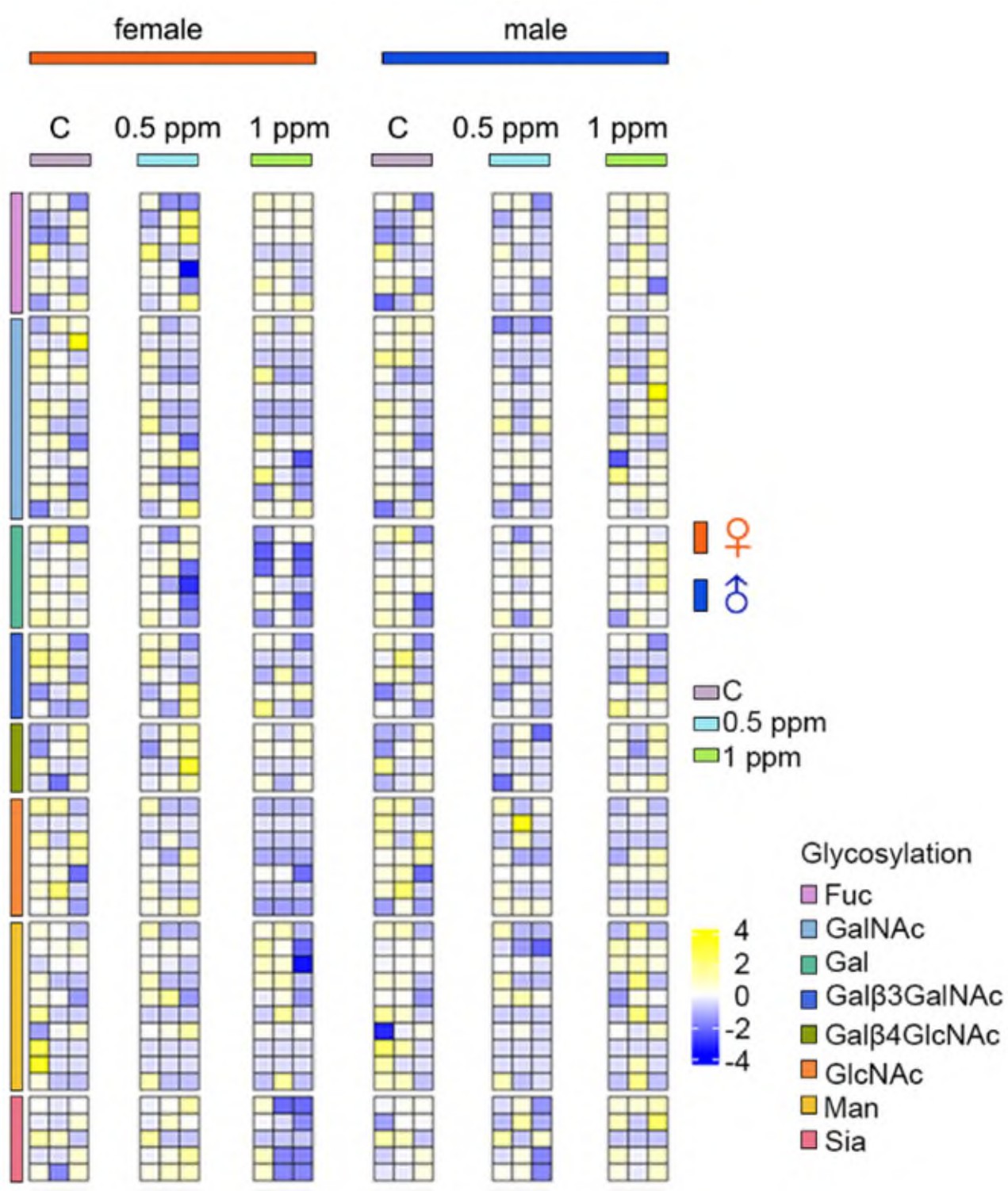

Fig. 10. IgG glycosylation profiles in male/female offspring serum at PND14. The squares of different colors to indicate differences in O_3 exposure (i.e., C, 0.5 ppm, and 1 ppm).

Reviewer:

Figure 4:

-Please add hb-values per O_3 exposure.

-Please show individual points in all graphs.

-Why is the 1ppm O_3 missing now in figure 4e, h.

Authors:

- We thank the reviewer for this comment. According to the comments, we have supplemented the hemoglobin (Hb) values for each group in the Fig. 4a of the revised manuscript.

- We have now included individual data points in all graphs throughout the manuscript.

- We sincerely appreciate the reviewer's careful attention to this detail. The transcriptomic analysis (RNA-seq) was specifically performed on lung tissues from male offspring in the 0.5 ppm O_3-induced MIA group because our lectin microarray data revealed that maternally derived IgG underwent significant glycosylation alterations specifically at this exposure level, which is the main finding of our current research. We hypothesize that pro- and anti-inflammatory glycosylated IgG provided by the dam work in concert to shape the immune priming phenotypes in offspring. In contrast, the 1.0 ppm O_3-induced MIA group, which did not exhibit significant glycan changes. To further elucidate and focus on the genetic regulation underlying IgG glycosylation-mediated immune priming effects, we analyzed lung transcriptomes from PND14 male offspring (from 0.5 ppm O_3-induced MIA). We have clarified this research focus in the revised Results section (lines 443-446).

Reviewer:
Figure 6:
-While the different glycosylation surely has an impact, enhanced allergic reaction is not shown in any experiments, this should be shown using in vitro generated antibodies and monitoring for anaphylaxis, otherwise this is only speculation. However, inflammation seems to be facilitated.
Authors:

We appreciate the reviewer's comments. In response to the reviewer's comment, we have further clarified and strengthened the existing evidence in our manuscript regarding enhanced allergic reactions. As supported by previous studies, IgG-mediated anaphylaxis in mice is characterized by several clinical manifestations including reduced core body temperature, vasodilation, increased vascular permeability, decreased motor activity, and impaired consciousness[1, 2]. The measurement of core body temperature changes has been well established as a reliable indicator of anaphylaxis severity in multiple studies[3-7]. The Results section (revised manuscript, lines 197-216) presents our findings: "In the ASA model, core body temperature measurements confirmed the successful establishment of allergic reaction models, as antigen-challenged offspring exhibited significantly greater temperature reductions compared to their respective solvent controls, regardless of maternal air or O_3 exposure (ACA vs. ACS, ALA vs. ALS, AHA vs. AHS). Female offspring from the ALA group developed severe hypothermia within 15 minutes post-challenge, with a temperature drop 1.33-fold greater than that observed in the ACA group. Notably, in male offspring, prolonged hypothermia persisted in both ALA and AHA groups for 75 minutes post-challenge, with a maximum core body temperature decline of 6.27°C in the ALA group, which failed to recover even after 105 minutes (Fig. 1l in the revised manuscript)." Additionally, we measured antigen-specific IgG levels and found: "Consistently, anti-BSA IgG levels in male offspring from the ALA and AHA groups were elevated 2.63-fold and 1.97-fold, respectively, compared to the ACA group (Fig. 1m in the revised manuscript). Similarly, following intravenous administration of an identical dose of OVA-IgG immune complexes, post-metabolic circulating anti-OVA IgG levels measured post-injection were 1.91-fold higher in the PLA group than in the PCA group (Fig. 1o, p in the revised manuscript), suggesting altered IgG-IC pharmacokinetics in these offspring, this might result from the reprogramming of immune effector functions during embryonic development. In contrast, levels of anti-BSA IgE and anti-OVA IgE showed no significant differences among the groups (Fig. 1n, q in the revised manuscript)". These results indicated that maternal alterations led to enhanced allergic reaction in offspring.

FcγRIII serves as the primary activating receptor in IgG-mediated allergic responses, enabling immune cells to engage IgG immune complexes (ICs) and initiate downstream pro-inflammatory signaling. To assess whether IgG-IC accumulation in the lungs was dependent on FcγR-mediated immune cell activation, we examined FcγRIII/FcγRIIB expression levels. Compared to allergen-naive offspring, allergen-challenged mice exhibited a marked reduction in FcγRIII/FcγRIIB^{+} neutrophils,

alveolar macrophages, interstitial macrophages, and eosinophils in lung tissue (Fig. 2k and Supplementary Fig. 11 in the revised manuscript). Notably, the downregulation of FcγRIII on neutrophils serves as a sensitive indicator of receptor engagement and allergic severity. Upon allergen challenge, FcγRIII/FcγRIIB expression on neutrophils in male offspring from MIA-exposed dams was significantly reduced compared to controls, with the ratio declining to 58% of ACA group levels in the ALA group (Fig. 2j, k in the revised manuscript). Subsequent glycosylation profiling and transcriptomic analysis indicated that the GlcNAc modification enhances the binding of IgG to FcγRIII. This increased engagement likely leads to enhanced receptor internalization and degradation, thereby reducing surface FcγRIII/FcγRIIB expression on effector cells such as neutrophils.

However, as noted in our response to previous comment and acknowledged as a study limitation in the manuscript, species-specific differences in IgG glycosylation and the current lack of convinced methods to generate mouse IgG with the specific bisecting GlcNAc modification limit our ability to directly validate the contribution of GlcNAc-modified IgG–FcγRIII binding to allergic susceptibility in mouse models through *in vitro* approaches.

Reference:

1. Godon O, Hechler B, Jönsson F. The role of IgG subclasses and platelets in experimental anaphylaxis. J Allergy Clin Immunol. 2021 Apr;147(4):1209-1211.
2. Miyajima I, Dombrowicz D, Martin TR, Ravetch JV, Kinet JP, Galli SJ. Systemic anaphylaxis in the mouse can be mediated largely through IgG1 and Fc gammaRIII. Assessment of the cardiopulmonary changes, mast cell degranulation, and death associated with active or IgE- or IgG1-dependent passive anaphylaxis. J Clin Invest. 1997 Mar 1;99(5):901-14.
3. Beutier H, Gillis CM, Iannascoli B, Godon O, England P, Sibilano R, Reber LL, Galli SJ, Cragg MS, Van Rooijen N, Mancardi DA, Bruhns P, Jönsson F. IgG subclasses determine pathways of anaphylaxis in mice. J Allergy Clin Immunol. 2017 Jan;139(1):269-280.e7.
4. Khodoun MV, Kucuk ZY, Strait RT, Krishnamurthy D, Janek K, Clay CD, Morris SC, Finkelman FD. Rapid desensitization of mice with anti-FcγRIIb/FcγRIII mAb safely prevents IgG-mediated anaphylaxis. J Allergy Clin Immunol. 2013 Dec;132(6):1375-87.
5. Epp A, Hobusch J, Bartsch YC, Petry J, Lilienthal GM, Koeleman CAM, Eschweiler S, Möbs C, Hall A, Morris SC, Braumann D, Engellenner C, Bitterling J, Rahmöller J, Leliavski A, Thurmann R, Collin M, Moremen KW, Strait RT, Blanchard V, Petersen A, Gemoll T, Habermann JK, Petersen F, Nandy A, Kahlert H, Hertl M, Wuhrer M, Pfützner W, Jappe U, Finkelman FD, Ehlers M. Sialylation of IgG antibodies inhibits IgG-mediated allergic reactions. J Allergy Clin Immunol. 2018 Jan;141(1):399-402.e8.
6. Strait RT, Morris SC, Yang M, Qu XW, Finkelman FD. Pathways of anaphylaxis in the mouse. J Allergy Clin Immunol. 2002 Apr;109(4):658-68.
7. Shade KC, Conroy ME, Washburn N, Kitaoka M, Huynh DJ, Laprise E, Patil SU, Shreffler WG, Anthony RM. Sialylation of immunoglobulin E is a determinant of allergic pathogenicity. Nature. 2020 Jun;582(7811):265-270.

Reviewer:

Minor

-Please explain the abbreviation, if possible write the whole word and do not use abbrevitations.

-Statistical test employed should be stated in the figure legends and shown in the figure with * for $p<0.05$, ** $p<0.01$ and so on, but the specific p-Values between the different groups should not be in the figure legend.

Authors:

We thank the reviewer for these constructive suggestions regarding the clarity and presentation of our data.

- We have now expanded all relevant abbreviations in the figure legends. Full terms are provided upon their first appearance in the respective legend.

- Regarding the statistical presentation, we have revised all figure legends to explicitly state the specific statistical test used for the comparisons shown in each panel. Furthermore, we have removed any specific p-values from the legends and have consistently used asterisks within the figures themselves to denote statistical significance.

Reviewer 3 Comments for the Author

Reviewer:

The manuscript entitled "Elevated ozone activates maternal immunity to drive offspring allergy through IgG glycosylation reprogramming" aimed to rely prenatal oxidative stress during pregnancy induced by exposure to ozone, to offspring immunity – notably propensity to develop allergy. The authors postulate the effects resulted from modification of IgG glycosylation in early life. While timely and of scientific interest, the study presented many issues and impreciseness that doesn't support its acceptance.

Authors:

We thank you for your constructive feedback and valuable insights on our manuscript. We have carefully considered all points raised by editor and reviewers, and have revised the manuscript thoroughly to meet the standard of Nature Communications.

Response to comment

Reviewer:

Major concerns

First, in my opinion, the causality between IgG glycosylation and immune response in the progeny is not demonstrated. Actually, we cannot ascertain that it is altered immunity in offspring that results in modification of IgG glycosylation profiles, and not the reverse as stated by authors: oxidative stress induced by ozone may induce various effects in mothers then progeny, for example altering mother microbiota composition (and then that of the progeny) and epigenetic mark in tissues and cells in utero, all of which may alter immunity in offspring independently of IgG glycosylation. Moreover, exposure has a great impact on weight and dietary intake of mothers, which

are stress impacting in utero development and functions maturation (as exemplified in many parameters in supplementary figures S2 and S3 – on the opposite to the authors statement lines 138 139).

For example, were adoptions tested to address the respective role of in utero vs lactation (and then potentially gly-IgG transfer through yolk sac vs milk) phases in the effects observed in progeny? What would be the results using IgG deficient dams or FcRn deficient mice?

Authors:

We sincerely thank the reviewer for their insightful comments on the mechanistic interpretation of our study and for highlighting the potential involvement of other pathways, such as maternal microbiota, epigenetic reprogramming, metabolic stress effects, maternal body weight, and gestational food intake. We agree that maternal environmental exposure programs offspring immunity through a multifactorial process. We also agree with the reviewer that employing IgG-deficient or FcRn-deficient dams would provide conclusive causal evidence. However, considering that the use of IgG-deficient dams can lead to reduced pup survival rates, which may complicate the interpretation of developmental immune phenotypes. Therefore, we opted for the cross-fostering approach as a reliable and physiologically relevant alternative.

Offspring from control (C) or 0.5 ppm O_3-exposed dams (L) were reciprocally fostered at birth and assessed in adulthood using an active systemic anaphylaxis (ASA) model: ACS/ACA (unexposed controls, i.e., control offspring nursed by control dams, challenged with saline or BSA, respectively), ALS/ALA (exposed both in utero and via lactation, i.e., exposed offspring nursed by exposed dams, challenged with saline or BSA, respectively), CLS/CLA (exposed only in utero, i.e., exposed offspring nursed by control dams, challenged with saline or BSA, respectively), and LCS/LCA (exposed only during lactation, i.e., control offspring nursed by exposed dams, challenged with saline or BSA, respectively). As anticipated, the ALA group exhibited the most severe allergic phenotype. Crucially, direct comparison of the single-exposure groups revealed that both CLA and LCA offspring developed significant and broadly comparable allergic sensitization relative to ACA controls. Specifically, both groups displayed significant hypothermia upon allergen challenge, elevated levels of allergen-specific IgG (anti-BSA IgG), and increased total immune cell ($CD45^+$) infiltration in the lungs. Statistical analysis confirmed no significant difference between the CLA and LCA groups in these core metrics of anaphylaxis severity and humoral sensitization. However, nuanced differences in the inflammatory profile were observed: LCA offspring exhibited more pronounced neutrophil infiltration in the lungs, while CLA offspring uniquely demonstrated a significant increase in eosinophils (Fig. 11 in the response to reviewers). The comparable sensitizing efficacy of CLA (in utero-only) and LCA (lactational-only) exposures aligns perfectly with the established physiology of maternal IgG transfer, where antibodies are delivered via the yolk sac during gestation and via milk during the postnatal period. The effectiveness of the lactational-only (LCA) window provides strong, indirect evidence for the involvement of a milk-borne factor; given its known immunomodulatory capacity and our documented alterations in its profile, differentially glycosylated maternal IgG is a prime candidate. These findings

have been incorporated into the revised manuscript in the Results section (lines 599-657).

Thus, the cross-fostering experiment reveals that this programming effect can be transmitted through either the gestational or lactational route. This finding provides direct experimental evidence supporting the hypothesis that glycosylation-modified maternal IgG serves as a transmissible signal across these critical windows. The fact that *in utero* exposure alone (CLA) is effective underscores the potential importance of the altered IgG glycosylation signature we detected in maternal serum at E18.5. We acknowledge, as the reviewer rightly notes, that other factors (altered microbiota, metabolites, cytokines) could also be transmitted through these same windows and contribute to the phenotype. Our data do not exclude these parallel mechanisms; instead, we highlight that MIA-induced maternal IgG glycosylation reprogramming provides a complementary perspective on the mechanisms that may operate during critical windows of developmental immune transfer following prenatal exposure.

Further supporting this causal link between maternal IgG glycosylation and offspring outcome, we performed complementary *in situ* evidence at the transfer interface (Fig. 1 in the response to reviewers). Triple immunofluorescence co-staining of yolk sac tissue at E18.5—for FcRn, total IgG, and the specific lectins marking exposure-altered glycans (GHA for 0.5 ppm, TL for 1 ppm)—revealed that IgG carrying these glycosylation modifications exhibited significantly higher colocalization with the FcRn receptor in yolk sacs of male fetuses compared to females. This direct visualization demonstrates that MIA-induced glycosylation remodeling actively and sex-dependently influences the IgG-FcRn interaction at the maternal-fetal interface, providing a mechanistic basis for the differential transfer efficiency and subsequent sex-specific IgG levels observed in offspring. These findings have been incorporated into the revised manuscript in the Results section (lines 361-367 in the Results section of the revised manuscript).

Our findings suggest that modifications to maternal IgG can serve as a key carrier of environmental programming information. We have incorporated these results (lines 599-657, lines 361-367) and a balanced discussion of multifactorial causality into the revised manuscript: "While the primary focus of this study is on the role of IgG glycosylation modifications in O_3 exposure-affected maternal–fetal immune transfer, we have not excluded other plausible parallel mechanisms. For instance... Other maternal factors—including alterations in gut microbiota composition (which can vertically shape the offspring microbiome) and intrauterine epigenetic modifications—may independently or synergistically influence offspring immunity…Overall, the present study demonstrated that MIA-induced maternal IgG glycosylation reprogramming provides a complementary perspective on the mechanisms that may operate during critical windows of developmental immune transfer following prenatal exposure" (lines 857-880). We have also explicitly highlighted a critical future research direction in the revised discussion: "However, several limitations require further investigation: studies employing IgG deficient or FcRn deficient dams would be valuable to establish definitive causal evidence for the role of glycosylated maternal IgG in this intergenerational transmission; studies generating GlcNAc-modified mouse

IgG are needed to directly test its contribution to allergic susceptibility *in vivo*; and population-based studies are needed to validate the allergic relevance of MIA-driven offspring IgG glycosylation reprogramming observed in murine models" (lines 890-896).

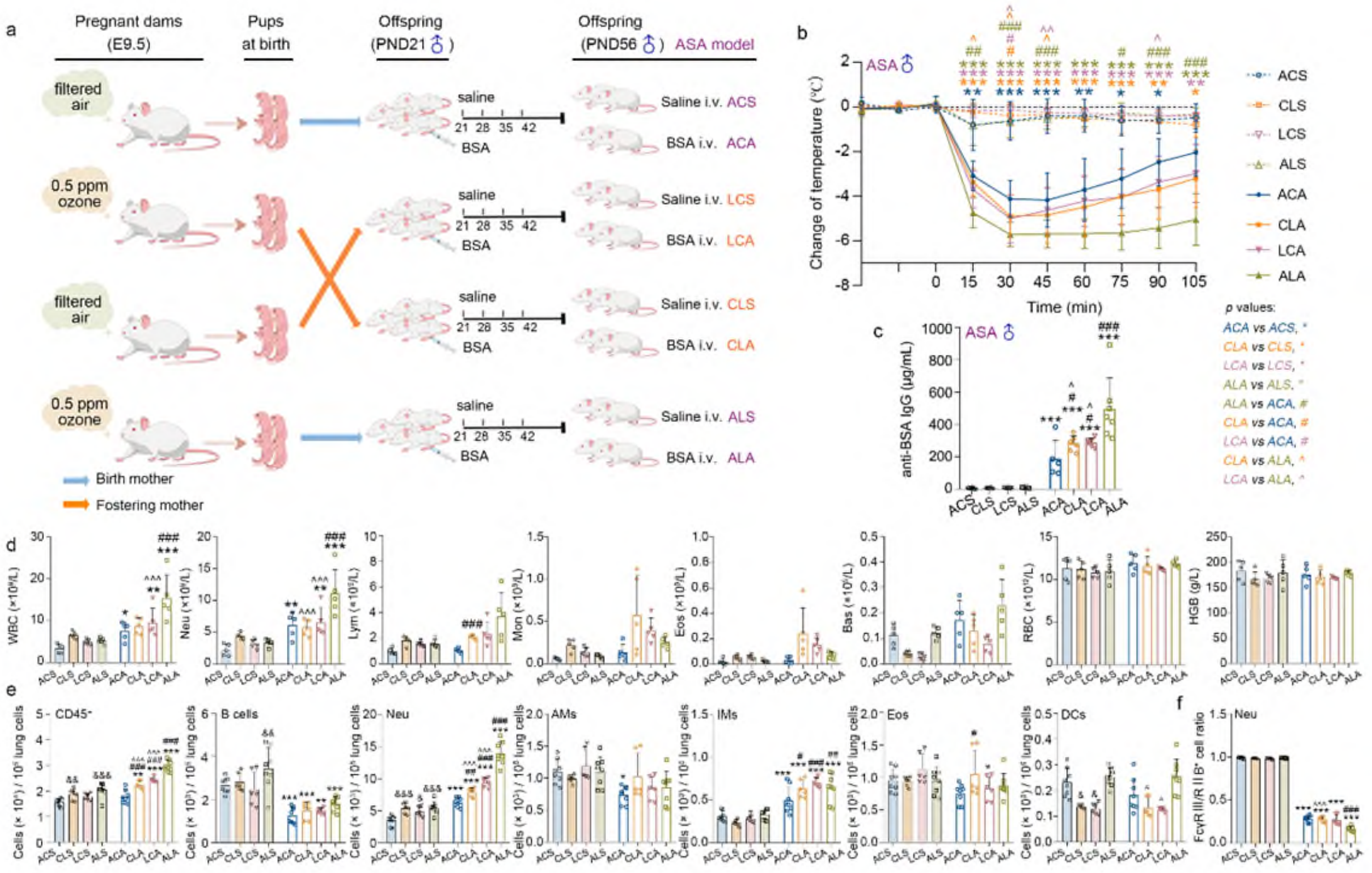

Fig. 11. Allergic programming is transmitted via either in utero or lactational exposure. **a** Experimental design of cross-fostering within the 0.5 ppm O_3 exposure paradigm. Pregnant dams were exposed to filtered air (labeled as control (C) group) or O_3 (0.5 ppm) for 4 hours daily from gestational day 9.5 (E9.5) until parturition. Two parallel cohorts of dams were established: one cohort participated in the cross-fostering procedure, in which entire pups were reciprocally exchanged between control dams and dams subjected to 0.5 ppm O_3 exposure within 12 hours after birth; the other cohort did not undergo cross-fostering, and pups remained with their biological dams throughout the nursing period. All male offspring from these groups were then evaluated, starting from PND21, using the identical ASA sensitization and challenge protocol described in Fig. 1 in the revised manuscript, resulting in the following four experimental groups: ACS/ACA (control pups nursed by control dams, challenged with saline or bovine serum albumin (BSA), respectively); ALS/ALA (exposed pups nursed by 0.5 ppm O_3-exposed dams, challenged with saline or BSA, respectively); CLS/CLA (exposed pups nursed by control dams, challenged with saline or BSA, respectively); and LCS/LCA (control pups nursed by 0.5 ppm O_3-exposed dams, challenged with saline or BSA, respectively). The elements in this figure were created using BioRender.com. b Body temperature changes in ASA challenged male offspring from the cross fostering design (n = 8-10). **c** Serum anti BSA IgG levels in ASA challenged male offspring from the cross fostering design (n = 5-7). **d** Complete blood count (CBC) analysis of ASA challenged male offspring from the cross fostering design. Quantitative measurements included white blood cells (WBC), neutrophils (Neu), lymphocytes (Lym), monocytes (Mon), eosinophils (Eos), basophils (Bas), red blood cell (RBC) count, and hemoglobin (HGB) concentration (n = 5). **e** Inflammatory cell counts ($CD45^+$ cells, B cells, neutrophils (Neu), alveolar macrophages (AMs), interstitial macrophages (IMs), eosinophils

(Eos), and dendritic cells (DCs)) in ASA challenged male offspring lungs from the cross fostering design (n = 6). **f** FcγRIII/FcγRIIB⁺ lung neutrophil subset proportions in ASA challenged male offspring from the cross fostering design (n = 6-8). Data were shown as the mean ± SD. Statistical analysis was performed using one-way ANOVA. **p* < 0.05, ***p* < 0.01, ****p* < 0.001 (ACA vs. ACS, CLA vs. CLS, LCA vs. LCS, ALA vs. ALS); #*p* < 0.05, ##*p* < 0.01, ###*p* < 0.001 (CLA vs. ACA, LCA vs. ACA, ALA vs. ACA); ^*p* < 0.05, ^^*p* < 0.01, ^^^*p* < 0.001 (CLA vs. ALA, LCA vs. ALA); &*p* < 0.05, &&*p* < 0.01, &&&*p* < 0.001 (CLS vs. ACS, LCS vs. ACS, ALS vs. ACS). [Editorial note: The elements in this figure were Created with BioGDP.com. Jiang S, Li H, Zhang L, et al. Generic Diagramming Platform (GDP): a comprehensive database of high-quality biomedical graphics. Nucleic Acids Res. 2025;53(D1):D1670-D1676. doi:10.1093/nar/gkae973]

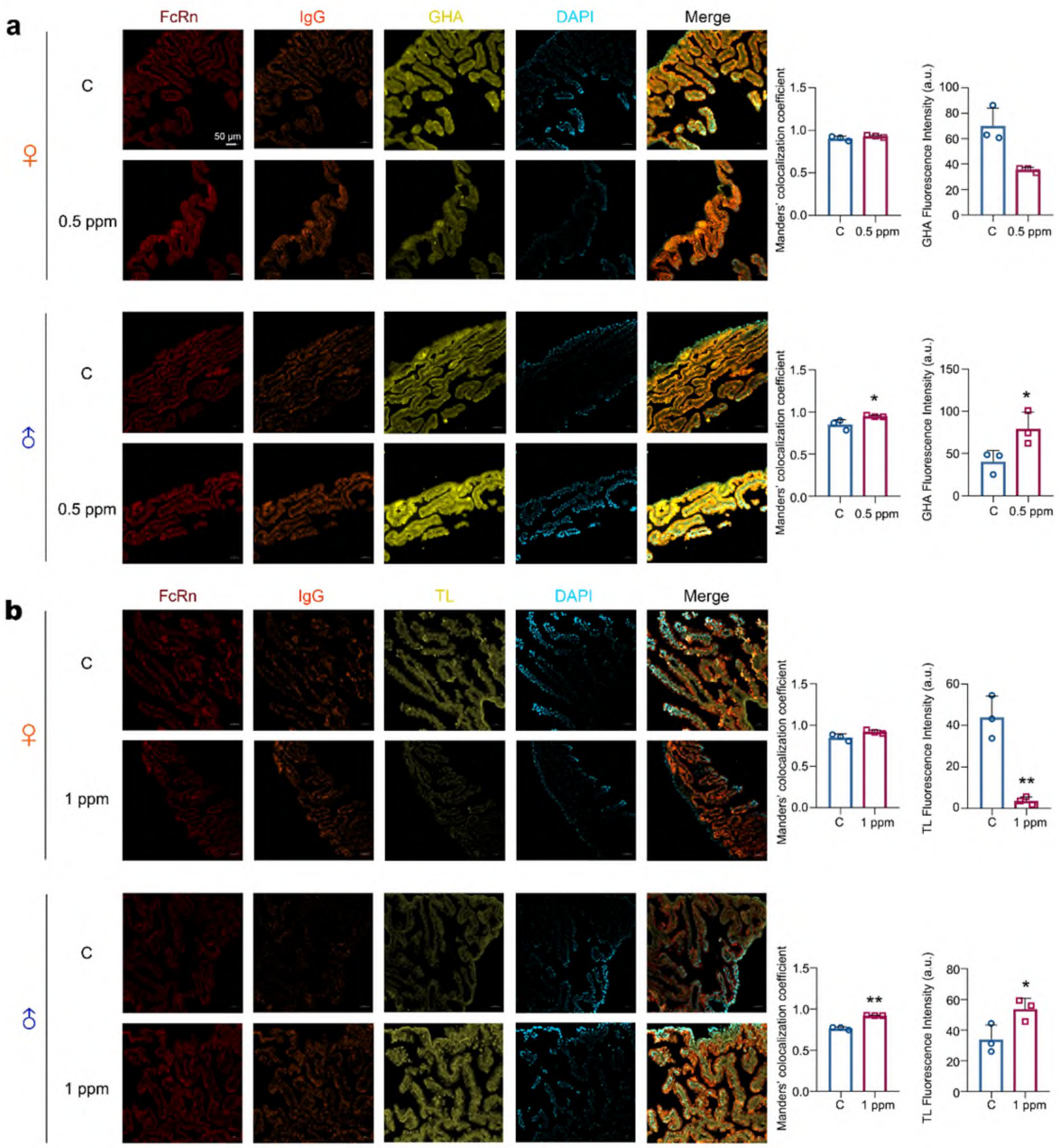

Fig. 1. Co-localization and quantification of FcRn, IgG, and differential lectin staining in E18.5 mouse yolk sacs. **a** Left: Representative immunofluorescence images of yolk sac sections from control and 0.5 ppm O_3-exposed groups at E18.5, stained for FcRn (red), IgG (orange), and FITC-conjugated GHA lectin (yellow), with DAPI counterstain (cyan), scale bar, 50 μm. Right:

Quantification of the FcRn & IgG Manders' colocalization coefficient and the fluorescence intensity of GHA (6 random fields per sample). Top panels show female fetal yolk sacs; bottom panels show male fetal yolk sacs (n = 3). **b** Left: Representative images of yolk sac sections from control and 1 ppm O_3-exposed groups at E18.5, stained for FcRn (red), IgG (orange), and FITC-conjugated TL lectin (yellow), with DAPI (cyan), scale bar, 50 µm. Right: Quantification of FcRn & IgG Manders' colocalization coefficient and TL fluorescence intensity (6 random fields per sample). Top: female; bottom: male (n = 3). Data were presented as mean ± SD. Statistical analysis was performed by two-tailed Student's t-test. $^*p < 0.05$ and $^{**}p < 0.01$ compared with the corresponding control group.

Reviewer:

Concerning IgG transfer through yolk sac, it concerns only IgG2 and IgG3 whereas IgG1 are only transfer through breast milk (pioneer studies form Morshed et al., 1993 & Grey et al., 1971)? Could you please discuss your results in front of that knowledge?

Authors:

We fully agree with the reviewer's perspective. Pioneering work, including the study by Morshed et al. in the context of a specific pathogen model (anti-Borrelia duttonii infection) and the characterization of mouse IgG subclasses by Grey et al., established fundamental principles regarding maternal antibody transfer in mice. These studies indicate that, mechanistically, the yolk sac serves as a primary route for the fetal transfer of certain IgG subclasses (e.g., IgG2 and IgG3), while breast milk is a major pathway for postnatal acquisition of others (e.g., IgG1)[1, 2]. This framework forms the basis for our understanding of the passive transfer of maternal antibodies.

At the same time, research by Appleby et al. suggests that these transfer pathways are not entirely mutually exclusive in physiology; rather, they constitute a dynamic and continuous process involving multiple stages and cooperative pathways[3]. This pioneering work systematically demonstrates that major IgG subclasses—including IgG1, IgG2a, and IgG2b—are already present in the fetal circulation by gestational day 15, at concentrations of approximately 10% to 15% of adult levels. This confirms that low levels of IgG1 can indeed undergo early intrauterine transfer via the yolk sac. Subsequently, fetal IgG levels continue to rise throughout gestation, reaching about 20% to 30% of adult levels at birth, thereby providing foundational passive immunity to the neonate. Additionally, the same study documented substantial postnatal transfer of IgG2a and IgG2b via milk, confirming that the yolk sac is not their exclusive route of transfer[3]. In the postnatal phase, milk becomes the primary pathway for antibody acquisition, mediated by the neonatal Fc receptor (FcRn) expressed on the intestinal brush border. The efficiency of antibody transfer exhibits subclass-specific differences: IgG1 transfer is the most efficient, reaching serum concentrations comparable to adult levels within the first week after birth, whereas the accumulation of IgG2a and IgG2b proceeds more gradually. Although gut closure—the cessation of macromolecular absorption—occurs around postnatal day 16, milk continues to contain all IgG subclasses at concentrations approximately 20% of maternal serum levels[3]. Thus, overall, maternal antibody transfer constitutes a complex system involving multiple subclasses and pathways: while the yolk sac is indeed the principal fetal route for

IgG2/IgG3 and breast milk is the main postnatal source for IgG1, the two pathways are functionally complementary and sequentially connected. Together, they form a continuous and integrated network of passive immune protection, providing comprehensive defense for the offspring.

Within this theoretical framework, our experimental results can be reasonably explained. Following maternal immune activation (MIA), at E18.5, the maternal IgG subclass profile—particularly the downregulation of IgG1 and upregulation of IgG2a—undergoes remodeling. This altered “antibody blueprint” is then transmitted to the offspring through the aforementioned pathways. By PND14, the sex- and dose-specific patterns observed in total serum IgG and subclass distribution in the offspring—such as elevated IgG1 in male offspring from the 0.5 ppm exposure group and reduced IgG1 in female offspring from the 1 ppm exposure group—represent the outcome of this reprogrammed maternal antibody being cooperatively transferred via both the yolk sac and milk, and subsequently interacting with the offspring's own immune status.

This interpretation has been incorporated into the Discussion section of the revised manuscript (lines 719-748): "In offspring at PND14, maternally derived IgG retained its specific glycosylation features and exhibited marked sexual dimorphism: total IgG levels were elevated in male offspring but decreased in females. Notably, male offspring from the 0.5 ppm O_3-induced MIA group showed the most significant IgG glycosylation alterations, including increased N-acetylglucosamine and core fucosylation along with reduced mannosylation. Subclass analysis revealed that the increase of total IgG in males is primarily driven by expansion of IgGl, on which pro-inflammatory GlcNAc modifications were predominantly enriched, whereas anti-inflammatory fucosylation was observed on IgG2a. This phenomenon finds a plausible explanation within the theoretical framework of subclass-specific IgG transport. Classical studies have confirmed that IgG transport in mice exhibits distinct subclass specificity: IgG2a and IgG2b are primarily transferred prenatally via the yolk sac pathway, whereas IgG1 is predominantly delivered postnatally through breast milk. Building on this, our results indicate that in dams subjected to 0.5 ppm O_3-induced MIA, the galactosylated IgG2a and IgG2b antibodies are predominantly transported via the yolk sac. Conversely, IgG1, which is primarily transmitted postnatally via breast milk, exhibits enrichment of pro-inflammatory GlcNAc glycosylation. This indicates that MIA reprograms the glycosylation state of IgG1, thereby further reinforces this pro-inflammatory trend. These findings are consistent with established research: elevated N-acetylglucosamine has been linked to atherosclerosis through IgG-driven pro-inflammatory enhancement. Reduced core fucosylation amplifies FcγRIII-mediated inflammation in dengue, while high-mannose IgG correlates with melanoma disease activity. This intricate regulatory network suggests a synergistic interplay between the IgG glycosylation state and the inherent subclass-specific transport pathways, thereby constitutes a temporally precise regulatory framework, collectively enabling "selective transfer" based on immunological demands."

Reference:

1. Morshed MG, Yokota M, Nakazawa T, Konishi H. Transfer of antibody against Borrelia duttonii

from mother to young in ddY mice. Infect Immun. 1993 Oct;61(10):4147-52.
2. Grey HM, Hirst JW, Cohn M. A new mouse immunoglobulin: IgG3. J Exp Med. 1971 Feb 1;133(2):289-304.
3. Appleby P, Catty D. Transmission of immunoglobulin to foetal and neonatal mice. J Reprod Immunol. 1983 Jul;5(4):203-13.

Reviewer:

Moreover, isotype-specific glycosylation is not explored at all despite studies demonstrating that glycosylation and its functional effects are different for IgG1 and IgG3 versus IgG2 in mice (Barrientos et al., 2020), as observed in humans (Sneed et al., 2025). A more precise and deep analysis of sub-isotype glycosylation appear necessary to draw conclusions.

Authors:

We sincerely thank the reviewer for raising this critical point regarding the necessity of isotype-specific IgG glycosylation analysis. We fully agree that analyzing only the total IgG glycosylation profile presents a significant limitation, as it fails to capture the distinct functional nuances inherent to individual subclasses. The functional differences in IgG glycosylation have been elucidated at the subclass level by studies conducted by Barrientos et al. and Sneed et al[1, 2]. In direct response to this insightful suggestion, we have conducted comprehensive supplementary analyses to thoroughly profile both the concentrations and the glycosylation patterns of total IgG and its specific subclasses in maternal serum at E18.5 and in offspring serum at PND14 (Fig.2, 3 in the response to reviewers).

Our deeper, subclass-resolved analysis revealed a significant redistribution of IgG subclass abundance at E18.5, even though total IgG levels remained unchanged. Specifically, we observed that IgG1 was downregulated in the O_3-exposed dams, IgG2a was specifically elevated in the 0.5 ppm exposure group, and IgG3 levels were reduced. Crucially, our lectin microarray analysis uncovered that each of these altered subclasses acquired a distinct glycosylation signature. In the 0.5 ppm group, the overall increase in galactosylation (reflected by stronger binding to the GHA lectin) was driven by elevated levels of pro-inflammatory glycan motifs—specifically, increased galactosylation (e.g., structures such as Galβ4GlcNAc and Galβ3GalNAc) on the IgG2a and IgG2b subclasses. In contrast, the 1 ppm group induced a different pattern (marked by an overall increase in fucosylation), which was characterized by elevated sialylation (indicated by stronger binding to SNA and SNA-I lectins) and mannosylation specifically on the IgG1 subclass—modifications that are typically associated with immunoregulatory or anti-inflammatory functions.

This preconceptionally programmed maternal antibody "blueprint" was subsequently transmitted and manifested in the offspring by PND14 in a remarkable sex-specific manner. Male offspring from the 0.5 ppm MIA group exhibited elevated total IgG, a change driven primarily by an expansion of the IgG1 subclass. Glycosylation analysis further showed that this expanded IgG1 pool in males was armed with pro-inflammatory modifications, such as increased GlcNAc (reflected by stronger DSL lectin binding), aligning with the pro-inflammatory shift observed in the

total IgG profile. Concurrently, their IgG2a subclass displayed enhanced fucosylation, which may provide a degree of anti-inflammatory balancing. Female offspring from the 1 ppm group presented a contrasting profile: they showed reduced total IgG due to a significant decrease in IgG1, along with a reduction in pro-inflammatory galactosylation of IgG2a subclass (e.g., evidenced by decreased lectin binding to ACL [specific for Galβ3GalNAc] and PHA-E [specific for Galβ4GlcNAc]), resulting in an overall net anti-inflammatory or protective phenotype.

In direct response to the reviewer's suggestion to consider isotype-specific glycosylation, we have incorporated these detailed subclass-level analyses—encompassing both quantitative concentrations and qualitative glycosylation patterns—into the revised manuscript. These new data are now presented in the Results section (lines 342-354, lines 370-378 and lines 385-396) and are discussed extensively in the revised manuscript (lines 696-707 and lines 717-748).

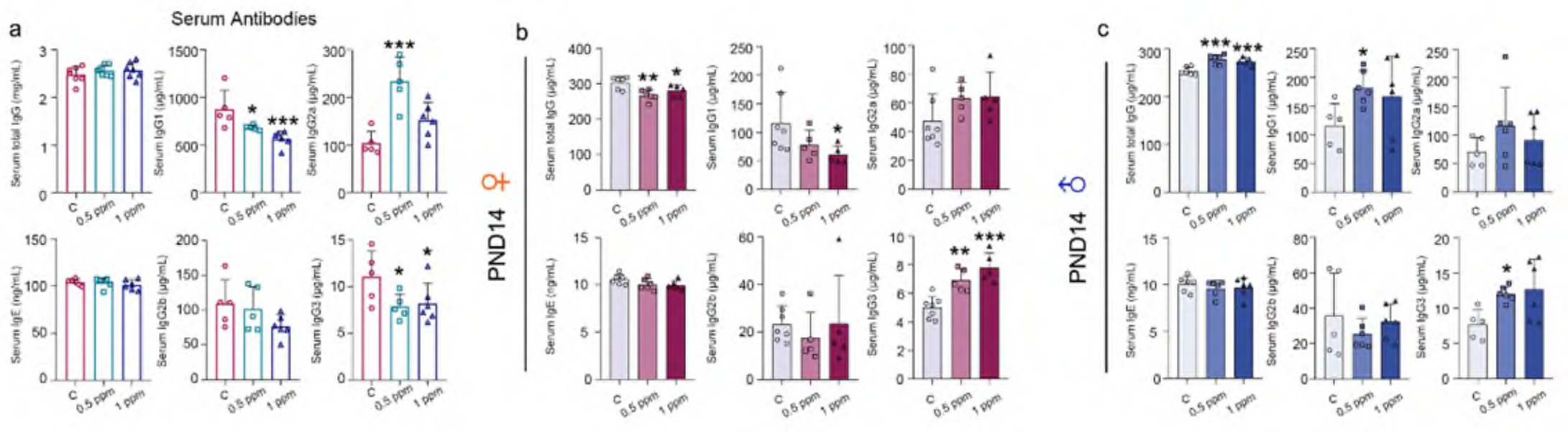

Fig. 2. Serum concentrations of total IgG and IgG subclasses (IgG1, IgG2a, IgG2b, and IgG3) in E18.5 dams **(a)**, PND14 female offspring **(b)**, and PND14 male offspring **(c)** (n = 5-6). Data were shown as the mean ± SD. Statistical analysis was performed by one-way ANOVA. **p* < 0.05, ***p* < 0.01, and ****p* < 0.001 compared with the corresponding control group.

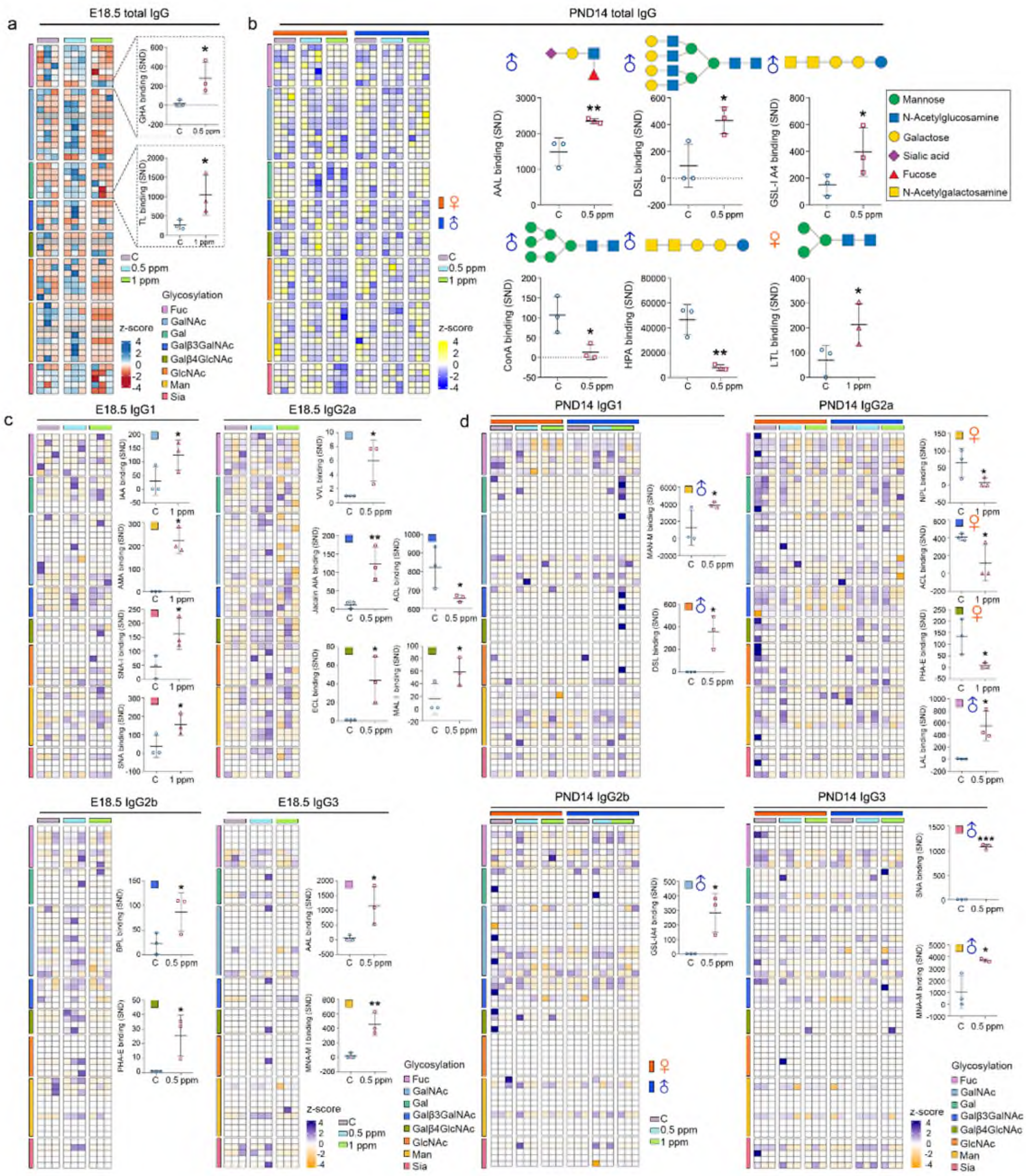

Fig. 3. IgG glycosylation profiles in maternal and offspring serum. **a** Glycosylation profile of total IgG in maternal serum at E18.5. **b** Glycosylation profile of total IgG in serum of female and male offspring at PND14. **c** Subclass-specific glycosylation profiles (IgG1, IgG2a, IgG2b, and IgG3) in maternal serum at E18.5. **d** Subclass-specific glycosylation profiles (IgG1, IgG2a, IgG2b, and IgG3) in serum of female and male offspring at PND14. The dot plots display differential lectin binding in offspring from the 0.5 ppm O_3- and 1 ppm O_3-induced MIA groups relative to offspring from control dams (n = 3). Data were shown as the mean ± SD. Statistical analysis was performed by one-way ANOVA. **p* < 0.05, ***p* < 0.01, and ****p* < 0.001 compared with the corresponding control group.

Reference:

1. Barrientos G, Habazin S, Novokmet M, Almousa Y, Lauc G, Conrad ML. Changes in subclass-specific IgG Fc glycosylation associated with the postnatal maturation of the murine immune system. Sci Rep. 2020 Sep 17;10(1):15243.

2. Sneed SL, Strandberg EA, Laureano AFS, Vattepu R, Sun Y, Tran TT, Conroy ME, Anthony RM. Molecular determinants of sialylated IgG anti-inflammatory activity. Proc Natl Acad Sci U S A. 2025 May 20;122(20): e2411600122.

Reviewer:

In addition, IgG-dependent anaphylaxis is more relevant for high doses of circulating antigens, whereas IgE-dependent anaphylaxis is the mechanism involved at low dose of antigen and at mucosal sites, i.e. food or respiratory allergic reactions. Indeed, as clearly reviewed in Reber et al (cited as ref 22 by authors), IgG-mediated anaphylaxis requires a much larger dose of antigen than does IgE-mediated anaphylaxis, and systemic anaphylaxis also requires systemic absorption of ingested antigen. Such conditions could be encountered in the case of anaphylaxis occurring in response to infusion of large quantities of a drug or a therapeutic monoclonal antibodies. In that context, the rationale of the present study, and the animal models used, does not seem relevant and transposable to human. Moreover, induction of severe systemic anaphylaxis remain, in my opinion, ethically criticizing (2 out 8 males mice in the ALA group exhibited signs of anaphylactic shock and loss of vital functions; decline of core temperature superior to 5°C): what were the limit points and were those mice sacrificed to limit suffering?

Authors:

We sincerely appreciate the reviewer's valuable insights regarding the mechanisms of IgG-mediated anaphylaxis and its clinical significance. The protocols for both active and passive systemic anaphylaxis in our study were designed according to well-established and meticulously detailed procedures from the literature, which utilize doses appropriate for investigating IgG-mediated allergic responses. For active systemic anaphylaxis (ASA) model, Todorova et al. sensitized mice with weekly intraperitoneal injections of 200 μg BSA, followed by an intravenous challenge with 500 μg of the same antigen to induce the reaction[1]. Similarly, Tsujimura et al. described a model involving intraperitoneal priming with antigen (0.5 mg), followed by an intravenous challenge with the corresponding antigen (100 μg) two weeks later[2]. Accordingly, in our ASA model, offspring were sensitized via weekly intraperitoneal injections of 200 μg BSA starting post-weaning (PND21). After four sensitizations, at adulthood (PND56), they were challenged via tail vein injection with 500 μg BSA to elicit the reaction. For passive systemic anaphylaxis (PSA) model, Tsujimura et al. employed intravenous sensitization with 500 μg of a specific IgG1 monoclonal antibody, followed three hours later by a challenge with 1 mg of antigen. Qiao et al. directly administered pre-formed complexes consisting of 90 μg IgG and 30 μg antigen[3]. Therefore, in our PSA model, offspring at PND56 received an intravenous injection of pre-formed IgG immune complexes containing 90 μg IgG1 and 30 μg OVA.

Thus, the antigen and IgG-IC doses used in our study were selected to align with these established models and coincide with the critical experimental time windows, thereby ensuring specific activation of the IgG-mediated anaphylactic pathway under investigation. The rationale for employing these models is grounded in the established understanding that IgG-induced anaphylaxis primarily depends on the IgG-FcγR

cascade. As highlighted by Galli et al., IgG immune complexes can engage activating FcγRs (such as FcγRIII) on myeloid cells, triggering inflammatory cascades. Our primary objective was to investigate whether prenatal O_3 exposure reprograms offspring immunity, potentially altering IgG glycosylation and its interaction with FcγRIII, thereby modulating the severity of the allergic response and subsequent inflammation. These established models allow us to probe this specific mechanism.

We fully acknowledge the reviewer's observation that IgG-mediated anaphylaxis typically requires higher antigen doses compared to the IgE-mediated pathway, a perspective that has been clearly articulated in the review by Reber et al[4]. We agree that such conditions, involving systemic exposure to substantial amounts of antigen, align with clinical scenarios like reactions to infusions of drugs or therapeutic antibodies for those who truly need it.

Beyond these high-dose contexts, it is increasingly recognized that IgG-mediated pathways constitute a significant and distinct mechanism of hypersensitivity, supported by a growing body of clinical and experimental evidence[5]. Clinical observations indicate that many patients presenting with anaphylactic symptoms do not always exhibit elevated allergen-specific IgE or histamine release, pointing to alternative triggering mechanisms. In specific food allergies, such as those to lipid transfer proteins (LTPs) or galactose-alpha-1,3-galactose (α-gal), patients often possess both allergen-specific IgE and IgG antibodies[6-9]. Studies suggest that IgG subclasses and the expression of activating FcγR may influence reaction severity, indicating a potential modulatory or synergistic role for IgG alongside the classical IgE pathway.

Importantly, experimental models have demonstrated that IgG-mediated systemic anaphylaxis can be triggered with relatively modest doses of antibody and antigen (e.g., as low as 0.04 mg antibody and 0.008 mg antigen in some systems), engaging activating FcγR (e.g., FcγRIIA, FcγRIII) on neutrophils, macrophages, and basophils to release potent mediators like platelet-activating factor (PAF)[10, 11]. This mechanism may explain severe reactions even in the absence of marked histamine release. Furthermore, the pathophysiological relevance of IgG immune complex-driven inflammation is well-established in a range of conditions beyond anaphylaxis, including acute lung injury (ALI)/acute respiratory distress syndrome (ARDS), systemic lupus erythematosus, and rheumatoid arthritis. These diseases share critical effector mechanisms—such as FcγR activation, inflammatory mediator release, and granulocyte recruitment—with aspects of IgG-mediated hypersensitivity[12-16].

Therefore, while our experimental models employ doses consistent with established protocols for probing the IgG-FcγR axis, they are situated within this broader biological framework. The primary goal of our study was to investigate whether prenatal ozone exposure reprograms offspring immunity, potentially by altering IgG glycosylation and its interaction with FcγRIII, which could modulate the inflammatory response upon immune challenge later in life. This approach aims to contribute to understanding how early-life environmental exposures might influence such alternative hypersensitivity pathways.

We have added the following exposition to the Discussion section of the revised manuscript (lines 790-807) to further elaborate on this point: "…This finding aligns

with recent experimental evidence. This pathway holds significant relevance in clinical scenarios involving substantial systemic antigen exposure, such as reactions to drug or therapeutic antibody infusions. Beyond these high-dose scenarios, growing experimental evidence supports that IgG-mediated systemic anaphylaxis can occur at antigen doses lower than conventionally recognized thresholds (e.g., as low as 0.008 mg antigen and 0.04 mg antibody). The key mechanism involves IgG–antigen complexes engaging Fcγ receptors (e.g., FcγRIII), which activates effector cells including neutrophils and macrophages and promotes the release of inflammatory mediators such as platelet-activating factor (PAF). Such IgG immune complex-triggered responses have been demonstrated in populations with specific food allergies, such as to lipid transfer protein (LTP)-rich foods, and are also pathophysiologically relevant to a range of conditions including acute lung injury (ALI), acute respiratory distress syndrome (ARDS), and rheumatoid arthritis. We have demonstrated that male offspring from O_3-exposed dams (the ALA group) exhibit a significant increase in neutrophils—key responder cells in allergic reactions—accompanied by markedly altered FcγRIII expression levels on these cells, findings that directly corroborate this antibody-myeloid receptor interaction mechanism."

Regarding the two mice that exhibited severe anaphylaxis as noted by the reviewer, we apologize for not including these experimental details in the original manuscript. We fully recognize that animal welfare is of utmost importance, and any clear signs of severe suffering should be promptly addressed according to established humane endpoint criteria. In our study, all procedures were conducted under strict protocols approved by the Committee of Scientific Research at Shanxi University. Prior to the study, we established clear humane endpoint criteria in compliance with the AVMA Guidelines for the euthanasia of animals and China's guideline of assessment for humane endpoints in animal experiment (RB/T 173-2018). These criteria included: 1) loss of righting reflex, 2) severe dyspnea, or 3) any signs of circulatory failure indicative of shock. Regarding the two male mice in the ALA group that showed signs of anaphylactic shock and loss of vital signs, they were humanely euthanized immediately to prevent prolonged suffering. We have now clarified these ethical safeguards in the Method section of the revised manuscript (lines 1030-1032), stating clearly: "Any animal exhibiting loss of righting reflex, severe respiratory distress, or signs of circulatory failure was euthanized immediately to avoid unnecessary suffering."

Reference:

1. Todorova B, Godon O, Conde E, Gillis CM, Iannascoli B, Richard-Le Goff O, Fiole D, Roumenina LT, Leusen JHW, Murphy AJ, Macdonald LE, Reber LL, Jönsson F, Bruhns P. IgG Subclass-Dependent Pulmonary Antigen Retention during Acute IgG-Dependent Systemic Anaphylaxis in Mice. J Immunol. 2022 Oct 1;209(7):1243-1251.
2. Tsujimura Y, Obata K, Mukai K, Shindou H, Yoshida M, Nishikado H, Kawano Y, Minegishi Y, Shimizu T, Karasuyama H. Basophils play a pivotal role in immunoglobulin-G-mediated but not immunoglobulin-E-mediated systemic anaphylaxis. Immunity. 2008 Apr;28(4):581-9.
3. Qiao SW, Kobayashi K, Johansen FE, Sollid LM, Andersen JT, Milford E, Roopenian DC, Lencer WI, Blumberg RS. Dependence of antibody-mediated presentation of antigen on FcRn. Proc Natl

Acad Sci U S A. 2008 Jul 8;105(27):9337-42.
4. Reber LL, Hernandez JD, Galli SJ. The pathophysiology of anaphylaxis. J Allergy Clin Immunol. 2017 Aug;140(2):335-348.
5. Beutier H, Gillis CM, Iannascoli B, Godon O, England P, Sibilano R, Reber LL, Galli SJ, Cragg MS, Van Rooijen N, Mancardi DA, Bruhns P, Jönsson F. IgG subclasses determine pathways of anaphylaxis in mice. J Allergy Clin Immunol. 2017 Jan;139(1):269-280.e7.
6. Muñoz-Cano R, Picado C, Valero A, Bartra J. Mechanisms of Anaphylaxis Beyond IgE. J Investig Allergol Clin Immunol. 2016;26(2):73-82; quiz 2p following 83.
7. Muñoz-Cano R, Pascal M, Bartra J, Picado C, Valero A, Kim DK, Brooks S, Ombrello M, Metcalfe DD, Rivera J, Olivera A. Distinct transcriptome profiles differentiate nonsteroidal anti-inflammatory drug-dependent from nonsteroidal anti-inflammatory drug-independent food-induced anaphylaxis. J Allergy Clin Immunol. 2016 Jan;137(1):137-146.
8. Cianferoni A. Non-IgE-mediated anaphylaxis. J Allergy Clin Immunol. 2021 Apr;147(4):1123-1131.
9. Rispens T, Derksen NI, Commins SP, Platts-Mills TA, Aalberse RC. IgE production to α-gal is accompanied by elevated levels of specific IgG1 antibodies and low amounts of IgE to blood group B. PLoS One. 2013;8(2): e55566.
10. Ishikawa R, Tsujimura Y, Obata K, Kawano Y, Minegishi Y, Karasuyama H. IgG-mediated systemic anaphylaxis to protein antigen can be induced even under conditions of limited amounts of antibody and antigen. Biochem Biophys Res Commun. 2010 Nov 26;402(4):742-6.
11. Jönsson F, Mancardi DA, Kita Y, Karasuyama H, Iannascoli B, Van Rooijen N, Shimizu T, Daëron M, Bruhns P. Mouse and human neutrophils induce anaphylaxis. J Clin Invest. 2011 Apr;121(4):1484-96.
12. Li C, Li Y, Zhang H, Zhuo Y, Zhang L, Yang L, Gao Q, Tu Z, Shao R, Wang Y, Zhang J, Cui L, Zhang S. Xuanfei Baidu Decoction suppresses complement overactivation and ameliorates IgG immune complex-induced acute lung injury by inhibiting JAK2/STAT3/SOCS3 and NF-κB signaling pathway. Phytomedicine. 2023 Jan;109: 154551.
13. Yan C, Chen J, Ding Y, Zhou Z, Li B, Deng C, Yuan D, Zhang Q, Wang X. The Crucial Role of PPARγ-Egr-1-Pro-Inflammatory Mediators Axis in IgG Immune Complex-Induced Acute Lung Injury. Front Immunol. 2021 Feb 25;12: 634889.
14. Scherlinger M, Kolios AGA, Kyttaris VC, Tsokos GC. Advances in the treatment of systemic lupus erythematosus. Nat Rev Drug Discov. 2025 Dec;24(12):926-944.
15. Zubler RH, Nydegger U, Perrin LH, Fehr K, McCormick J, Lambert PH, Miescher PA. Circulating and intra-articular immune complexes in patients with rheumatoid arthritis. Correlation of 125I-Clq binding activity with clinical and biological features of the disease. J Clin Invest. 1976 May;57(5):1308-19.
16. Nangaku M, Couser WG. Mechanisms of immune-deposit formation and the mediation of immune renal injury. Clin Exp Nephrol. 2005 Sep;9(3):183-91.

Reviewer:

In the same line, what about IgE transfer through milk and their glycosylation?

Authors:

We sincerely appreciate the reviewer's insightful question regarding IgE transfer through breast milk and its glycosylation. Regarding the placental and yolk sac transfer, IgG has traditionally been considered the only maternal antibody subtype capable of

crossing the placental barrier[1, 2]. However, recent studies on human cord blood samples and mouse models suggest that maternal IgE can also be transferred to the fetus, albeit through an IgG-mediated immune complex (IgE-IgG ICs) mechanism rather than direct transport of free IgE[3, 4]. Surface plasmon resonance (SPR) structural studies have demonstrated that FcRn selectively mediates IgG transport while showing no binding affinity for IgE[5]. This finding is further supported by FcRn knockout models where IgE placental transfer was completely abolished, whereas CD23 (the primary IgE receptor) deficient mice still showed detectable IgE levels, confirming that FcRn indirectly mediates IgE transfer through IgG-ICs transport[6].

Clinical observations reveal that IgE concentrations detected in cord blood are 50-100 times lower than matched maternal blood samples, indicating that IgE does not undergo the same active, FcRn-mediated placental transport process as IgG[7, 8]. Moreover, an alternative perspective in human research suggests that IgE detected in cord blood may originate from fetal synthesis rather than maternal placental antibody transfer[9]. This view is supported by Yu et al.'s systematic examination of 57 maternal-neonatal paired samples, which found that while total and specific IgE detection rates were 100% in maternal blood, only 7% (4/57) of cord blood samples showed detectable total IgE, with no significant correlation between maternal and neonatal total IgE levels and no detectable specific IgE in any cord blood sample[10]. Another maternal-neonatal analysis similarly found that only 2.2% of neonates had detectable specific IgE antibodies, further corroborating that IgE cannot effectively cross the placental barrier like IgG[11].

Regarding breast milk transmission, current evidence shows that while IgE can be detected in breast milk, its concentration is extremely low (0.3 μg/L), with no significant difference observed between atopic and non-atopic mothers[12-14]. The IgE concentration in breast milk is substantially below the threshold required for infant immune regulation (approximately three orders of magnitude lower than effective immunomodulatory doses)[15]. Bahna et al. proposed that IgE cannot be passively transferred from maternal circulation to milk, as only 41% of colostrum samples (16/39) contained detectable IgE (0.5-6 IU/ml), while the rest were below the detection limit[16]. Quantitative analysis revealed that IgE constitutes only 0.000004% and 0.000028% of immunoglobulins in human colostrum and mature milk, respectively[17]. Therefore, it is reasonable to hypothesize that maternally transferred IgG likely plays a dominant role in neonatal immune programming, while IgE's contribution under non-high dose experimental conditions appears negligible.

In our study, we directly measured serum IgE levels in E18.5 dams, IgE in PND14 whey (breast milk), and serum IgE in both female and male PND14 offspring to further evaluate its potential role. The results showed that IgE concentrations were low across all samples, with no significant or biologically meaningful differences observed between the control (C) and exposure groups (0.5 ppm and 1 ppm) (Fig.3 b-e in the revised manuscript, Fig. 12 in the response to reviewers). Notably, the results were consistent with literature indicating minimal transfer via breast milk. Therefore, we did not perform further analysis of IgE glycosylation. We have expanded the Discussion section to include the following points (lines 857-880): "While the primary focus of

this study is on the role of IgG glycosylation modifications in O_3 exposure-affected maternal–fetal immune transfer, we have not excluded other plausible parallel mechanisms……In addition, in contrast to IgG, the transfer efficiency of maternal IgE to offspring via the placenta or breast milk is remarkably low, with its limited transport believed to depend primarily on the formation of complexes with IgG rather than on direct transfer—further underscoring the dominant role of maternal IgG in neonatal immune programming. Whether glycosylation of maternal IgE might also fine-tune its interaction with IgG and thereby influence the formation and transport of IgE–IgG immune complexes represents an interesting direction for future research into the potential functions of antibodies at very low concentrations. Overall, the present study demonstrated that MIA-induced maternal IgG glycosylation reprogramming provides a complementary perspective on the mechanisms that may operate during critical windows of developmental immune transfer following prenatal exposure."

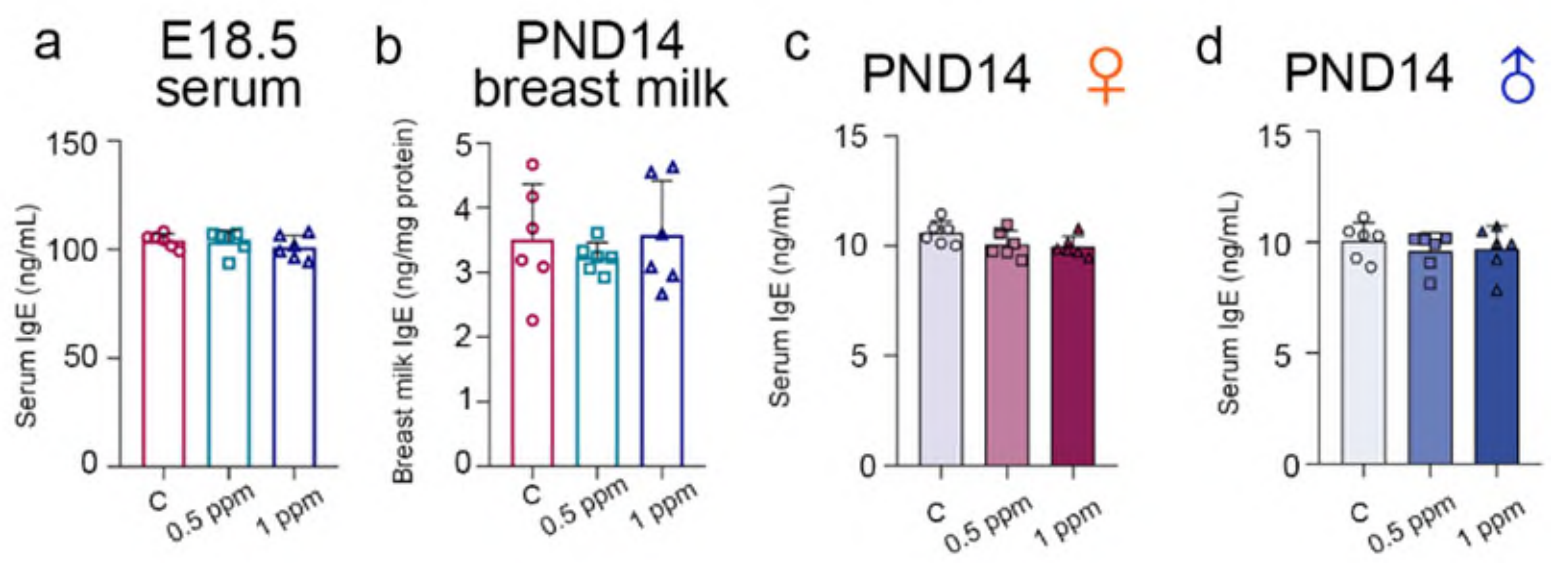

Fig. 12. IgE concentrations in maternal samples and offspring serum. **a** Total IgE levels in maternal serum at E18.5 (n = 6). **b** Total IgE levels in breast milk collected at PND14 (n = 6). **c** Total IgE levels in serum of female offspring at PND14 (n = 6). **d** Total IgE levels in serum of male offspring at PND14 (n = 6). Data were presented as mean ± SD. Statistical analysis was performed by one-way ANOVA.

Reference:

1. Chucri TM, Monteiro JM, Lima AR, Salvadori ML, Kfoury JR Jr, Miglino MA. A review of immune transfer by the placenta. J Reprod Immunol. 2010 Dec;87(1-2):14-20.
2. Pou C, Nkulikiyimfura D, Henckel E, Olin A, Lakshmikanth T, Mikes J, Wang J, Chen Y, Bernhardsson AK, Gustafsson A, Bohlin K, Brodin P. The repertoire of maternal anti-viral antibodies in human newborns. Nat Med. 2019 Apr;25(4):591-596.
3. Bundhoo A, Paveglio S, Rafti E, Dhongade A, Blumberg RS, Matson AP. Evidence that FcRn mediates the transplacental passage of maternal IgE in the form of IgG anti-IgE/IgE immune complexes. Clin Exp Allergy. 2015 Jun;45(6):1085-98.
4. Msallam R, Balla J, Rathore APS, Kared H, Malleret B, Saron WAA, Liu Z, Hang JW, Dutertre CA, Larbi A, Chan JKY, St John AL, Ginhoux F. Fetal mast cells mediate postnatal allergic responses dependent on maternal IgE. Science. 2020 Nov 20;370(6519):941-950.
5. Brinkhaus M, van der Kooi EJ, Bentlage AEH, Ooijevaar-de Heer P, Derksen NIL, Rispens T, Vidarsson G. Human IgE does not bind to human FcRn. Sci Rep. 2022 Jan 7;12(1):62.
6. Balla J, Rathore APS, St John AL. Maternal IgE Influence on Fetal and Infant Health. Immunol Rev. 2025 May;331(1): e70029.
7. Michel FB, Bousquet J, Greillier P, Robinet-Levy M, Coulomb Y. Comparison of cord blood

immunoglobulin E concentrations and maternal allergy for the prediction of atopic diseases in infancy. J Allergy Clin Immunol. 1980 Jun;65(6):422-30.
8. Bertino E, Bisson C, Martano C, Coscia A, Fabris C, Monti G, Testa T, Conti A. Relationship between maternal- and fetal-specific IgE. Pediatr Allergy Immunol. 2006 Nov;17(7):484-8.
9. Weil GJ, Hussain R, Kumaraswami V, Tripathy SP, Phillips KS, Ottesen EA. Prenatal allergic sensitization to helminth antigens in offspring of parasite-infected mothers. J Clin Invest. 1983 May;71(5):1124-9.
10. Yu Z, Chen J, Zhang Q, Yin X, Wang Y, Fu J, Zou L, Kong W. Maternofetal transfer of antibodies and the influence of maternal atopic status on the neonate. Am J Rhinol Allergy. 2015 Mar-Apr;29(2):119-23.
11. Platts-Mills TA, Erwin EA, Allison AB, Blumenthal K, Barr M, Sredl D, Burge H, Gold D. The relevance of maternal immune responses to inhalant allergens to maternal symptoms, passive transfer to the infant, and development of antibodies in the first 2 years of life. J Allergy Clin Immunol. 2003 Jan;111(1):123-30.
12. Underdown BJ, Knight A, Papsin FR. The relative paucity of IgE in human milk. J Immunol. 1976 May;116(5):1435-8.
13. Espinosa-Martos I, Montilla A, de Segura AG, Escuder D, Bustos G, Pallás C, Rodríguez JM, Corzo N, Fernández L. Bacteriological, biochemical, and immunological modifications in human colostrum after Holder pasteurisation. J Pediatr Gastroenterol Nutr. 2013 May;56(5):560-8.
14. Meng X, Dunsmore G, Koleva P, Elloumi Y, Wu RY, Sutton RT, Ambrosio L, Hotte N, Nguyen V, Madsen KL, Dieleman LA, Chen H, Huang V, Elahi S. The Profile of Human Milk Metabolome, Cytokines, and Antibodies in Inflammatory Bowel Diseases Versus Healthy Mothers, and Potential Impact on the Newborn. J Crohns Colitis. 2019 Mar 30;13(4):431-441.
15. Duchén K, Björkstén B. Total IgE levels in human colostrum. Pediatr Allergy Immunol. 1996 Feb;7(1):44-7.
16. Bahna SL, Keller MA, Heiner DC. IgE and IgD in human colostrum and plasma. Pediatr Res. 1982 Aug;16(8):604-7.
17. Rio-Aige K, Azagra-Boronat I, Castell M, Selma-Royo M, Collado MC, Rodríguez-Lagunas MJ, Pérez-Cano FJ. The Breast Milk Immunoglobulinome. Nutrients. 2021 May 26;13(6):1810.

Reviewer:

It should have been interesting to present and to discuss the role anti-inflammatory role of terminal galactose/sialic acid vs the pro-inflammatory role of agalactosylated IgG, and the natural evolution from agalactosylated to galactosylated/sialylated after weaning in mice. For example, this decrease of pro-inflammatory profile parallels the transition form innate passive immunity to endogenous production of IgG, and the corresponding timeline (also considering half-life of mother transferred IgG). In my opinion, glycosylation and its specificity is not deeply present and discussed in regards to the results and discussion. There is also some issues in experimental section, and one of the major concern is also the number of mice per groups that are far too low in my opinion, even in a reduction context of animal use for science. As an example, n=3 for flow cytometry analysis (fig 1, 2 and 4), cytokines, or lung histology and functions (fig 2 and S7)…., and 3-8 mice are shown in allergy models, with variable numbers between groups and figures, so it’s difficult to assess number of mice in each experiments/results.

Figures should be provided as individual point for each mouse and median. Moreover, how were managed the littermates for the allergy models? How many mothers and litters were considered and were litters mixed within exposure groups?

Authors:

We sincerely thank the reviewer for raising this profound question, which prompted us to conduct a more detailed examination of the dynamic functional role of IgG glycosylation during development. Accordingly, we supplemented our study with longitudinal data on IgG glycosylation in male offspring exposed to 0.5 ppm O_3 (postnatal day 28, PND28, and postnatal day 42, PND42), aiming to understand the evolution of the "pro- versus anti-inflammatory" balance in glycosylation patterns during the critical transition from passive immunity mediated by maternal antibodies to the offspring's own antibody production.

Considering the approximately 21-day half-life of IgG in mice, our selected time points appropriately cover this critical transition period. At postnatal day 14 (PND14), when circulating IgG is primarily derived from maternal transfer (via the yolk sac prenatally and breast milk postnatally), the glycosylation profile we detected was characterized by elevated core fucosylation (increased AAL binding) and elevated terminal N-acetylglucosamine (GlcNAc, increased DSL binding), alongside relatively lower galactosylation signals. While increased core fucosylation is generally associated with anti-inflammatory modulation by reducing IgG affinity for FcγRIII, the concurrent increase in terminal GlcNAc is a well-established pro-inflammatory marker. This combination, particularly the enrichment of GlcNAc on the IgG1 subclass, represents the initial glycan "imprint" transferred from the dam, reflecting state of immune activation (lines 717-739 in the revised manuscript).

As the offspring's own plasma cells began synthesizing IgG (from PND28 to PND42), the glycosylation pattern underwent a directional shift. At PND28, there was a notable increase in galactosylated structures (increased binding of GSL-IA4 and ABA). As a precursor to sialylation, it contributed to a significant increase in sialic acid expression (increased SSA binding) by PND42. We also observed a decrease in core fucosylation levels (significantly reduced binding of PSA, LCA, and TL at PND42), while galactosylated structures (WFA, GSL I-B4, and ACL) (Fig. 13 in the response to reviewers). Therefore, from PND14 to PND42, we observed the natural evolution from agalactosylated to galactosylated/sialylated after weaning in mice. Meanwhile, we observed the continuous presence of N-acetylglucosamine (increased DSL binding) (lines 761-769 in the revised manuscript).

At PND56, GlcNAc was still upregulated. Transcription factors, c-Jun and c-Fos, were activated and directly upregulated glycosyltransferase genes such as *Mgat4a* (involved in generating GlcNAc branches). This provides a mechanistic explanation for how specific glycosylation programs "set" by early maternal exposure—such as the maintenance of specific GlcNAc branching structures via *Mgat4a*—can be partially perpetuated even during the period when the offspring's own antibody production dominates (PND42 and beyond). This sustained, endogenously-driven glycosylation remodeling is directly associated with the observed exacerbation of allergic inflammatory responses in adulthood, as it may persistently influence the functional

interaction between IgG and receptors such as FcγRIII (lines 815-851 in the revised manuscript).

In summary, our data depict a dynamic scenario: maternal O_3 exposure transfers IgG carrying a specific glycan imprint via the yolk sac and breast milk, shaping the early postnatal immune milieu. As the offspring's own immune system takes over antibody production, the glycosylation profile shows dynamic characteristics. However, molecular imprints left by the early exposure through transcriptional programming (e.g., persistently activated glycosyltransferases) cause the endogenously synthesized antibodies of the offspring to partially retain pro-inflammatory glycan features. This contributes to a long-term shift in immune homeostasis and an increase in allergic susceptibility.

We sincerely thank the reviewer for raising these critical points regarding the depth of our glycosylation analysis and the rigor of our experimental design. In direct response to the comment on glycosylation specificity, we have now substantially expanded both the results and discussion. We have supplemented our data with detailed, subclass-specific glycosylation profiles and integrated a deeper mechanistic discussion of their functional implications.

Regarding the concern about sample size, we agree on the importance of robust group numbers. While our original study was conducted within the framework of reducing animal use, we have repeated key experiments (including flow cytometry, cytokine analysis, and lung histology/function mentioned by reviewer) to increase the sample size where feasible. All data in the revised figures are now presented with individual data points, and the data of above experiments contains at least five biological replicates. For the allergy models, we have clarified the experimental design in the Methods section. Offspring from O_3-exposed dams were randomly assigned to either saline or challenge groups, ensuring that mice within a single experimental group originated from different litters to minimize litter effects. The allergy model experiments involved a total of 107 dams and 627 offspring, with consistent numbers across comparison groups detailed in each figure legend. These revisions have been incorporated throughout the manuscript to strengthen the statistical validity and reproducibility of our findings.

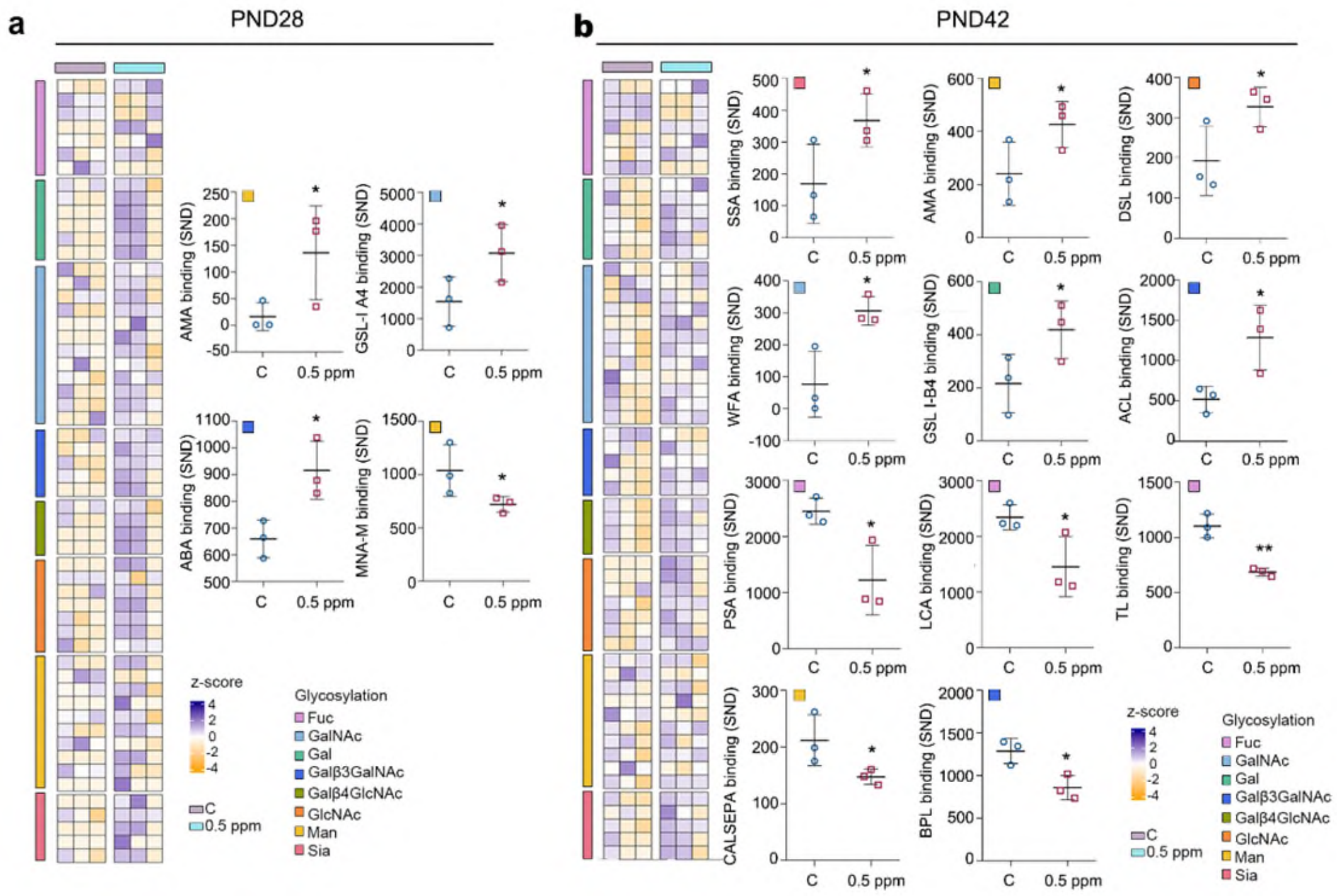

Fig. 13. IgG glycosylation profiles and differential lectin binding in male offspring at PND28 and PND42. **a** Serum IgG glycosylation profiles and significantly altered lectin binding patterns in male offspring at PND28, comparing those born to dams exposed to 0.5 ppm O_3 during pregnancy with offspring from control dams (n = 3). **b** Serum IgG glycosylation profiles and significantly altered lectin binding patterns in the same groups of male offspring at PND42 (n = 3). Data were presented as mean ± SD. Statistical analysis was performed by two-tailed Student's t-test. **p* < 0.05 and ***p* < 0.01 compared with the corresponding control group.

Reviewer:

Considering result from human cohort, we can also observe that O_3 exposure during T1 has a significant anti inflammatory effect (table 1) – i.e. the opposite effect of exposure in mid-to-late pregnancy: this has to be presented and discussed

Authors:

We thank the reviewer for this valuable comment. As rightly pointed out, our cohort analysis revealed that first-trimester O_3 exposure showed an effect opposite to that in mid-to-late gestation—namely, a significant reduction in the risk of neutrophil and monocyte abnormalities. We have incorporated this point into the Results section of the revised manuscript (lines 130-138) as follows: "Window-specific analyses revealed significant associations between O_3 exposure and abnormal risk during mid-to-late gestation, with the strongest effects observed in the third trimester. Each 10 μg/m^3 increase in O_3 concentration during mid-late gestation was associated with 2.0% (95% confidence interval (CI): 1.001-1.039) and 2.1% (95% CI: 1.000-1.041) elevated risks for neutrophil and monocyte abnormalities, respectively. In contrast, the same increment in O_3 concentration during the first trimester was associated with odds ratios of 0.984 (95% CI: 0.972-0.996) for neutrophil and 0.982 (95% CI: 0.969-0.995) for

monocyte abnormalities."

Recent studies environmental pollutants, such as $PM_{2.5}$, exposure during pregnancy have similarly documented window-specific immunological effects. Specifically, $PM_{2.5}$ exposure during the third trimester (final three months before delivery) was found to exert the most pronounced impacts on maternal immune and epigenetic alterations - a finding consistent with our observation of significantly elevated risks of inflammatory abnormalities in the third trimester. Notably, this research study also observed that exposure during an earlier window (from early to mid-pregnancy) was associated with a significant decrease in four inflammatory cytokines (IL-1RA, IL-8/CXCL8, IL-18, and MIF). Crucially, this study provided compelling evidence that these effects are pregnancy-specific, as identical $PM_{2.5}$ exposures in non-pregnant individuals induced upward trends in these cytokines[1]. These findings, together with our observations, suggest that environmental pollutant effects on the immune system may critically depend on the maternal physiological stage at exposure. The first trimester represents a critical window for establishing maternal-fetal immune tolerance, characterized by intricate immunological adaptations that shift the maternal immune profile toward regulation and tolerance rather than inflammatory responses[2]. Our findings may indicate that O_3 exposure during this specific period modulates the intrinsic maternal immune adaptation mechanisms, thereby mediating reduced risks of neutrophil and monocyte abnormalities. These observations collectively underscore the dynamic immunological plasticity of the maternal system during pregnancy, which appears to recalibrate immune homeostasis to minimize the risk of adverse pregnancy outcomes.

Furthermore, we have expanded the Discussion section (lines 678-688) to address this point: "First, epidemiological evidence from our cohort study indicates that exposure to O_3 during the third trimester of pregnancy is associated with enhanced maternal inflammatory activation. This finding is consistent with previous research on $PM_{2.5}$, suggesting that the third trimester represents a critical window during which environmental exposures influence maternal immunity. Interestingly, $PM_{2.5}$ exposure during the early to mid-stages of pregnancy was linked to reduced levels of inflammatory cytokines, a trend that parallels our observations concerning the effects of O_3 exposure in the first trimester. These stage-specific findings highlight the dynamic nature of gestational immune programming. This changes from an immune-tolerant state in early gestation to a highly controlled, physiologically regulated state of activation in late gestation."

Reference:

1. Jung YS, Aguilera J, Kaushik A, Ha JW, Cansdale S, Yang E, Ahmed R, Lurmann F, Lutzker L, Hammond SK, Balmes J, Noth E, Burt TD, Aghaeepour N, Waldrop AR, Khatri P, Utz PJ, Rosenburg-Hasson Y, DeKruyff R, Maecker HT, Johnson MM, Nadeau KC. Impact of air pollution exposure on cytokines and histone modification profiles at single-cell levels during pregnancy. Sci Adv. 2024 Nov 29;10(48): eadp5227.
2. Alippe Y, Hatterschide J, Coyne CB, Diamond MS. Innate immune responses to pathogens at the maternal-fetal interface. Nat Rev Immunol. 2025 Dec;25(12):869-884.

Reviewer:

I'm not convinced by the conclusion "collectively, … whereas 1 ppm O_3" appear to elicit immunosuppressive effects" (lines 158-161). In my opinion, cytometry results presented in figure 1 did not allow to affirm such a conclusion. Moreover, were cytokines assessed in dams lung tissue or blood? How do you explain differences in anti-Ova IgG levels between groups in the passive anaphylaxis model, since same doses of anti-IgG OVA should have been administered to mice! (lines 193 and following)?

Authors:

We sincerely thank the reviewer for their critical comment. We agree that the original wording may have overinterpreted the flow cytometry data. Accordingly, we have removed this inappropriate statement. To further substantiate the immune profiles observed, we assessed a panel of inflammatory cytokines in both maternal serum and lung tissue at E18.5. The results showed that in both serum and lung homogenates, the concentrations of the pro-inflammatory cytokines IFN-γ, TNF-α, and IL-1β were significantly elevated in the 0.5 ppm O_3 exposure group compared to the control. Notably, these markers did not show a similar pronounced increase with 1 ppm O_3 exposure (Fig. 14 in the response to reviewers). This pattern of cytokine release reinforces the conclusion that 0.5 ppm O_3 triggered a more pronounced pro-inflammatory state, while the response at the higher dose was comparatively attenuated. We have incorporated these new findings into the revised manuscript to provide a more comprehensive basis for our interpretation (lines 173-177).

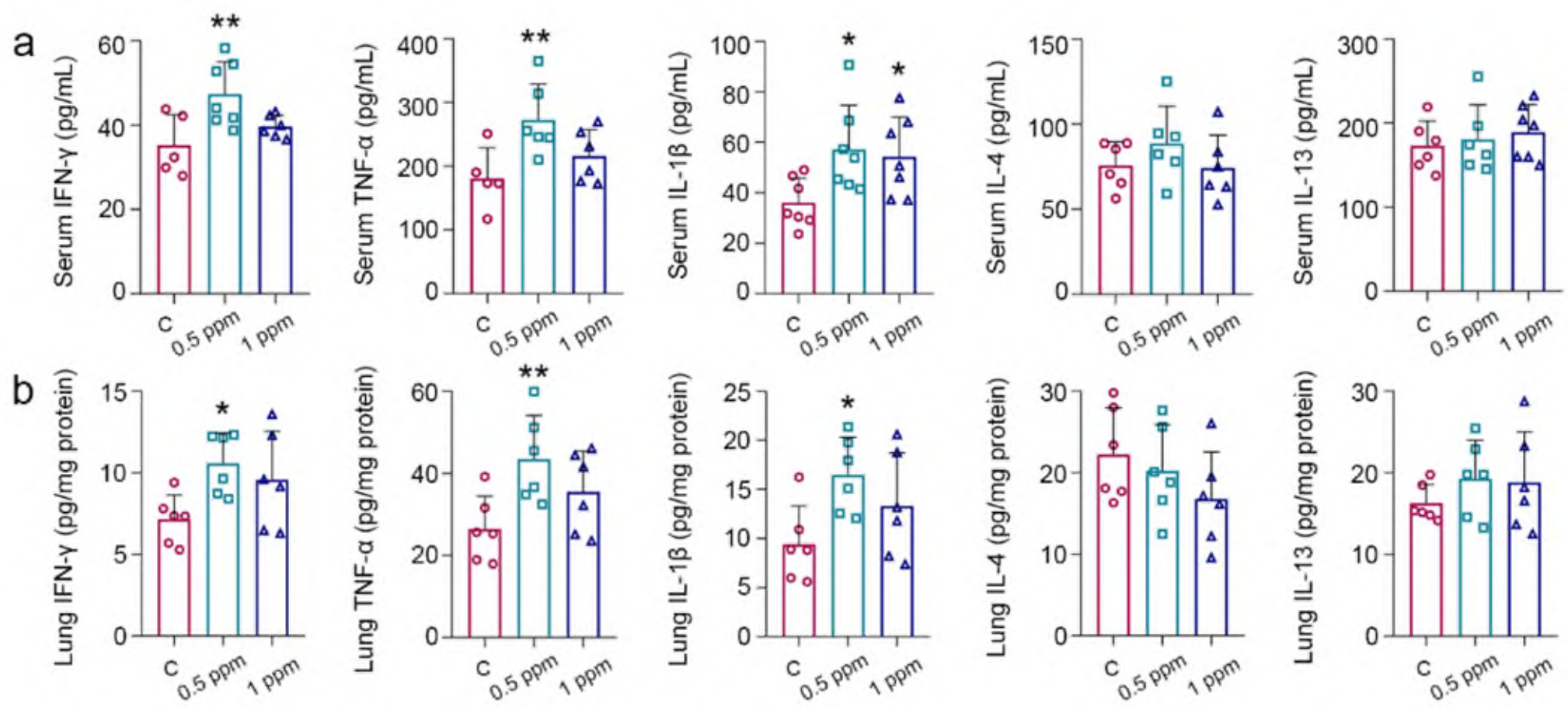

Fig. 14. Inflammatory cytokine profiles in maternal serum and lung tissue at E18.5. **a** Concentrations of inflammatory cytokines (IFN-γ, TNF-α, IL-1β, IL-4, and IL-13) in maternal serum at E18.5 (n = 5-6). **b** Levels of the same panel of inflammatory cytokines (IFN-γ, TNF-α, IL-1β, IL-4, and IL-13) in maternal lung tissue homogenates at E18.5 (n = 6). Data were presented as mean ± SD. Statistical analysis was performed by one-way ANOVA. *$p < 0.05$ and **$p < 0.01$ compared with the corresponding control group.

Regarding the differences in anti-OVA IgG levels among groups in the passive anaphylaxis model, we sincerely thank the reviewer for raising this critical question, which has allowed us to clarify an important aspect of our passive anaphylaxis model. The reviewer's observation is correct: all mice received an identical intravenous dose of pre-formed OVA-IgG1 immune complexes. Therefore, the observed inter-group

differences in serum anti-OVA IgG levels (e.g., the 1.91-fold increase in the PLA group) do not reflect variations in the administered dose but rather differences in their post-metabolic circulating concentrations. We interpret these findings as evidence that maternal O_3 exposure alters the *in vivo* handling of IgG-ICs in the offspring—likely affecting their clearance kinetics, tissue distribution, or stability—leading to varied residual levels at the time of measurement. This altered pharmacokinetics represents a biologically meaningful consequence of prenatal immune reprogramming. Importantly, the functional outcome (hypothermia) correlated with these circulating IgG-IC levels, supporting the conclusion that maternal exposure exacerbates anaphylactic responses in offspring via modified IgG-IC dynamics. To prevent any misunderstanding, we have revised the relevant section in the manuscript (lines 209-214 in the revised manuscript) to explicitly state that all mice received the same dose of IgG-ICs and that the reported anti-OVA IgG levels reflect post-injection circulating concentrations, which may differ due to altered pharmacokinetics rather than the injected amount. The revised text now reads: "Similarly, following intravenous administration of an identical dose of OVA-IgG immune complexes, post-metabolic circulating anti-OVA IgG levels measured post-injection were 1.91-fold higher in the PLA group than in the PCA group (Fig. 1o, p in the revised manuscript), suggesting altered IgG-IC pharmacokinetics in these offspring, this might result from the reprogramming of immune effector functions during embryonic development."

Reviewer:

Moreover, if I correctly understand, each lectin probe included triplicate technical replicate (lines 883—884), meaning that figure f and g show results from these technical replicates, and not that of individual mice with n=3. You can not perform statistical analysis in those conditions and draw conclusion that glycosylation patterns form dams and males/females offspring are different (all the more considering the high heterogeneity within technical triplicates!).

Authors:

We sincerely appreciate the reviewer's careful review and comments regarding our lectin microarray experiments. We acknowledge that the original statement "Each lectin probe included triplicate technical replicates" in lines 883-884 was indeed ambiguous and could potentially lead to misunderstanding of our experimental approach. We sincerely apologize for this lack of clarity.

The triplicate spots for each lectin probe on the microarray represent technical replicates of the same biological sample. This technical replication allows us to assess measurement precision within each sample. In addition to these technical replicates, we also performed biological replicates (with data from 3 mice per group). Each biological replicate (each mouse) generated a single dataset after processing the triplicate technical replicates through our established data analysis pipeline. Fig. 3f and 3g display results from biological replicates rather than technical replicate values. For example, the representative image in the figure below displays the chip scan and the corresponding lectin layout for the three biological replicates from the E18.5 control group, with each lectin printed in triplicate spots (technical replicates) (Fig. 15 in the response to

reviewers).

We have carefully revised the methodological description in the manuscript to improve its rigor and clarity. In the Methods section under "Lectin Microarray Profiling of IgG Glycosylation" (lines 1187-1200 in the revised manuscript), we have modified the text to read: "Each group consisted of three biological replicate samples. For each biological sample, lectin binding signals were measured in triplicate on the microarray. The lectin microarray data were processed using GenePix Pro 6.0 to extract the foreground signal intensity (F532, representing the median signal foreground value at 532 nm channel) and background signal intensity (B532, the median signal background value at 532 nm channel) for all lectin microarray spots. For the extracted data, background correction was performed to eliminate signal non-uniformity caused by inconsistent background values among different lectin spots by calculating the foreground-to-background difference (F-B) for each lectin spot. To eliminate errors from nonspecific lectin signals, the F-B values of blank controls (control) were subtracted from the F-B values of each lectin spot, and the SND was defined based on this calculation. For each sample, the mean value of technical replicate spots was calculated for each lectin. Differentially reactive lectins between biological samples were identified using threshold criteria (fold change > 1.2, $p < 0.05$)."

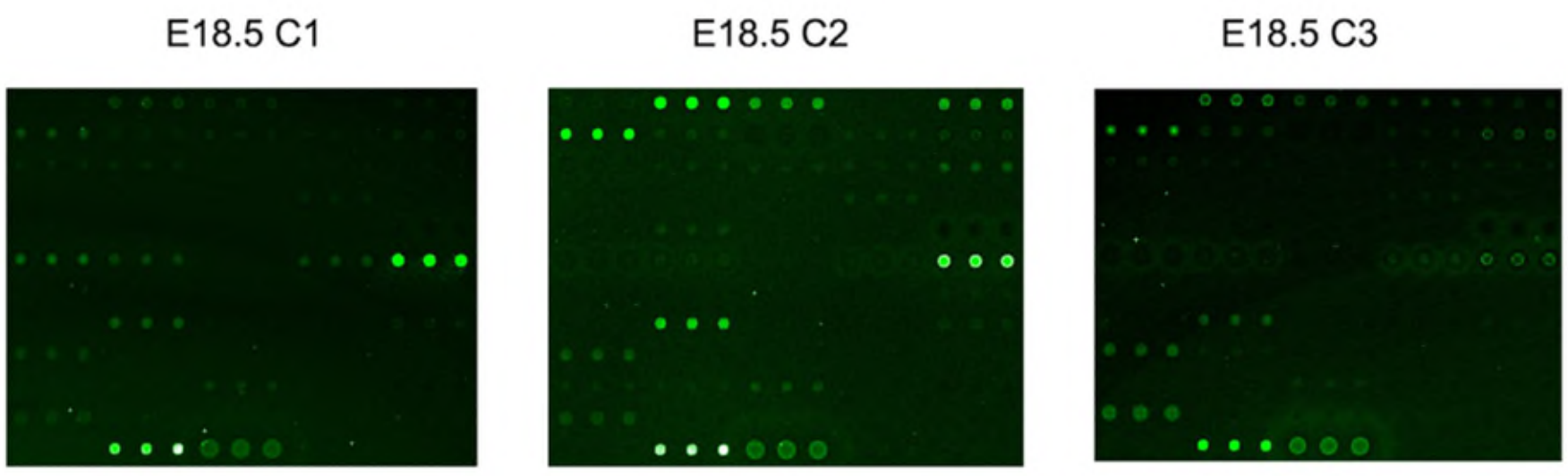

LTL	PSA	LCA	UEA I	AAL
LAL	TL	MAA	SNA	SSA
MAL II	SNA-I	NPL	ConA	GNL
HHL	CALSEPA	AMA	MNA-M	VFA
VVA mannose	ASA	GSL II	LEL	STL
UDA	PWM	WGA	DSL	HPA
VVL	DBA	SBA	PTL I	WFA
CSA	Black bean crude	GSL-IA4	IRA	IAA
HMA	GHA	MNA-G	RCA I	GSL I-B4
EEL	PNA	BPL	ABA	Jacalin,AIA
ACL	MPL	PHA-L	PHA-E	ECL
MAL I	CY3	CY5	Printing buffer	Printing buffer

Fig. 15. Representative lectin microarray scan for E18.5 control samples (three biological replicates) and corresponding lectin layout. Each lectin was printed at three independent spots (i.e., technical replicates).

Reviewer:

Were IgG concentrations and glycosylation assessed in milk from mothers at PND14?

Authors:

We agree with the reviewer on the importance of assessing IgG levels in breast

milk. In response to this suggestion, we supplemented our analysis by measuring the concentrations of total IgG and its subclasses in breast milk collected at postnatal day 14 (PND14), and these data have been incorporated into our revised manuscript (lines 353-354). The results showed no significant differences in total IgG, IgG1, IgG2a, or IgG2b between the control and O_3-exposed dams. Only the level of IgG3, which is present in very low abundance, was reduced in the breast milk of exposed dams compared to controls (Fig. 16 in the response to reviewers).

Regarding glycosylation, we acknowledge that direct analysis of IgG glycosylation in breast milk might provide further insight into the local immune environment. We also tried to check the glycosylation level of IgG in breast milk as your suggestion. However, the collected mouse breast milk cannot meet the technical standards for detecting glycotypes using lectin chips. Meanwhile, our study aims to assess the impact of maternal exposure on offspring immune function through changes in antibody glycosylation. Therefore, our glycosylation analysis primarily focuses on two key sites. 1) Maternal serum collected at E18.5 was analyzed as the primary source of IgG subsequently transferred to the fetus and neonate; alterations here represent the initial "source" modifications induced by O_3 exposure. 2) Offspring serum collected at PND14 was examined as the most direct functional indicator of the offspring's systemic immune status, since IgG glycosylation patterns are closely linked to disease susceptibility. In this study, although the levels of IgG and its subclasses in breast milk remained unchanged, we detected significant exposure-related alterations in IgG glycosylation in both maternal and offspring serum, which were closely associated with immune reprogramming in the offspring. We propose that this systemic reprogramming of IgG glycosylation—initiating at the maternal site and manifesting in the offspring—constitutes a key mechanism for the increased allergic susceptibility.

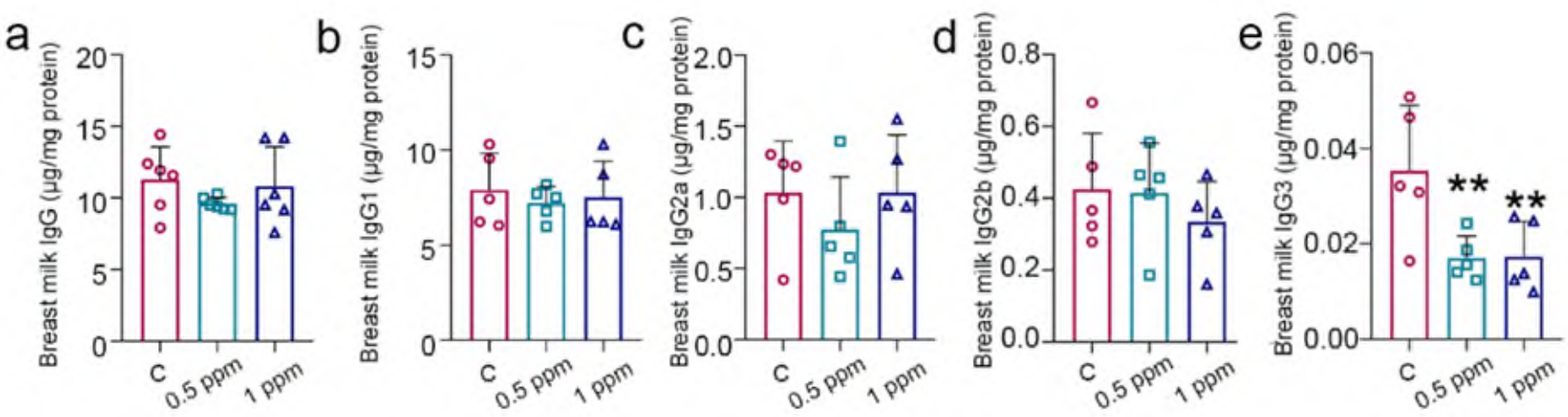

Fig. 16. Concentrations of total IgG and IgG subclasses in breast milk collected at PND14. **a** Levels of total IgG (n = 6). **b** Levels of the IgG1 subclass (n = 5). **c** Levels of the IgG2a subclass (n = 5). **d** Levels of the IgG2b subclass (n = 5). **e** Levels of the IgG3 subclass (n = 5). Data were shown as the mean ± SD. Statistical analysis was performed by one-way ANOVA. **$p < 0.01$ compared with the corresponding control group.

Minor points

Reviewer:

Introduction: Revise sentences lines 56-59 and add ref Lines 70-72: please specify at what PND transfer of IgG from mother to infant via milk end (both in human and mice). Lines 72; please clarify the sentence “IgG play a fundamental role in opsonizing innate immune cells… to enhance their function.”

Authors:

We thank the reviewer for this comment. In the revised manuscript (lines 72-75), we have added the following sentence: "In mice, this lactogenic IgG transfer, mediated by the neonatal Fc receptor (FcRn) in the intestine, continues until approximately postnatal day 16 (PND16), whereas in humans, it persists throughout breastfeeding, providing sustained mucosal protection."

In the revised manuscript (lines 75-78), we have rephrased the sentence to: "As a pivotal component of neonatal immunity, maternally-derived IgG engages innate immune cells to instruct functional responses, such as enhanced phagocytic capacity and broader immunomodulation." We have also added corresponding references to support this statement.

Reviewer:

Methods & results

Please provide a ref for the clinical reference ranges considered (lines 675-679), and the time of blood collection for mothers (at delivery??)

Authors:

We sincerely appreciate the reviewer's careful examination and kind reminder regarding these important methodological details. We apologize for our oversight in not originally citing the reference standards for hematological parameters. As suggested, we have now explicitly stated the reference standard in the Methods section (lines 925-926 of the revised manuscript) as follows: "Hematological reference ranges followed the Chinese Health Industry Standard (WS/T 405-2012) for blood cell analysis." Regarding the timing of maternal blood collection, all samples were obtained during the third trimester (gestational weeks 28-32).

Reviewer:

How euthanasia of pregnant dams was performed (please be more precise that “humanely euthanized”)?

Authors:

We sincerely appreciate the reviewer's comments regarding the need for more precise description of our euthanasia protocol. All animal procedures in this study were approved by the Committee of Scientific Research at Shanxi University and strictly followed the AVMA Guidelines for the euthanasia of animals[1]. In consist with previous study[2], we have now provided a more detailed description in the revised manuscript (lines 971-975): "Mice were humanely euthanized by cervical dislocation under deep isoflurane anesthesia (using an ABS small animal anesthesia system from Shanghai Yuyan Instruments Co., Ltd.), and neonates were sacrificed by decapitation. Death was confirmed by cessation of cardiac and respiratory activity prior to any subsequent analyses." This protocol ensures that the procedure was conducted in a painless and ethical manner while maintaining scientific rigor.

Reference:

1. LEARY S, UNDERWOOD W, ANTHONY R, et al. AVMA Guidelines for the euthanasia of animals: 2020 Edition. Schaumburg: American Veterinary Medical Association, 2020.

2. Erickson JJ, Archer-Hartmann S, Yarawsky AE, Miller JLC, Seveau S, Shao TY, Severance AL, Miller-Handley H, Wu Y, Pham G, Wasik BR, Parrish CR, Hu YC, Lau JTY, Azadi P, Herr AB, Way SS. Pregnancy enables antibody protection against intracellular infection. Nature. 2022 Jun;606(7915):769-775.

Reviewer:
Please specify the number of mice of both sex at each time point in progeny (i.e. at PND14 and PND56 for pulmonary testing and tissue collection), the same for mice submitted to systemic allergic reaction model.
Authors:
Thank you for raising this important point. Below is the detailed sex-specific breakdown of the progeny numbers at each analyzed time point: at PND14, including 80 females and 69 males; at PND28, including 36 females and 31 males; at PND42, including 37 females and 27 males; and at PND56, including 32 females and 26 males. For the systemic allergic reaction model conducted at PND56, a total of 332 female and 295 male offspring were included in the analysis.

Reviewer:
Please mention if samples from mice were treated individually or as pools (i.e. for flow cytometry analysis, ELISA, RT PCR, IgG-glycosylation assessment…)
Authors:
We sincerely appreciate the reviewer's inquiry regarding sample processing methodology. We confirm that all analyses in this study were performed on individual biological replicates, with no pooling of samples across animals. In the revised manuscript (lines 981-982), we have added the following statement in the Methods section: "All samples were processed and analyzed individually (no pooling across animals) to maintain biological independence." Additionally, we have clearly indicated this in the relevant figure legends.

Reviewer:
I’m not convinced pentobarbital is the best anaesthesia to perform lung function assessment in mice?
Authors:
We thank the reviewer for the opportunity to clarify our experimental approach. Our study employed two complementary techniques for pulmonary function assessment, selected based on the age and developmental stage of the animals to ensure both data quality and animal welfare. To avoid invasive procedures in immature lungs, non-invasive pulmonary function in neonatal pups (at PND14) was assessed using unrestrained whole-body plethysmography (WBP; Tow-Int Tech system). This method is performed on conscious, freely moving animals.

Invasive pulmonary function analysis was conducted specifically in the adult ASA and PSA model mice, anesthetized animals (intraperitoneal sodium pentobarbital, 50 mg/kg) underwent tracheostomy with insertion of an 18-gauge tracheal cannula connected to the AniRes2005 system (v: 3.5, Beijing Bestlab Technology Co., Ltd.).

After carefully reviewing both the manufacturer's instructions for the AniRes2005 system and relevant literature using this equipment, we found that pentobarbital sodium is indeed the most commonly used anesthetic in published studies employing this specific pulmonary function analysis system. One study reported that "after anesthetized by intraperitoneal injection of pentobarbital (100 mg/kg), the mice were connected to a computer-controlled small animal ventilator through a tracheal tube according to the instructions of the manufacturer of Anires2005 pulmonary function analysis system (version 2.0, bestlab, China)"[1]. Another study described their protocol where "after exposure, mice were anaesthetized with pentobarbital sodium solution (1% g/mL, 8 μL/g) and connected to the AniRes2005-BigAnimal2 ventilator via Y-tubing"[2]. Similar methodologies have been reported in several other studies[3-7], including our own previous work where mice were anesthetized with 50 mg/kg pentobarbital sodium for lung function measurements using the same system[8]. The consistency of these protocols across multiple independent studies, along with the manufacturer's recommendations, supports the reliability of pentobarbital anesthesia for lung function measurements with the AniRes2005 system.

Reference:

1. Geng Y, Li L, Yan J, Liu K, Yang A, Zhang L, Shen Y, Gao H, Wu X, Noth I, Huang Y, Liu J, Fan X. PEAR1 regulates expansion of activated fibroblasts and deposition of extracellular matrix in pulmonary fibrosis. Nat Commun. 2022 Nov 19;13(1):7114.
2. Jiang M, Li D, Piao J, Li Y, Chen L, Li J, Yu D, Pi J, Zhang R, Chen R, Chen W, Zheng Y. Nrf2 modulated the restriction of lung function via impairment of intrinsic autophagy upon real-ambient PM2.5 exposure. J Hazard Mater. 2021 Apr 15;408: 124903.
3. Ma Y, Deng L, Ma P, Wu Y, Yang X, Xiao F, Deng Q. In vivo respiratory toxicology of cooking oil fumes: Evidence, mechanisms and prevention. J Hazard Mater. 2021 Jan 15;402: 123455.
4. Xiang M, Lu M, Quan J, Xu M, Meng D, Cui A, Li N, Liu Y, Lu P, Kang X, Wang X, Sun N, Zhao M, Liang Q, Le L, Wang X, Zhang J, Chen S. Direct in vivo application of induced pluripotent stem cells is feasible and can be safe. Theranostics. 2019 Jan 1;9(1):290-310.
5. Shenshen Wu, Yin L, Han K, Jiang B, Meng Q, Aschner M, Li X, Chen R. NAT10 accelerates pulmonary fibrosis through N4-acetylated TGFB1-initiated epithelial-to-mesenchymal transition upon ambient fine particulate matter exposure. Environ Pollut. 2023 Apr 1;322: 121149.
6. Song D, Song G, Niu Y, Song W, Wang J, Yu L, Yang J, Lv X, Steinberg H, Liu SF, Wang B. Ulinastatin activates haem oxygenase 1 antioxidant pathway and attenuates allergic inflammation. Br J Pharmacol. 2014 Oct;171(19):4399-412.
7. Wu Y, Pei C, Wang X, Wang Y, Huang D, Shi S, Shen Z, Li S, He Y, Wang Z, Wang J. Probiotics ameliorates pulmonary inflammation via modulating gut microbiota and rectifying Th17/Treg imbalance in a rat model of PM2.5 induced lung injury. Ecotoxicol Environ Saf. 2022 Oct 1;244: 114060.
8. Yue H, Yan W, Ji X, Gao R, Ma J, Rao Z, Li G, Sang N. Maternal Exposure of BALB/c Mice to Indoor NO2 and Allergic Asthma Syndrome in Offspring at Adulthood with Evaluation of DNA Methylation Associated Th2 Polarization. Environ Health Perspect. 2017 Sep 13;125(9):097011.

Reviewer:

What is the isotype of OVA-specific IgG used in the passive anaphylaxis model, and

the purity of both IgG and BSA injected i.v.? (line 750)

Authors:

We thank the reviewer for this comment. The OVA-specific IgG used in the passive systemic anaphylaxis model was of the IgG1 isotype, as demonstrated in previous mouse model studies by Ishikawa and colleagues, IgG1 has been identified as the key subclass mediating IgG-dependent systemic anaphylaxis[1]. The specific antibody used was the anti-OVA IgG1 (Clone F2-3.58, BioXcell, West Lebanon, NH, USA), which has also been employed in other studies, as reported by Shuo-Wang Qiao et al., for forming IgG immune complexes with OVA[2]. As for the purity of the reagents, the OVA-specific IgG had a purity > 95%, and the BSA used for injection was ≥ 96% pure. We have now explicitly included these experimental details in the revised manuscript (line 1000 and lines 1023-1024).

Reference:

1. Ishikawa R, Tsujimura Y, Obata K, Kawano Y, Minegishi Y, Karasuyama H. IgG-mediated systemic anaphylaxis to protein antigen can be induced even under conditions of limited amounts of antibody and antigen. Biochem Biophys Res Commun. 2010 Nov 26;402(4):742-6.
2. Qiao SW, Kobayashi K, Johansen FE, Sollid LM, Andersen JT, Milford E, Roopenian DC, Lencer WI, Blumberg RS. Dependence of antibody-mediated presentation of antigen on FcRn. Proc Natl Acad Sci U S A. 2008 Jul 8;105(27):9337-42.

Reviewer:

Multiplex assay：There is an issue concerning the assay, with a confuse methodology mixing MSD analysis (solid phase) and Luminex (bead assay) one. This has to be clarified.

Authors:

We appreciate the reviewer's vigilance in bringing this important technical clarification to our attention. We have carefully revised both the main text and supplementary materials to correct this error, changing the original "Meso Scale Discovery (MSD) analysis" designation to the accurate "Cytokine quantification by Luminex".

Reviewer:

Please translate rpm to xg, antibodies dilution to exact quantities (e.g. μg/ml for western blot, or μg/10^6 cells for cytometry) to ensure reproducibility of experiments Flow cytometry analysis: Please provide FMO and precise how many cells were acquired.

Authors:

We sincerely thank the reviewer for these valuable suggestions to enhance the rigor and reproducibility of the methodology. In response, we have revised the relevant sections of the manuscript. Centrifugation steps have been converted from revolutions per minute (rpm) to relative centrifugal force (×g), and all antibody dilutions have been updated to report precise working concentrations. Furthermore, in the flow cytometry section, we have now provided fluorescence-minus-one (FMO) controls and the number of cells acquired for analysis in the revised manuscript (Supplementary Fig. 21 in the revised manuscript). Specifically, FMO controls were performed for low-

abundance and continuously expressed antigens, including Ly6c, Siglec-F, F4/80, MHC-II, CD16/32, CD3, CD4, CD25, Foxp3, IFN-γ, IL-4, and IL-17, to ensure accurate gating (Fig. 17 in the response to reviewers). Regarding the FMO controls for inflammatory cells, they were gated and presented from the $CD45^+$ population, which is consistent with our sample processing strategy of selecting $CD45^+$ cells for downstream analysis. Similarly, for T-cell subsets, the gating was performed and displayed from viable cells. The number of cells analyzed is indicated in the figure legends.

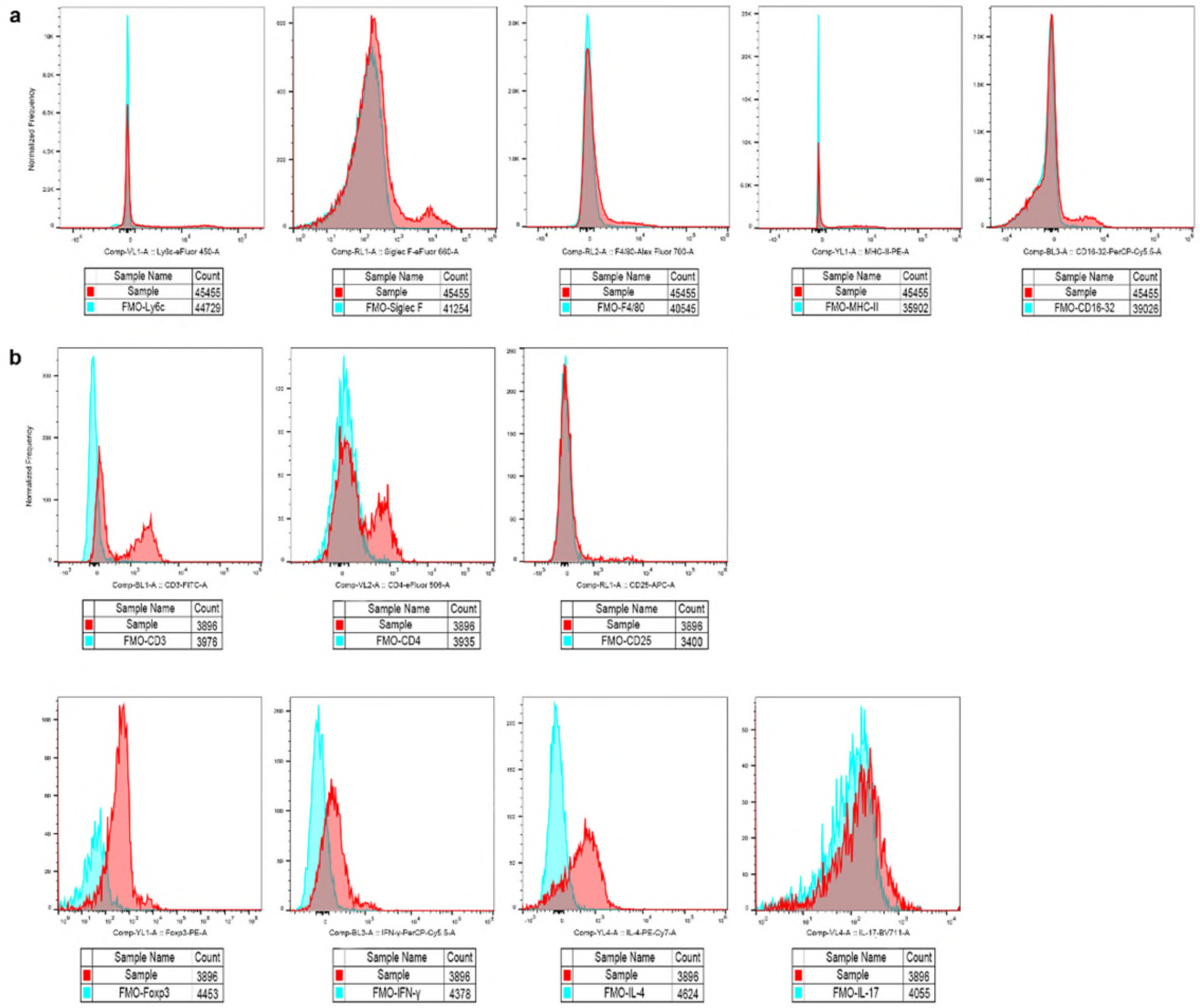

Fig. 17. Representative fluorescence-minus-one (FMO) control histograms for flow cytometry gating. **a** Histograms for inflammatory cell markers: Ly6c, Siglec-F, F4/80, MHC-II, and CD16/32. **b** Histograms for T cell markers: CD3, CD4, CD25, IFN-γ, Foxp3, IL-4, and IL-17.

Reviewer:

Figure 5h: for DSL fluorescence, C vs 0.5 ppm tend to be different, but this is not significant: please modify corresponding text (lines 412-413)

Authors:

We thank the reviewer for this observation. We agree that the comparison noted describes a trend, not a statistically significant difference. We have therefore revised the text to accurately reflect this (lines 511-513 in the revised manuscript).

In a word, we thank the reviewer for so many helpful suggestions and comments, which help improve our manuscript greatly.

Title: Elevated Ozone Activates Maternal Immunity to Drive Offspring IgG-Mediated Anaphylaxis through IgG Glycosylation Reprogramming

The point-by-point response to the Reviewers' comments

Reviewer 1 Comments for the Author

I have reviewed the revised version of the manuscript entitled "Elevated Ozone Activates Maternal Immunity to Drive Offspring Allergy through IgG Glycosylation Reprogramming" (Manuscript ID: NCOMMS-25-29897).

I am satisfied that the authors have carefully and thoughtfully addressed all of the concerns raised during the review process. The additional experiments and analyses meaningfully strengthen the study, particularly with respect to the proposed mechanistic link between prenatal ozone exposure, maternal IgG glycosylation, FcRn-mediated antibody transfer, and the sex-specific immune outcomes observed in the offspring. The expanded treatment of IgG subclass specificity, receptor interactions, and the human cohort analyses adds important clarity and depth to the work.

In its current form, the manuscript is clear, rigorous, and well balanced. It offers genuinely novel insight into how environmental exposures during pregnancy can influence immune development through antibody-mediated mechanisms, and it will be of broad interest to readers working in immunology, environmental health, and developmental biology.

I have no remaining concerns and believe the manuscript now meets the standards for publication in Nature Communications.

I therefore recommend acceptance of this manuscript.

Authors:

We sincerely thank the reviewer for the time and effort spent on evaluating our manuscript, and for the very positive and encouraging comments.

Reviewer 2 Comments for the Author

Reviewer:

Thank you for the revised responses. I have three remaining concerns.

Authors:

Thank you for reviewing our revised responses. We appreciate the opportunity to further improve our manuscript and have elaborated on the three remaining concerns as follows.

Response to comment

Reviewer:

(1) Thank you for providing the unadjusted logistic regression models for haematological abnormalities. However, my comment regarding haemoglobin was not that HGB should be included as an adjustment variable in those models, but rather that it should be evaluated directly as an endpoint to determine whether ozone-exposure is associated with altered HGB in the last pregnancy stage (the only stage for which HGB appears available).

Please therefore add an analysis of HGB vs exposure (restricted to the last pregnancy

stage), reporting group-wise HGB values (n and mean ± SD or median[IQR]), an effect estimate with 95% CI, and the statistical test used. This will allow readers to see whether HGB changes with exposure. Any HGB-adjusted logistic model may be retained as a sensitivity analysis, but it does not replace reporting whether HGB differs by exposure.

Authors:

We sincerely apologize for our earlier misunderstanding of your comment regarding the hemoglobin (HGB) analysis, and we greatly appreciate your further clarification. We fully agree with your valuable suggestion that evaluating HGB directly as an endpoint is essential for understanding the potential impact of O_3 exposure on iron metabolism during the last pregnancy stage.

Following your guidance, we have now conducted a direct analysis assessing the association between O_3 exposure and HGB concentrations in the third trimester (T3). Using the World Health Organization's diagnostic threshold for anemia during pregnancy (HGB < 110 g/L), we stratified the 5,299 pregnant women into two groups: those with normal HGB levels (≥ 110 g/L, n = 4,510) had a mean value of 124.57 ± 8.88 g/L, while those with abnormal HGB levels (< 110 g/L, n = 789) had a mean value of 102.22 ± 7.21 g/L. Binary logistic regression analysis, with Wald tests for statistical significance and profile likelihood-based confidence intervals, revealed that O_3 exposure in the third trimester was significantly associated with an increased risk of HGB abnormality: each 10 μg/m^3 increment in O_3 concentration was associated with a 3.0% increase in the risk of abnormal HGB (OR = 1.030, 95% CI: 1.012–1.048). As you mentioned earlier, O_3 not only alters glycan structures but may also affect HGB levels through interference with iron metabolism.

This iron metabolism/HGB pathway may coexist with the IgG glycan alterations identified in our study, together revealing the complex impact of O_3 on maternal health. This notion is supported by sensitivity analyses: even after adjusting for HGB, the significant associations between O_3 and other hematological parameters (neutrophils, monocytes, and fibrinogen) persisted. Specifically, for each 10 μg/m^3 increase in O_3 concentration during the mid-to-late pregnancy period (T2-T3), we observed a 2.0% elevated risk of neutrophil abnormalities (95% CI: 1.001-1.040) and a 2.1% increased risk of monocyte abnormalities (95% CI: 1.000-1.041). During the third trimester (T3), each 10 μg/m^3 increase in O_3 was associated with a 1.5% higher risk of neutrophil abnormalities (95% CI: 1.003–1.028), a 2.2% higher risk of monocyte abnormalities (95% CI: 1.008–1.036), and a 3.0% higher risk of fibrinogen abnormalities (95% CI: 1.011–1.050). Importantly, these effect estimates showed remarkable consistency with our initial unadjusted models, and the HGB covariate itself did not significantly contribute to the model fit. These results suggest that O_3 -induced oxidative stress may simultaneously affect multiple systems—directly influencing erythrocytes or iron metabolism while also activating the maternal immune system and remodeling IgG glycosylation, thereby impacting maternal-fetal immune transfer. These parallel mechanisms are not mutually exclusive and collectively contribute to the potential health effects of O_3 exposure.

Furthermore, we investigated the effects of gestational O_3 exposure on HGB levels

in our mouse model, examining both maternal mice and their offspring. The results showed no significant differences in HGB levels between the O_3-exposed and control groups, whether at the end of gestational exposure (E18.5) or during offspring development (PND14, PND56) and after allergen challenge. This cross-species discrepancy may be attributed to the nutritionally complete standard diet provided to the mice under experimental conditions, which effectively buffered against O_3 -induced iron depletion. In contrast, pregnant women in their third trimester naturally face peak physiological iron demands and diverse nutritional statuses, potentially rendering their iron metabolism more susceptible to O_3-induced oxidative stress and systemic inflammation[1, 2]. Additionally, differences in exposure timing, dosage, or metabolic responses may also contribute to these observations. These findings underscore the need for future research to further explore the specific mechanisms underlying O_3's effects on hematological systems across different species.

We have incorporated this HGB analysis into the Results section and expanded the Discussion to address these parallel mechanisms. Specifically, in the Results section (revised manuscript, lines 139-146), we have added the following description: "As a supplementary finding, O3 exposure in the third trimester was also found to be significantly associated with an increased risk of hemoglobin (HGB) abnormality (OR = 1.030, 95% CI: 1.012-1.048 per 10 μg/m3 increment), with 4,510 pregnant women exhibiting normal HGB levels (124.57 ± 8.88 g/L) and 789 exhibiting abnormal HGB levels (102.22 ± 7.21 g/L) (Table 1). Crucially, to verify the robustness of these primary findings, sensitivity analyses including multi-pollutant models and adjustment for maternal HGB levels yielded highly consistent effect estimates." In the Discussion section (revised manuscript, lines 858-870), we have added the following statement: "While the primary focus of this study is on the role of IgG glycosylation modifications in O_3 exposure-affected maternal–fetal immune transfer, we have not excluded other plausible parallel mechanisms. For instance, previous studies have indicated that O_3 can interact with heme iron and disrupt iron homeostasis, thereby potentially affecting HGB levels. In our cohort study, we did observe a significant association between O_3 exposure in the third trimester and an increased risk of anemia, although adjusting for HGB as a covariate did not materially alter our primary findings. From a physiological perspective, the substantially increased iron demand during late pregnancy may render maternal iron metabolism more susceptible to O_3-induced oxidative stress and inflammation. This also suggests that gestational O_3 exposure may directly affect maternal health through disruption of iron metabolism, and future studies could further explore the underlying mechanisms of this pathway and its potential interactions with immune effects."

Reference:

1. Sangkhae V, Fisher AL, Ganz T, Nemeth E. Iron Homeostasis During Pregnancy: Maternal, Placental, and Fetal Regulatory Mechanisms. Annu Rev Nutr. 2023 Aug 21; 43: 279-300.
2. Sangkhae V, Fisher AL, Chua KJ, Ruchala P, Ganz T, Nemeth E. Maternal hepcidin determines embryo iron homeostasis in mice. Blood. 2020 Nov 5; 136(19): 2206-2216.

Reviewer:

(2) Statistical reporting: The manuscript still lists omnibus tests (e.g., ANOVA) without specifying the post-hoc procedure. Please state which post-hoc test(s) were used, whether comparisons were versus control only or all pairwise, and how multiplicity was controlled. Where relevant, indicate assumption checks and the alternative tests used if assumptions were violated.

Authors:

We thank the reviewer for this important methodological guidance. We have revised the "Statistical Analysis" section of the Methods (revised manuscript, lines 1281-1302). Data are presented as mean ± standard deviation (SD). Normality was assessed using the Shapiro-Wilk test, and homogeneity of variances was verified using Levene's test. For multi-group analyses, one-way ANOVA was performed. When both normality and homogeneity of variances assumptions were satisfied, Fisher's Least Significant Difference (LSD) test was applied for post-hoc pairwise comparisons. Statistical theory indicates that LSD procedure preserves the experimentwise type I error rate at the nominal significance level if, and only if, the number of treatment groups is three. This property stems from the two-step nature of the procedure: the global F-test in the first step provides protection against Type I error inflation, and when the number of groups is exactly three, the maximum number of non-orthogonal comparisons is limited to two, which does not exceed the treatment degrees of freedom (k-1). Consequently, under these conditions, no further adjustment for multiple comparisons is required to maintain the family-wise error rate at α[1]. Therefore, for analyses involving only three groups where comparisons were exclusively made between each exposure group and the control group, we did not apply additional multiplicity correction. For the allergy challenge model, we performed comparisons in the offspring with prenatal O_3 or clean air exposure versus their respective controls (ALA vs. ACA, AHA vs. ACA), in the saline groups versus their respective controls (ALS vs. ACS, AHS vs. ACS), and between the corresponding modeled and non-modeled groups (ACA vs. ACS, ALA vs. ALS, AHA vs. AHS). To control for multiple testing in these cases, we applied the Benjamini-Hochberg false discovery rate (FDR) procedure to the raw *p*-values obtained from the LSD tests. When homogeneity of variances was violated after data transformation, we used Tamhane's T2 tests for post-hoc pairwise comparisons. These tests do not assume equal variances and inherently adjust for multiple comparisons to control the family-wise error rate. When data were not normally distributed, we first attempted data transformations such as logarithmic transformation. If the transformed data met the assumptions of normality and homoscedasticity, the parametric procedures described above were applied. If, after transformation, the data remained non-normal, we employed non-parametric methods, assessing overall group differences using the Kruskal-Wallis test, followed by Dunn's post-hoc test for pairwise comparisons. As with the parametric analyses, multiplicity was controlled using the Benjamini-Hochberg FDR procedure. For two-group comparisons, the Student's t-test was applied when both normality and homogeneity of variances assumptions were satisfied; when variances were unequal but normality held, Welch's t-test was used. All analyses were performed using SPSS (v: 26.0) and

statistical significance was defined as $p < 0.05$. Following the reviewer's suggestion, we have re-analyzed all multi-group comparisons in the allergy challenge model groups with correction for multiple testing. Applying the Benjamini-Hochberg correction did not alter the main conclusions of the manuscript, and the results presented in the figures now reflect the BH-adjusted *p*-values. Specific statistical tests and comparisons are also indicated in the figure legends.

Reference:

1. Meier U. A note on the power of Fisher's least significant difference procedure. Pharm Stat. 2006 Oct-Dec; 5(4): 253-63.

Reviewer:

(3) Mechanistic support for IgG glycosylation: I understand the stated technical limitation preventing in vitro generation of defined IgG glycoforms. However, the current data remain largely associative. To strengthen the claim of a direct and meaningful contribution to anaphylaxis, it would be helpful to isolate/enrich the specific glycosylated IgG fraction from ozone-exposed mice and test its anaphylactic potential (or Fc-dependent effector activity) compared with an appropriate control fraction, with glycoform composition verified analytically and IgG dose/subclass matched. If such experiments are not feasible, the mechanistic claims should be tempered accordingly.

All other points appear adequately addressed.

Authors:

We thank the reviewer for the valuable experimental suggestions regarding the mechanistic support for IgG glycosylation. We consulted with BCBIO Biotechnology Co., Ltd. (Guangzhou, China; specializes in antibody isolation and glycosylation analysis), GlycanLink Biomedicine Co., Ltd. (Shanghai, China), and glycochemistry expert Prof. Feng Tang from the Shanghai Institute of Materia Medica (Shanghai, China). We learned that isolating or enriching GlcNAc-modified IgG glycoforms from O_3-exposed mice presents substantial technical challenges. Our collaborators at BCBIO previously attempted to isolate glycoform-specific IgG fractions using lectin-coated columns that recognize specific sugar moieties. However, the quantity and purity of the isolated fractions were insufficient for downstream functional assays, including anaphylaxis induction or Fc-dependent effector activity testing. Currently, methods with sufficient glycan resolution, such as LC-MS-based analytical techniques, are suitable for detection but cannot achieve preparative isolation of IgG fractions with specific glycoforms[1]. We have incorporated this technical limitation into the Discussion section of the revised manuscript (lines 894-901): "However, several limitations require further investigation: studies employing IgG deficient or FcRn deficient dams would be valuable to establish definitive causal evidence for the role of glycosylated maternal IgG in this intergenerational transmission; due to current technical limitations in isolating specific IgG glycoforms, studies generating GlcNAc-modified mouse IgG are needed to directly test its contribution to IgG-mediated anaphylaxis susceptibility *in vivo*; and population-based studies are needed to validate the allergic relevance of MIA-

driven offspring IgG glycosylation reprogramming observed in murine models."

In addition, we have revised the wording of our conclusions in the manuscript accordingly, tempering the mechanistic claims. In the Abstract section of the revised manuscript (lines 41-43), we have modified the text to read: "In adulthood, although IL-17/TNF signaling subsided, its downstream transcription factors c-Jun/c-Fos directly activated *Mgat4a* to sustain GlcNAc glycosylation. This glycosylation of IgG was associated with enhanced IgG-FcγRIII affinity and more severe inflammatory responses during anaphylaxis." In the Results section of the revised manuscript (lines 575-578), we have modified the text to read: "These results suggest that the severe IgG-mediated anaphylaxis observed in male mice from the ALA group may be associated with *Mgat4a*-mediated N-acetylglucosamine glycosylation and a consequent enhancement in IgG's ability to activate FcγRIII." In the Results section of the revised manuscript (lines 602-606), we have modified the text to read: "Having observed that male offspring from the 0.5 ppm O_3-exposed dams (ALA group) exhibited enhanced IgG-mediated anaphylaxis, which was associated with increased *Mgat4a*-mediated GlcNAc modification and enhanced IgG-FcγRIII interaction, we sought to determine through which physiological route(s) this glycosylation-mediated programming is transmitted." In the Discussion section of the revised manuscript (lines 885-889), we have modified the text to read: "Our findings suggest that the enhanced IgG-mediated anaphylaxis observed in offspring following maternal O_3 exposure may be linked to MIA-induced alterations in neonatal IgG glycosylation. These alterations, through modulation of inflammatory pathways and *Mgat4a*-mediated GlcNAc glycosylation, shape immune reprogramming in the offspring, accompanied by enhanced IgG binding to FcγRIII on effector cells."

Reference:

1. Falck D, Wuhrer M. GlYcoLISA: antigen-specific and subclass-specific IgG Fc glycosylation analysis based on an immunosorbent assay with an LC-MS readout. Nat Protoc. 2024 Jun; 19(6): 1887-1909.

Reviewer 3 Comments for the Author

Reviewer:

Dear authors,

Thanks for taking into consideration all points raised by the reviewers, and for carefully answering each remark - either by clarifying the text or by providing additional experiments.

Authors:

We thank the reviewer for the positive feedback and invaluable comments. We are pleased that our revisions and explanations have met with your approval.

Response to comment

Reviewer:

Two points for the reviewed manuscript:

- I would really prefer the authors used "IgG-mediated anaphylaxis" instead of "allergic reaction"

- what is the rational for assessing anti-OVA IgE in the passive anaphylaxis model!!!! Results may correspond to non-specific signals and should be removed!

Authors:

We thank the reviewer for the helpful suggestions. We have revised the manuscript by replacing "allergic reaction" with "IgG-mediated anaphylaxis" throughout the text.

We agree with the reviewer's concern regarding the assessment of anti-OVA IgE in the passive anaphylaxis model. Given that this model is mediated by IgG rather than IgE, the detection of anti-OVA IgE may indeed reflect non-specific signals. Accordingly, we have removed these results from the manuscript.

Overall, we are grateful to the reviewers for the numerous insightful comments, which have significantly strengthened our work.
